# Systematic benchmarking of multi-ancestry fine-mapping under variant heterogeneity and LD mismatch

**DOI:** 10.1093/bib/bbag574

**Published:** 2026-10-19

**Authors:** Guangxin Chen, Peristera Paschou, Boran Gao

**Affiliations:** Department of Biological Sciences, Purdue University, 915 Mitch Daniels Boulevard, West Lafayette, IN 47907, United States; Department of Biological Sciences, Purdue University, 915 Mitch Daniels Boulevard, West Lafayette, IN 47907, United States; Department of Biological Sciences, Purdue University, 915 Mitch Daniels Boulevard, West Lafayette, IN 47907, United States; Department of Statistics, Purdue University, 150 N. University Street, West Lafayette, IN 47907, United States

**Keywords:** multi-ancestry fine-mapping, GWAS, statistical fine-mapping, linkage disequilibrium, benchmarking, population genetics

## Abstract

Increasingly diverse, multi-ancestry genome-wide association studies (GWASs) provide unprecedented opportunities to better understand the genetic architecture of complex traits. In particular, differences in linkage disequilibrium (LD) patterns across populations can be leveraged to improve the resolution and reliability of statistical fine-mapping. However, cross-ancestry effect heterogeneity, population-specific variant support, and LD mismatches pose substantial challenges, motivating a systematic evaluation of multi-ancestry fine-mapping methods to provide practical guidance for analyses across diverse populations. We conducted a comprehensive benchmarking of nine methods, spanning joint probabilistic models that explicitly model cross-ancestry LD and effect sharing, and univariate-based integration approaches that aggregate ancestry-specific results. We evaluated fine-mapping accuracy, robustness, and computational efficiency using realistic simulations derived from UK Biobank genotype data, with real-data validation in the *All of Us* Research Program. Our study revealed that joint modeling approaches achieve improved power and resolution for causal variants shared across populations. In comparison, *post-hoc* integration is more robust in recovering causal variants present in only a subset of populations by accommodating differences in variant availability. Moreover, methods are generally underpowered in the presence of systematic discrepancies between GWAS summary statistics and external LD reference panels. Motivated by these complementary performance profiles, we propose the multi-ancestry fine-mapping decision strategy, which avoids a priori method selection by adaptively selecting approaches based on locus-level evidence of ancestry-specific variants. Together, our benchmarking and decision strategy offer practical guidance for robust multi-ancestry fine-mapping and highlight the importance of joint modeling that effectively accounts for ancestry-specific variant availability and LD mismatch.

## Introduction

Large-scale biobanks now provide unprecedented opportunities to dissect the genetic architecture of complex traits across diverse human populations. Whereas early genome-wide association studies (GWASs) were largely restricted to European-ancestry cohorts, contemporary efforts increasingly leverage multi-ancestry datasets comprising millions of participants and thousands of phenotypes [1–5]. This expansion enhances representation and statistical power, offering the potential for more equitable genomic discovery. Yet despite these advances, systematically identifying the biological mechanisms underlying trait-associated loci remains a central challenge in human genetics.

A central obstacle arises because most variants identified in GWAS are not themselves causal but are correlated with causal variants through linkage disequilibrium (LD) [6, 7]. Statistical fine-mapping seeks to resolve this uncertainty by estimating the posterior probability that each variant is causal, thereby refining association signals into a smaller set for functional interrogation [8–10]. In principle, incorporating data from multiple ancestries can improve fine-mapping resolution by leveraging differences in LD structure across populations [11–13].

However, multi-ancestry fine-mapping is inherently complicated by biological and statistical heterogeneity across populations. Although many causal variants appear to have effects across ancestries, accumulating evidence indicates that ancestry-specific contributions meaningfully shape complex trait variation [14–16]. Crucially, ancestry-specific signals do not arise from a single underlying mechanism. Different sources of cross-ancestry heterogeneity have distinct biological and statistical implications. While recent methods model cross-ancestry and ancestry-specific signals, the inferential consequences of different forms of ancestry heterogeneity remain unclear.

To clarify the origins of cross-ancestry heterogeneity, we consider two conceptually distinct categories of ancestry-specific causal signals. The first comprises variants that are polymorphic in certain populations but monomorphic in others. We refer to these as ancestry-specific segregating variants (AS-SVs), reflecting their population-restricted allele presence and their contribution to trait variation only within those groups. AS-SVs arise from population-specific evolutionary histories, including demographic events and local adaptation, but their empirical identification may also be influenced by differences in imputation quality, sequencing coverage, or quality-control filtering across cohorts [13, 17]. Well-characterized examples include the missense variant rs28362263 in *PCSK9*, present predominantly in individuals of African ancestry and influencing low-density lipoprotein cholesterol levels, as well as the damaging missense variant rs730881101 in *TNNT2*, associated with increased risk of heart failure in East Asian populations [13, 18].

The second category includes variants that are present across ancestries but exhibit ancestry-dependent effects. We define these as ancestry-specific contextual variants (AS-CVs), emphasizing that their causal impact depends on ancestry-specific genetic backgrounds, environmental exposures, or gene–gene interactions rather than allele presence alone. For example, the missense variant rs58542926 in *TM6SF2* is observed in both European and African populations but shows a detectable effect on lipid traits primarily in individuals of European ancestry [12]. Distinguishing AS-SVs from AS-CVs is essential: the former reflects population-restricted genetic architecture, whereas the latter reflects context-dependent effects of variants present across ancestries. Both have direct implications for the transferability of genetic findings and for understanding population-specific disease mechanisms [13, 19]. For comparison, we refer to variants that are present and causal in all ancestries under study as cross-ancestry causal variants. Together, these three categories provide a framework for systematically evaluating how causal sharing and distinct sources of cross-ancestry heterogeneity affect model calibration, power, and the interpretation of posterior inclusion probabilities across populations.

Existing multi-ancestry fine-mapping methods integrate cross-population data using two broad strategies (Fig. 1). Joint modeling approaches simultaneously analyze association signals across ancestries within a unified probabilistic framework. Representative methods, including Paintor [20], SuSiEx [11], MultiSuSiE [21], MESuSiE [12], XMAP [22], and CARMA-X [23], differ in how they represent multiple causal signals and model cross-ancestry effect heterogeneity. Despite these differences, joint approaches commonly assume some degree of shared causal structure and are typically applied to the intersection of variants observed across ancestries [12, 22]. As a result, AS-SVs may be excluded from consideration, and posterior probability may be redistributed toward cross-ancestry candidates.

**Figure 1:**
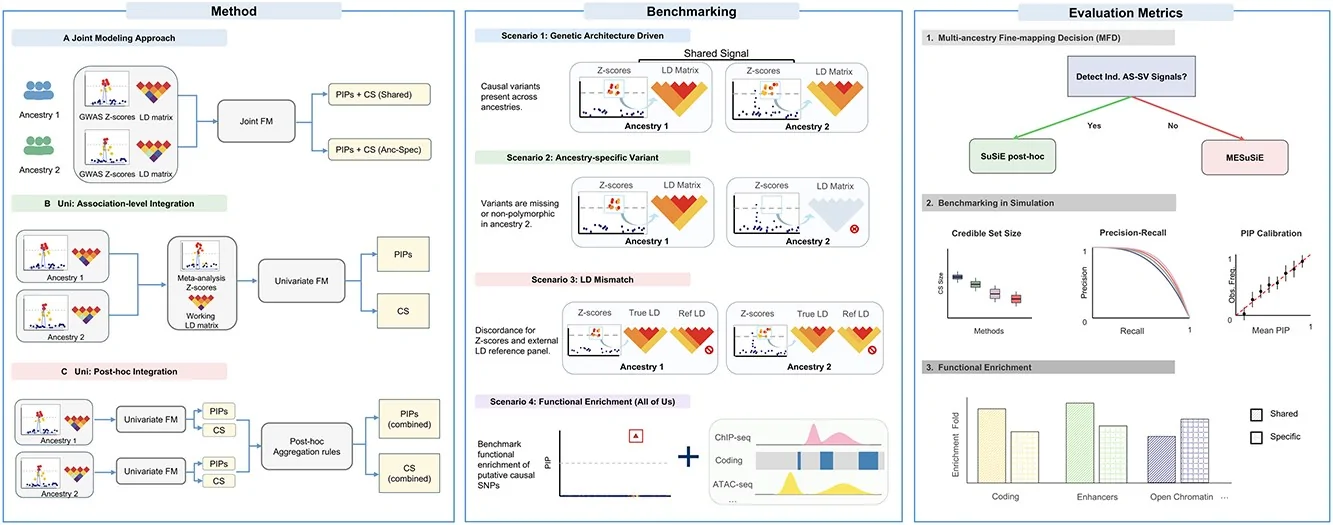
Overview of multi-ancestry fine-mapping evaluation. The study is organized into three components. Left, multi-ancestry fine-mapping strategies are grouped into three classes: joint modeling, which jointly analyzes ancestry-specific Z-scores and LD matrices to infer cross-ancestry and ancestry-specific signals; association-level integration, which meta-analyzes association statistics and applies univariate fine-mapping using a combined LD matrix; and *post-hoc* integration, which performs ancestry-specific univariate fine-mapping separately and then combines the results. Middle, method performance is evaluated across four scenarios: genetic architecture-driven simulations, ancestry-specific variant simulations, LD mismatch simulations, and functional enrichment analysis in All of Us. Right, we present the multi-ancestry fine-mapping decision (MFD) strategy, simulation-based benchmarking metrics including credible set size, precision-recall, and PIP calibration, and functional enrichment of variants prioritized in the All of Us analysis.

In contrast, univariate-based approaches either perform univariate fine-mapping on meta-analyzed summary statistics or combine results from separate ancestry-specific fine-mapping analyses through *post-hoc* integration [15, 24–27]. Association-level integration combines association statistics and constructs a single LD representation across ancestries, potentially reducing ancestry-resolved LD information. *Post-hoc* integration preserves population-specific LD structure and can accommodate AS-SVs more naturally, but does not explicitly borrow strength across populations for cross-ancestry signals. Although these strategies produce comparable outputs, including posterior inclusion probabilities (PIPs) and credible sets (CSs), they rely on distinct assumptions regarding variant sharing and LD compatibility.

Despite rapid methodological development, it remains unclear how these modeling assumptions interact with realistic forms of ancestry heterogeneity. In particular, whether joint frameworks can reliably distinguish ancestry-specific signals from cross-ancestry signals, whether univariate approaches sacrifice efficiency for robustness, and how posterior inference behaves when assumptions are violated have not been systematically characterized. Without a principled understanding of these interactions, it is difficult to determine which modeling strategy is appropriate for a given locus.

An additional and largely underappreciated challenge arises from LD mismatch. Because individual-level genotype data are often inaccessible, most large-scale multi-ancestry fine-mapping studies rely on GWAS summary statistics paired with external ancestry-matched LD reference panels [6, 8]. This framework implicitly assumes that the LD structure underlying the summary statistics is accurately represented by the reference panel. In practice, this assumption is frequently imperfect. Reference panels are typically constructed from smaller cohorts that may differ from GWAS populations in sequencing platform, imputation quality, ancestry composition, and sample size [23, 28, 29]. Such discrepancies introduce LD mis-specification that can bias PIPs and distort CSs [29, 30].

LD mismatch may be particularly consequential in multi-ancestry analyses. Integrating LD matrices across distinct populations can amplify discrepancies, increasing the risk of miscalibrated posterior probabilities and erroneous attribution of causal signals. Although LD outlier detection procedures have been proposed [28, 29], their performance under multi-ancestry integration has not been systematically evaluated. LD mismatch therefore represents a structural source of model mis-specification that may distort inference independently of biological ancestry heterogeneity.

Beyond the challenges posed by ancestry heterogeneity and LD mismatch, functional annotations can provide complementary biological evidence for distinguishing correlated variants with similar statistical support. Yet their direct integration into multi-ancestry fine-mapping remains limited. This issue is increasingly relevant as DNA sequence foundation models provide a growing source of variant-level functional information [31, 32]. It remains unclear how annotation informativeness, enrichment strength, and prior dimensionality affect multi-ancestry fine-mapping performance.

Here, we systematically examine how cross-ancestry effects, contextual ancestry-specific effects, segregating variants, and LD mismatch interact with modeling assumptions in multi-ancestry fine-mapping. Using biologically realistic simulations and real-world GWAS summary statistics from the *All of Us* Research Program, we characterize the conditions under which different modeling strategies succeed or fail. We quantify detection power, resolution, calibration, robustness to LD mis-specification, computational scalability, and functional enrichment across diverse genetic architectures. Alongside this core benchmark, we evaluate how annotation informativeness and prior specification affect multi-ancestry fine-mapping performance, including the use of an Evo2 sequence effect score [32] within the annotation panels. Based on the core benchmarking results, we propose the multi-ancestry fine-mapping decision (MFD) framework—a data-driven strategy that links observable locus-level characteristics to appropriate modeling approaches. Rather than introducing a new statistical model, MFD translates empirical insights into actionable decision rules for multi-ancestry fine-mapping. By explicitly aligning modeling assumptions with ancestry architecture and LD properties, this framework aims to improve the robustness and interpretability of causal inference in increasingly diverse genomic datasets.

As global biobank resources continue to expand in ancestry representation and scale, multi-ancestry fine-mapping will play a central role in equitable genomic discovery. Clarifying the structural assumptions underlying existing methods and identifying their consequences under realistic data conditions are essential steps toward accurate and transferable causal variant prioritization across populations.

## Methods

To systematically benchmark current multi-ancestry fine-mapping strategies, we selected nine representative methods and compared their modeling assumptions with respect to LD structure, variant support, and genetic effect sharing across ancestry groups. Based on these assumptions, we classified the methods into two primary categories. First, joint modeling approaches simultaneously model association signals across ancestries and allow information sharing through shared or correlated genetic effects and causal variant configurations; these include Paintor, SuSiEx, MultiSuSiE, XMAP, MESuSiE, and CARMA-X. Second, univariate-based approaches use single-ancestry or meta-analyzed evidence and subsequently integrate results across populations; these include association-level integration approaches, represented by SuSiE-weighted and SuSiE-merged, and a *post-hoc* integration approach, represented by SuSiE *post-hoc*. A unified description of these method classes, their assumptions, and their relationship to the multi-ancestry data-generating model is provided in Supplementary Note S1. Detailed software implementation, parameter settings, and output harmonization procedures are provided in Supplementary Note S2.

### Simulation scenarios

Our simulations were designed to systematically evaluate the performance of fine-mapping methods under a range of realistic multi-ancestry settings. We first defined a reference simulation scenario in which causal variants were restricted to a common subset of variants present in all ancestries, while allowing their causal status to differ across populations, representing the presence of ancestry-specific contextual variants (AS-CVs). Within this reference setting, we varied key aspects of the underlying genetic architecture known to influence fine-mapping resolution, including the number of causal variants, per-ancestry heritability, and sample-size imbalance across ancestries. Building on this reference scenario, we considered two core extensions. In the first extension, we introduced ancestry-specific variants to account for population-specific variant availability commonly observed in multi-ancestry GWAS. In the second extension, we introduced mismatch between GWAS summary statistics and LD reference panels to assess robustness to LD heterogeneity. Separately, as a complementary sensitivity analysis, we evaluated annotation-informed fine-mapping across varying levels of functional annotation enrichment and prior specification. Together, the core scenarios and complementary sensitivity analysis enabled us to evaluate the distinct effects of genetic architecture, variant availability, LD misspecification, and functional annotations on multi-ancestry fine-mapping.

### General simulation framework

We first randomly sampled 100 genomic regions from 266 candidate loci showing genome-wide significant associations in the Psychiatric Genomics Consortium GWAS of six neuropsychiatric disorders [24, 26, 33–36]. Candidate regions were identified by selecting SNPs that reached genome-wide significance ($P<5\times 10^{-8}$). For each significant SNP, we defined a 1-Mb region centered on it (±500 kb up and downstream) and merged the overlapping genomic regions. The length of the sampled genomic regions varied from 0.51 to 2.56 Mb (mean = 1.09 Mb; median = 1.24 Mb), with 575–5648 SNPs per region (mean = 2038 SNPs; median = 2190 SNPs).

For each region, we obtained genotype data from 50 000 randomly selected White British individuals and 6374 Black British individuals from UK Biobank to construct ancestry-specific LD reference matrices. Genotypes were standardized within each ancestry before LD estimation. We then simulated GWAS summary statistics for White British and Black British populations under controlled causal architectures, with a default target GWAS sample size of 300 000 individuals per ancestry. Marginal effect estimates and Z-scores were generated using a simulation framework that accounts for ancestry-specific LD, simulated causal effects, and sampling noise, with full technical details provided in Supplementary Note S3.

### Genetic architecture-driven simulation scenario

Under the simulation framework described above, we constructed genetic architecture-driven scenarios by varying the number of causal variants, their ancestry-sharing patterns, and per-variant effect sizes within each genomic region. Specifically, within each locus, we first randomly selected ${n}_0\in \left\{2,6,10\right\}$ causal variants. For each causal variant, we randomly assigned it to be effective across ancestries with probability *f=0.5*; otherwise, it was effective only in one ancestry as an AS-CV. We then simulated effect sizes for these causal variants in each ancestry, setting per-SNP heritability to ${h}^2\in \left\{{10}^{-4},2\times{10}^{-4}\right\}$. Across the 100 simulated genomic regions, this corresponds to a cumulative SNP-based heritability of ~ 0.02–0.20, reflecting the total contribution expected from roughly 100 genome-wide significant loci. This magnitude aligns with the proportion of trait heritability typically captured by top associated loci in large psychiatric GWAS, such as major depressive disorder (${h}_{\mathrm{SNP}}^2=10.3\%$) [37] and anxiety disorders (${h}_{\mathrm{SNP}}^2=4.4\%$) [38] when considered at similar locus counts, indicating that our simulated regions represent a realistic concentration of heritable signals observed in real data. To further evaluate robustness, we varied the GWAS sample-size ratio between the White British and Black British cohorts from 1:1 to 6:1, as well as a setting where all causal SNPs are cross-ancestry.

### Ancestry-specific variant availability scenario

We further considered ancestry-specific SNP scenarios to account for population-specific variant availability in multi-ancestry GWAS summary statistics, which may arise from differences in allele frequency, imputation quality, sequencing coverage, or quality-control filtering. We examined three settings: ancestry-specific noncausal variants, ancestry-specific causal variants mimicking AS-SVs, and ancestry-specific variant availability combined with incomplete LD reference-panel coverage.

Using the same 100 genomic regions and simulated effect sizes as in the genetic architecture-driven scenario, we first randomly masked 30% of noncausal variants in the GWAS summary statistics of either one or both ancestries, while retaining all causal variants. We selected 30% here to match the masking proportion used in the MESuSiE supplementary simulations [12] and to represent a moderate degree of ancestry-specific variant availability consistent with that observed in the All of Us analysis. We then considered settings involving ancestry-specific causal variants by designating one causal variant per region with regional heritability of either ${10}^{-4}$ or $2\times{10}^{-4}$, and restricting its availability to only a subset of ancestries. To maintain comparability, the overall proportion of ancestry-specific variants was fixed at 30% by additionally masking randomly selected noncausal variants. Finally, to mimic incomplete external LD reference coverage, we further removed 30% of variants from the LD reference panel, yielding ~ 51% cumulative variant unavailability in the affected ancestry or ancestries. Detailed masking procedures and variant-availability settings are provided in Supplementary Note S3.1.

### LD mismatch scenario

We further considered LD mismatch scenarios to evaluate the robustness of fine-mapping methods to discrepancies between the LD structure underlying GWAS summary statistics and that estimated from reference panels. First, we replaced in-sample LD with external LD reference panels from the 1000 Genomes Project Phase 3 [39], including 503 European and 661 African samples. This setting mimics a common summary-statistic fine-mapping application in which LD is estimated from a finite external reference panel rather than the original GWAS cohort. Fine-mapping performance was evaluated across the same 100 genomic regions and simulated effect sizes used in the genetic architecture-driven scenario, with three LD-control regimes: no control, Slalom [29] filtering, and SuSiE-RSS LD outlier filtering [28].

We next evaluated systematic LD mismatch by generating artificial SNP-level discrepancies in the GWAS summary statistics following Zhang et al. [40]. This scenario was designed to mimic structural sources of LD inconsistency, such as unmodeled population substructure or allele alignment errors, which frequently occur in large-scale GWAS meta-analyses [29, 41, 42]. Using the same 100 UK Biobank-derived regions, we simulated ${n}_0=6$ causal variants per region, each with a 50% probability of being ancestry-specific, and set per-SNP heritability to ${h}^2=2\times{10}^{-4}$. We randomly selected a proportion of variants, $p\in \left\{\mathrm{0.001,0.01,0.1}\right\}$, and multiplied their Z-scores by a mismatch factor $\rho \in \left\{0.5,0,-0.5,-1.0\right\}$ within each ancestry. Fine-mapping was then performed using the mismatched summary statistics and in-sample LD matrices, with and without LD outlier detection. Detailed procedures for generating mismatch and applying LD control are provided in Supplementary Note S3.2.

### Functional annotation-informed simulation scenario

We evaluated the effects of functional annotations and prior specification using Paintor, the only method in our benchmark that directly incorporates SNP-level annotations through enrichment priors. The simulation otherwise followed the genetic architecture-driven scenario, with causal variants sampled from an annotation-informed softmax prior using enrichment weights $w\in \left\{0,1,2\right\}$, representing no, moderate, and strong enrichment, respectively. At each enrichment level, we compared three Paintor configurations: no annotations; a selected panel comprising H3K27ac, DNase hypersensitivity, H3K4me3, conservation, nonsynonymous status, and a scalar sequence-effect score derived from Evo2 [32]; and a high-dimensional panel comprising all 187 baselineLF2.2.UKB annotations [43] together with the Evo2 score. Because Paintor reports SNP-level PIPs but does not distinguish cross-ancestry from ancestry-specific causal effects, we evaluated it using an either-ancestry causal indicator, where a variant was considered causal if it had a nonzero effect in at least one ancestry. Complete simulation and Evo2 scoring procedures are provided in Supplementary Note S3.4.

### Performance evaluation

For each scenario, we first evaluated fine-mapping performance using CS metrics. We compared the resolution of each method by examining the size of the 95% CSs, which are defined as the smallest set of variants 95% likely to contain a causal SNP effective in at least one ancestry. In this context, smaller sets indicate higher resolution. Simultaneously, we assessed the power achieved by these CSs, defined as the proportion of true causal variants successfully captured within the 95% CSs. For the genetic architecture–driven simulation scenario, we also assessed empirical CS coverage separately for each method. Each reported CS was treated as an individual unit and considered covered if it contained at least one known simulated causal variant. Empirical coverage was calculated as the proportion of reported CSs that met this criterion (see Supplementary Note S3.8 for details). We further assessed fine-mapping performance using high-PIP variants, as CS size is influenced by LD and may include many correlated but weakly supported SNPs. Specifically, we compared the power to identify three distinct types of causal signals: (i) either (variants causal in at least one ancestry), (ii) cross-ancestry, and (iii) ancestry-specific (AS-CVs). The classification of SNPs for each of these three signal types was based on applying a threshold *t* to its corresponding PIP. In the analysis, we varied the threshold *t* in the range of 0.01–0.99 to plot the precision-recall curve. In addition, we obtained the threshold *t* that corresponds to an FDR threshold of either 1%, 5%, or 10% following Wang et al. [44] and reported power of each method based on these thresholds. We additionally assessed the calibration of PIPs across methods by comparing inferred PIPs to the true causal proportions, following [12].

To quantify sampling uncertainty in the genetic architecture-driven simulations, we performed region-level bootstrap resampling with 1000 replicates. For each method and simulation setting, regions were sampled with replacement, and CS power, FDR, median CS size, and PIP-based power at nominal FDR targets of 0.05 and 0.10 were recomputed. Percentile-based 95% confidence intervals (CIs) were defined by the 2.5th and 97.5th percentiles of the corresponding bootstrap distributions. Additional methodological details on the bootstrap analyses are provided in Supplementary Note S3.7.

In settings where a causal variant was ancestry-specific and present in a single ancestry, mimicking the presence of AS-SV, we evaluated method performance based on both physical proximity and LD tagging of the detected signal relative to the true causal variant. Specifically, we identified the top detected signal for each method as the variant with the highest PIP. We then calculated the genomic distance between this top detected variant and the true causal variant. Second, to assess whether the detected signal was a valid proxy, we quantified the LD correlation (*r*) between the top detected variant and the true causal variant. To account for LD heterogeneity, we computed correlations in both ancestries and reported the maximum absolute correlation observed in either population (${r}_{\mathrm{max}}=\max \left(\left|{r}_{\mathrm{anc}1}\right|,\left|{r}_{\mathrm{anc}2}\right|\right)$). This approach assesses whether a method successfully prioritizes a variant that is physically close to and in strong LD with the true causal signal. Similar performance metrics were used when the true causal variant was unobserved (masked) across both ancestries.

### Computation time and memory usage

We evaluated computational efficiency by recording wall-clock runtime and peak memory usage across simulated genomic regions. These analyses were performed under the genetic architecture-driven scenario and summarized across repeated fine-mapping runs. For the functional annotation-informed simulations, we additionally recorded Paintor runtime and peak memory across the three annotation configurations (Supplementary Note S3.4). Runtime and memory usage were used to compare the practical scalability of methods across region sizes and model classes. Detailed profiling procedures, software-specific monitoring tools, exclusion rules for external meta-analysis runtime, and high-performance computing hardware specifications are provided in Supplementary Note S3.9.

### Real-data analysis in the All of Us Research Program

We applied multi-ancestry fine-mapping to body mass index (BMI), diastolic blood pressure (DBP), and systolic blood pressure (SBP) using GWAS summary statistics from the All of Us Research Program Data Release 8. These traits were selected because they are well-studied cardiometabolic phenotypes with sufficient sample sizes in both European and African ancestry groups. The analysis included short-read whole-genome sequencing data from 223 997 individuals of European ancestry and 72 538 individuals of African ancestry. Genetic ancestry labels were provided by All of Us and inferred using a random forest classifier trained on Human Genome Diversity Project [45] and 1000 Genomes Project reference samples. GWAS was performed separately within each ancestry group using REGENIE [46], adjusting for age, sex at birth, age-related interaction terms, ethnicity, and the top 16 genetic principal components. Ancestry-matched LD matrices were constructed using 1000 Genomes Project Phase 3 reference panels, including 503 European and 661 African samples. SNP-level quality control excluded variants with MAF < 1%, genotype call rate < 95%, Hardy–Weinberg equilibrium $P<1\times{10}^{-25}$, strand ambiguity, multi-allelic status, detected inconsistencies between GWAS summary statistics and LD matrices, or location within the major histocompatibility complex region (chr6: 25–34 Mb; detailed GWAS and QC procedures are provided in Supplementary Note S4).

Candidate fine-mapping regions were defined from marginal GWAS signals. Specifically, we selected SNPs reaching genome-wide significance $(P<5\times{10}^{-8})$ in at least one ancestry, constructed 1-Mb windows centered on each significant SNP (±500 kb), and merged overlapping intervals. This procedure yielded 289 fine-mapping regions, including 204 for BMI, 39 for DBP, and 46 for SBP. Regions ranged from 0.95 to 4.83 Mb in length, with a mean of 1.19 Mb and median of 1.01 Mb, and contained 1908–20 469 SNPs per region, with a mean of 3719 and median of 4170 SNPs. Fine-mapping was performed one region at a time using ancestry-specific marginal Z-scores and LD matrices as input. Because true causal variants are unknown in real data, method performance was assessed using the number of detected 95% CSs, CS size, median Z-scores of variants within CSs, and functional enrichment of prioritized variants. To identify high-confidence variants for enrichment analysis, we additionally considered PIP thresholds ranging from 0.5 to 0.9 [11, 12, 22, 42, 43, 47, 48].

We evaluated the functional relevance of real data fine-mapping results by testing enrichment of prioritized variants across coding and regulatory annotations, including non-synonymous variants, synonymous variants, 5′/3′ UTRs, promoters, cis-regulatory elements, and independent GTEx v8 eQTLs [49, 50]. Functional annotations were used only for this *post-hoc* evaluation and were not provided as inputs to the fine-mapping methods. Trait-relevant eQTL tissues included heart and artery for DBP and SBP, and 13 brain tissues for BMI. Enrichment was evaluated at multiple prioritization levels, including variants in 95% CSs, the top 100 variants ranked by overall PIP, and the top 50 variants ranked by cross-ancestry or ancestry-specific PIPs. Methods were compared by their enrichment within each annotation category and by their average rank across annotations. Fold-enrichment estimates were accompanied by approximate 95% CIs obtained by scaling Wilson score intervals for the prioritized-variant annotation proportion by the corresponding background annotation rate. Enrichment was tested using Pearson’s chi-square test or Fisher’s exact test when expected cell counts were below five, followed by Bonferroni correction across methods within each annotation and prioritization threshold. Detailed annotation sources, enrichment tests, and ranking procedures are provided in Supplementary Note S4.

### Multi-ancestry fine-mapping decision (MFD)

Motivated by the complementary strengths and limitations observed across methods, we developed the MFD strategy as a practical framework for selecting between existing fine-mapping approaches according to locus-level genetic characteristics. MFD provides an empirical decision rule for choosing between a joint multi-ancestry approach, represented by MESuSiE, and *post-hoc* integration of single-ancestry fine-mapping results, represented by SuSiE *post-hoc* (Fig. S22).

The decision framework is based on whether a locus contains significant ancestry-specific variants and whether these variants are independent of, or correlated with, shared association signals. In loci without evidence of ancestry-specific variants, MFD uses MESuSiE to leverage cross-ancestry information sharing and improve power for shared or partially shared causal architectures. In loci where ancestry-specific variants appear independent of cross-ancestry signals, MFD uses SuSiE *post-hoc* to preserve ancestry-specific variant support and reduce the risk of excluding AS-SVs. For ancestry-specific variants that are in LD with cross-ancestry signals, MFD further evaluates the consistency of association evidence across ancestries before selecting the final strategy (full details are provided in Supplementary Note S2).

### Application of methods across benchmarking scenarios

We applied the evaluated methods across all simulation and real-data analyses, with adjustments to account for method compatibility in each setting. In the genetic architecture-driven scenario, all methods were applied using the full set of simulated summary statistics. In the ancestry-specific SNP scenario and real data analysis, input handling varied by method class: standard joint modeling approaches (Paintor, SuSiEx, MultiSuSiE, MESuSiE, XMAP, and CARMA-X) were restricted to variants present in all ancestries, whereas association-level integration, *post-hoc* integration approaches, and the MFD framework operated on the union of variants observed in at least one ancestry. Additionally, the LD matrix used in the association-level integration approach in the ancestry-specific SNP scenario was estimated from pooled genotype data across individuals. Finally, for the LD mismatch scenario with artificial LD outliers, we evaluated all methods except for the association-level integration approach. It was excluded because our artificial LD outliers’ simulation modified ancestry-specific GWAS summary statistics relative to fixed ancestry-specific LD reference panels, whereas meta-analysis operates on pooled summary statistics and pooled LD matrices, making this simulation design not directly applicable. For the real-data analysis in *All of Us*, we excluded MultiSuSiE due to the high similarity in model assumptions and performance redundancy with SuSiEx and XMAP observed in simulations. For the association-level integration approach in the real-data analysis, we assumed a single causal variant per locus to avoid requiring an LD matrix, following [26] (SuSiE-Meta).

## Results

### Overview of evaluation framework and methods

We evaluated nine multi-ancestry fine-mapping methods including joint modeling and univariate-based integration approaches (Fig. 1; Supplementary Note S1). Joint modeling approaches (Paintor, SuSiEx, MultiSuSiE, XMAP, MESuSiE, and CARMA-X) model cross-ancestry association signals within a unified probabilistic framework, whereas univariate-based approaches were further stratified into association-level integration (SuSiE-weighted, SuSiE-merged) versus *post-hoc* integration (SuSiE *post-hoc*) approaches. These strategies rely on distinct assumptions regarding effect sharing and LD compatibility, suggesting that performance may depend on alignment between model assumptions and locus-specific data characteristics. To formalize this alignment, we developed the MFD strategy that adaptively selects among modeling approaches based on evidence for ancestry-specific variants and LD structure (Methods).

We assessed method performance using two complementary data resources (Fig. 1). First, we performed simulations using imputed UK Biobank genotypes to test methods under controlled genetic architectures and LD patterns. Second, we analyzed 289 fine-mapping loci associated with body mass index (BMI), diastolic blood pressure (DBP), and systolic blood pressure (SBP) from the All of Us Research Program. Across settings, we evaluated fine-mapping resolution, detection power, PIP calibration, robustness to misspecification, computational efficiency, and biological validity based on functional enrichment (Figs 1 and 2; Methods). To summarize performance across metrics, we provide a comprehensive comparison table for all methods (Fig. 2; Table S1; Supplementary Note S6).

**Figure 2:**
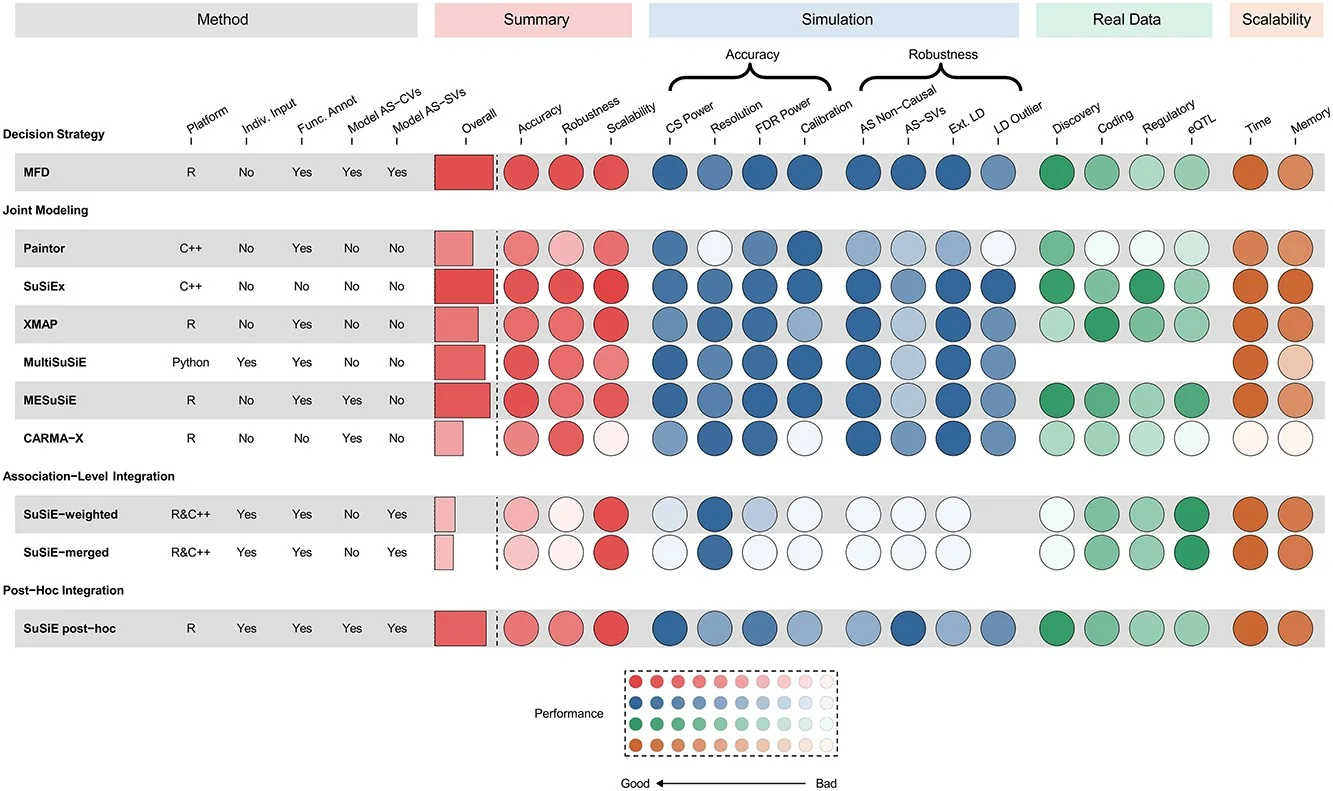
Summary table of the performance of all methods. We visualized the overall method performance using bars, where a longer bar represents a higher aggregate overall score, and detailed performance in terms of summary metrics, simulation accuracy and robustness, real data validation, and scalability using dots. We listed the decision strategy, programming platform, and modeling requirements (individual-level input, functional annotations, and ancestry-specific variant modeling) for each method. Functional annotation support includes direct annotation input or annotation-informed priors. For all dots, darker shading represents better performance.

### Simulation studies

We conducted extensive simulations to evaluate multi-ancestry fine-mapping under three complementary sources of cross-ancestry heterogeneity. First, we varied genetic architecture, with causal variants present across ancestries but allowing differences in contextual effects (mimicking ancestry-specific contextual variants, AS-CVs), with additional variation in the number of causal signals, per-ancestry heritability, and sample-size imbalance. Second, we modeled ancestry-specific variant availability, including scenarios with AS-SVs. Third, we introduced LD mismatch between GWAS summary statistics and reference panels to assess robustness to LD misspecification. Additionally, we evaluated the effects of annotation informativeness, enrichment strength, and prior dimensionality through a functional annotation-informed simulation.

### Genetic architecture-driven simulation scenario

Across methods, detection power and resolution improved with increasing per-SNP heritability and fewer causal variants per region, as reflected by smaller 95% CSs and higher power (Fig. 3A and B). Within joint modeling approaches, XMAP and CARMA-X produced the most compact CSs (median sizes of 20 and 19, respectively) but with lower detection power (0.58 and 0.54, respectively). SuSiE-based joint models (MESuSiE, SuSiEx, and MultiSuSiE) demonstrated balanced performance, with mean power of 0.63–0.66 and median CS sizes of 25–32. Paintor achieved comparable power (0.62) but produced larger CSs (median size 92). The *post-hoc* integration approach (SuSiE *post-hoc*) showed mean detection power comparable to joint models (0.67) but with larger CSs (median size 49). Association-level integration approaches (SuSiE-weighted, SuSiE-merged) showed reduced detection power (0.33–0.37) despite relatively compact CSs (median size 16–20). Empirical coverage of the reported CSs exceeded 0.90 for most joint modeling approaches and for SuSiE *post-hoc*, at 0.933 for MESuSiE (2107/2258 reported sets), 0.929 for SuSiEx (1981/2133), and 0.942 for SuSiE *post-hoc* (2682/2848), whereas SuSiE-weighted and SuSiE-merged showed substantially lower coverage of 0.616 and 0.288, respectively (Table S2b and S2c). Overall, joint modeling approaches tended to achieve higher resolution than *post-hoc* integration and higher detection power than association-level integration in this scenario.

**Figure 3:**
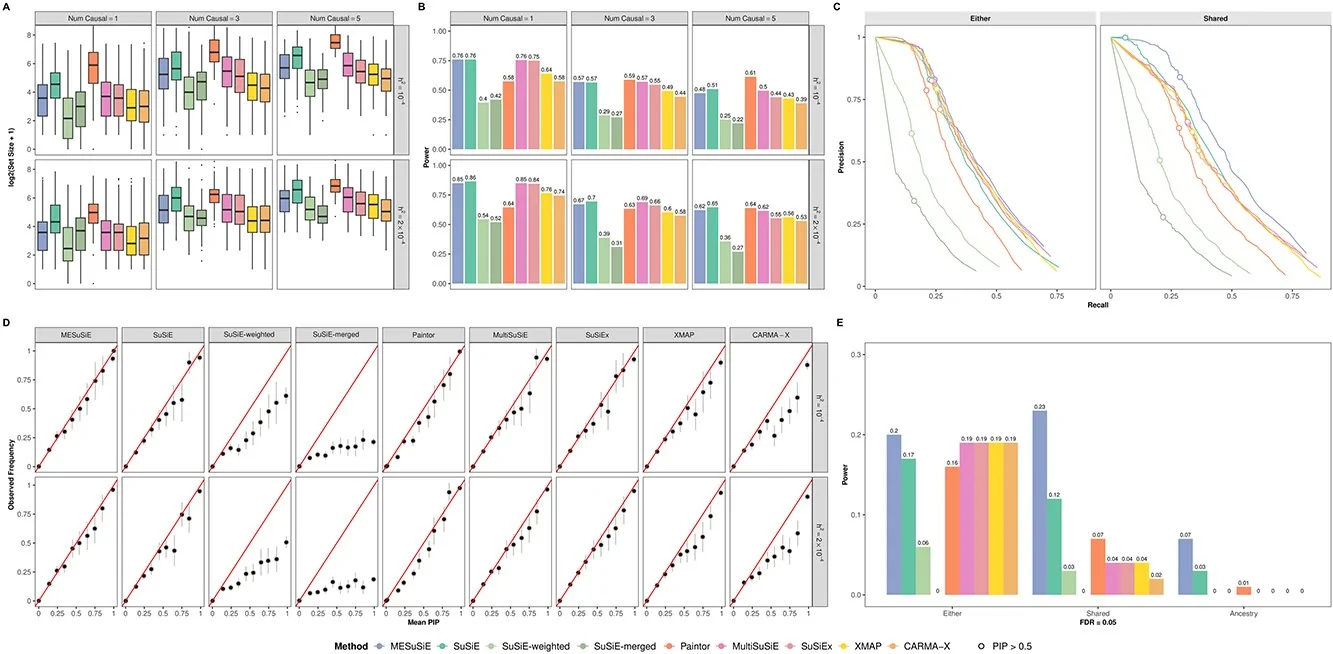
Evaluation of multi-ancestry fine-mapping method performance under the genetic architecture-driven simulation scenario. Method performance was evaluated across 100 simulated regions, stratified by the number of causal SNPs per ancestry $\left({n}_0=1,3,5\right.$; corresponding to 2, 6, and 10 total causal SNPs across ancestries) and per-causal SNP heritability $\big({h}^2={10}^{-4}$or $2\times{10}^{-4}\big)$. (A) Distribution of 95% credible set sizes, shown as ${\log}_2\left(\mathrm{set}\ \mathrm{size}+1\right)$. (B) Credible set power, defined as the proportion of causal variants captured by the 95% credible sets. (C) Precision-recall curves for detecting variants causal in at least one ancestry $\left({\mathrm{PIP}}_{\mathrm{either}}\right)$ and cross-ancestry causal variants $\left({\mathrm{PIP}}_{\mathrm{shared}}\right)$, with circles marking PIP = 0.5. Precision is defined as $1-\mathrm{FDR}$, and recall as $\mathrm{TP}/\left(\mathrm{TP}+\mathrm{FN}\right)$. (D) PIP calibration plots across 300 simulated regions, with variants ranked by ${\mathrm{PIP}}_{\mathrm{either}}$ and grouped into 10 bins. Points show observed causal proportions versus mean PIP; bars indicate *± 2* standard errors, and the diagonal indicates perfect calibration. (E) Power to detect overall, cross-ancestry, and ancestry-specific causal signals at 5% FDR.

Region-level bootstrap analyses produced relatively narrow CIs and preserved the principal performance rankings. For example, overall CS power was 0.658 for MESuSiE (95% CI, 0.640–0.676), 0.674 for SuSiE *post-hoc* (0.657–0.691), and 0.663 for MultiSuSiE (0.645–0.679). Full CIs for CS power, FDR, and median CS size are provided in Tables S22a and S22b.

To complement the CS evaluation, we further assessed fine-mapping performance using PIP-based precision–recall metrics. We first evaluated the ability to detect signals that are causal in at least one ancestry, using the corresponding PIP for all methods. We then separately examined the detection of cross-ancestry causal variants and AS-CVs, representing putative causal variants with effects common across ancestries or restricted to a single ancestry. For detection of variants causal in at least one ancestry, SuSiE-based joint methods (MESuSiE, SuSiEx, MultiSuSiE, and XMAP) and CARMA-X achieved the largest AUPRC values, followed by SuSiE (*post-hoc*), Paintor, and association-level integration approaches (Fig. 3C). At 5% FDR, MESuSiE detected 20% of causal signals on average, with SuSiEx, XMAP, MultiSuSiE, and CARMA-X achieving ~ 19%. SuSiE (*post-hoc*) detected 17%, while Paintor achieved 16%. Association-level approaches showed lower power at this threshold (0%–6%) (Fig. 3E; Fig. S1; Table S2). Complete locus-level bootstrap 95% CIs for power at nominal FDR targets of 0.05 are reported in Table S22c. PIP calibration was generally strongest among joint modeling approaches, with MESuSiE, MultiSuSiE, Paintor, and SuSiEx showing closer agreement between predicted PIPs and empirical causal frequencies. Association-level approaches and CARMA-X showed greater deviations (Fig. 3D).

When stratifying by signal type, we compared detection of cross-ancestry variants and AS-CVs using MESuSiE and SuSiE (*post-hoc*), the two methods explicitly modeling ancestry-specific effects. For cross-ancestry signals, MESuSiE detected 23% of causal variants at 5% FDR, compared to 12% for SuSiE (*post-hoc*) (Fig. 3E; Fig. S2). For ancestry-specific contextual signals, detection was lower across methods; MESuSiE achieved 10% power in the Black British group at 5% FDR, compared to 4% for SuSiE (*post-hoc*), with similar trends in the White British group (Fig. 3E; Figs S3–S5; Table S2a).

Additionally, we observed that detection power decreased under sample-size imbalance and increased when all causal SNPs were cross-ancestry (Figs S7–S12 and S19–S21; Tables S3 and S4). Relative method rankings remained largely unchanged across these settings and in extended simulations with 15 and 20 causal variants per region, although greater allelic heterogeneity reduced variant-level detection power and enlarged CSs (Supplementary Note S3.5; Figs S84–S89; Table S20). Notably, MFD selected MESuSiE across loci under this scenario due to the absence of ancestry-specific variants (Fig. S22).

### Ancestry-specific variant availability scenario

Most joint modeling approaches assume that causal variants are observed across ancestry groups. We evaluated method performance when this assumption is violated by simulating ancestry-specific variants through selective removal of causal and noncausal variants from the GWAS summary statistics.

When ancestry-specific variants were simulated as noncausal, restricting analyses to variants present in all ancestries reduced median CS sizes across joint modeling methods (e.g. MESuSiE: 30 to 22; SuSiEx: 25 to 20; MultiSuSiE: 32 to 24), with a similar reduction observed for the MFD strategy (30 to 26; Fig. 4A). Detection power for MFD and joint methods remained stable (0.64–0.67), whereas association-level integration (SuSiE-merged) declined substantially (0.33 to 0.13; Fig. 4B). Consistent with these trends, evaluation using PIP-based detection at 5% FDR indicated a modest power gain for joint models and MFD (~0.19–0.22), while SuSiE-merged remained at zero. Stratification by signal type further showed that MESuSiE detected the largest proportion of cross-ancestry signals (26%, up from 23%) and ancestry-specific contextual variants (e.g. 11% in the Black British group) compared to MFD and SuSiE (*post-hoc*; Fig. 4C; Tables S5 and S6). Similar patterns were observed when ancestry-specific variants were noncausal in both ancestries, with the performance of MFD closely aligned with MESuSiE due to the absence of AS-SVs (Fig. 4; Figs S23–S26).

**Figure 4:**
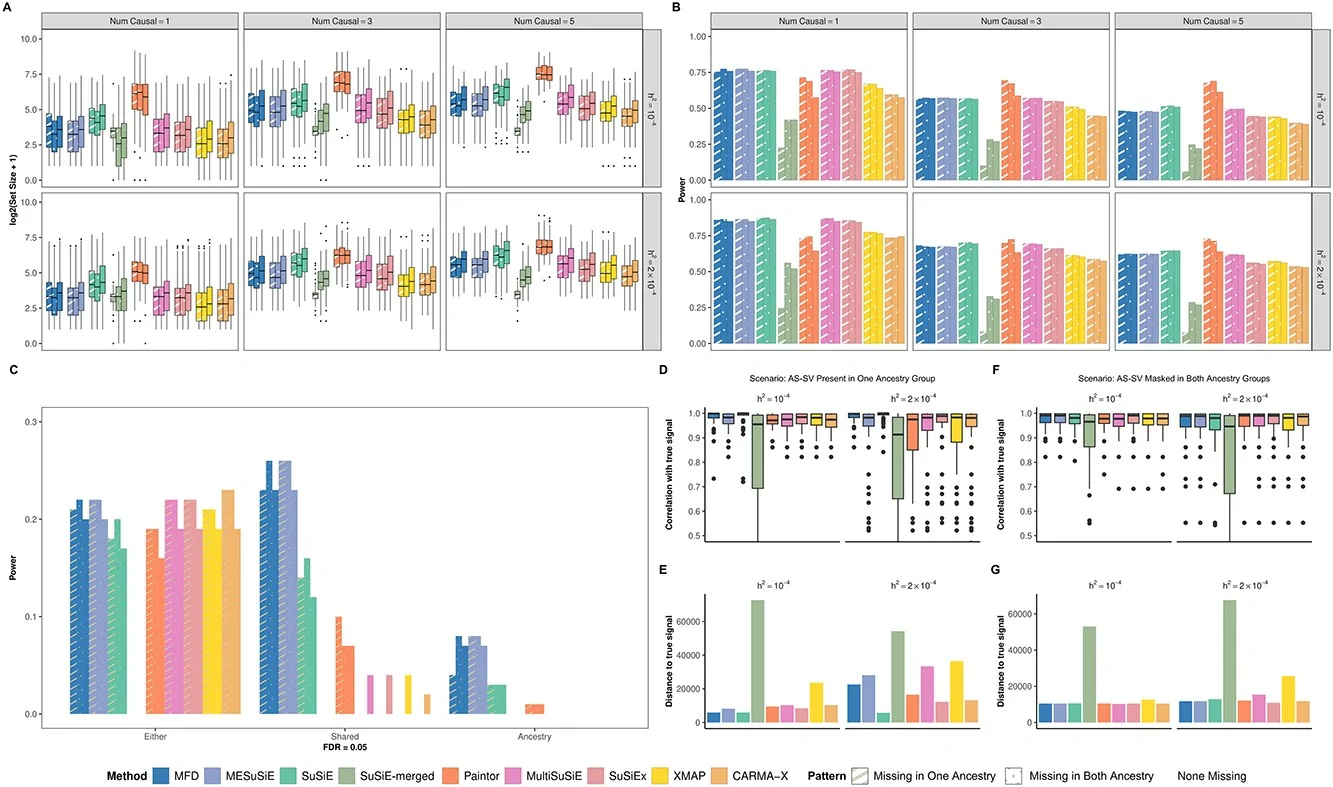
Evaluation of multi-ancestry fine-mapping method performance under the ancestry specific SNP scenario. (A–C) Performance in the presence of ancestry-specific noncausal SNPs missing in one ancestry (striped fills), missing in both ancestries (dotted fills), or without missing variants (solid fills). Results are summarized across 100 simulated regions, stratified by the number of causal SNPs per ancestry $\left({n}_0=1,3,5\right.$; corresponding to 2, 6, and 10 total causal SNPs across ancestries) and per-causal SNP heritability $\big({h}^2={10}^{-4}$ or $2\times{10}^{-4}\big)$. (A) Distribution of 95% credible set sizes, shown as ${\log}_2\left(\mathrm{set}\ \mathrm{size}+1\right)$. (B) Credible set power, defined as the proportion of causal variants captured by the 95% credible sets. (C) Power to detect overall $\left(\mathrm{Either}\right)$, cross-ancestry, and ancestry-specific causal signals at 5% FDR. (D–G) Performance when causal variants are unobserved in one ancestry or in both ancestries. (D, F) Correlation between the top prioritized variant and the true causal variant. (E, G) Mean genomic distance between the top prioritized variant and the true causal variant.

We next considered settings in which a causal variant was ancestry-specific to a single ancestry, mimicking the presence of AS-SV, and performance was evaluated using both physical distance and LD correlation between the detected signal and the hidden causal variant. Under this setting, the *post-hoc* integration approach showed the strongest alignment with the true signal (Fig. 4D and E; Tables S7 and S8). Specifically, SuSiE (*post-hoc*) achieved the highest mean correlations approaching 0.99 at higher heritability (${h}^2=2\times{10}^{-4}$) with mean physical distance ~ 5.7 kb from the causal site. Joint modeling approaches showed varying degrees of proxy divergence; for example, MESuSiE and MultiSuSiE exhibited increased mean distances (~28–33 kb) in the higher heritability setting. The MFD framework offered partial recovery of these signals; at high heritability, it improved upon MESuSiE (correlation 0.97 versus 0.90; distance 22.6 kb versus 28.1 kb), though it did not reach the performance of SuSiE (*post-hoc*). However, at lower heritability (${h}^2=1\times{10}^{-4}$), MFD performance effectively converged with SuSiE (*post-hoc*, mean |𝑟| = 0.98, distance *∼ 5.9* kb) and outperformed MESuSiE (mean $\left|r\right|=0.97$, distance *∼* 8.2 kb).

Finally, we examined the identification of causal variants when the true variant was unobserved (masked) in both ancestries. Under this setting, CARMA-X and SuSiEx showed high correlation with the unobserved signal (|𝑟| ≈ 0.94–0.97), with MESuSiE and MFD also exhibiting correlations exceeding 0.92 (Fig. 4F and G). Association-level integration showed lower correlations (~0.84) and larger physical distances (>50 kb; Fig. 4F and G; Tables S7 and S8). Overall, similar patterns were observed when variants were absent from both GWAS summary statistics and LD reference panels (Figs S27–S33).

### LD mismatch scenario

We further evaluated the robustness of fine-mapping methods to uncertainty in LD specification, assessing performance both with and without LD outlier detection. When using external LD reference panels, we observed a moderate reduction in power across all methods, though relative performance rankings remained consistent with those obtained using in-sample LD (Figs S13–S18). For example, MESuSiE, MultiSuSiE, and SuSiE (*post-hoc*) showed power decreases of 0.04–0.05. Paintor showed a notable increase in CS size (from 92 to 176 variants). Furthermore, in this specific setting, the application of LD outlier detection methods (Slalom or SuSiE-RSS) resulted in minor differences in performance metrics compared to analyzing the data without LD control (Figs S34–S38; Table S9).

We next evaluated method performance under SNP-level LD mismatch, mimicking allele flipping and other sources of systematic discrepancies between GWAS summary statistics and reference LD [29, 41, 42]. In the absence of correction, increasing the severity and proportion of mismatched SNPs led to a consistent decline in 95% CS power and PIP-based AUPRC across all methods (Fig. 5; Figs S39–S48; Table S10). Joint modeling approaches (SuSiEx, XMAP, MESuSiE, and MultiSuSiE) tended to produce overly confident, shrinking CSs under mismatch, whereas Paintor exhibited substantial expansion in set size, indicating a loss of resolution. Furthermore, the effectiveness of LD correction was highly dependent on the mismatch context. While Slalom improved performance under mild discrepancies, it offered limited recovery in severe settings. Conversely, SuSiE-RSS successfully recovered detection power and maintained better precision-recall performance under extreme allele-flipping scenarios, but showed limited improvement in other mismatch settings (Table S10; Figs S39 and S40).

**Figure 5:**
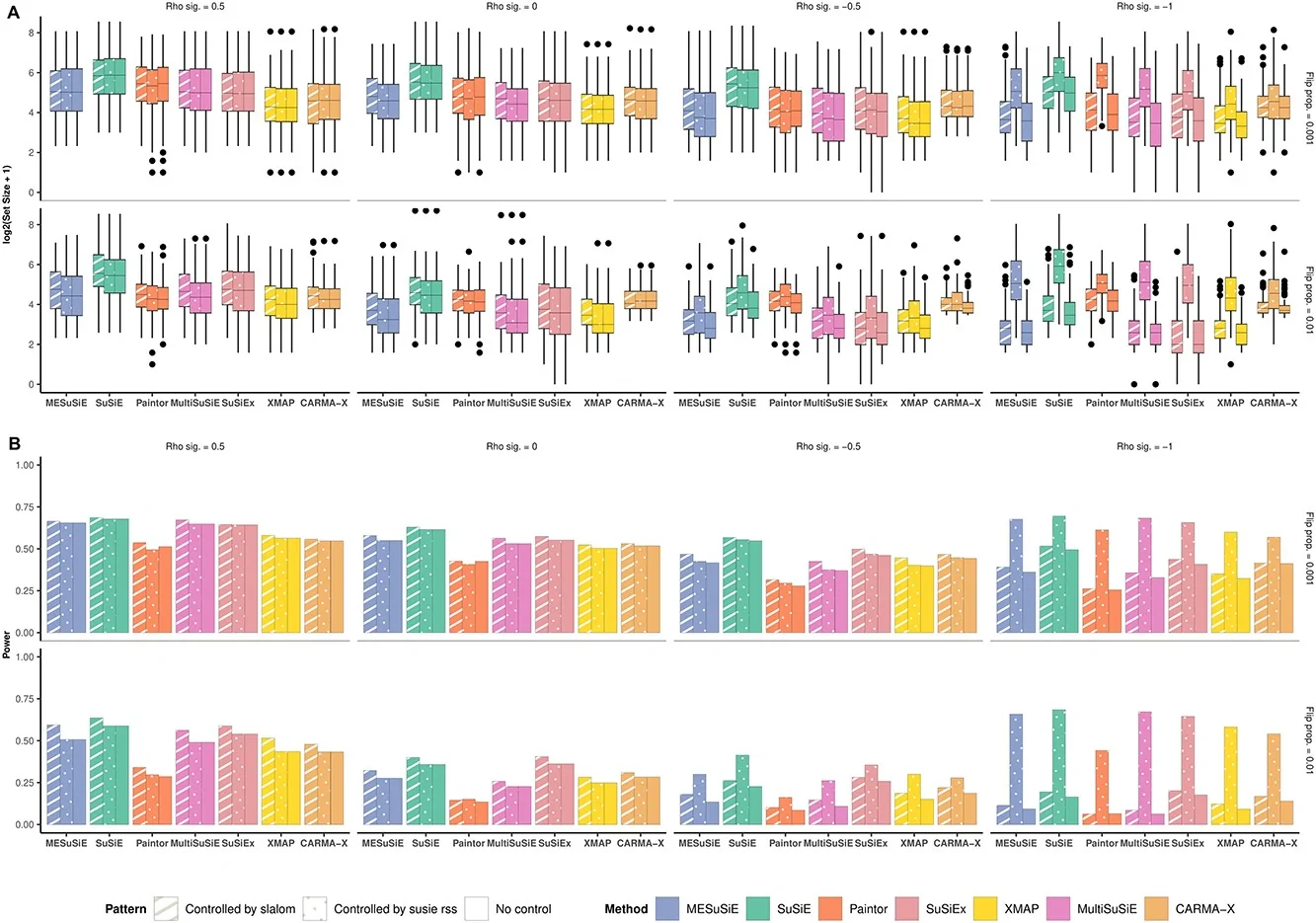
Performance metrics based on credible sets with introduced LD mismatch. Comparison of 95% credible set sizes and power in detecting causal signals with non-zero effects in at least one ancestry. Results are summarized across *n=100* independent regions. To denote LD handling strategies, plots are filled with diagonal stripes for methods controlled by Slalom, dots for methods controlled by SuSiE-RSS, and solid colors for methods with no additional control. (A) Boxplots displaying the ${\log}_2\left(\mathrm{set}\ \mathrm{size}+1\right)$ of the 95% credible sets. (B) Bar plots displaying the power of the constructed 95% credible sets, defined as the proportion of simulated causal variants successfully included in the credible set. Panels are stratified by the proportion of variants with induced mismatch (rows: 0.001, 0.01) and the scale of the mismatch factor (columns: $\rho \in \left\{0.5,0,-0.5,-1\right\}$).

### Functional annotation-informed simulation scenario

We next evaluated how functional annotations and prior specification affected multi-ancestry fine-mapping performance using Paintor, the only method in our benchmark that directly incorporates SNP-level annotations through enrichment priors. Under strong annotation enrichment, Paintor with the selected annotation panel outperformed Paintor without annotations across CS- and PIP-based metrics. The selected panel reduced the median CS size from 80 to 34 variants and increased mean CS power from 0.612 to 0.691. It also increased FDR-controlled power from 0.167 to 0.267, exceeding the values observed for MESuSiE, SuSiEx, and XMAP, which ranged from 0.195 to 0.205. Under moderate enrichment, the benefit was smaller: the median CS size decreased from 85 to 59.5 variants, but mean CS power decreased slightly from 0.616 to 0.585, while FDR-controlled power increased from 0.170 to 0.190 (Figs S70, S71, S76, and S77; Table S19).

The high-dimensional annotation panel performed poorly under both enrichment strengths despite containing the same informative annotations. It produced small CSs, with a median size of nine variants, but mean CS power decreased to 0.436 under strong enrichment and 0.402 under moderate enrichment, and FDR-controlled power at 5% FDR was zero. The combination of small CSs and low CS power indicated overly concentrated PIPs rather than improved resolution. These results show that annotation-informed priors can improve fine-mapping when annotations are informative and the prior is well specified, whereas high-dimensional panels containing annotations of uncertain relevance can reduce causal-variant recovery and calibration (Figs S64–S83; Table S19).

### Computational efficiency

Computational costs scaled with SNP count across 100 candidate regions (Fig. S6; Table S11). MultiSuSiE, SuSiEx, SuSiE (*post-hoc*), and SuSiE-weighted were fastest (median 0.015–0.028 min), followed by an intermediate tier comprising SuSiE-merged, XMAP, and MESuSiE (0.08–0.15 min). Paintor and CARMA-X were the most computationally demanding methods; Paintor was consistently slower (median 3.11 min), while CARMA-X was substantially more expensive (median 15.8 min, 95th percentile: 51.1 min). Within the functional annotation-informed simulations, Paintor with the high-dimensional annotation panel had a median runtime of ~ 5 min, with a small number of regions requiring 599–758 min, indicating computational instability (Table S11a). Peak memory was lowest for SuSiEx (median 38.8 MB), with most methods clustering between 200 and 330 MB. Conversely, MultiSuSiE (737 MB) and CARMA-X (1141 MB) required the highest memory allocations and exhibited steeper scaling with region size (Fig. S6).

### Real-data analysis in All of Us

We applied multi-ancestry fine-mapping to 289 loci for BMI, DBP, and SBP using All of Us GWAS summary statistics (V8) with LD constructed from reference panels (1000 Genomes Phase 3; 503 European and 661 African samples; Figs S49–S50; Table S12).

We first compared methods by the number of CSs they identified across loci (Fig. 6A). Across traits, MFD and SuSiE-based joint models identified the largest number of CSs (Fig. 6A). For BMI (204 loci), MFD, MESuSiE, SuSiEx, and SuSiE (*post-hoc*) identified 187–190 CSs, compared with 143, 81, 64, and 16 from Paintor, CARMA-X, XMAP, and SuSiE-Meta, respectively. Similar patterns were observed for DBP and SBP. Importantly, high recovery among SuSiE-based methods did not come at the cost of resolution (Fig. 6B): median CS sizes for MFD and SuSiE-based joint models ranged from 11 to 16 variants. In contrast, SuSiE-Meta, XMAP, and CARMA-X produced smaller CSs (median size 1–4) but with substantially reduced detection, whereas Paintor yielded larger sets (median size 537). We further quantified signal strength using the median Z-scores of variants within CSs (Fig. 6C). Most methods, including MFD, MESuSiE, CARMA-X, SuSiEx, and SuSiE (*post-hoc*), showed similar values (~5.5–5.9). In contrast, XMAP and SuSiE-Meta yielded higher median Z-scores (6.25 and 6.42), whereas Paintor CSs contained substantially weaker signals (~1.9).

**Figure 6:**
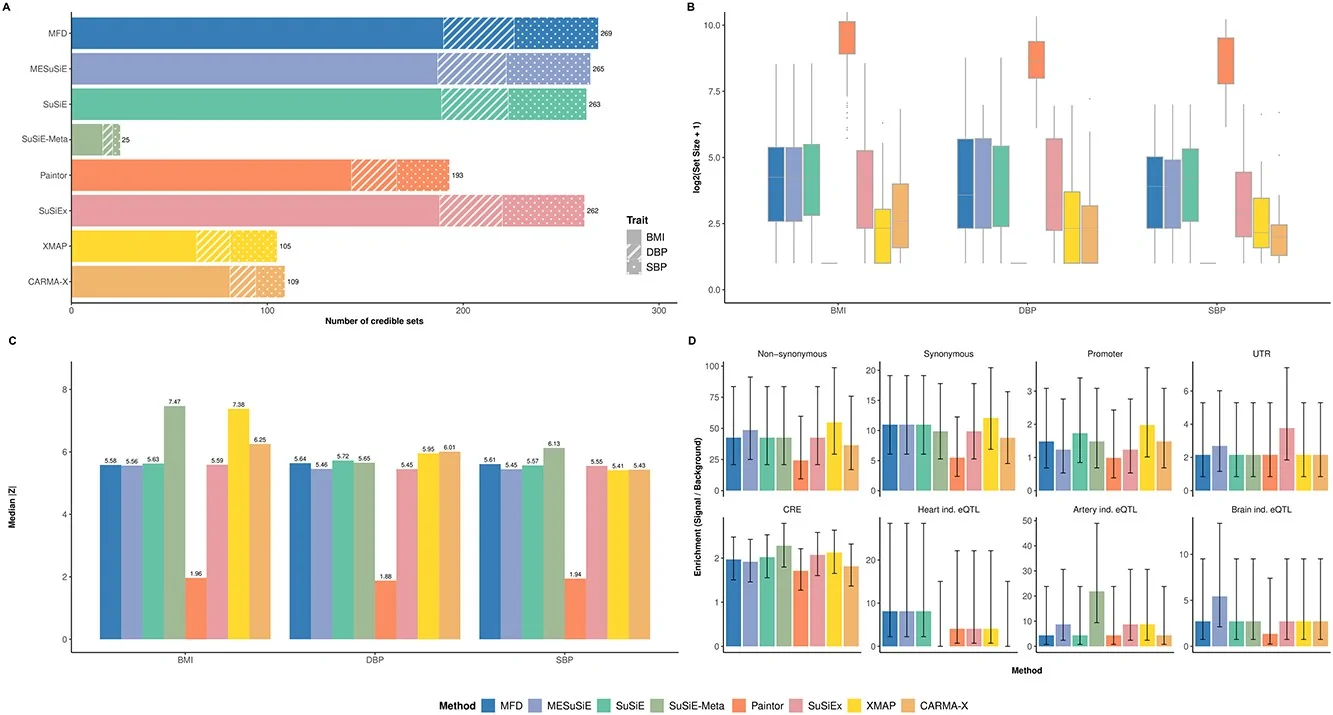
Multi-ancestry fine-mapping results summary on three *All of Us* quantitative traits. This figure summarizes multi-ancestry fine-mapping results for body mass index (BMI), diastolic blood pressure (DBP), and systolic blood pressure (SBP). (A) Stacked horizontal bars show the number of credible sets discovered for each method across traits (solid fills for BMI, stripes for DBP, and dots for SBP). (B) Boxplots display the distribution of log2 (set size +1) for the 95% credible sets across the 289 genomic regions analyzed, stratified by trait and method. (C) Bar plots show the marginal association strength of SNPs within the 95% credible set, represented by the median absolute GWAS Z-score across ancestries. (D) Fold enrichment across eight functional annotation categories: non-synonymous, synonymous, promoter, UTR, cis-regulatory elements (CREs), independent heart eQTL, independent artery eQTL, and independent brain eQTL. Enrichment is calculated as the ratio of the proportion of potential causal SNPs (defined as the top 100 variants based on $PI{P}_{\mathrm{either}}$ of each method) carrying a specific annotation to the proportion of that annotation in the background set. Error bars indicate approximate 95% confidence intervals based on Wilson score intervals.

We next examined functional enrichment among high-PIP variants prioritized in at least one ancestry. Enrichment was evaluated across five annotation categories, including non-synonymous variants, synonymous variants, promoter regions, untranslated regions (UTRs), and cis-regulatory elements, following previous studies [42, 48]. Overall, XMAP achieved the strongest functional enrichment (average rank 1.6), driven heavily by coding annotations, including 54.8-fold non-synonymous enrichment (95% CI, 29.3–98.8; *P* = 1.19 × 10^−12^) and 12.1-fold synonymous enrichment (95% CI, 6.86–20.4; *P* = 1.94 × 10^−8^; Table S13a). SuSiE *post-hoc*, SuSiE-Meta, MFD, MESuSiE, and SuSiEx clustered closely (ranks 2.8–3.6), while CARMA-X (5.4) and Paintor (7.0) ranked lower. Noncoding regulatory enrichments were generally modest (<4-fold) and method-specific (Fig. 6D; Figs S51–S52; Tables S13 and S14). MESuSiE and MFD preferentially assigned high PIP to variants annotated as eQTLs in trait-relevant tissues (Fig. S53; Table S15a). For BMI, MESuSiE attained the strongest brain eQTL enrichment (7.0-fold; 95% CI, 3.02–15.6; *P* = .0064). For blood pressure traits, artery eQTLs showed substantial enrichment (e.g. 8.6-fold for DBP across four methods and up to 24.5-fold for SBP via MFD, MESuSiE, SuSiE *post-hoc*, CARMA-X, and SuSiE-Meta), while MESuSiE and SuSiEx uniquely identified the strongest SBP enrichment in heart tissue (10.9-fold; 95% CI, 3.71–30.6; *P* = .0225).

For cross-ancestry and ancestry-specific putative causal variant prioritization, MESuSiE identified 70 putative cross-ancestry causal variants, compared with 52 for MFD and 2 for SuSiE (*post-hoc*). SuSiE (*post-hoc*) identified 129 ancestry-specific variants (including 17 AS-SVs), compared with 73 (11 AS-SVs) for MFD and 46 (0 AS-SVs) for MESuSiE. Functional enrichment of the top 50 variants within each signal class showed that, for cross-ancestry signals, MESuSiE and MFD achieved higher overall enrichment across functional annotations. MFD showed the strongest artery eQTL enrichment (17.5-fold; 95% CI, 4.83–58.9; *P* = .018), whereas MESuSiE showed the strongest brain eQTL enrichment (10.9-fold; 95% CI, 4.29–25.6; *P* = .0015). For ancestry-specific signals, SuSiE (*post-hoc*) showed the strongest enrichment in non-synonymous variants (60.7-fold; 95% CI, 26.4–129.7; *P* = 7.3 × 10^−8^), whereas MFD maintained substantial enrichment in non-synonymous variants (36.4-fold; 95% CI, 12.5–98.4; *P* = 2.5 × 10^−4^) and uniquely captured heart eQTL enrichment (16.3-fold; 95% CI, 4.49–54.8; *P* = .021; Figs S54–S55; Table S16).

Finally, we assessed whether these patterns hold beyond two ancestries by adding East Asian summary statistics from the Taiwan Precision Medicine Initiative [4, 51], which extended the analysis to 334 loci (Table S12). Performance patterns were broadly consistent, although the stricter cross-ancestry variant overlap shifted MFD toward the SuSiE *post-hoc* endpoint (MESuSiE at 152 loci, SuSiE *post-hoc* at 182; Figs S105–S110; Tables S13b–S17b; Supplementary Note S5).

### Representative loci for cross-ancestry and ancestry-specific signals

We present two representative loci to compare the performance of multi-ancestry fine-mapping approaches. The first example, rs4932179 at the *FES* locus (chromosome 15), highlights a potential cross-ancestry causal variant for DBP. This variant exhibited genome-wide significance ($P\approx 6.31\times{10}^{-9}$; Fig. 7) in the African cohort and suggestive significance ($P\approx 6.10\times{10}^{-6}$) in Europeans. rs4932179 lies within an intronic enhancer and is an eQTL for *FES*, which encodes a kinase involved in vascular resistance [52, 53], consistent with a shared regulatory mechanism underlying DBP across ancestries. MFD and joint modeling methods (MESuSiE, SuSiEx, and XMAP) prioritized this variant with high posterior support as a cross-ancestry causal signal with high confidence ($\mathrm{PI}{\mathrm{P}}_{\mathrm{shared}}$  *≈* 0.64–0.70; Fig. S56). In contrast, the *post-hoc* integration approach classified it as an African-specific signal (PIP_EUR_ = 0.004, PIP_AFR_ = 0.74). This discrepancy is likely driven by complex, population-specific LD patterns: Europeans possessed 11 SNPs in high LD with rs4932179, whereas these variants are only weakly correlated in the African population. Furthermore, SuSiE-Meta failed to prioritize rs4932179 (PIP *≈* 0.11), instead prioritizing the variant with the strongest marginal meta-analysis GWAS signal. These findings suggest that joint modeling approaches leverage diverse LD structures across ancestries to achieve higher fine-mapping resolution and more accurate prioritization of putative causal variants.

**Figure 7:**
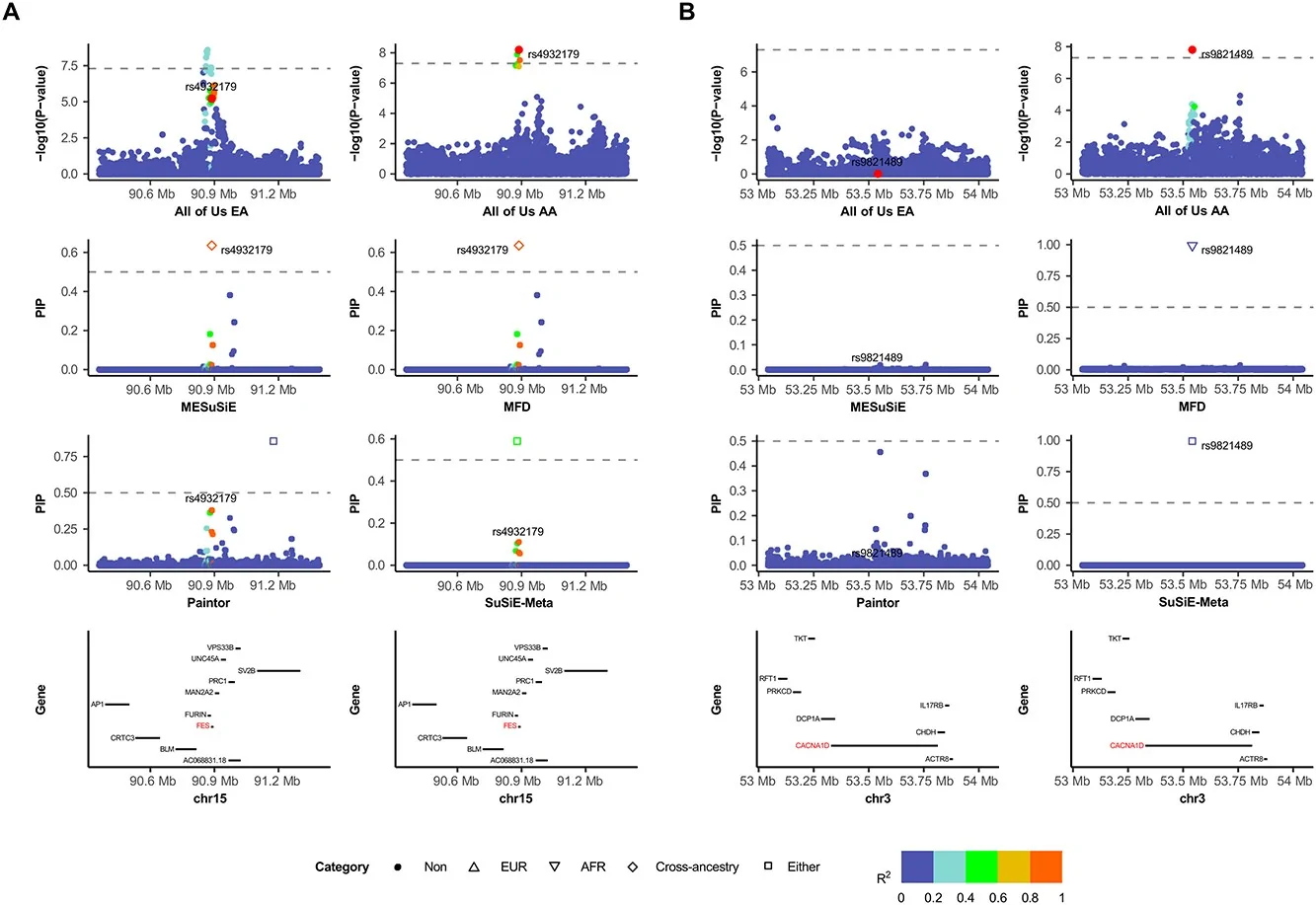
LocusZoom fine-mapping of diastolic blood pressure (DBP) in the *All of Us* European American (EA) and African American (AA) cohorts. (A, B) The first row shows LocusZoom plots displaying the negative log_10_(*P* value) from the marginal GWAS plotted against chromosomal position for EA and AA. The horizontal dashed line marks the genome-wide significance threshold ($P=5\times{10}^{-8}$). Color of a SNP represents the LD (${r}^2$) with the lead variant in the region. Second row shows LocusZoom plots displaying the PIP_either_ of MESuSiE (left) and MFD (right) across base pair positions. Third row shows LocusZoom plots for the PIP_either_ from Paintor (left) and SuSiE-Meta (right) across base pair positions. Signal types are determined based on the corresponding PIP values: Variants with PIP_shared_ > 0.5 are classified as cross-ancestry signals, those with PIP_EUR_ > 0.5 as European-specific and those with PIP_AFR_ > 0.5 as African-specific. The fourth row presents the gene annotation track for the locus, with the candidate gene highlighted. In the PIP panels, we denote European-specific signals by upward-pointing triangles, African-specific signals by downward-pointing triangles and cross-ancestry signals by diamonds. Squares indicate high-PIP variants from Paintor and SuSiE-Meta, without ancestry-specific classification, while dots indicate variants not meeting the corresponding PIP thresholds.

The second example illustrates rs9821489, a potential AS-SV for DBP in the African ancestry group, located within the *CACNA1D* gene on chromosome 3. This SNP was excluded from European GWAS due to a low minor allele frequency (MAF < 0.01) but demonstrated genome-wide significance in the African ancestry group ($P=1.58\times{10}^{-8}$, MAF = 0.49; Fig. 7). MFD and SuSiE (*post-hoc*) strongly prioritized rs9821489 (PIP = 0.994) and classified it as African-specific (Fig. 7; Fig. S57). SuSiE-Meta also detected the signal (PIP = 0.994), driven by the strong African association only without European evidence. In contrast, joint modeling approaches such as MESuSiE failed to recover the variant. Functional evidence further supports the biological plausibility of this prioritization: rs9821489 has been previously fine-mapped in non-European populations [15] and maps to an intronic region of *CACNA1D*, which encodes a voltage-gated calcium channel subunit implicated in blood pressure regulation and targeted by antihypertensive therapies [54]. This locus illustrates that methods that accommodate ancestry-specific effects may be better positioned to recover variants present in only a subset of populations, whereas joint modeling approaches optimized for cross-ancestry signals may have limited power in this setting.

## Discussion

We systematically evaluated nine representative multi-ancestry fine-mapping methods spanning both univariate-based and joint modeling approaches. Rather than restricting analysis to idealized simulation settings, we examined conditions that directly affect fine-mapping reliability in practice, including heterogeneity in genetic architecture across ancestries, differences in variant support, and mismatch between LD reference panels and GWAS summary statistics. These factors were assessed through structured simulations and further examined using GWAS summary statistics from the *All of Us* Research Program. Across these settings, we observed meaningful differences in method behavior, revealing trade-offs between univariate-based and joint modeling approaches in detection power, resolution, and sensitivity to model misspecification. We summarize these patterns in the MFD strategy, which provides a data-driven guideline for method selection under different analysis scenarios. Overall, our results suggest that performance differences across methods are closely tied to alignment between modeling assumptions and locus-specific ancestry architecture.

This dependence on locus-specific architecture was most evident in how methods handled causal-variant sharing, effect heterogeneity, and differences in variant availability across ancestries. For cross-ancestry causal variants, joint modeling approaches generally achieved higher power and better resolution by leveraging shared genetic architecture across populations. Performance diverged when causal effects or variant availability differed across ancestries. For AS-CVs, which are present across ancestries but have effects in only a subset, joint modeling methods that allow ancestry-specific effects generally outperformed *post-hoc* integration approaches by combining cross-ancestry information while accommodating effect heterogeneity. By contrast, for AS-SVs, most joint modeling methods exclude those causal variants because they analyze only the intersection of variants available across ancestries. *Post-hoc* integration approaches preserve the full ancestry-specific variant sets by fine-mapping each ancestry separately and were therefore better able to recover these variants directly. Their advantage for AS-SVs reflects broader input variant availability and robustness to differences in variant support, rather than an intrinsic statistical superiority of *post-hoc* modeling. An input-matched sensitivity analysis further supported this interpretation (Supplementary Note S3.1.1; Fig. S90; Tables S7 and S8). Joint modeling and *post-hoc* integration approaches are therefore complementary: joint methods provide a modeling advantage for cross-ancestry causal variants and AS-CVs, whereas *post-hoc* integration approaches offer an input-availability advantage for recovering AS-SVs.

Association-level integration approaches, which aggregate summary statistics and LD information across ancestries, generally showed reduced detection power, particularly when ancestry-specific variants were present. This reduction likely reflects the implicit assumption of cross-ancestry causal signals and the compression of ancestry-resolved LD information into a unified representation. Additionally, while some association-level integration studies restrict analyses to a single causal variant per locus to avoid explicit LD modeling [26, 55], our results suggest that this strategy may bias inference toward the strongest marginal association when multiple correlated signals are present. More broadly, within the genetic architecture-driven scenario, relative method rankings remained stable with up to 20 causal variants per region, although increasing allelic heterogeneity reduced variant-level detection power and enlarged CSs (Supplementary Note S3.5; Figs S84–S89; Table S20).

We translate these performance patterns into the MFD framework. Rather than introducing a new fine-mapping method, MFD provides a data-driven guideline for selecting among existing approaches based on locus-level summary characteristics. Applied to GWAS summary statistics from the All of Us cohort, MFD indicated that most loci for the traits examined were consistent with predominantly cross-ancestry signals, whereas a smaller subset showed evidence of ancestry-specific variant segregation (Table S17a). Sensitivity analyses further showed that the decision rule was portable across joint endpoints: replacing MESuSiE with SuSiEx or XMAP produced similar overall causal-variant recovery. MESuSiE nevertheless remained the preferred default because its ancestry-decomposed PIPs support classification of cross-ancestry and ancestry-specific effects (Supplementary Note S3.6; Figs S91–S104; Table S21).

The applicability of these findings and the MFD framework beyond pairwise ancestry analyses was further examined in a supplementary three-ancestry analysis of BMI, DBP, and SBP. The three-ancestry results showed patterns of locus-level signal detection and CS resolution broadly consistent with those of the two-ancestry analysis, which served as our primary real-data evaluation because its pairwise structure provides a more readily interpretable setting for characterizing method behavior (Figs S105–S110; Tables S13–S17). The three-ancestry analysis combined European- and African-ancestry GWAS summary statistics from the All of Us Research Program with East Asian-ancestry data from the Taiwan Precision Medicine Initiative (Supplementary Note S5; Table S12) [4, 51]. Adding the third ancestry imposed stricter requirements for shared-variant and LD-proxy support within MFD, shifting more loci toward the *post-hoc* endpoint. Although limited to a single three-ancestry configuration, this analysis provides initial support for extending MFD beyond pairwise settings while highlighting increased sensitivity to cross-dataset variant overlap.

Independent of locus-specific ancestry architecture and MFD endpoint selection, LD mismatch introduced a separate source of model misspecification. Sampling-induced LD uncertainty modestly reduced fine-mapping resolution (Table S9). In contrast, systematic discrepancies between GWAS summary statistics and external LD reference panels reduced detection power and distorted CS calibration (Table S10). Existing LD outlier detection procedures mitigated specific failure modes, such as allele flipping, but showed limited sensitivity to broader LD misalignment. Together, these results indicate that LD mismatch remains an incompletely addressed source of inference error in multi-ancestry fine-mapping.

Furthermore, most existing multi-ancestry fine-mapping methods are designed for summary statistics derived from separate ancestry-stratified cohorts, limiting their direct applicability to admixed populations. In admixed individuals, local ancestry varies across the genome, and local ancestry-specific association statistics estimated from the same individuals may therefore be correlated. In our local ancestry-aware simulation, CARMA-X achieved the strongest FDR-controlled performance, consistent with its explicit modeling of cross-ancestry covariance. Methods that assume independent ancestry-specific summary statistics, including the default joint endpoint used by MFD, were less well matched to this data-generating process (Supplementary Note S3.3; Figs S58–S63; Table S18). We therefore interpret this analysis as a stress test that motivates the development of frameworks jointly modeling local ancestry, cross-ancestry covariance, and ancestry-specific effects.

Taken together, our analyses highlight several limitations of current multi-ancestry fine-mapping approaches. First, many joint modeling methods assume shared causal structure and restrict analysis to variants observed across all ancestries, limiting their ability to detect AS-SVs. By design, these approaches exclude variants that are present only in certain populations due to allele frequency differences, imputation variability, or quality control filtering [13, 17]. While a recent method, PIPSORT [17], attempts to incorporate ancestry-specific variants within a joint framework, computational complexity remains a practical barrier. PIPSORT exhibited extended runtimes (>48 h) for moderate-sized regions (~1000 SNPs), with computational cost increasing substantially as the number of variants increased (Fig. S6). Second, reliance on external LD reference panels continues to constrain the robustness of fine-mapping using summary statistics. Existing LD discrepancy detection procedures were developed primarily for univariate settings and operate independently within each ancestry [28, 29], without explicitly modeling cross-ancestry inconsistencies. Third, scalable and well-calibrated integration of high-dimensional functional annotations remains limited. Despite the growing availability of rich epigenomic and regulatory annotations from large consortia and deep learning models [31, 32, 56–58], Paintor was the only benchmarked method that incorporated functional annotations directly, and its practical application favored compact, prespecified annotation sets. Finally, most available methods assume summary statistics from separate ancestry-stratified cohorts and are therefore not designed for correlated local ancestry-specific statistics estimated from admixed samples.

Several directions for future methodological development emerge from these findings. First, extending joint fine-mapping models to accommodate ancestry-specific variants and to scale to a larger number of ancestry groups will be essential for fully leveraging the growing diversity of global biobank resources. Second, improving scalability remains essential, as fully Bayesian joint models such as CARMA-X and PIPSORT incur substantial runtime and memory costs. Third, developing LD discrepancy diagnostics that explicitly account for cross-population inconsistencies may enhance robustness in multi-ancestry analyses. Finally, the continued development and collection of comprehensive whole-genome sequencing reference panels from diverse cohorts are essential. As resources such as *All of Us* continue to expand, the availability of accurately matched reference data will be pivotal in reducing the LD mismatch limitations that currently constrain fine-mapping performance.

**Key Points**

- Multi-ancestry fine-mapping performance depends on whether method assumptions match the locus-specific ancestry architecture.
- Joint modeling approaches generally improve power and resolution for cross-ancestry causal signals but can miss variants observed only in a subset of ancestries.
- *Post-hoc* integration preserves ancestry-specific variant support and LD patterns, making it more robust for ancestry-specific segregating variants.
- LD mismatch between GWAS summary statistics and reference panels remains a major source of miscalibration and incomplete causal variant recovery.
- A practical decision framework can guide method selection by combining evidence for ancestry-specific variants, LD structure, and signal consistency.

## Supplementary Material

Supplementary_material_bbag574

## Data Availability

Individual-level genotype data from UK Biobank are available upon application through the UK Biobank resource at: http://www.ukbiobank.ac.uk. Whole-genome sequencing and phenotype data from the *All of Us* Research Program are available through the Registered Tier and Controlled Tier which can be applied at: https://allofus.nih.gov/. GTEx v8 eQTL data are publicly available at: https://gtexportal.org/home/datasets. Baseline annotation data are available at: https://data.broadinstitute.org/alkesgroup/LDSCORE/baselineLF_v2.2.UKB.tar.gz. Phase 3 data from the 1000 Genomes Project are publicly available at: https://ftp.1000genomes.ebi.ac.uk/vol1/ftp/release/20130502/. GWAS summary statistics for BMI, DBP, and SBP from the All of Us Research Program generated in this study have been deposited in Figshare at: https://figshare.com/s/20a4e554de69e186ad59. East Asian GWAS summary statistics were obtained from the Taiwan Precision Medicine Initiative (TPMI). All TPMI summary statistics are freely available from the TPMI website (https://tpmi.ibms.sinica.edu.tw) and can be browsed and downloaded through TPMI PheWeb (https://pheweb.ibms.sinica.edu.tw). MFD is open-source software and is publicly available at: https://github.com/gchen4422/MFD. Analysis scripts used to reproduce the results are publicly available at: https://github.com/gchen4422/MFD_analysis, and scripts for conducting GWAS in *All of Us* are available in the *All of Us* researcher workbench.
