# Supplementary material for: Systematic benchmarking of multi-ancestry fine-mapping under variant heterogeneity and LD mismatch
Source: Brief Bioinform. 2026 Oct 19;27(5):bbag574. doi: 10.1093/bib/bbag574 (PMC13677793; doi:10.1093/bib/bbag574)
Supplement: Supplementary file 1 [file supplementary_material_bbag574.zip › Supplementary_Figures_Revision_submission_bbag574.pdf]

Supplementary Figures

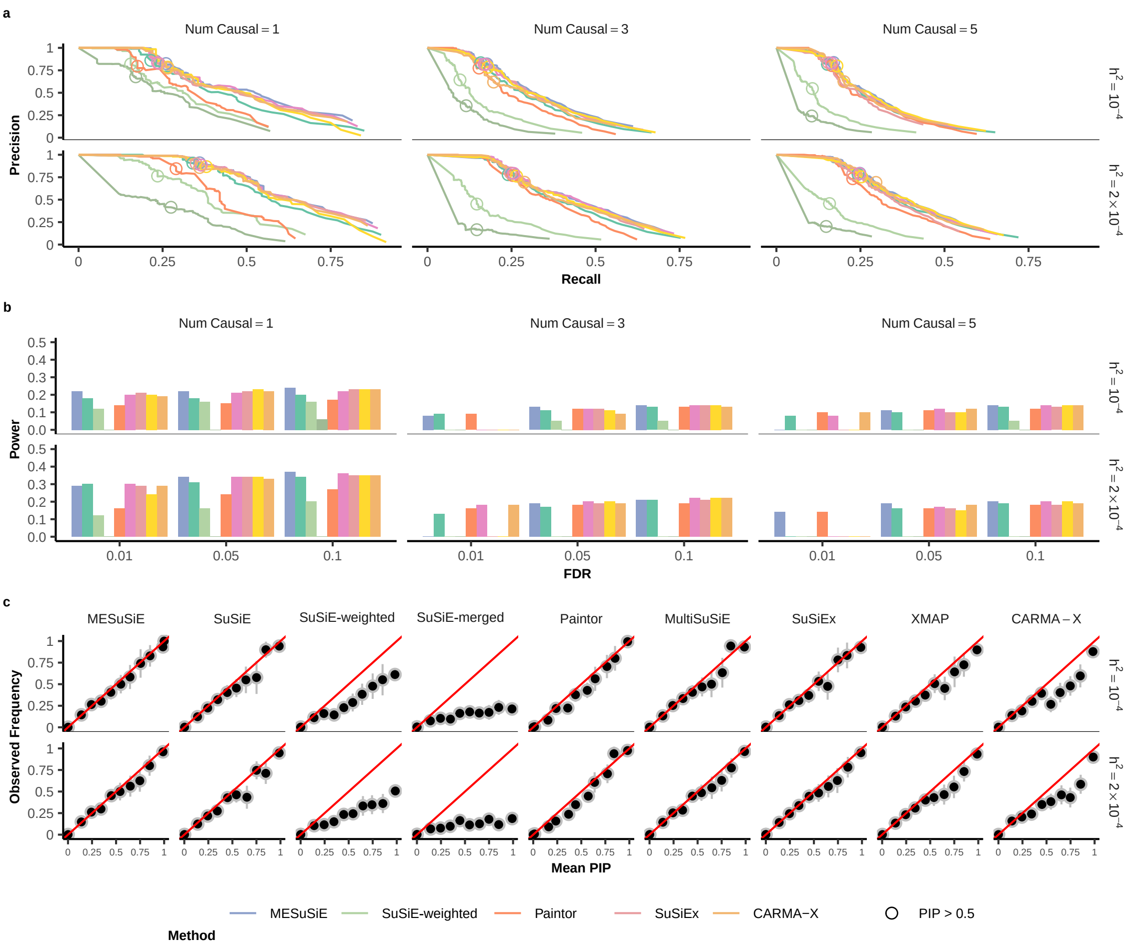

Supplementary Figure 1: **Comparison of method performance in detecting causal signals with non-zero effects in at least one ancestry (genetic architecture-driven simulation scenario, 'Either').** Results are stratified by the number of cross-ancestry causal SNPs (1, 3, or 5, corresponding to a total of 2, 6, and 10 causal SNPs, respectively) and per-causal SNP heritability ($h^2 = 10^{-4}$ or 2×10^{-4}; rows). **(a)** Precision-recall curves with PIP_{either} thresholds ranging from 0.01 to 1. Precision is defined as $1 - \text{FDR}$ and Recall as $\frac{\text{TP}}{\text{TP+FN}}$, where TP, FP, and FN represent true positives, false positives, and false negatives, respectively. The PIP threshold of 0.5 is marked with a circle. **(b)** Bar plots displaying power at controlled FDR levels of 0.01, 0.05, and 0.1. **(c)** Calibration plots aggregating $n = 300$ independent regions (combining all causal SNP scenarios). SNPs are ranked by PIP and divided into 10 equally sized bins. The y-axis shows the observed proportion of causal signals, and the x-axis shows the mean PIP within each bin. Gray error bars represent ± 2 standard errors. The red diagonal line indicates perfect calibration; points below the line suggest inflated PIPs (more false discoveries), while points above indicate conservative PIPs.

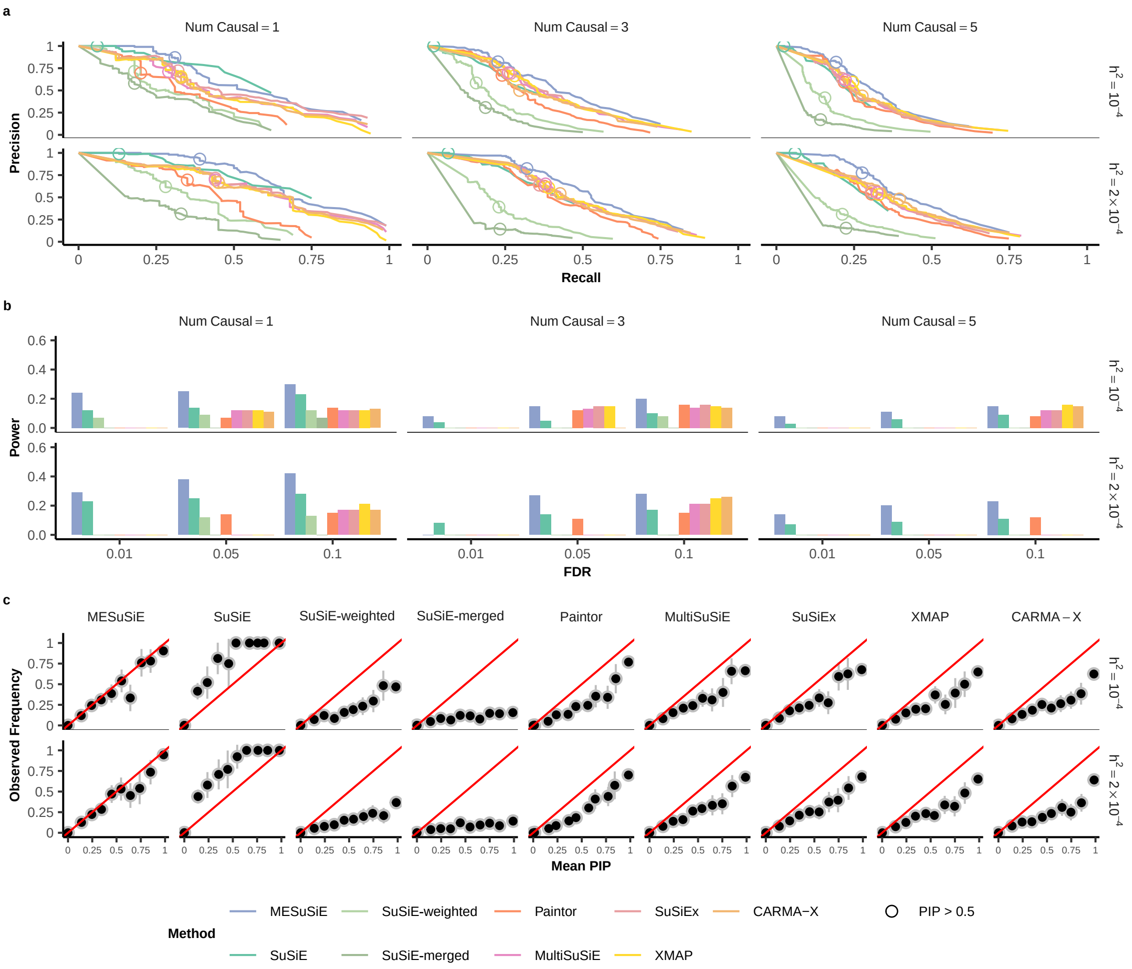

Supplementary Figure 2: **Comparison of method performance in detecting causal signals with non-zero effects in both ancestries (Genetic architecture driven simulation scenario, 'Cross-ancestry').** Results are stratified by the number of cross-ancestry causal SNPs (1, 3, or 5, corresponding to a total of 2, 6, and 10 causal SNPs, respectively) and per-causal SNP heritability ($h^2 = 10^{-4}$ or 2×10^{-4}; rows). **(a)** Precision-recall curves with PIP_{shared} thresholds ranging from 0.01 to 1. Precision is defined as $1 - \text{FDR}$ and Recall as $\frac{\text{TP}}{\text{TP+FN}}$, where TP, FP, and FN represent true positives, false positives, and false negatives, respectively. The PIP threshold of 0.5 is marked with a circle. **(b)** Bar plots displaying power at controlled FDR levels of 0.01, 0.05, and 0.1. **(c)** Calibration plots aggregating $n = 300$ independent regions (combining all causal SNP scenarios). SNPs are ranked by PIP and divided into 10 equally sized bins. The y-axis shows the observed proportion of causal signals, and the x-axis shows the mean PIP within each bin. Gray error bars represent ± 2 standard errors. The red diagonal line indicates perfect calibration; points below the line suggest inflated PIPs (more false discoveries), while points above indicate conservative PIPs.

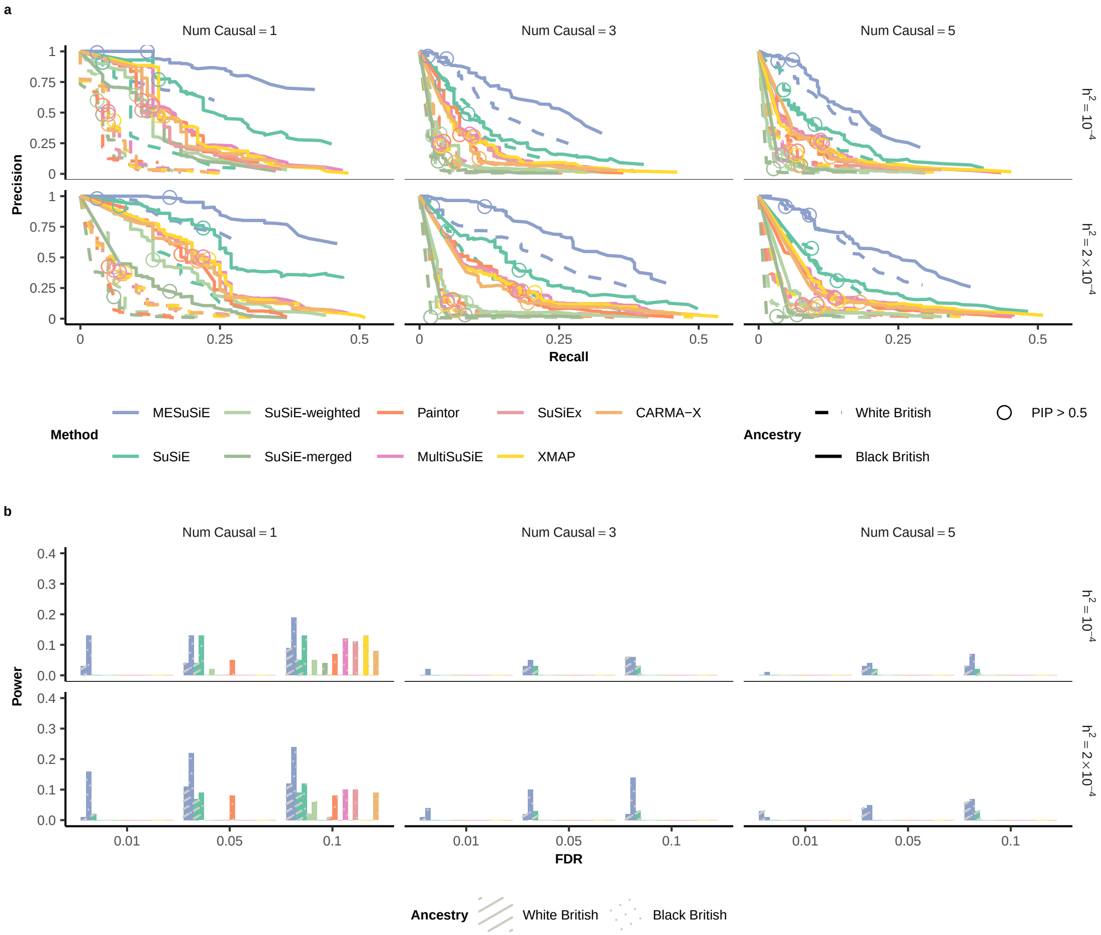

Supplementary Figure 3: **Performance metrics for ancestry-specific contextual causal signals (Genetic architecture driven simulation scenario).** Comparison of method performance in detecting causal signals with non-zero effects in White British only and Black British only. Results are stratified by the number of cross-ancestry causal SNPs (1, 3, or 5, corresponding to a total of 2, 6, and 10 causal SNPs, respectively) and per-causal SNP heritability ($h^2 = 10^{-4}$ or 2×10^{-4}; rows). **(a)** Precision-recall curves with PIP_{WB} and PIP_{BB} thresholds ranging from 0.01 to 1. Precision is defined as $1 - \text{FDR}$ and Recall as $\frac{\text{TP}}{\text{TP+FN}}$, where TP, FP, and FN represent true positives, false positives, and false negatives, respectively. The PIP threshold of 0.5 is marked with a circle. **(b)** Bar plots displaying power at controlled FDR levels of 0.01, 0.05, and 0.1.

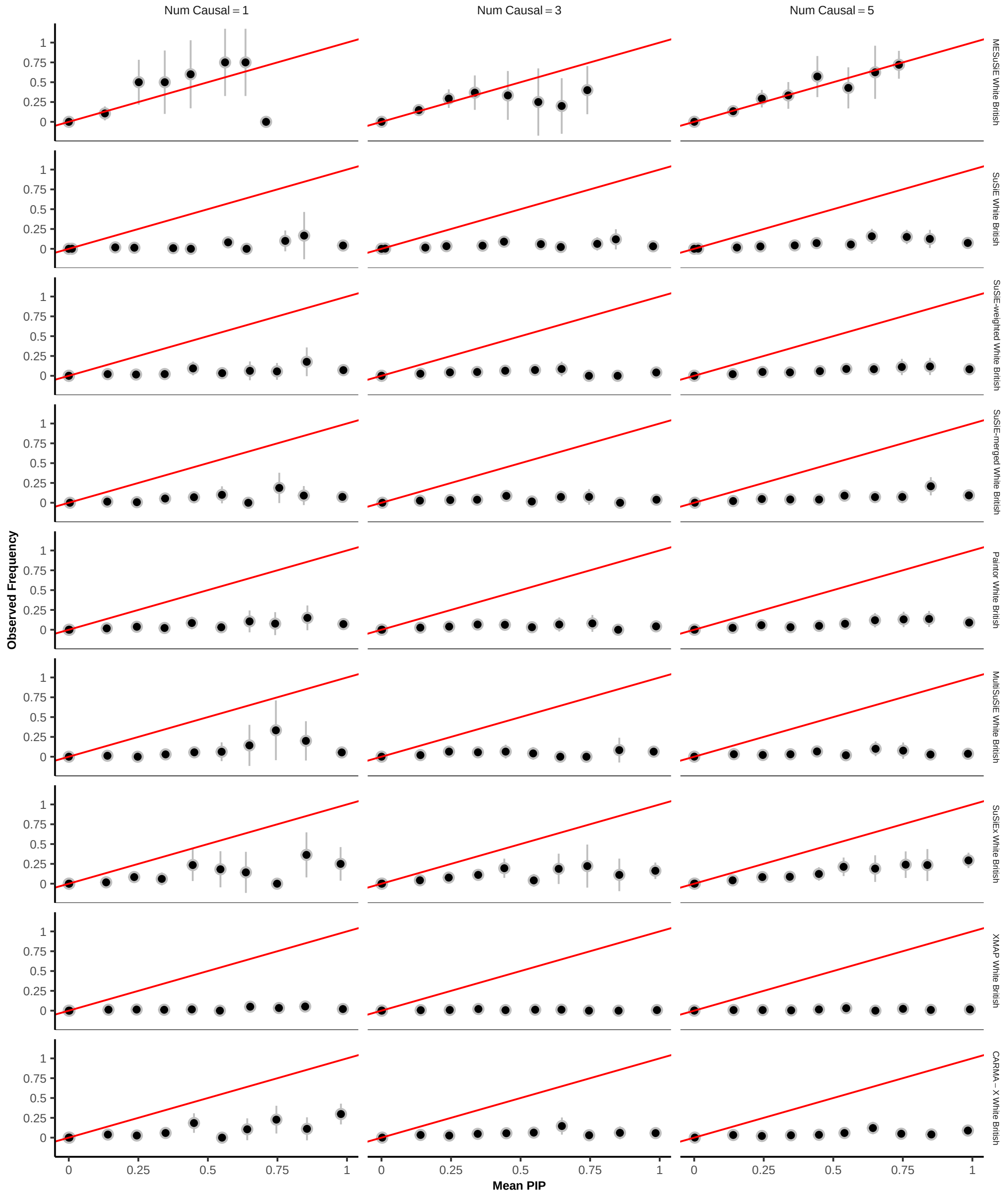

Supplementary Figure 4: **PIP Calibration for White British (WB) Ancestry (Genetic architecture driven simulation scenario).** Calibration plots aggregating $n = 300$ independent regions (combining all causal SNP scenarios). SNPs are ranked by PIP and divided into 10 equally sized bins. The y-axis shows the observed proportion of causal signals, and the x-axis shows the mean PIP within each bin. Gray error bars represent ± 2 standard errors. The red diagonal line indicates perfect calibration; points below the line suggest inflated PIPs (more false discoveries), while points above indicate conservative PIPs.

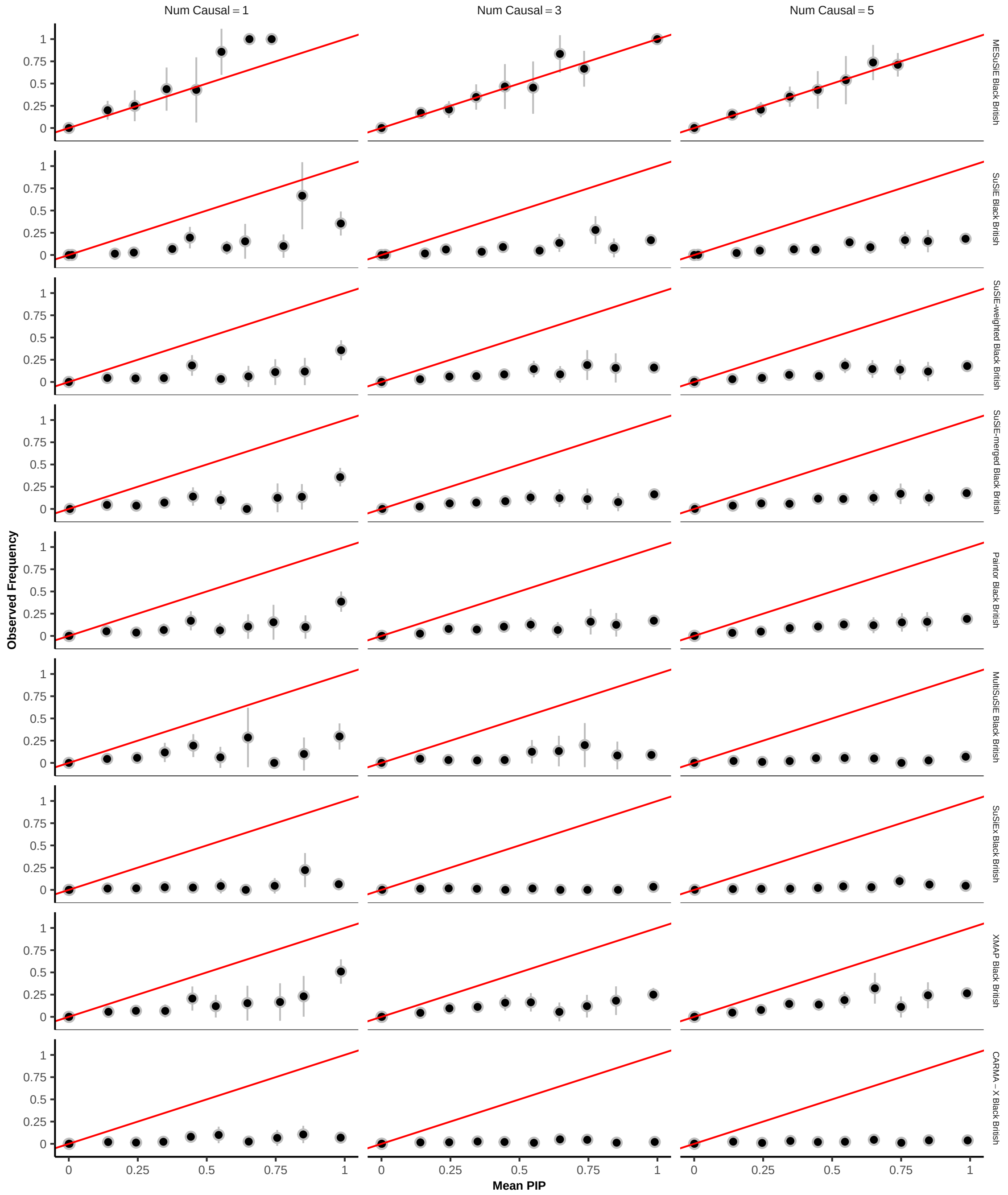

Supplementary Figure 5: **PIP Calibration for Black British (BB) Ancestry (Genetic architecture driven simulation scenario).** Calibration plots aggregating $n = 300$ independent regions (combining all causal SNP scenarios). SNPs are ranked by PIP and divided into 10 equally sized bins. The y-axis shows the observed proportion of causal signals, and the x-axis shows the mean PIP within each bin. Gray error bars represent ± 2 standard errors. The red diagonal line indicates perfect calibration; points below the line suggest inflated PIPs (more false discoveries), while points above indicate conservative PIPs.

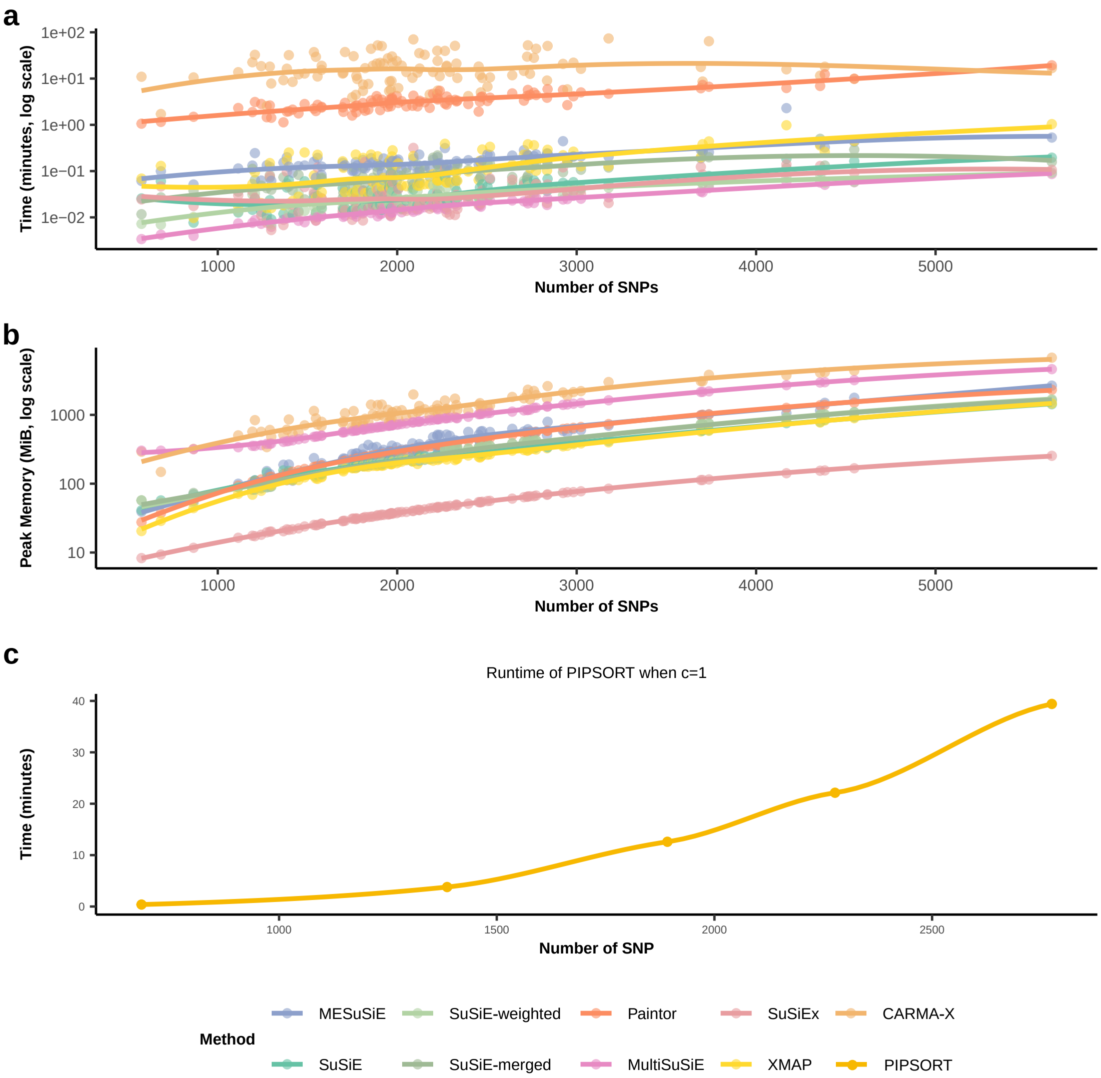

Supplementary Figure 6: **Computational cost and memory evaluation across fine-mapping methods.** Comparison of runtime and memory usage as a function of the number of variants (SNPs) in the genomic region. **(a)** Average runtime (in minutes, $\log_{10}$ scale) for evaluated methods. Points represent the mean execution time across replicates for each region size, with LOESS smoothing lines. **(b)** Average peak memory usage (in MiB, $\log_{10}$ scale). **(c)** Runtime specifically for PIPSORT in the single causal variant setting ($c = 1$). Note that runtime of PIPSORT is plotted on a linear scale.

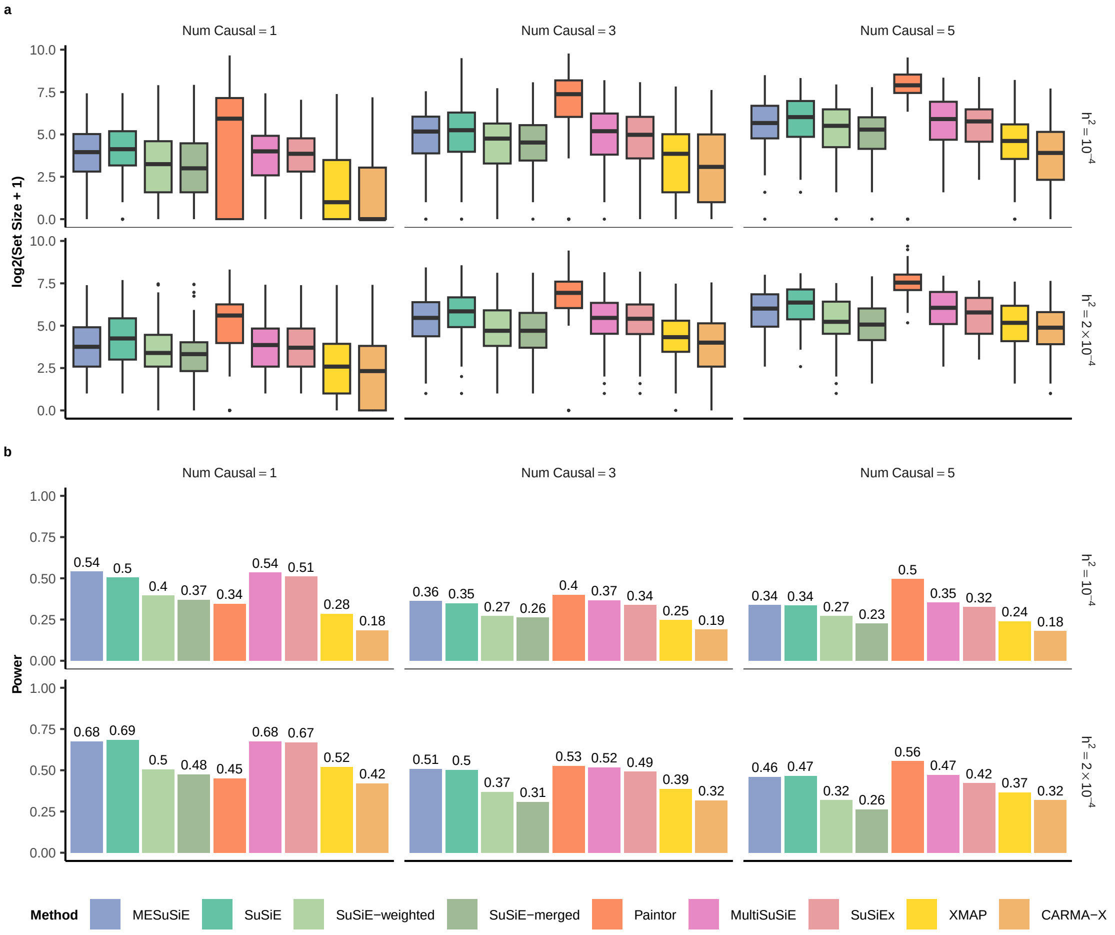

Supplementary Figure 7: **Performance metrics based on credible sets (Genetic architecture driven simulation scenario, 50,000 Black British samples).** Comparison of 95% credible set sizes and power in detecting causal signals with non-zero effects in at least one ancestry under the simulation setting with 50,000 Black British. Results are summarized across $n = 100$ independent regions, stratified by the number of causal SNPs (1, 3, or 5, corresponding to a total of 2, 6, and 10 causal SNPs, respectively) and per-causal SNP heritability ($h^2 = 10^{-4}$ or 2×10^{-4}; rows). **(a)** Boxplots displaying the $\log_2$(set size + 1) of the 95% credible sets. **(b)** Bar plots displaying the power of the constructed 95% credible sets, defined as the proportion of simulated causal variants successfully included in the credible set.

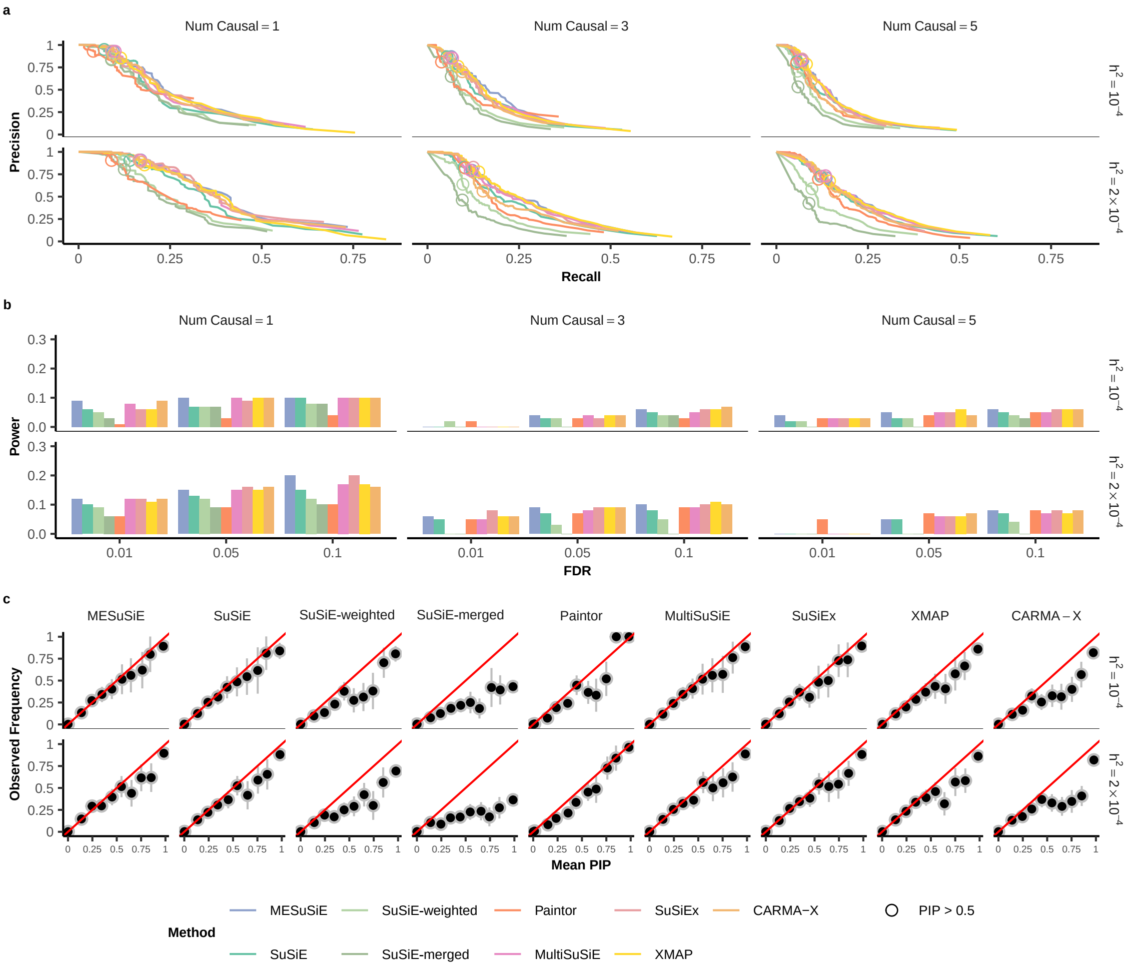

Supplementary Figure 8: **Comparison of method performance in detecting causal signals with non-zero effects in at least one ancestry (Genetic architecture driven simulation scenario, 50,000 Black British samples, 'Either').** Results are stratified by the number of cross-ancestry causal SNPs (1, 3, or 5, corresponding to a total of 2, 6, and 10 causal SNPs, respectively) and per-causal SNP heritability ($h^2 = 10^{-4}$ or 2×10^{-4}; rows). **(a)** Precision-recall curves with PIP_{either} thresholds ranging from 0.01 to 1. Precision is defined as $1 - \text{FDR}$ and Recall as $\frac{\text{TP}}{\text{TP+FN}}$, where TP, FP, and FN represent true positives, false positives, and false negatives, respectively. The PIP threshold of 0.5 is marked with a circle. **(b)** Bar plots displaying power at controlled FDR levels of 0.01, 0.05, and 0.1. **(c)** Calibration plots aggregating $n = 300$ independent regions (combining all causal SNP scenarios). SNPs are ranked by PIP and divided into 10 equally sized bins. The y-axis shows the observed proportion of causal signals, and the x-axis shows the mean PIP within each bin. Gray error bars represent ± 2 standard errors. The red diagonal line indicates perfect calibration; points below the line suggest inflated PIPs (more false discoveries), while points above indicate conservative PIPs.

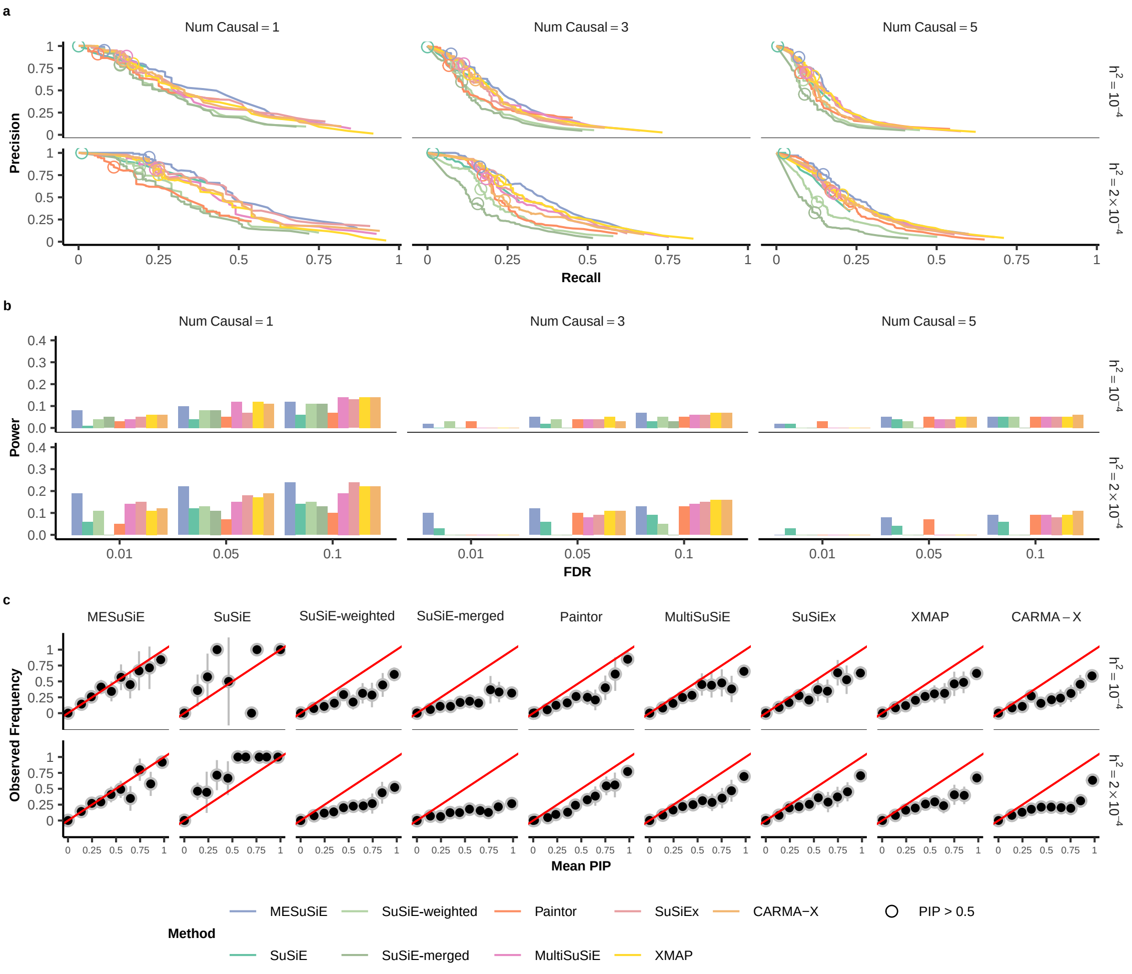

Supplementary Figure 9: **Comparison of method performance in detecting causal signals with non-zero effects in both ancestries (Genetic architecture driven simulation scenario, 50,000 Black British samples, 'Cross-ancestry').** Results are stratified by the number of cross-ancestry causal SNPs (1, 3, or 5, corresponding to a total of 2, 6, and 10 causal SNPs, respectively) and per-causal SNP heritability ($h^2 = 10^{-4}$ or 2×10^{-4}; rows). **(a)** Precision-recall curves with PIP_{shared} thresholds ranging from 0.01 to 1. Precision is defined as 1 − FDR and Recall as $\frac{\text{TP}}{\text{TP+FN}}$, where TP, FP, and FN represent true positives, false positives, and false negatives, respectively. The PIP threshold of 0.5 is marked with a circle. **(b)** Bar plots displaying power at controlled FDR levels of 0.01, 0.05, and 0.1. **(c)** Calibration plots aggregating $n = 300$ independent regions (combining all causal SNP scenarios). SNPs are ranked by PIP and divided into 10 equally sized bins. The y-axis shows the observed proportion of causal signals, and the x-axis shows the mean PIP within each bin. Gray error bars represent ±2 standard errors. The red diagonal line indicates perfect calibration; points below the line suggest inflated PIPs (more false discoveries), while points above indicate conservative PIPs.

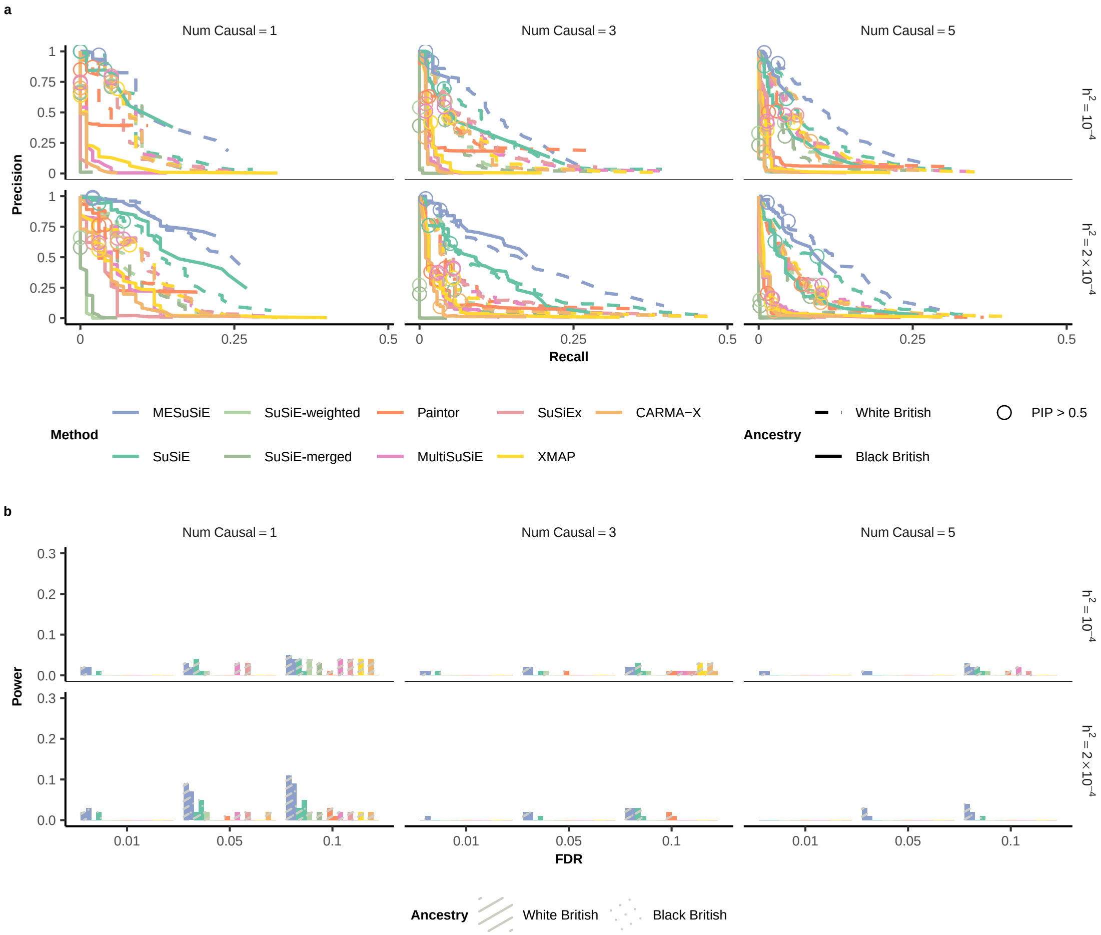

Supplementary Figure 10: **Performance metrics for ancestry-specific contextual causal signals (Genetic architecture driven simulation scenario, 50,000 Black British samples).** Comparison of method performance in detecting causal signals with non-zero effects in White British only and Black British only. Results are stratified by the number of cross-ancestry causal SNPs (1, 3, or 5, corresponding to a total of 2, 6, and 10 causal SNPs, respectively) and per-causal SNP heritability ($h^2 = 10^{-4}$ or 2×10^{-4}; rows). **(a)** Precision-recall curves with PIP_{WB} and PIP_{BB} thresholds ranging from 0.01 to 1. Precision is defined as $1 - \text{FDR}$ and Recall as $\frac{\text{TP}}{\text{TP+FN}}$, where TP, FP, and FN represent true positives, false positives, and false negatives, respectively. The PIP threshold of 0.5 is marked with a circle. **(b)** Bar plots displaying power at controlled FDR levels of 0.01, 0.05, and 0.1. **(c)** Calibration plots aggregating $n = 300$ independent regions (combining all causal SNP scenarios). SNPs are ranked by PIP and divided into 10 equally sized bins. The y-axis shows the observed proportion of causal signals, and the x-axis shows the mean PIP within each bin. Gray error bars represent ± 2 standard errors. The red diagonal line indicates perfect calibration.

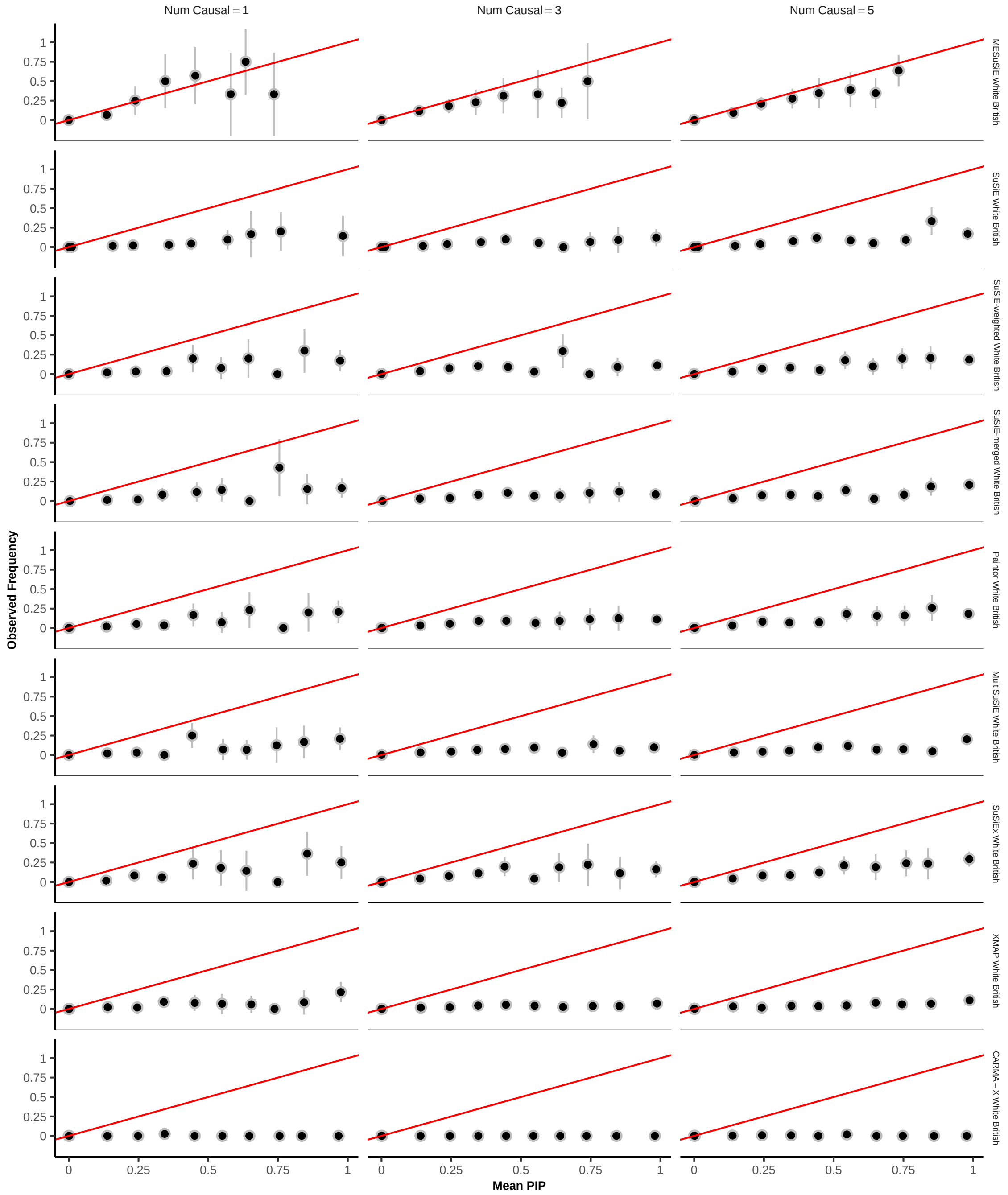

Supplementary Figure 11: **PIP Calibration for White British (WB) Ancestry (Genetic architecture driven simulation scenario, 50,000 Black British samples).** Calibration plots aggregating $n = 300$ independent regions (combining all causal SNP scenarios). SNPs are ranked by PIP and divided into 10 equally sized bins. The y-axis shows the observed proportion of causal signals, and the x-axis shows the mean PIP within each bin. Gray error bars represent ± 2 standard errors. The red diagonal line indicates perfect calibration; points below the line suggest inflated PIPs (more false discoveries), while points above indicate conservative PIPs.

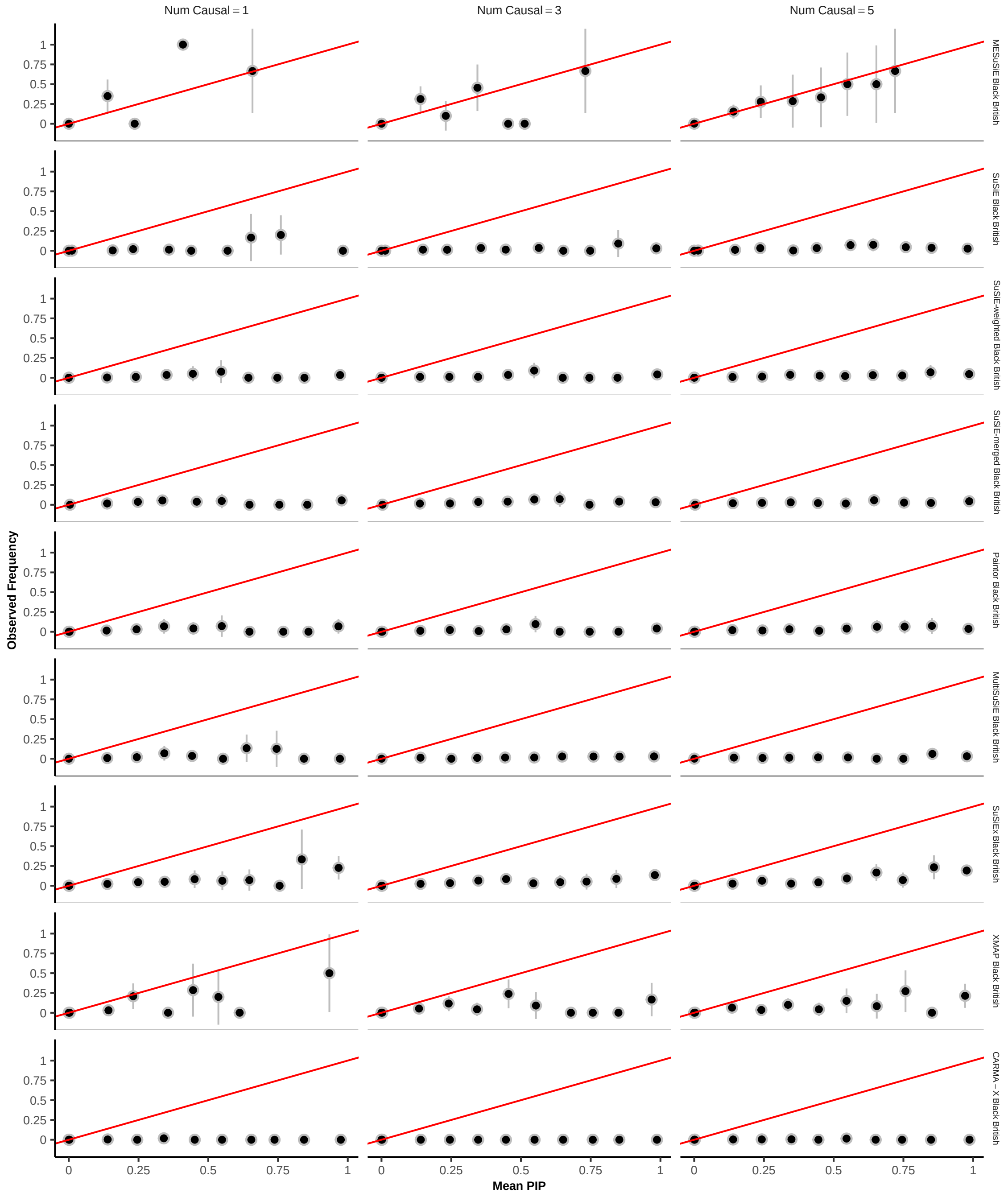

Supplementary Figure 12: **PIP Calibration for Black British (BB) Ancestry (Genetic architecture driven simulation scenario, 50,000 Black British samples).** Calibration plots aggregating $n = 300$ independent regions (combining all causal SNP scenarios). SNPs are ranked by PIP and divided into 10 equally sized bins. The y-axis shows the observed proportion of causal signals, and the x-axis shows the mean PIP within each bin. Gray error bars represent ± 2 standard errors. The red diagonal line indicates perfect calibration; points below the line suggest inflated PIPs (more false discoveries), while points above indicate conservative PIPs.

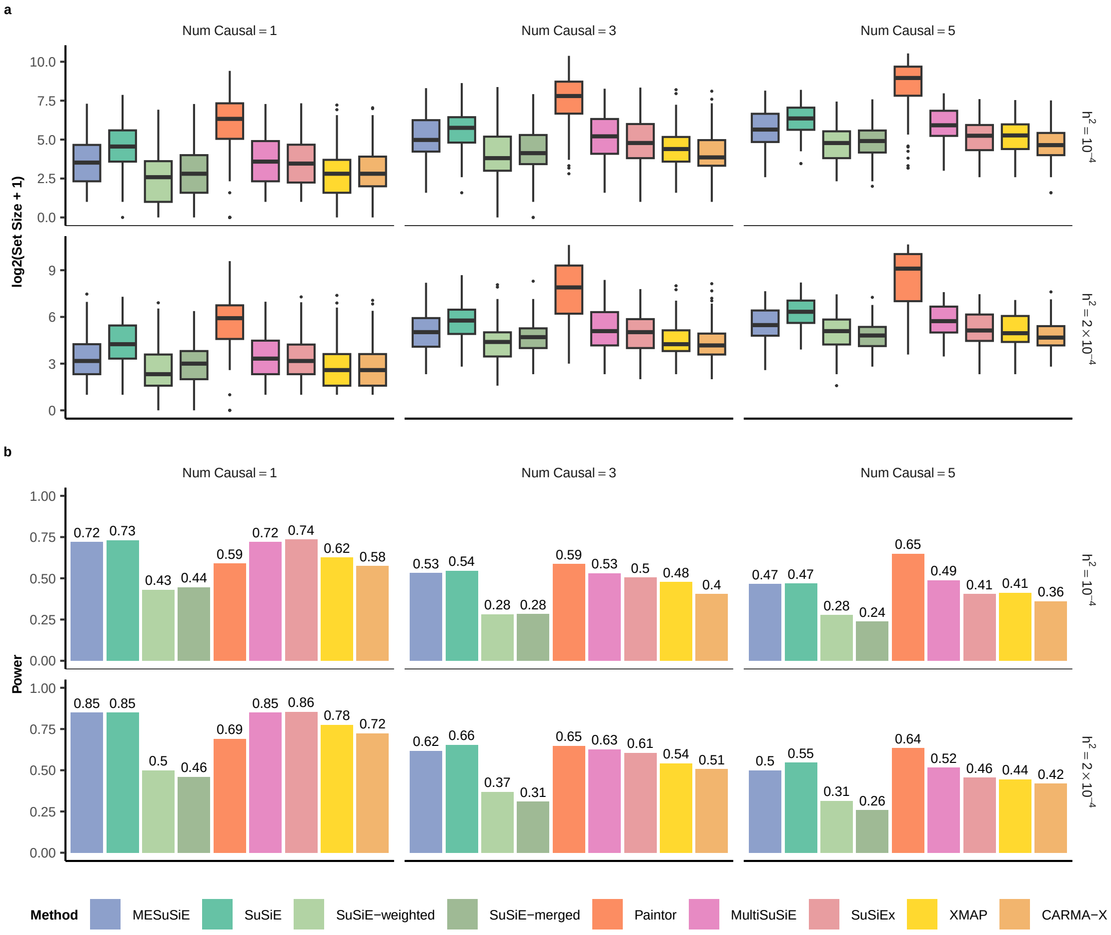

Supplementary Figure 13: **Performance metrics based on credible sets (Genetic architecture driven simulation scenario, external LD reference).** Comparison of 95% credible set sizes and power in detecting causal signals with non-zero effects in at least one ancestry. Results are summarized across $n = 100$ independent regions, stratified by the number of causal SNPs (1, 3, or 5, corresponding to a total of 2, 6, and 10 causal SNPs, respectively) and per-causal SNP heritability ($h^2 = 10^{-4}$ or 2×10^{-4}; rows). **(a)** Boxplots displaying the $\log_2(\text{set size} + 1)$ of the 95% credible sets. **(b)** Bar plots displaying the power of the constructed 95% credible sets, defined as the proportion of simulated causal variants successfully included in the credible set.

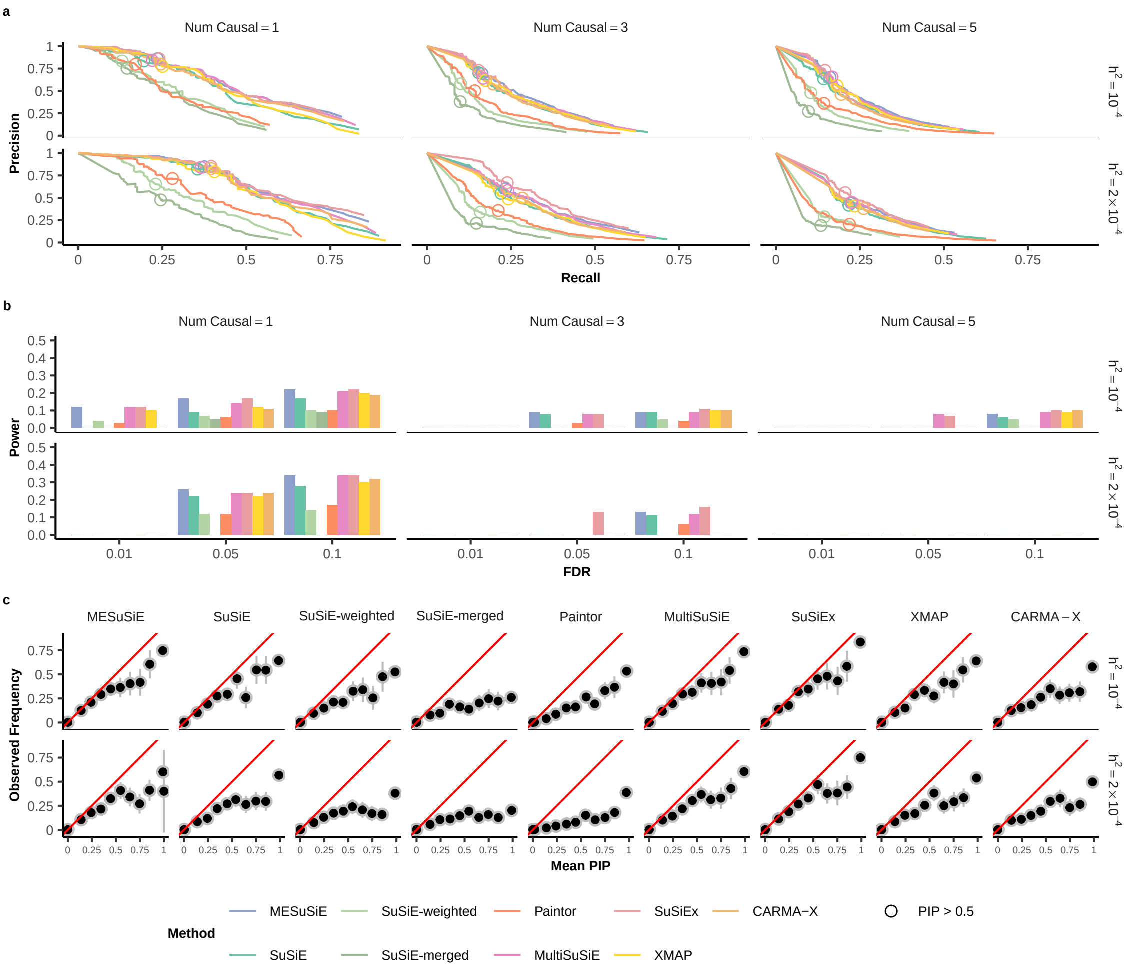

Supplementary Figure 14: **Comparison of method performance in detecting causal signals with non-zero effects in at least one ancestry (Genetic architecture driven simulation scenario, external LD reference, 'Either').** Results are stratified by the number of cross-ancestry causal SNPs (1, 3, or 5, corresponding to a total of 2, 6, and 10 causal SNPs, respectively) and per-causal SNP heritability ($h^2 = 10^{-4}$ or 2×10^{-4}; rows). **(a)** Precision-recall curves with PIP_{either} thresholds ranging from 0.01 to 1. Precision is defined as $1 - \text{FDR}$ and Recall as $\frac{\text{TP}}{\text{TP+FN}}$, where TP, FP, and FN represent true positives, false positives, and false negatives, respectively. The PIP threshold of 0.5 is marked with a circle. **(b)** Bar plots displaying power at controlled FDR levels of 0.01, 0.05, and 0.1. **(c)** Calibration plots aggregating $n = 300$ independent regions (combining all causal SNP scenarios). SNPs are ranked by PIP and divided into 10 equally sized bins. The y-axis shows the observed proportion of causal signals, and the x-axis shows the mean PIP within each bin. Gray error bars represent ± 2 standard errors. The red diagonal line indicates perfect calibration; points below the line suggest inflated PIPs (more false discoveries), while points above indicate conservative PIPs.

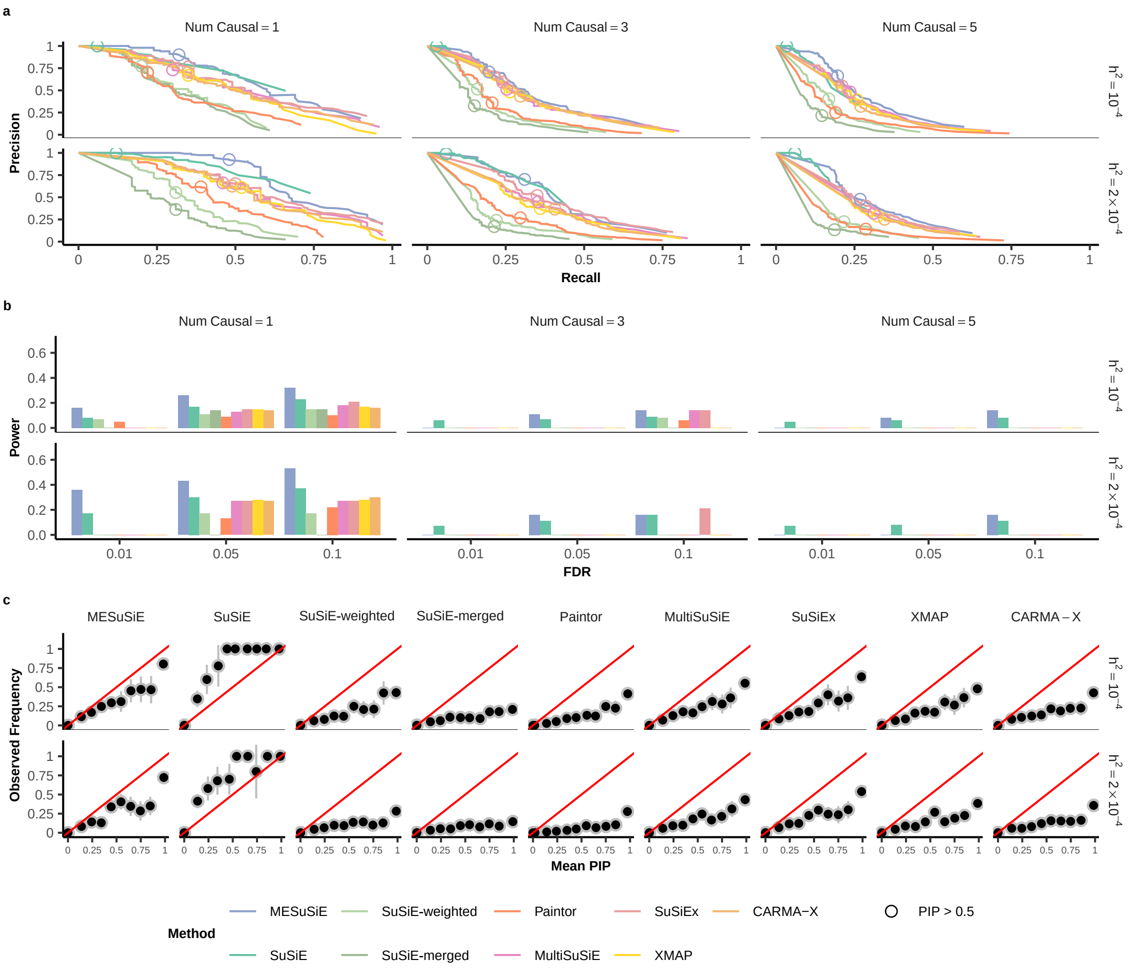

Supplementary Figure 15: **Comparison of method performance in detecting causal signals with non-zero effects in both ancestries (Genetic architecture driven simulation scenario, external LD reference, 'Cross-ancestry').** Results are stratified by the number of cross-ancestry causal SNPs (1, 3, or 5, corresponding to a total of 2, 6, and 10 causal SNPs, respectively) and per-causal SNP heritability ($h^2 = 10^{-4}$ or 2×10^{-4}; rows). **(a)** Precision-recall curves with PIP_{shared} thresholds ranging from 0.01 to 1. Precision is defined as 1 − FDR and Recall as $\frac{\text{TP}}{\text{TP+FN}}$, where TP, FP, and FN represent true positives, false positives, and false negatives, respectively. The PIP threshold of 0.5 is marked with a circle. **(b)** Bar plots displaying power at controlled FDR levels of 0.01, 0.05, and 0.1. **(c)** Calibration plots aggregating $n = 300$ independent regions (combining all causal SNP scenarios). SNPs are ranked by PIP and divided into 10 equally sized bins. The y-axis shows the observed proportion of causal signals, and the x-axis shows the mean PIP within each bin. Gray error bars represent ±2 standard errors. The red diagonal line indicates perfect calibration; points below the line suggest inflated PIPs (more false discoveries), while points above indicate conservative PIPs.

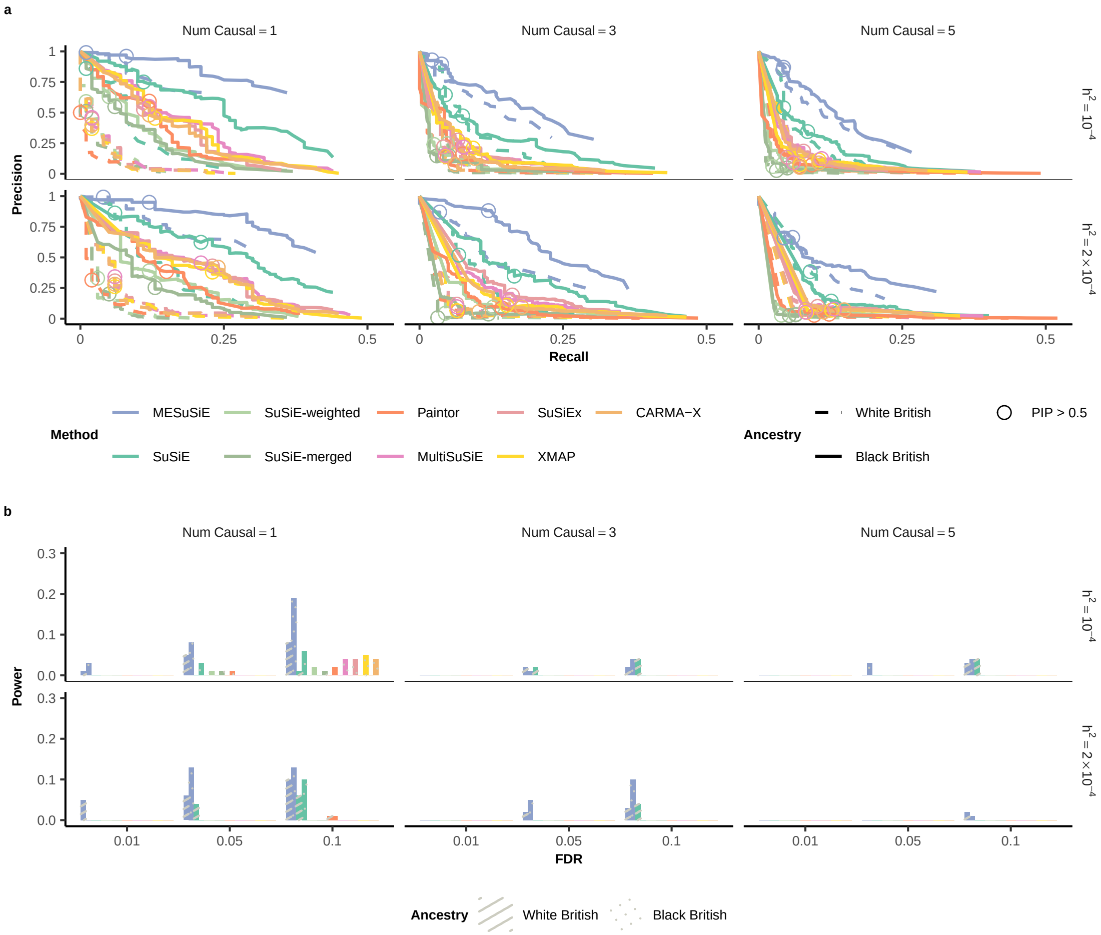

Supplementary Figure 16: **Performance metrics for ancestry-specific contextual causal signals (Genetic architecture driven simulation scenario, external LD reference).** Comparison of method performance in detecting causal signals with non-zero effects in White British only and Black British only. Results are stratified by the number of cross-ancestry causal SNPs (1, 3, or 5, corresponding to a total of 2, 6, and 10 causal SNPs, respectively) and per-causal SNP heritability ($h^2 = 10^{-4}$ or 2×10^{-4}; rows). **(a)** Precision-recall curves with PIP_{WB} and PIP_{BB} thresholds ranging from 0.01 to 1. Precision is defined as $1 - \text{FDR}$ and Recall as $\frac{\text{TP}}{\text{TP+FN}}$, where TP, FP, and FN represent true positives, false positives, and false negatives, respectively. The PIP threshold of 0.5 is marked with a circle. **(b)** Bar plots displaying power at controlled FDR levels of 0.01, 0.05, and 0.1.

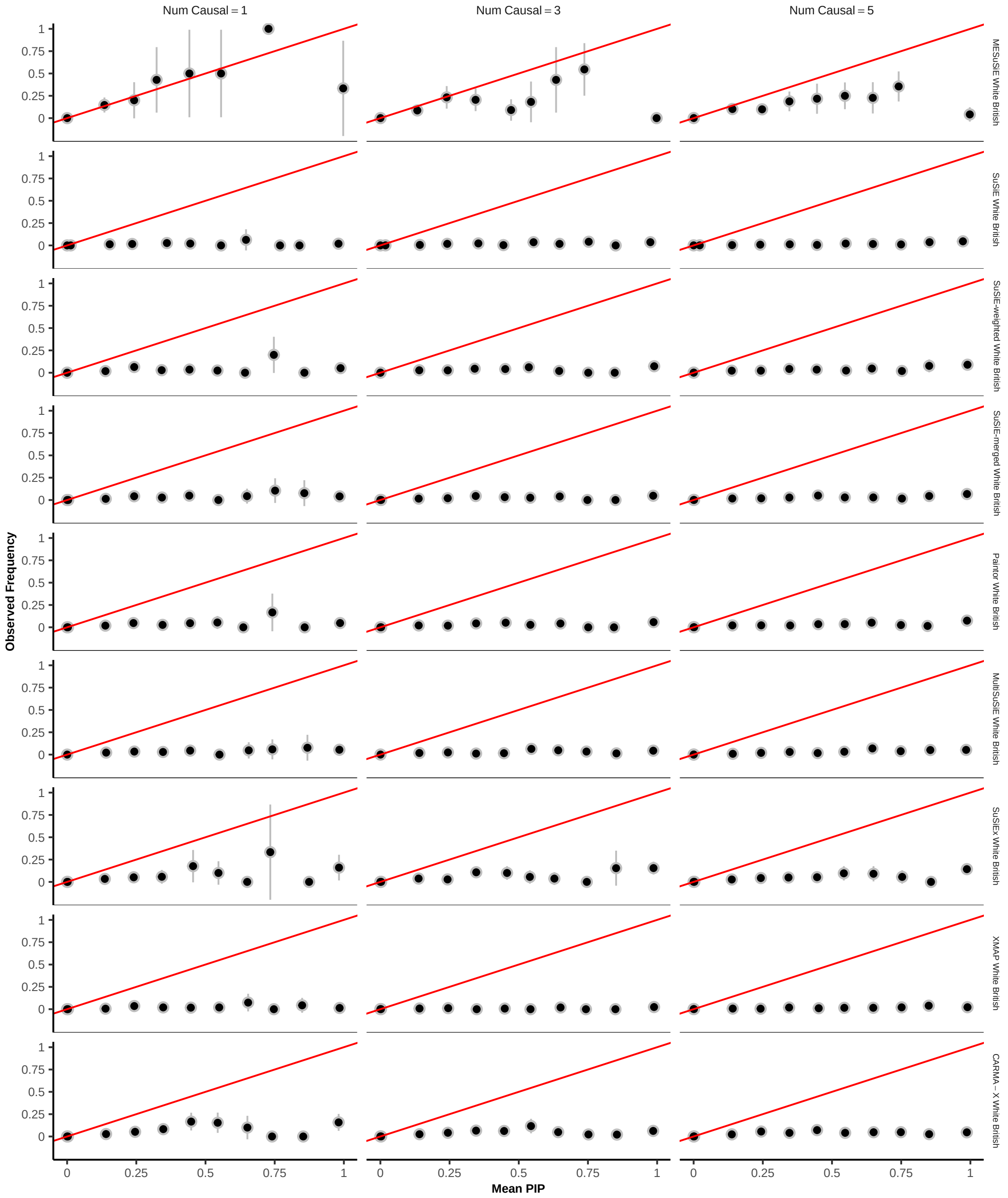

Supplementary Figure 17: **PIP Calibration for White British (WB) Ancestry (Genetic architecture driven simulation scenario, external LD reference).** Calibration plots aggregating $n = 300$ independent regions (combining all causal SNP scenarios). SNPs are ranked by PIP and divided into 10 equally sized bins. The y-axis shows the observed proportion of causal signals, and the x-axis shows the mean PIP within each bin. Gray error bars represent ± 2 standard errors. The red diagonal line indicates perfect calibration; points below the line suggest inflated PIPs (more false discoveries), while points above indicate conservative PIPs.

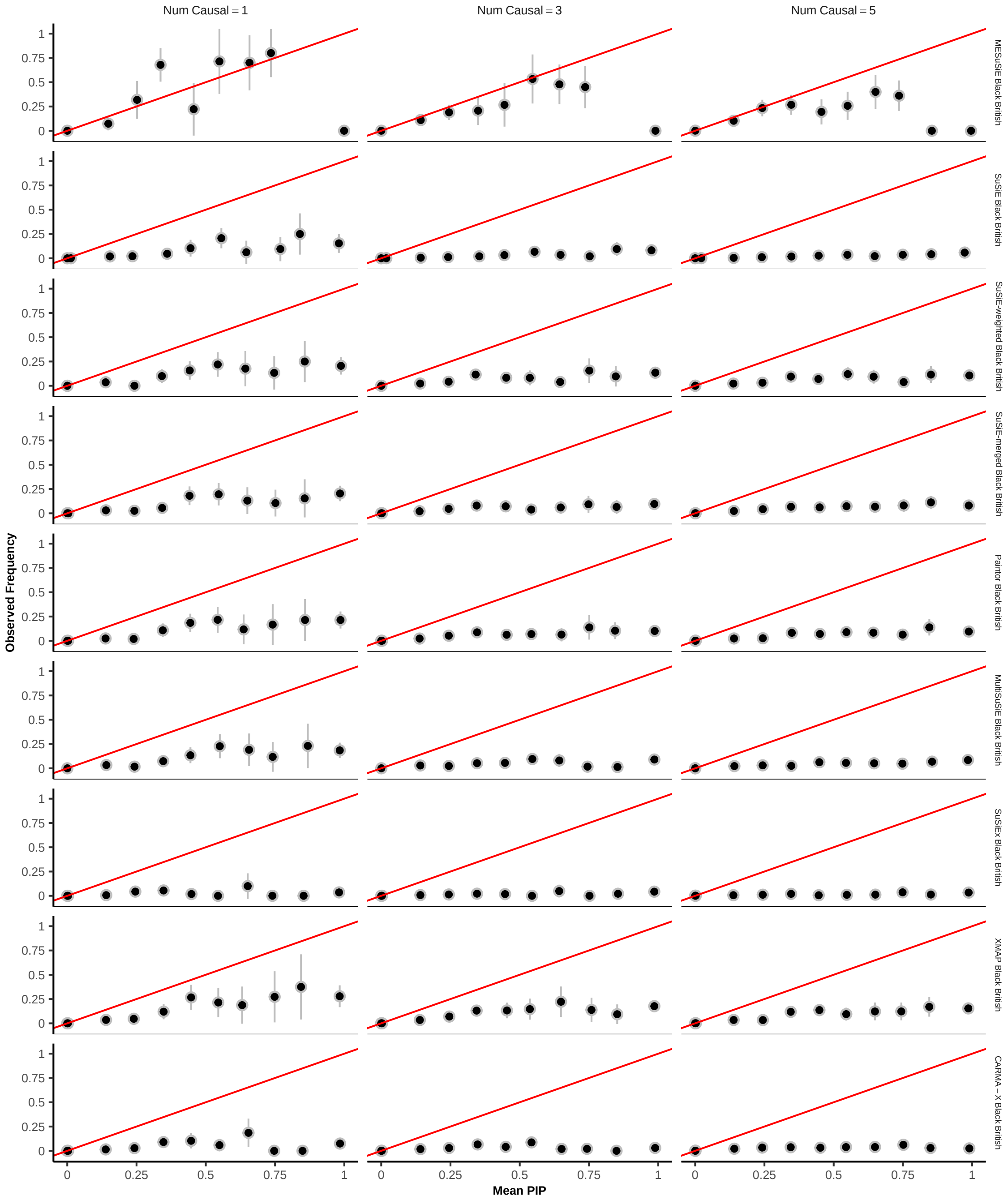

Supplementary Figure 18: **PIP Calibration for Black British (BB) Ancestry (Genetic architecture driven simulation scenario, external LD reference).** Calibration plots aggregating $n = 300$ independent regions (combining all causal SNP scenarios). SNPs are ranked by PIP and divided into 10 equally sized bins. The y-axis shows the observed proportion of causal signals, and the x-axis shows the mean PIP within each bin. Gray error bars represent ± 2 standard errors. The red diagonal line indicates perfect calibration; points below the line suggest inflated PIPs (more false discoveries), while points above indicate conservative PIPs.

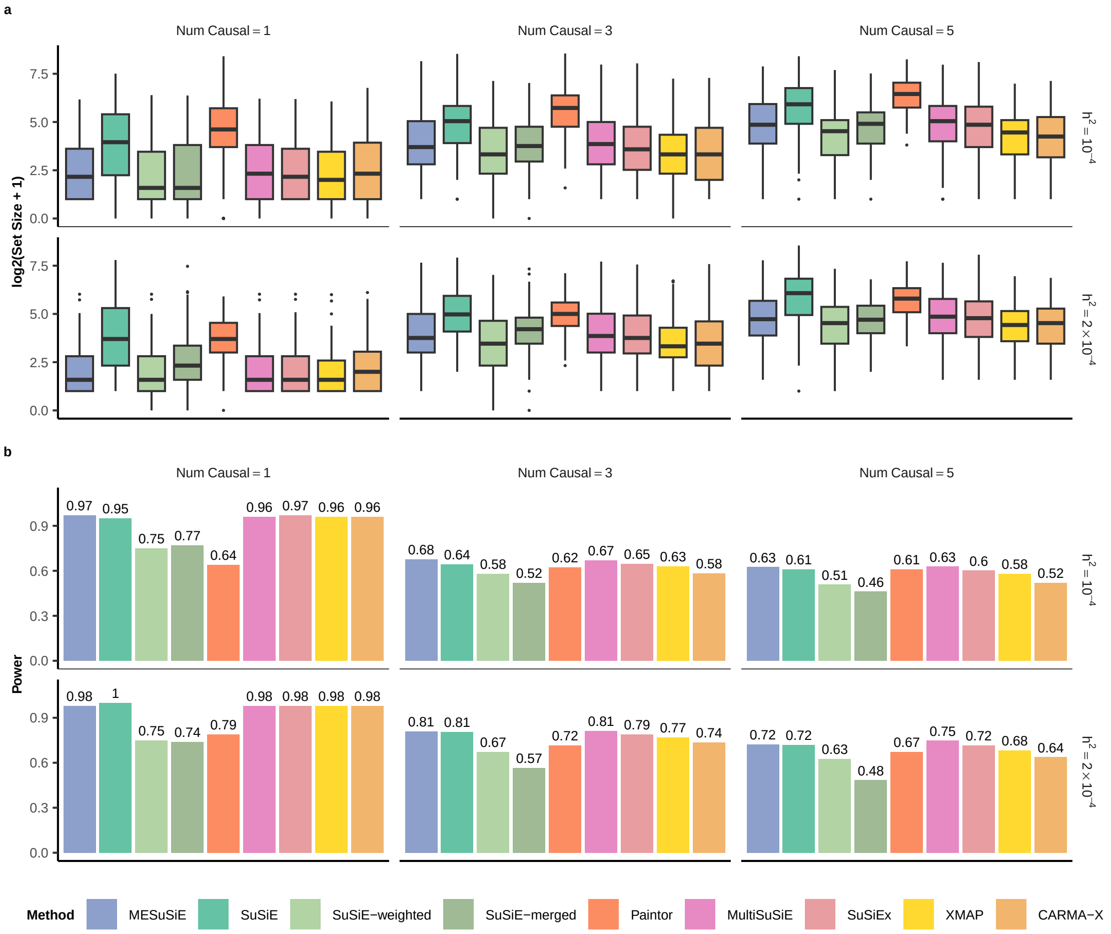

Supplementary Figure 19: **Performance metrics based on credible sets (Genetic architecture driven simulation scenario, 100% cross-ancestry causal variants).** Comparison of 95% credible set sizes and power in detecting causal signals with non-zero effects in at least one ancestry. Results are summarized across $n = 100$ independent regions, stratified by the number of causal SNPs (1, 3, or 5, corresponding to a total of 2, 6, and 10 causal SNPs, respectively) and per-causal SNP heritability ($h^2 = 10^{-4}$ or 2×10^{-4}; rows). **(a)** Boxplots displaying the $\log_2$(set size + 1) of the 95% credible sets. **(b)** Bar plots displaying the power of the constructed 95% credible sets, defined as the proportion of simulated causal variants successfully included in the credible set.

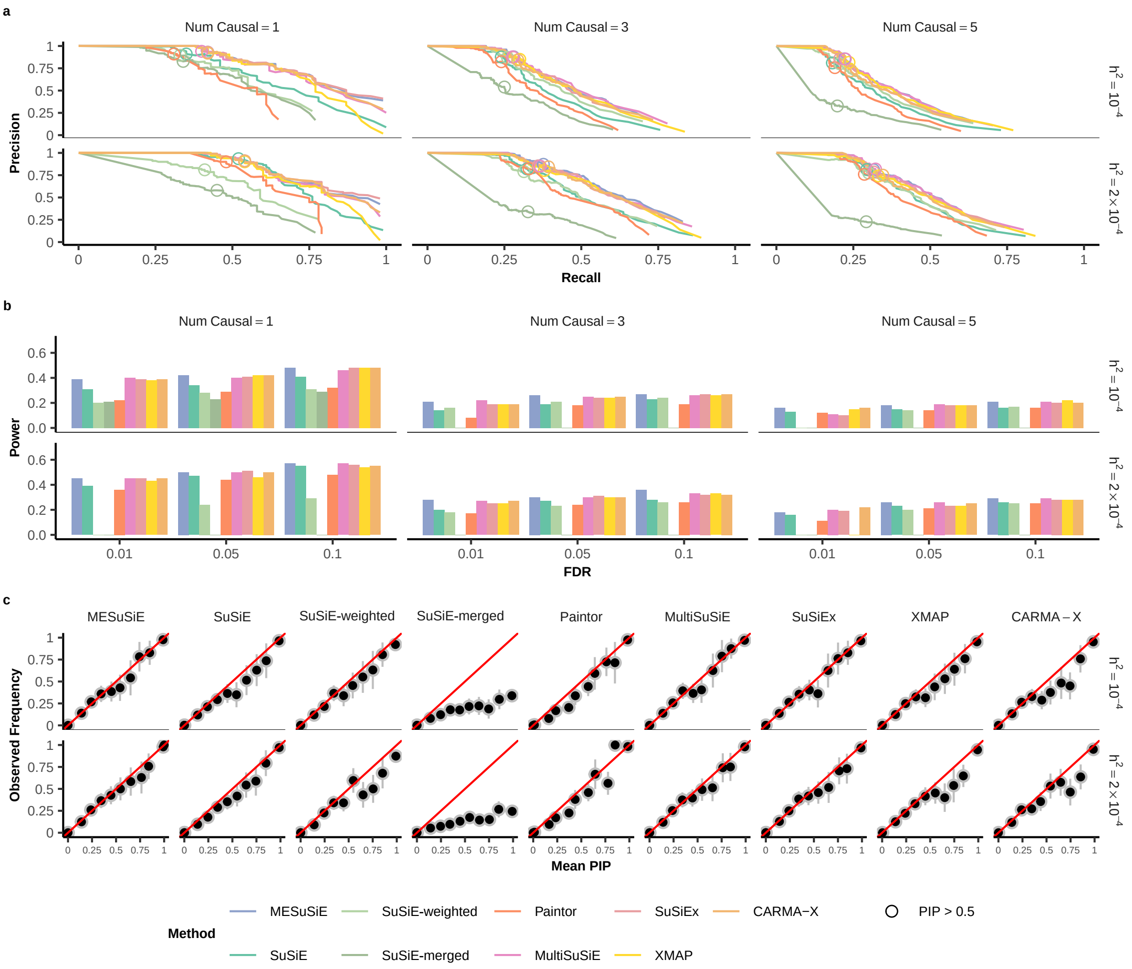

Supplementary Figure 20: **Comparison of method performance in detecting causal signals with non-zero effects in at least one ancestry (Genetic architecture driven simulation scenario, 100% cross-ancestry causal variants, 'Either').** Results are stratified by the number of cross-ancestry causal SNPs (1, 3, or 5, corresponding to a total of 2, 6, and 10 causal SNPs, respectively) and per-causal SNP heritability ($h^2 = 10^{-4}$ or 2×10^{-4}; rows). **(a)** Precision-recall curves with PIP_{either} thresholds ranging from 0.01 to 1. Precision is defined as $1 - \text{FDR}$ and Recall as $\frac{\text{TP}}{\text{TP+FN}}$, where TP, FP, and FN represent true positives, false positives, and false negatives, respectively. The PIP threshold of 0.5 is marked with a circle. **(b)** Bar plots displaying power at controlled FDR levels of 0.01, 0.05, and 0.1. **(c)** Calibration plots aggregating $n = 300$ independent regions (combining all causal SNP scenarios). SNPs are ranked by PIP and divided into 10 equally sized bins. The y-axis shows the observed proportion of causal signals, and the x-axis shows the mean PIP within each bin. Gray error bars represent ± 2 standard errors. The red diagonal line indicates perfect calibration; points below the line suggest inflated PIPs (more false discoveries), while points above indicate conservative PIPs.

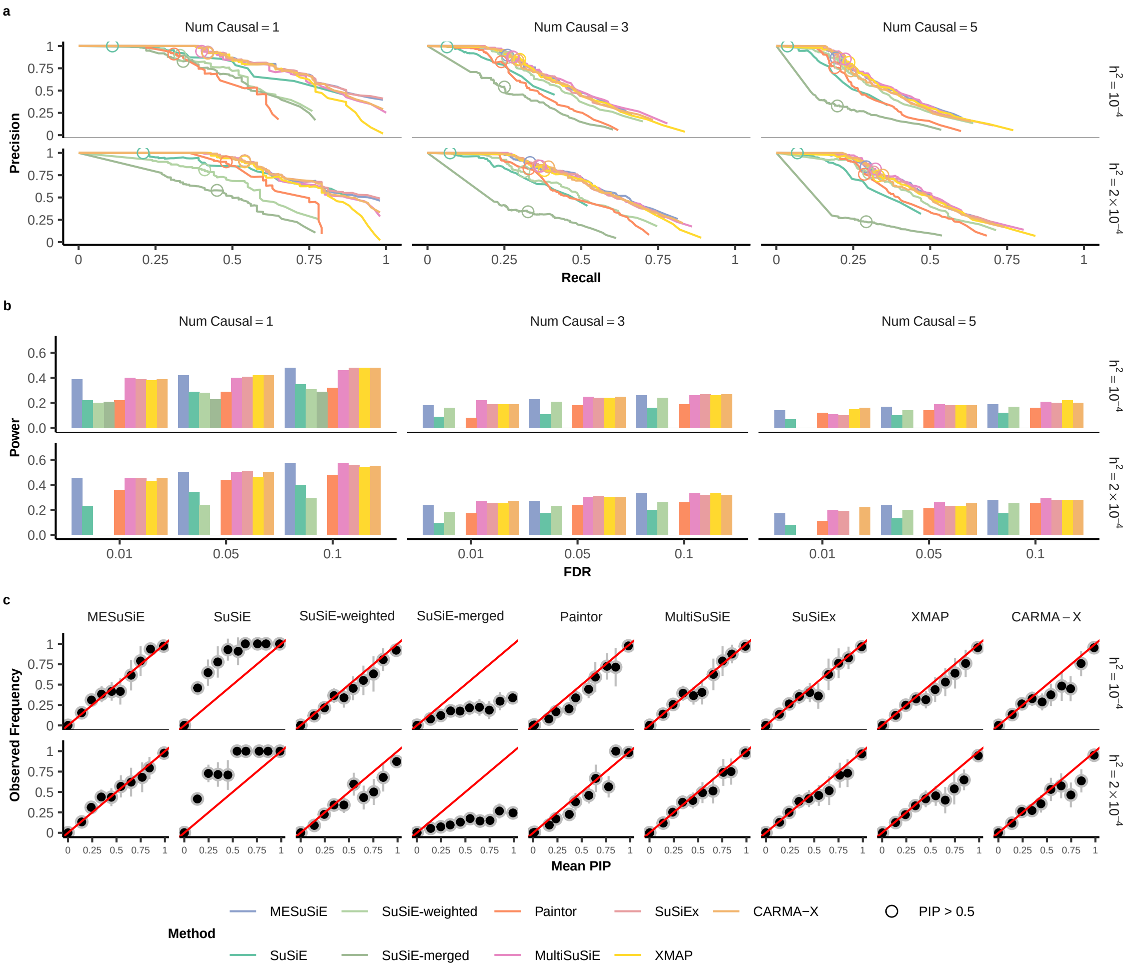

Supplementary Figure 21: **Comparison of method performance in detecting causal signals with non-zero effects in both ancestries (Genetic architecture driven simulation scenario, 100% cross-ancestry causal variants, 'Cross-ancestry').** Results are stratified by the number of cross-ancestry causal SNPs (1, 3, or 5, corresponding to a total of 2, 6, and 10 causal SNPs, respectively) and per-causal SNP heritability ($h^2 = 10^{-4}$ or 2×10^{-4}; rows). **(a)** Precision-recall curves with PIP_{shared} thresholds ranging from 0.01 to 1. Precision is defined as $1 - \text{FDR}$ and Recall as $\frac{\text{TP}}{\text{TP+FN}}$, where TP, FP, and FN represent true positives, false positives, and false negatives, respectively. The PIP threshold of 0.5 is marked with a circle. **(b)** Bar plots displaying power at controlled FDR levels of 0.01, 0.05, and 0.1. **(c)** Calibration plots aggregating $n = 300$ independent regions (combining all causal SNP scenarios). SNPs are ranked by PIP and divided into 10 equally sized bins. The y-axis shows the observed proportion of causal signals, and the x-axis shows the mean PIP within each bin. Gray error bars represent ± 2 standard errors. The red diagonal line indicates perfect calibration; points below the line suggest inflated PIPs (more false discoveries), while points above indicate conservative PIPs.

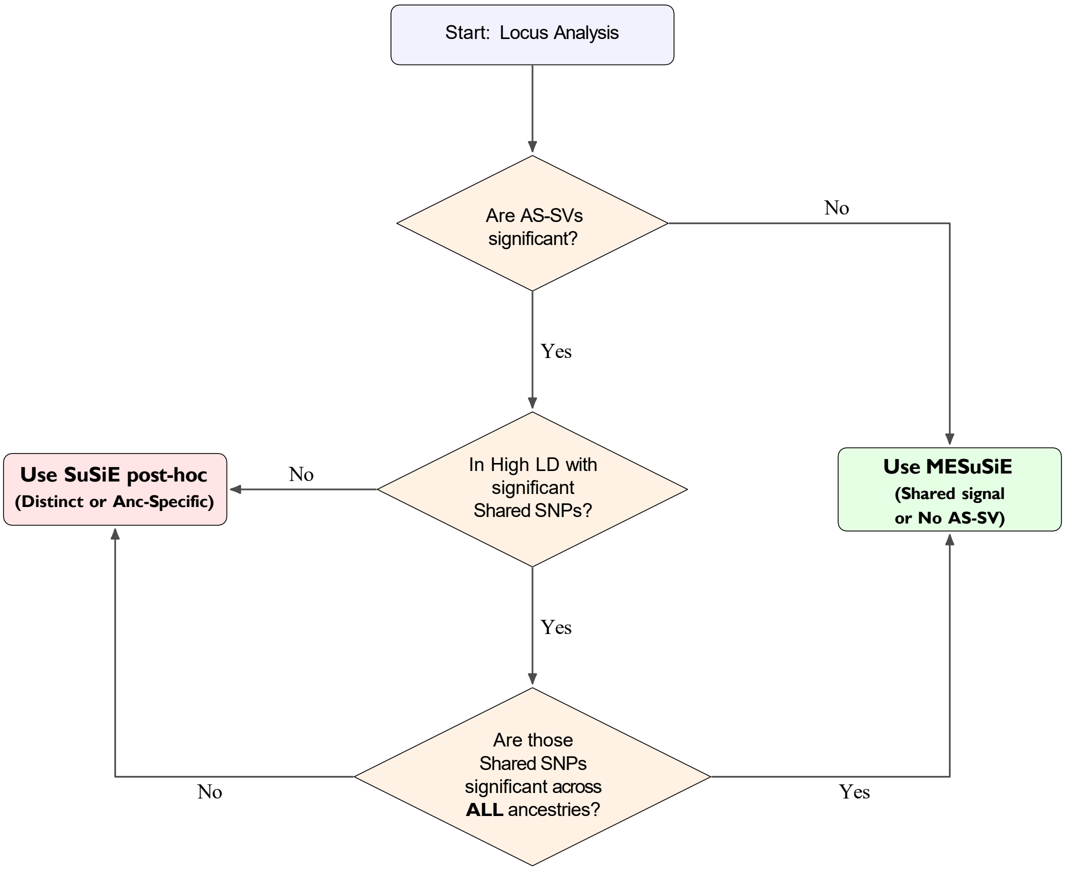

Supplementary Figure 22: **Decision tree for the Multi-ancestry Fine-mapping Decision (MFD) strategy.** This flowchart illustrates the hierarchical logic used to select the appropriate fine-mapping strategy for a given locus. The process begins by evaluating the significance of Ancestry-Specific Segregating Variants (AS-SVs). If no significant AS-SVs ($P < 5 \times 10^{-8}$) are present, or if they exist but are in high Linkage Disequilibrium (LD, $r^2 > 0.6$) with cross-ancestry SNPs that are significant across all ancestries, the method utilizes **MESuSiE** to characterize cross-ancestry signals. Conversely, if significant AS-SVs are distinct (not in high LD with cross-ancestry SNPs) or if the linked cross-ancestry SNPs are not significant across all ancestries, the framework employs a **SuSiE post-hoc** analysis to resolve ancestry-specific or distinct signals.

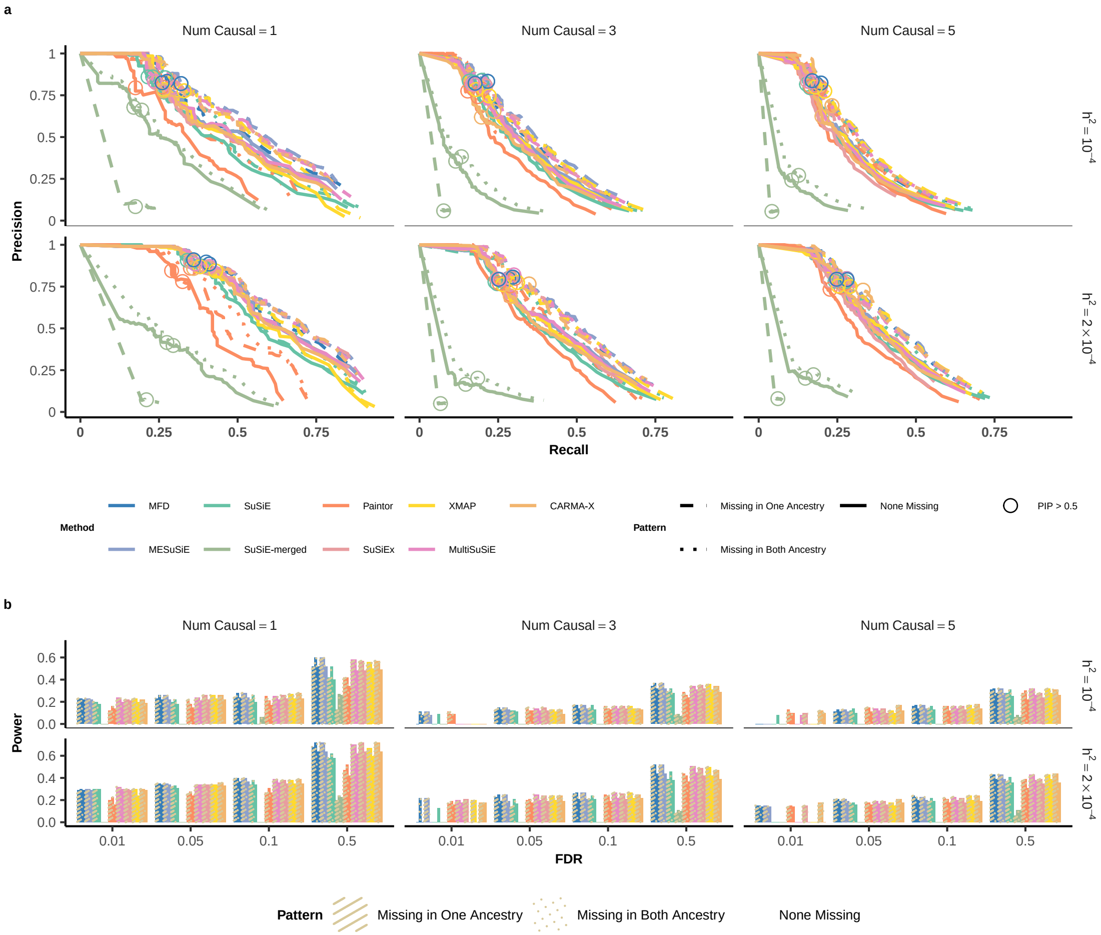

Supplementary Figure 23: **Comparison of method performance in detecting causal signals with non-zero effects in at least one ancestry with non-causal ancestry-specific variants from GWAS summary statistics in one (stripe) or both (circle) ancestries (Non-causal ancestry-specific variants from GWAS summary statistics, 'Either').** Results are stratified by the number of cross-ancestry causal SNPs (1, 3, or 5, corresponding to a total of 2, 6, and 10 causal SNPs, respectively) and per-causal SNP heritability ($h^2 = 10^{-4}$ or 2×10^{-4}; rows). **(a)** Precision-recall curves with PIP_{either} thresholds ranging from 0.01 to 1. Precision is defined as $1 - \text{FDR}$ and Recall as $\frac{\text{TP}}{\text{TP+FN}}$, where TP, FP, and FN represent true positives, false positives, and false negatives, respectively. The PIP threshold of 0.5 is marked with a circle. **(b)** Bar plots displaying power at controlled FDR levels of 0.01, 0.05, and 0.1 for each method.

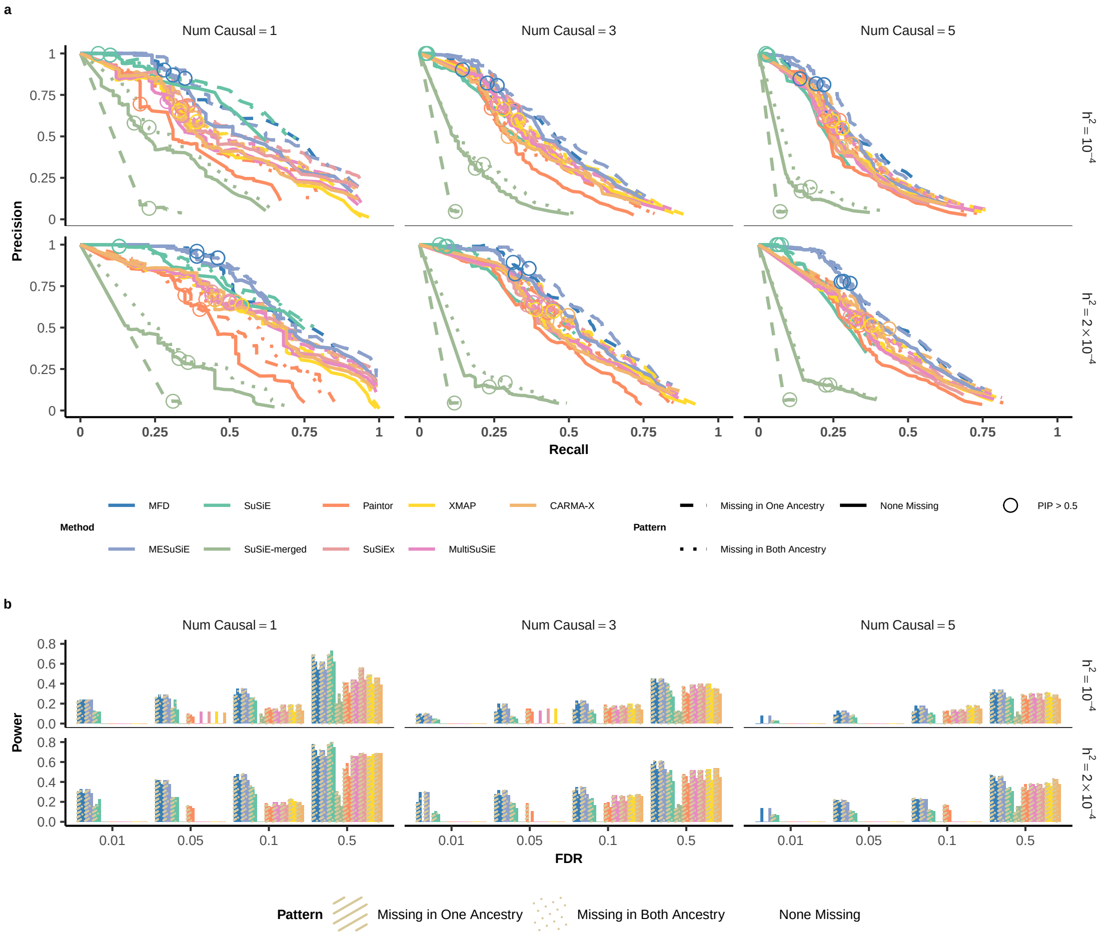

Supplementary Figure 24: **Comparison of method performance in detecting causal signals with non-zero effects in both ancestries with non-causal ancestry-specific variants from GWAS summary statistics in one (stripe) or both (circle) ancestries (Non-causal ancestry-specific variants from GWAS summary statistics, 'Cross-ancestry').** Results are stratified by the number of cross-ancestry causal SNPs (1, 3, or 5, corresponding to a total of 2, 6, and 10 causal SNPs, respectively) and per-causal SNP heritability ($h^2 = 10^{-4}$ or 2×10^{-4}; rows). **(a)** Precision-recall curves with PIP_{shared} thresholds ranging from 0.01 to 1. Precision is defined as $1 - \text{FDR}$ and Recall as $\frac{\text{TP}}{\text{TP+FN}}$, where TP, FP, and FN represent true positives, false positives, and false negatives, respectively. The PIP threshold of 0.5 is marked with a circle. **(b)** Bar plots displaying power at controlled FDR levels of 0.01, 0.05, and 0.1 for each method.

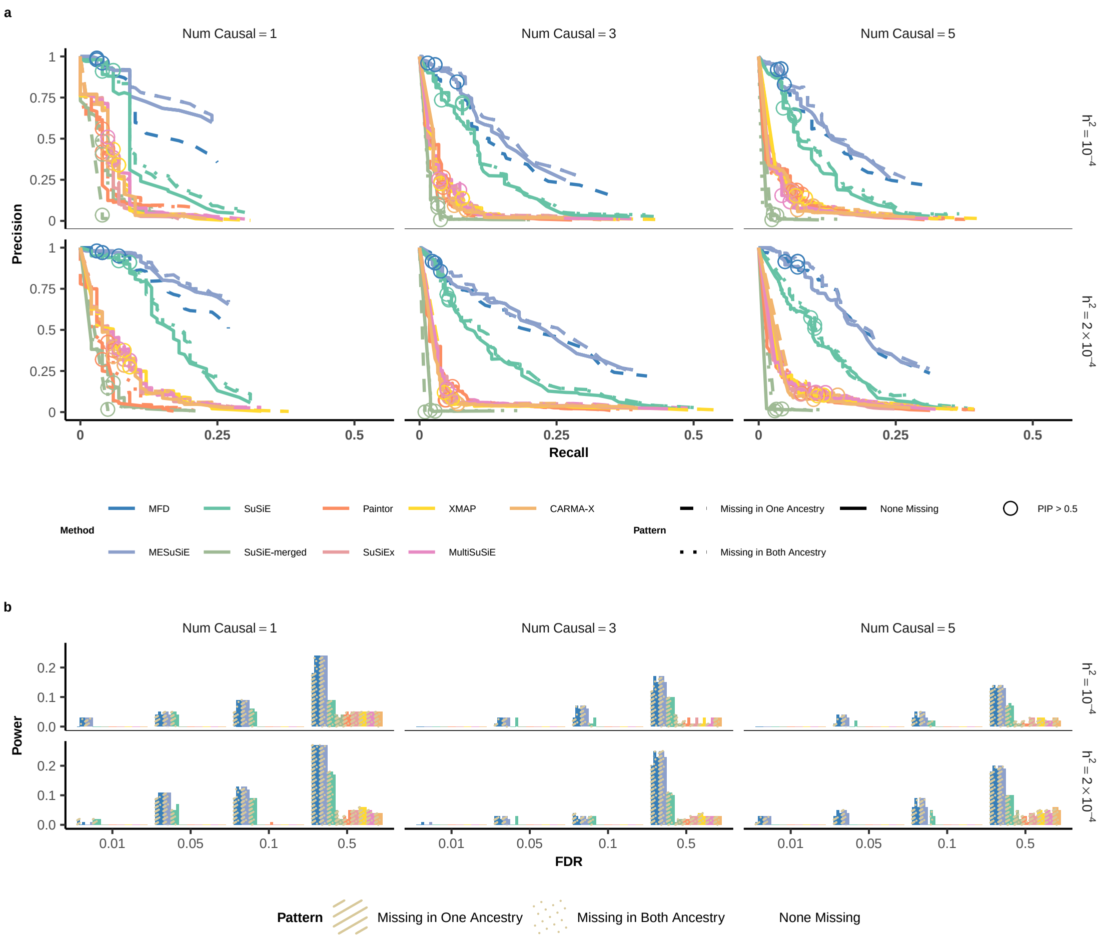

Supplementary Figure 25: **Performance metrics for White British (WB) contextual causal signals (Non-causal ancestry-specific variants from GWAS summary statistics).** Comparison of method performance in detecting causal signals with non-zero effects in WB only with non-causal ancestry-specific variants from GWAS summary statistics in one (stripe) or both (circle) ancestries. Results are stratified by the number of cross-ancestry causal SNPs (1, 3, or 5, corresponding to a total of 2, 6, and 10 causal SNPs, respectively) and per-causal SNP heritability ($h^2 = 10^{-4}$ or 2×10^{-4}; rows). **(a)** Precision-recall curves with PIP_{WB} thresholds ranging from 0.01 to 1. Precision is defined as $1 - \text{FDR}$ and Recall as $\frac{\text{TP}}{\text{TP+FN}}$, where TP, FP, and FN represent true positives, false positives, and false negatives, respectively. The PIP threshold of 0.5 is marked with a circle. **(b)** Bar plots displaying power at controlled FDR levels of 0.01, 0.05, and 0.1 for each method.

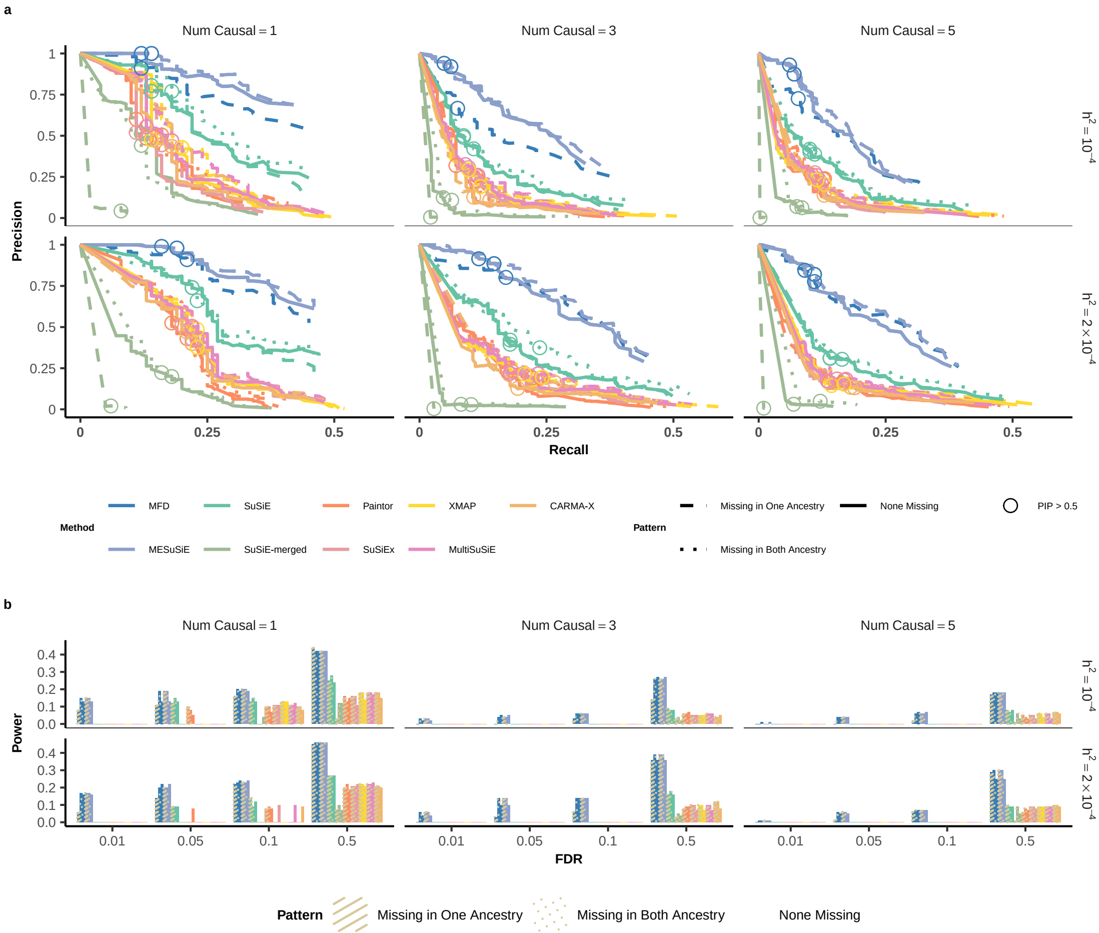

Supplementary Figure 26: **Performance metrics for Black British (BB) contextual causal signals (Non-causal ancestry-specific variants from GWAS summary statistics).** Comparison of method performance in detecting causal signals with non-zero effects in BB only with non-causal ancestry-specific variants from GWAS summary statistics in one (stripe) or both (circle) ancestries. Results are stratified by the number of cross-ancestry causal SNPs (1, 3, or 5, corresponding to a total of 2, 6, and 10 causal SNPs, respectively) and per-causal SNP heritability ($h^2 = 10^{-4}$ or 2×10^{-4}; rows). **(a)** Precision-recall curves with PIP_{BB} thresholds ranging from 0.01 to 1. Precision is defined as $1 - \text{FDR}$ and Recall as $\frac{\text{TP}}{\text{TP+FN}}$, where TP, FP, and FN represent true positives, false positives, and false negatives, respectively. The PIP threshold of 0.5 is marked with a circle. **(b)** Bar plots displaying power at controlled FDR levels of 0.01, 0.05, and 0.1 for each method.

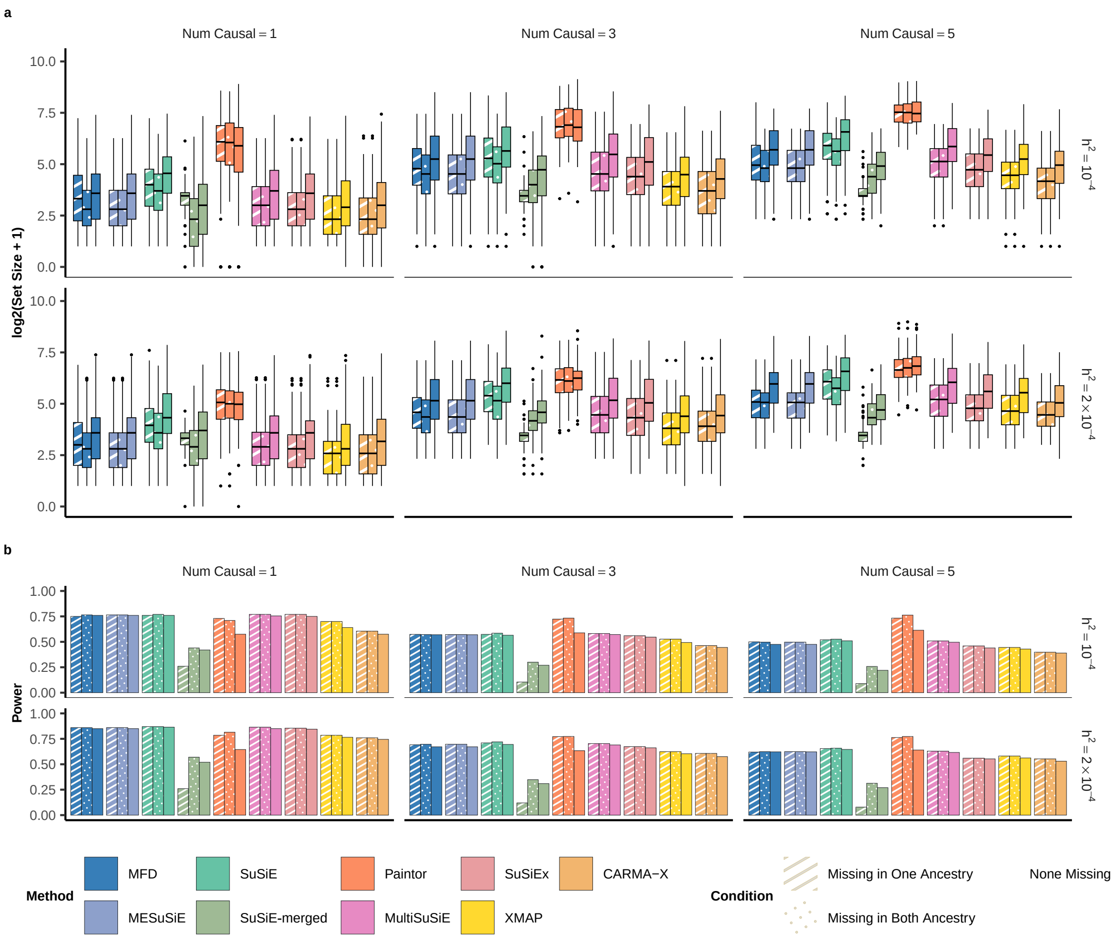

Supplementary Figure 27: **Performance metrics based on credible sets (Non-causal ancestry-specific variants from GWAS summary statistics and LD reference panel).** Comparison of 95% credible set sizes and power in detecting causal signals with non-zero effects in at least one ancestry when non-causal ancestry-specific variants from GWAS and reference panel in one (stripe) or both (circle). Results are summarized across $n = 100$ independent regions, stratified by the number of causal SNPs (1, 3, or 5, corresponding to a total of 2, 6, and 10 causal SNPs, respectively) and per-causal SNP heritability ($h^2 = 10^{-4}$ or 2×10^{-4}; rows). **(a)** Boxplots displaying the $\log_2(\text{set size} + 1)$ of the 95% credible sets. **(b)** Bar plots displaying the power of the constructed 95% credible sets, defined as the proportion of simulated causal variants successfully included in the credible set.

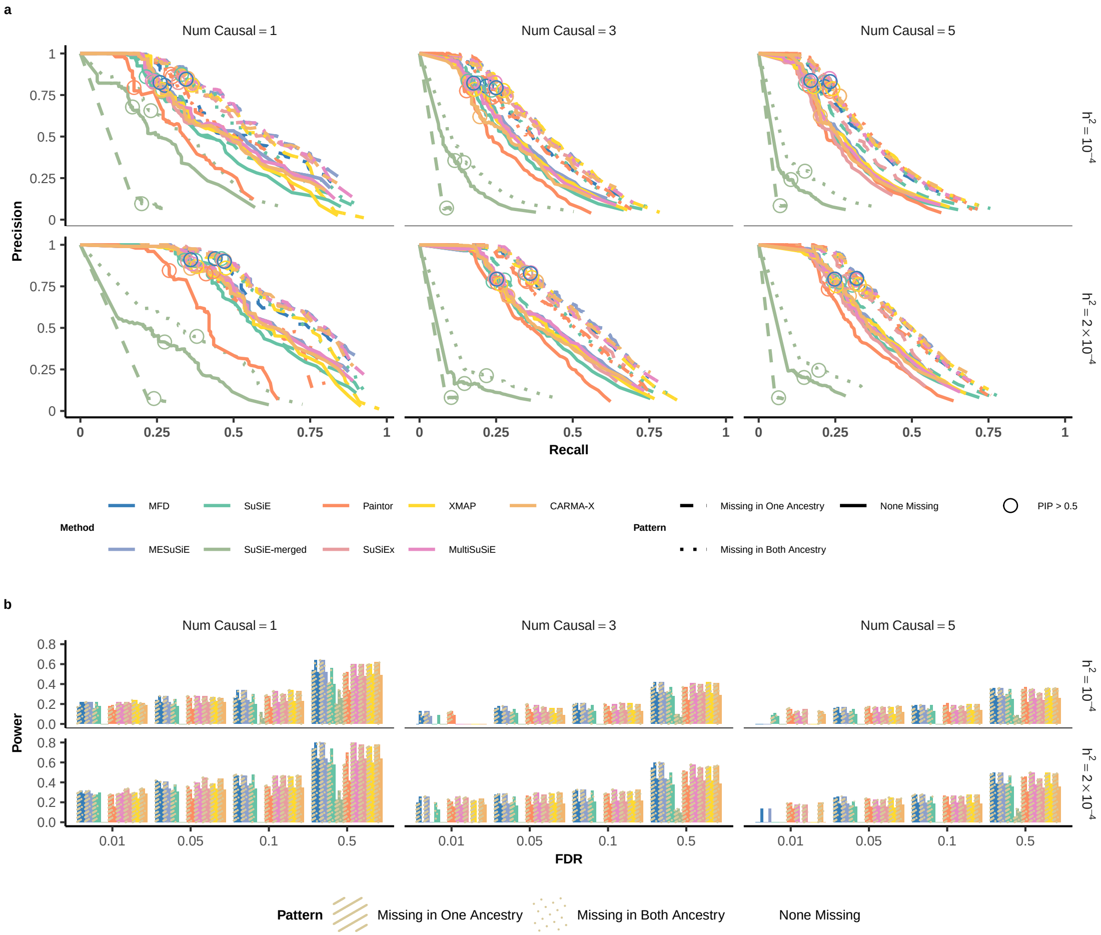

Supplementary Figure 28: **Comparison of method performance in detecting causal signals with non-zero effects in at least one ancestry with non-causal ancestry-specific variants from GWAS summary statistics and LD reference panel in one (stripe) or both (circle) ancestries (Non-causal ancestry-specific variants from GWAS summary statistics and LD reference panel, 'Either').** Results are stratified by the number of cross-ancestry causal SNPs (1, 3, or 5, corresponding to a total of 2, 6, and 10 causal SNPs, respectively) and per-causal SNP heritability ($h^2 = 10^{-4}$ or 2×10^{-4}; rows). **(a)** Precision-recall curves with PIP_{either} thresholds ranging from 0.01 to 1. Precision is defined as $1 - \text{FDR}$ and Recall as $\frac{\text{TP}}{\text{TP+FN}}$, where TP, FP, and FN represent true positives, false positives, and false negatives, respectively. The PIP threshold of 0.5 is marked with a circle. **(b)** Bar plots displaying power at controlled FDR levels of 0.01, 0.05, and 0.1 for each method.

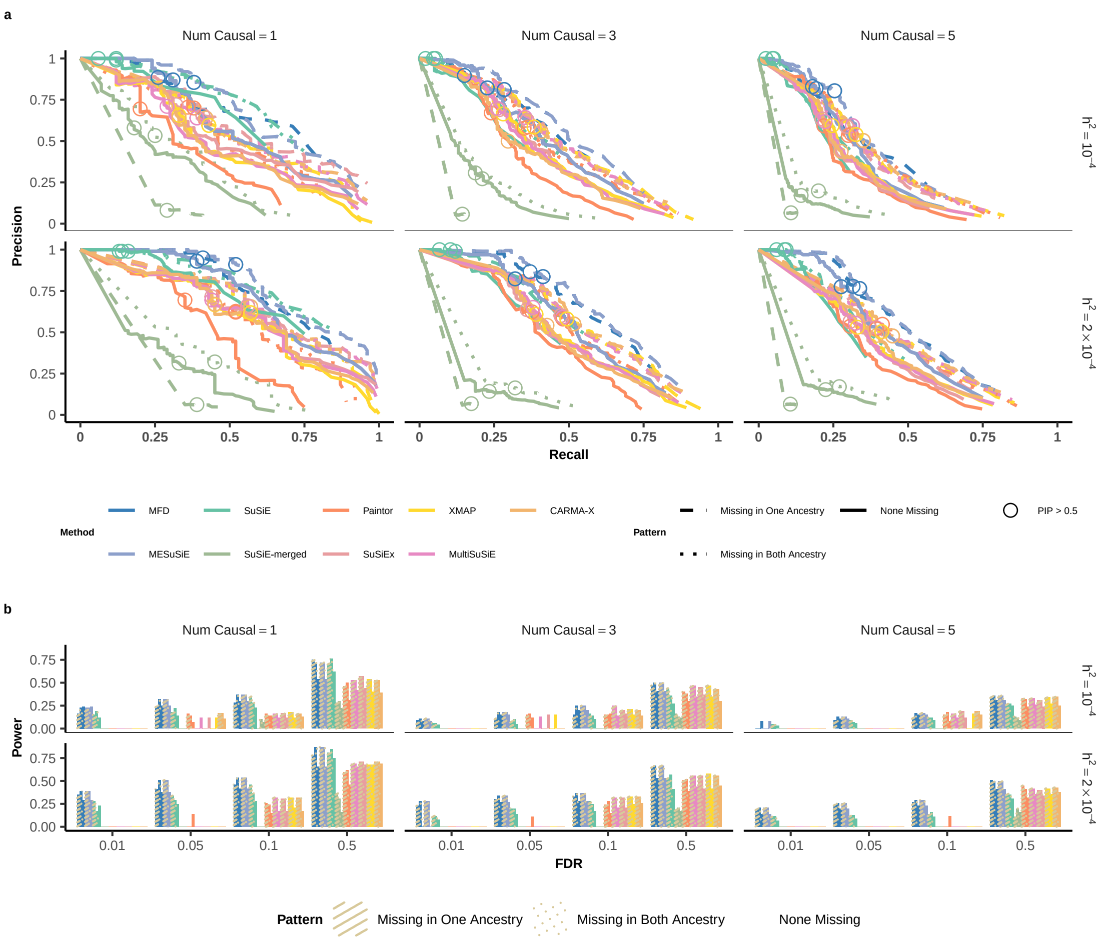

Supplementary Figure 29: **Comparison of method performance in detecting causal signals with non-zero effects in both ancestries with non-causal ancestry-specific variants from GWAS summary statistics and LD reference panel in one (stripe) or both (circle) ancestries (Non-causal ancestry-specific variants from GWAS summary statistics and LD reference panel, 'Cross-ancestry').** Results are stratified by the number of cross-ancestry causal SNPs (1, 3, or 5, corresponding to a total of 2, 6, and 10 causal SNPs, respectively) and per-causal SNP heritability ($h^2 = 10^{-4}$ or 2×10^{-4}; rows). **(a)** Precision-recall curves with PIP_{shared} thresholds ranging from 0.01 to 1. Precision is defined as $1 - \text{FDR}$ and Recall as $\frac{\text{TP}}{\text{TP+FN}}$, where TP, FP, and FN represent true positives, false positives, and false negatives, respectively. The PIP threshold of 0.5 is marked with a circle. **(b)** Bar plots displaying power at controlled FDR levels of 0.01, 0.05, and 0.1 for each method.

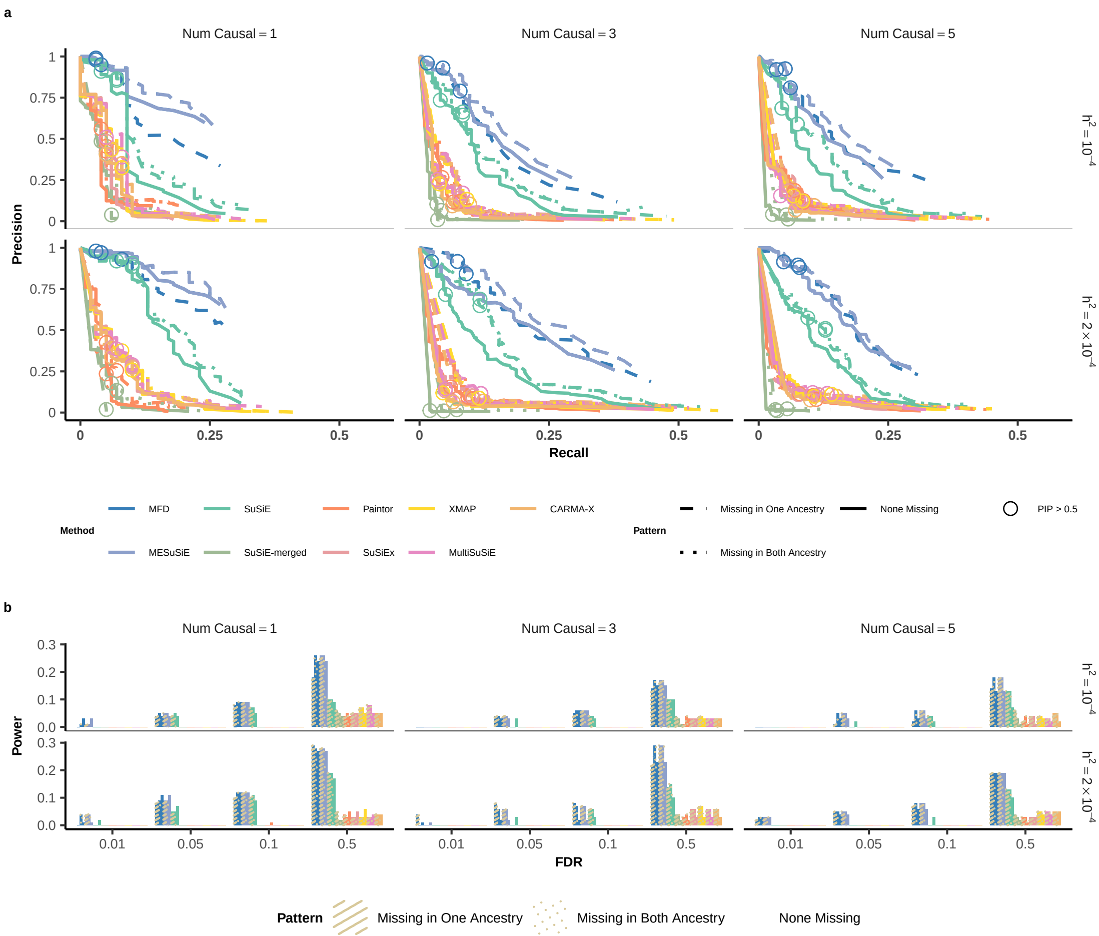

Supplementary Figure 30: **Performance metrics for White British (WB) contextual causal signals (Non-causal ancestry-specific variants from GWAS summary statistics and LD reference panel).** Comparison of method performance in detecting causal signals with non-zero effects in WB only with non-causal ancestry-specific variants from GWAS summary statistics and LD reference panel in one (stripe) or both (circle) ancestries. Results are stratified by the number of cross-ancestry causal SNPs (1, 3, or 5, corresponding to a total of 2, 6, and 10 causal SNPs, respectively) and per-causal SNP heritability ($h^2 = 10^{-4}$ or 2×10^{-4}; rows). **(a)** Precision-recall curves with PIP_{WB} thresholds ranging from 0.01 to 1. Precision is defined as $1 - \text{FDR}$ and Recall as $\frac{\text{TP}}{\text{TP+FN}}$, where TP, FP, and FN represent true positives, false positives, and false negatives, respectively. The PIP threshold of 0.5 is marked with a circle. **(b)** Bar plots displaying power at controlled FDR levels of 0.01, 0.05, and 0.1 for each method.

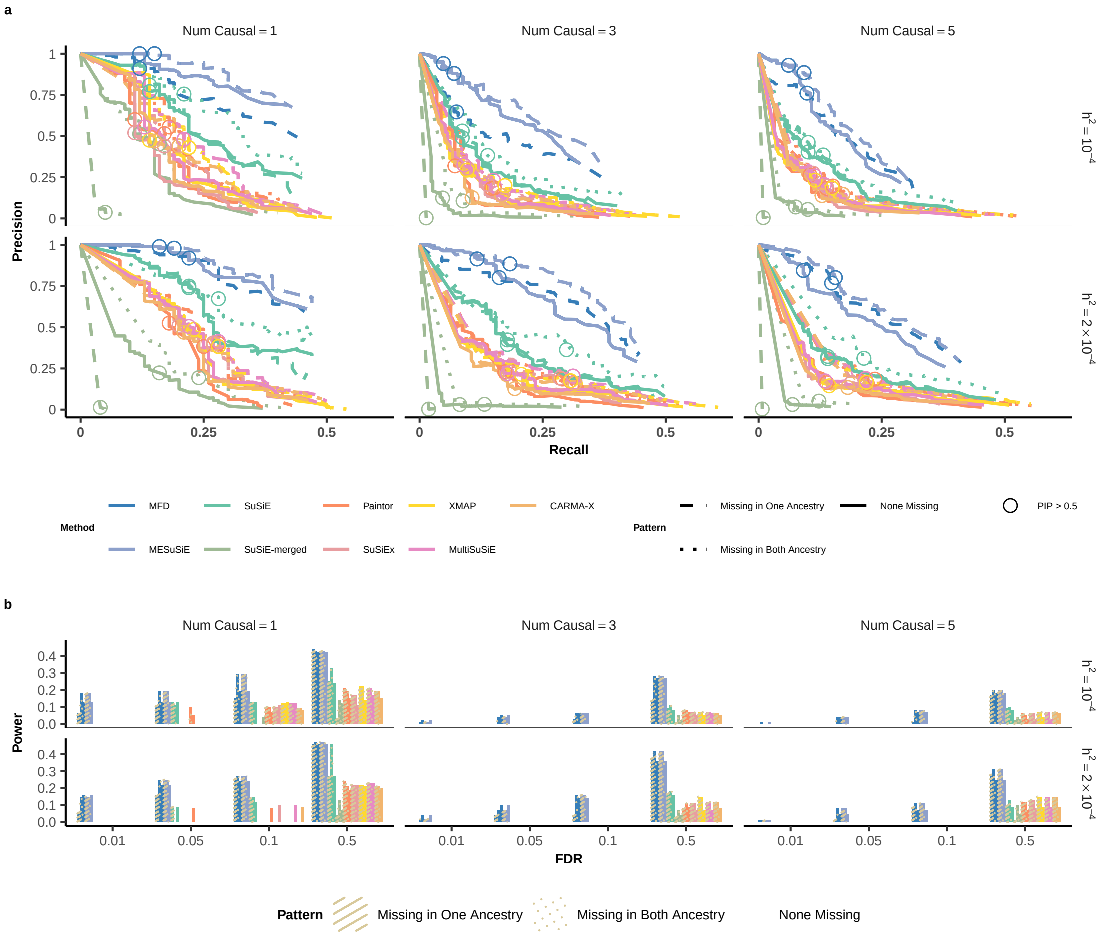

Supplementary Figure 31: **Performance metrics for Black British (BB) contextual causal signals (Missing non-causal variants from GWAS summary statistics and LD reference panel).** Comparison of method performance in detecting causal signals with non-zero effects in BB only with non-causal ancestry-specific variants from GWAS summary statistics and LD reference panel in one (stripe) or both (circle) ancestries. Results are stratified by the number of cross-ancestry causal SNPs (1, 3, or 5, corresponding to a total of 2, 6, and 10 causal SNPs, respectively) and per-causal SNP heritability ($h^2 = 10^{-4}$ or 2×10^{-4}; rows). **(a)** Precision-recall curves with PIP_{BB} thresholds ranging from 0.01 to 1. Precision is defined as $1 - \text{FDR}$ and Recall as $\frac{\text{TP}}{\text{TP+FN}}$, where TP, FP, and FN represent true positives, false positives, and false negatives, respectively. The PIP threshold of 0.5 is marked with a circle. **(b)** Bar plots displaying power at controlled FDR levels of 0.01, 0.05, and 0.1 for each method.

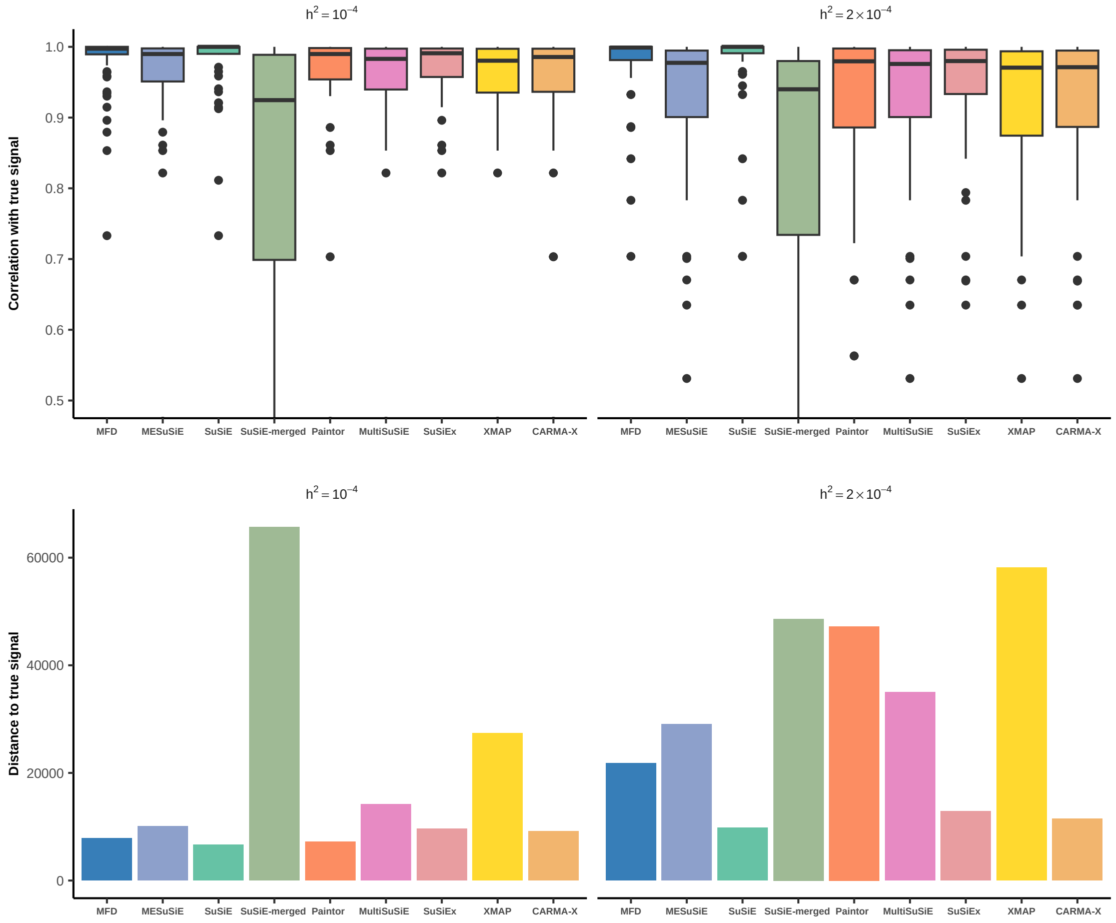

Supplementary Figure 32: **Performance evaluation with ancestry-specific segregating variant (AS-SV) present in one ancestry from GWAS summary statistics and LD reference panel.** Comparison of the correlation and genomic distance between the detected signal and the true causal signal when ancestry-specific segregating variant (AS-SV) are present from both GWAS and LD reference panel in one ancestry. Results are summarized across $n = 100$ independent regions of interest, stratified by per-causal SNP heritability ($h^2 = 10^{-4}$ or 2×10^{-4}). **(a)** Boxplots displaying the correlation (LD r^2) of the detected signal with the AS-SV for all evaluated methods. **(b)** Bar plots displaying the mean genomic distance (in base pairs) from the detected signal to AS-SV for all methods.

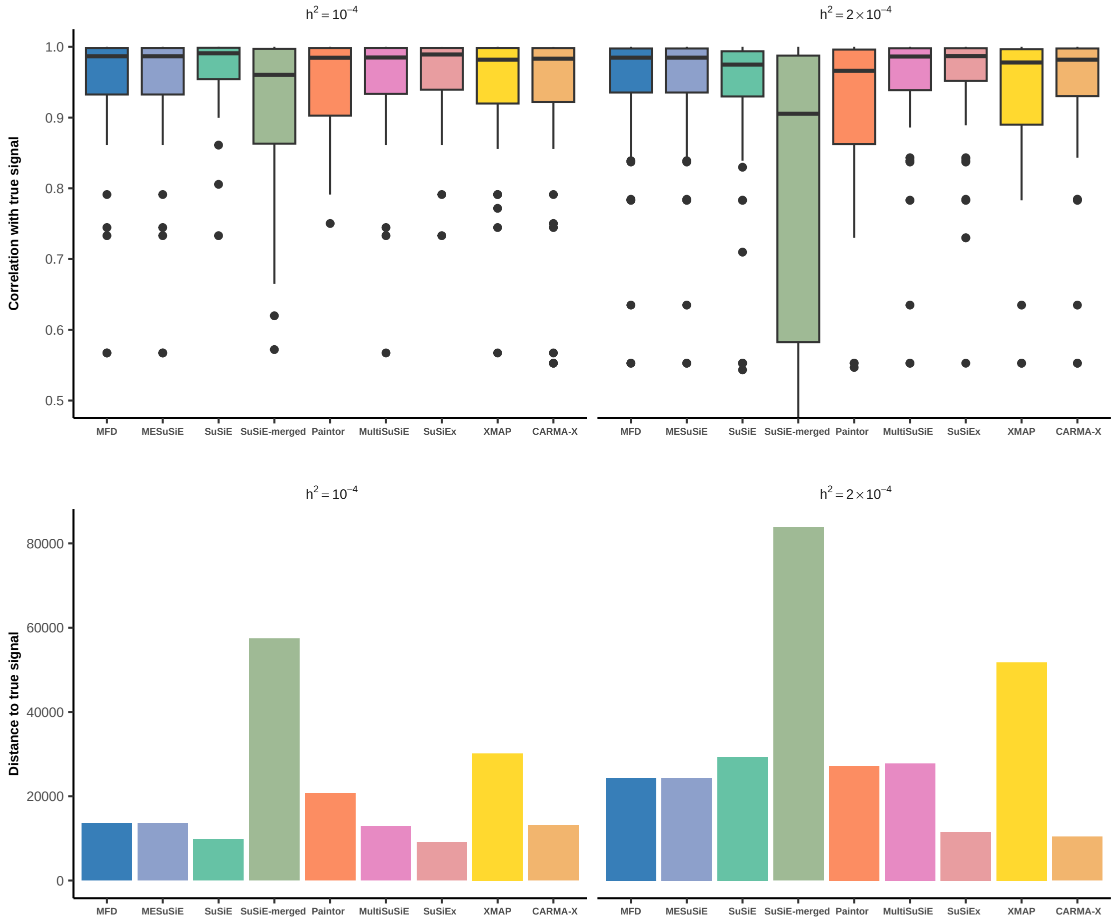

Supplementary Figure 33: **Performance evaluation with ancestry-specific segregating variant (AS-SV) masked in both ancestries from GWAS summary statistics and LD reference panel.** Comparison of the correlation and genomic distance between the detected signal and the true causal signal when ancestry-specific segregating variant (AS-SV) are masked from both GWAS and LD reference panel in both ancestries. Results are summarized across $n = 100$ independent regions of interest, stratified by per-causal SNP heritability ($h^2 = 10^{-4}$ or 2×10^{-4}). **(a)** Boxplots displaying the correlation (LD r^2) of the detected signal with the AS-SV for all evaluated methods. **(b)** Bar plots displaying the mean genomic distance (in base pairs) from the detected signal to the AS-SV for all methods.

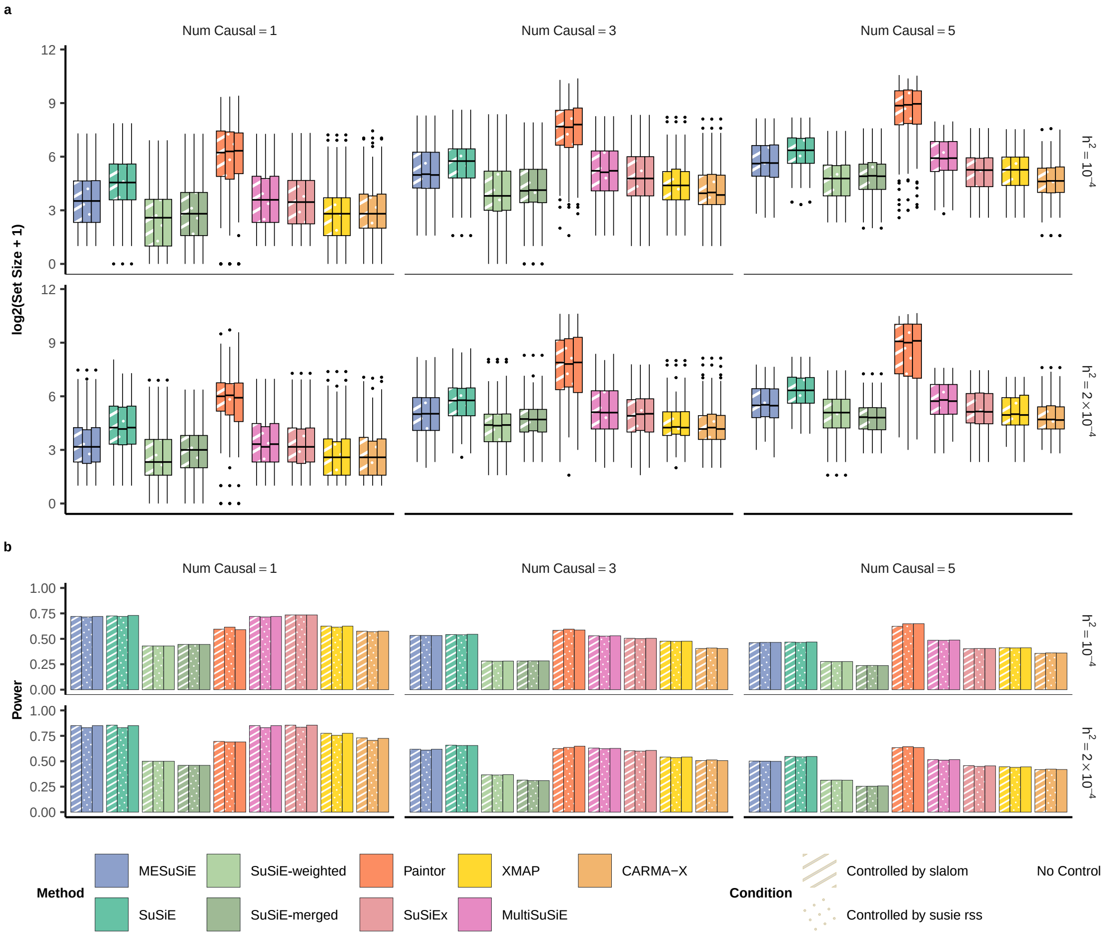

Supplementary Figure 34: **Performance metrics based on credible sets (LD Mismatch with External LD Reference Panel).** Comparison of 95% credible set sizes and power in detecting causal signals with non-zero effects in at least one ancestry. Results are summarized across $n = 100$ independent regions, stratified by the number of causal SNPs (1, 3, or 5 corresponding to a total of 2, 6, and 10 causal SNPs, respectively) and per-causal SNP heritability ($h^2 = 10^{-4}$ or 2×10^{-4}; rows). **(a)** Boxplots displaying the $\log_2$(set size + 1) of the 95% credible sets. **(b)** Bar plots displaying the power of the constructed 95% credible sets. Comparison includes methods with different strategies for handling LD mismatch: bars filled with **diagonal stripes** indicate methods controlled by **Slalom**, bars filled with **dots** indicate methods controlled by **SuSiE-RSS**, and **solid** bars indicate methods with no additional control.

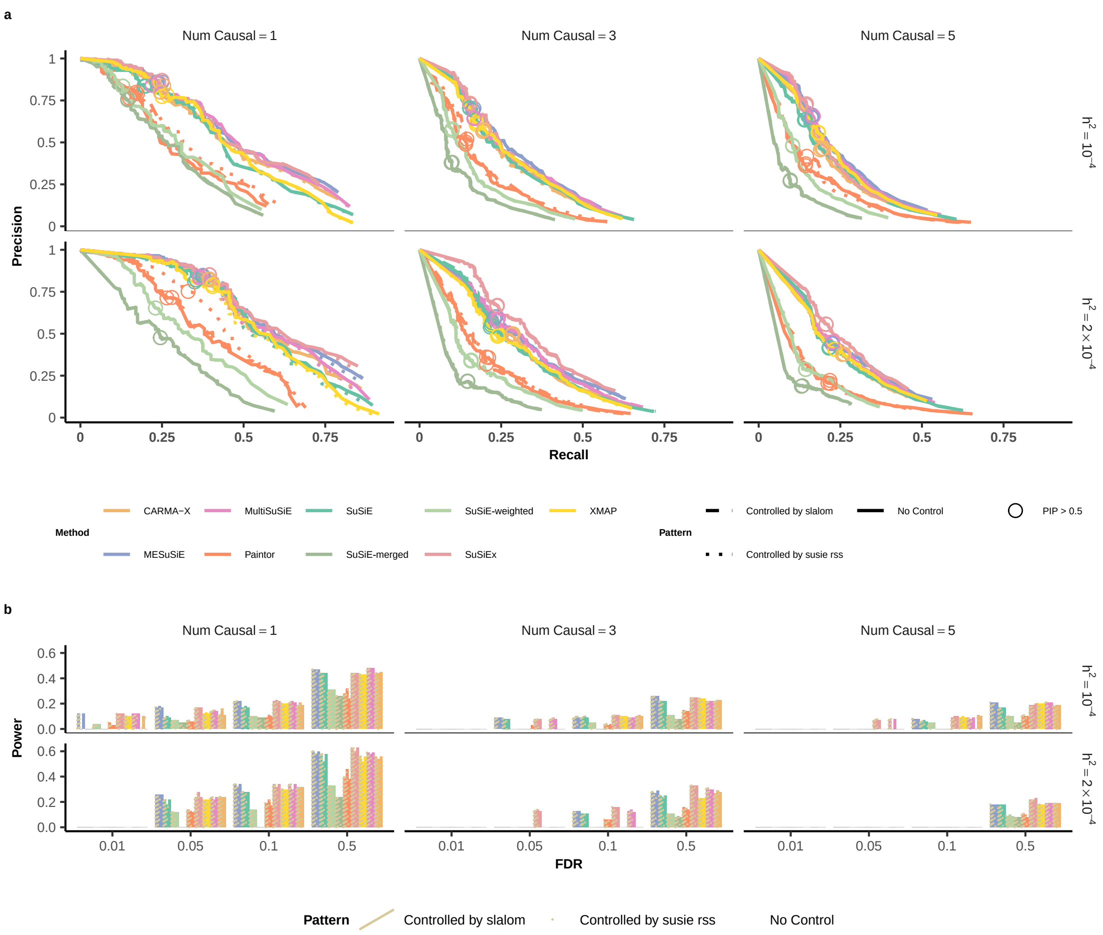

Supplementary Figure 35: **Comparison of method performance in detecting causal signals with non-zero effects in at least one ancestry (LD Mismatch with External LD Reference Panel, 'Either').** Results are stratified by the number of cross-ancestry causal SNPs (1, 3, or 5 corresponding to a total of 2, 6, and 10 causal SNPs, respectively) and per-causal SNP heritability ($h^2 = 10^{-4}$ or 2×10^{-4}; rows). **(a)** Precision-recall curves with PIP_{either} thresholds ranging from 0.01 to 1. Precision is defined as $1 - \text{FDR}$ and Recall as $\frac{\text{TP}}{\text{TP+FN}}$, where TP, FP, and FN represent true positives, false positives, and false negatives, respectively. The PIP threshold of 0.5 is marked with a circle. **(b)** Bar plots displaying power at controlled FDR levels of 0.01, 0.05, and 0.1. Bars filled with **diagonal stripes** indicate methods controlled by **Slalom**, bars filled with **dots** indicate methods controlled by **SuSiE-RSS**, and **solid** bars indicate no additional control.

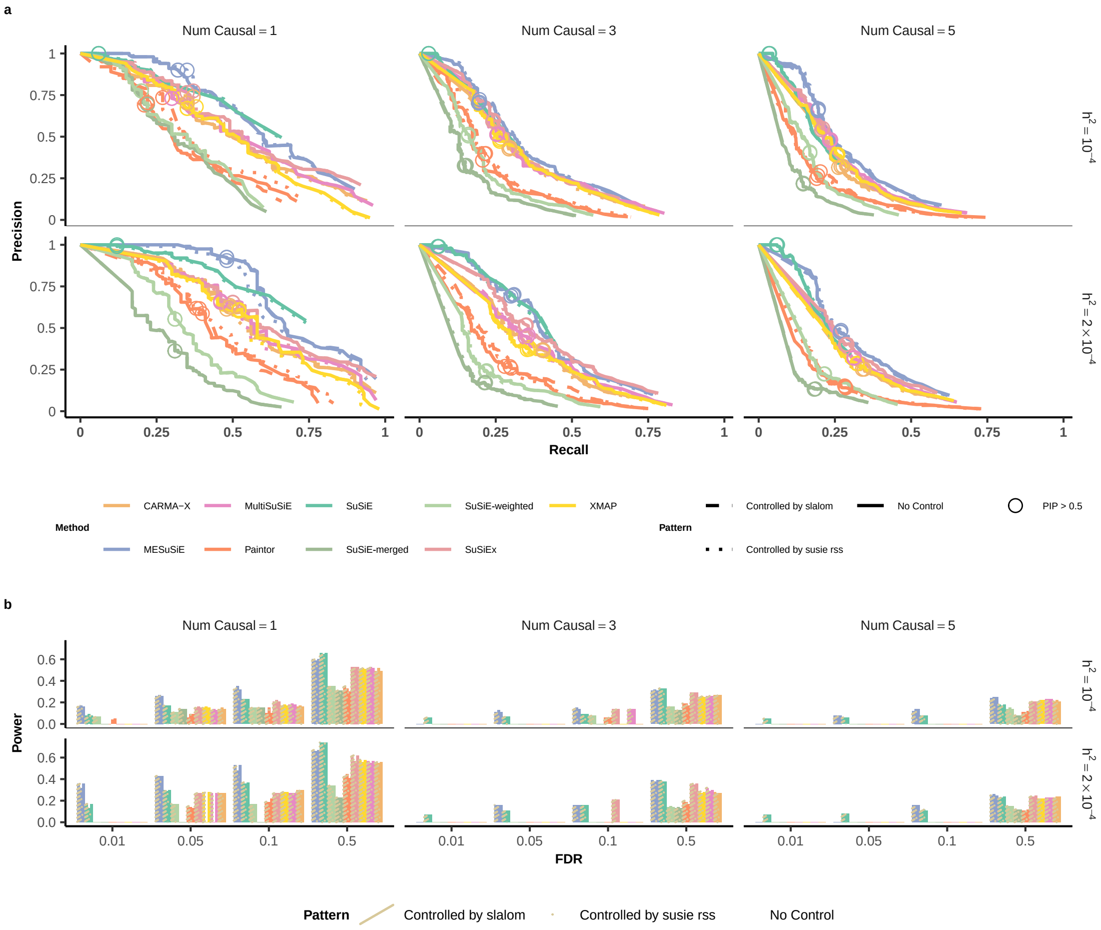

Supplementary Figure 36: **Comparison of method performance in detecting causal signals with non-zero effects in both ancestries (LD Mismatch with External LD Reference Panel, 'Cross-ancestry').** Results are stratified by the number of cross-ancestry causal SNPs (1, 3, or 5 corresponding to a total of 2, 6, and 10 causal SNPs, respectively) and per-causal SNP heritability ($h^2 = 10^{-4}$ or 2×10^{-4}; rows). **(a)** Precision-recall curves with PIP_{shared} thresholds ranging from 0.01 to 1. Precision is defined as $1 - \text{FDR}$ and Recall as $\frac{\text{TP}}{\text{TP+FN}}$, where TP, FP, and FN represent true positives, false positives, and false negatives, respectively. The PIP threshold of 0.5 is marked with a circle. **(b)** Bar plots displaying power at controlled FDR levels of 0.01, 0.05, and 0.1. Bars filled with **diagonal stripes** indicate methods controlled by **Slalom**, bars filled with **dots** indicate methods controlled by **SuSiE-RSS**, and **solid** bars indicate no additional control.

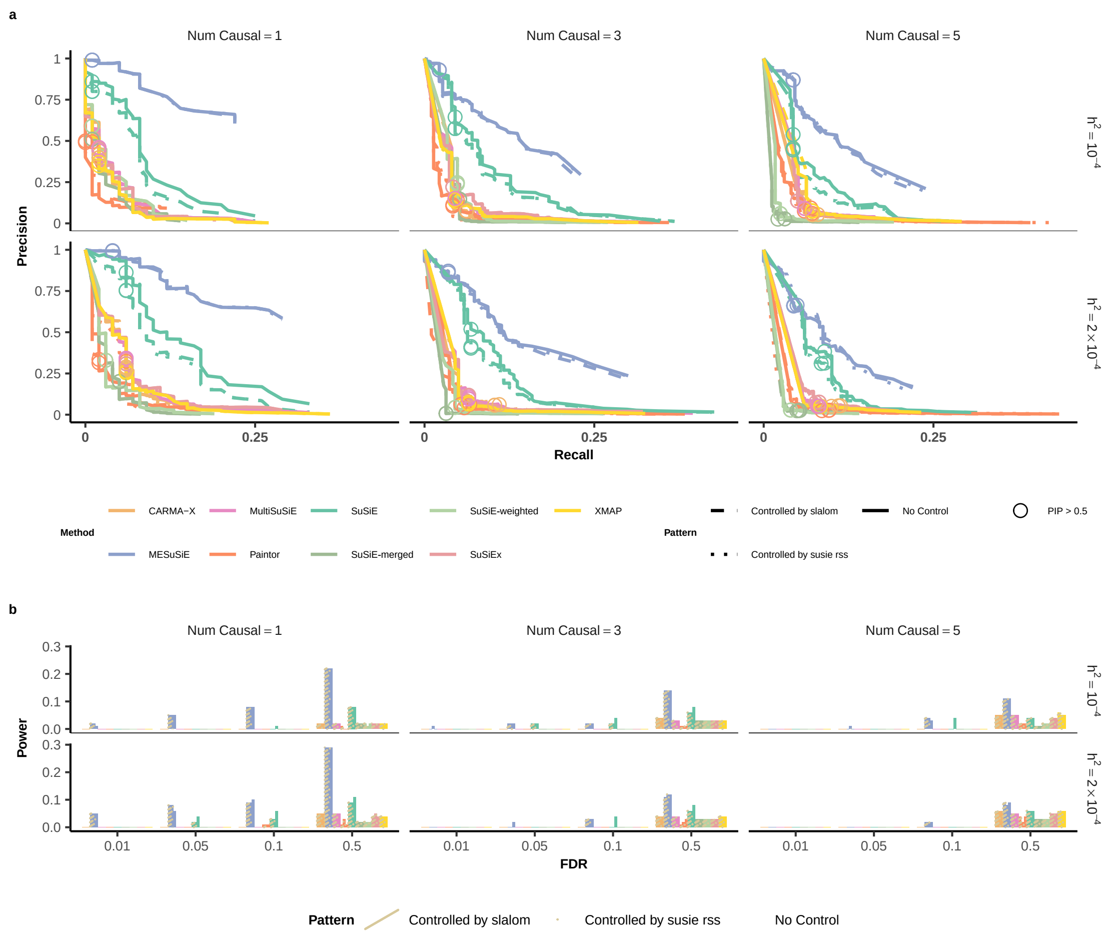

Supplementary Figure 37: **Performance metrics for White British (WB) contextual causal signals (LD Mismatch with External LD Reference Panel).** Comparison of method performance in detecting causal signals with non-zero effects in WB only. Results are stratified by the number of cross-ancestry causal SNPs (1, 3, or 5 corresponding to a total of 2, 6, and 10 causal SNPs, respectively) and per-causal SNP heritability ($h^2 = 10^{-4}$ or 2×10^{-4}; rows). **(a)** Precision-recall curves with PIP_{WB} thresholds ranging from 0.01 to 1. Precision is defined as $1 - \text{FDR}$ and Recall as $\frac{\text{TP}}{\text{TP+FN}}$, where TP, FP, and FN represent true positives, false positives, and false negatives, respectively. The PIP threshold of 0.5 is marked with a circle. **(b)** Bar plots displaying power at controlled FDR levels of 0.01, 0.05, and 0.1. Bars filled with **diagonal stripes** indicate methods controlled by **Slalom**, bars filled with **dots** indicate methods controlled by **SuSiE-RSS**, and **solid** bars indicate no additional control.

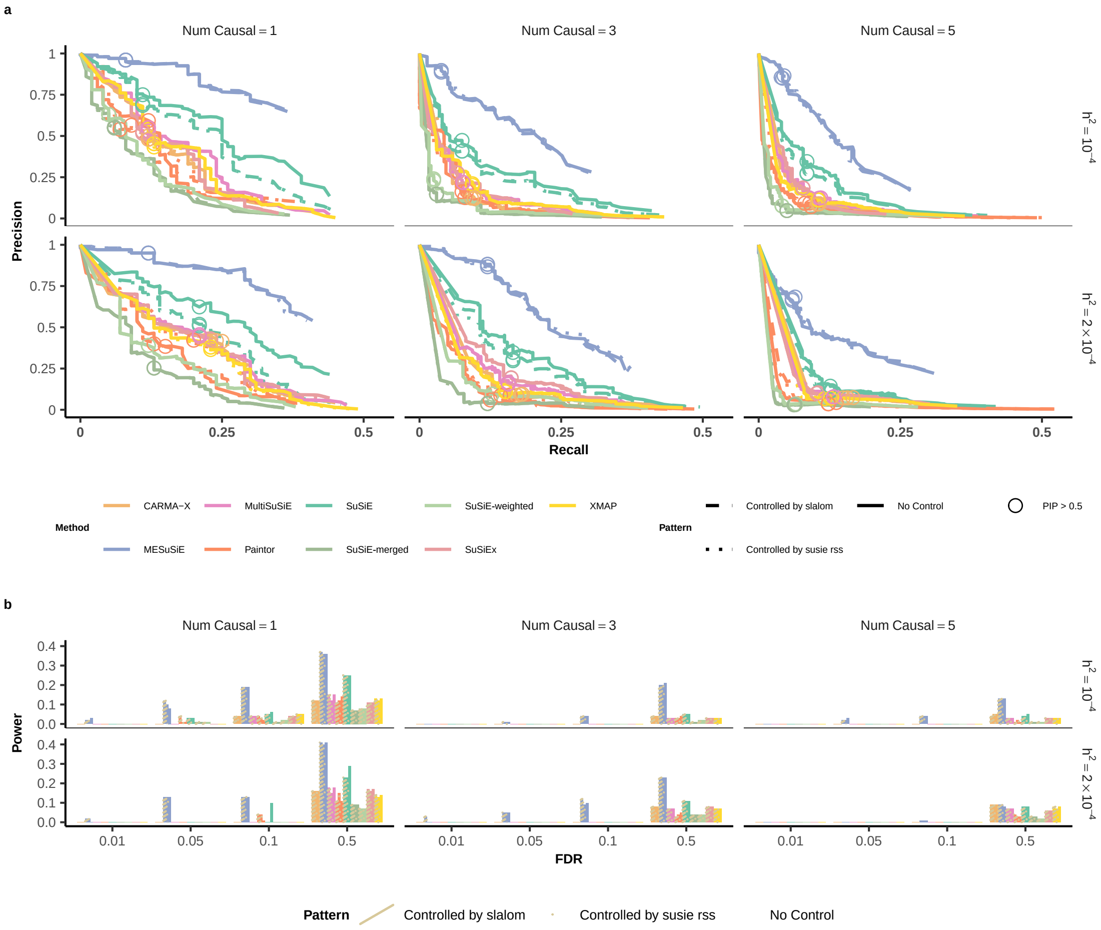

Supplementary Figure 38: **Performance metrics for Black British (BB) contextual causal signals (LD Mismatch with External LD Reference Panel).** Comparison of method performance in detecting causal signals with non-zero effects in BB only. Results are stratified by the number of cross-ancestry causal SNPs (1, 3, or 5 corresponding to a total of 2, 6, and 10 causal SNPs, respectively) and per-causal SNP heritability ($h^2 = 10^{-4}$ or 2×10^{-4}; rows). **(a)** Precision-recall curves with PIP_{BB} thresholds ranging from 0.01 to 1. Precision is defined as $1 - \text{FDR}$ and Recall as $\frac{\text{TP}}{\text{TP+FN}}$, where TP, FP, and FN represent true positives, false positives, and false negatives, respectively. The PIP threshold of 0.5 is marked with a circle. **(b)** Bar plots displaying power at controlled FDR levels of 0.01, 0.05, and 0.1. Bars filled with **diagonal stripes** indicate methods controlled by **Slalom**, bars filled with **dots** indicate methods controlled by **SuSiE-RSS**, and **solid** bars indicate no additional control.

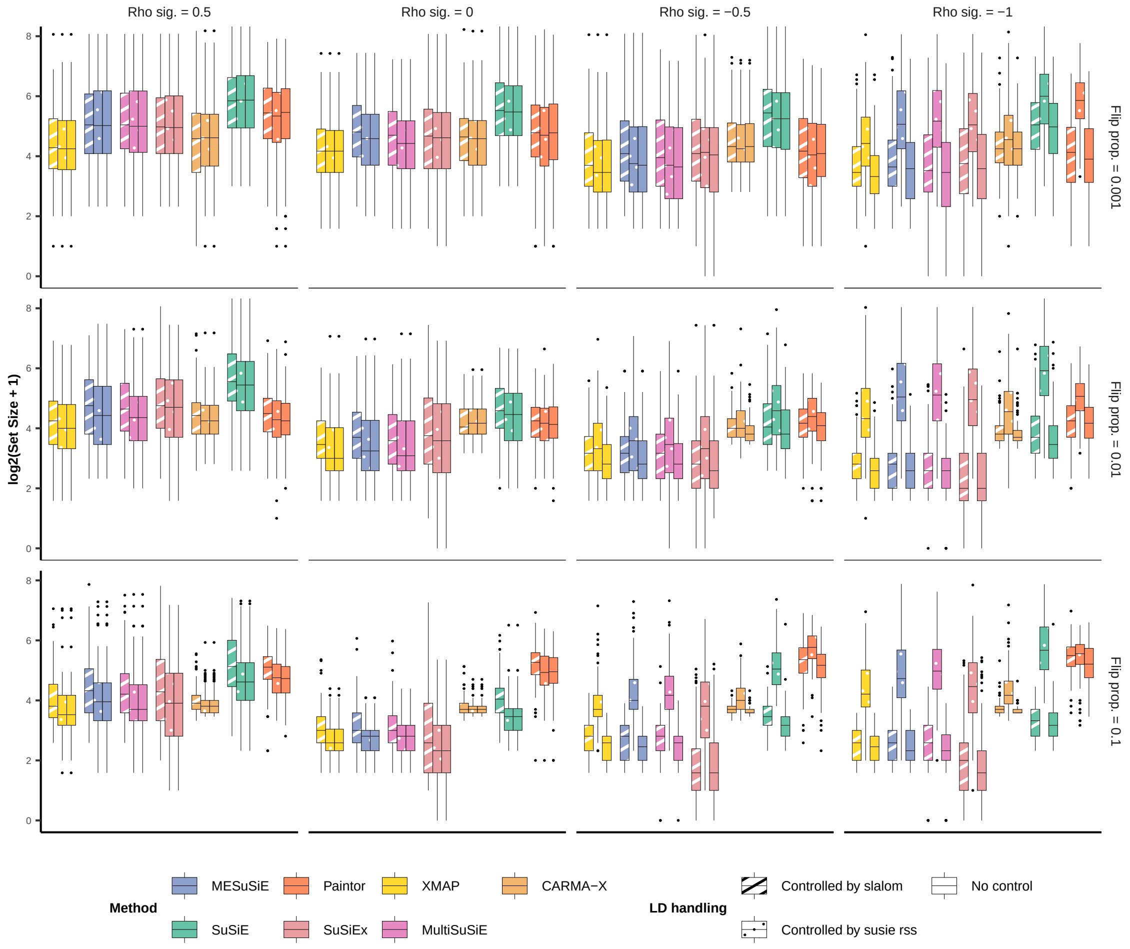

Supplementary Figure 39: **Impact of systematic LD mismatch on credible set sizes (LD Mismatch with Systematic Mismatch).** Distribution of 95% credible set sizes (transformed as $\log_2(\text{Set Size} + 1)$) across $n = 100$ independent genomic regions. Panels are stratified by the proportion of variants with induced mismatch (rows: 0.001, 0.01, 0.1) and the scale of the mismatch factor (columns: ρ_{sig} = 0.5, 0, -0.5, -1). To denote LD handling strategies, boxplots are filled with **diagonal stripes** for methods controlled by **Slalom**, **dots** for methods controlled by **SuSiE-RSS**, and **solid colors** for methods with no additional control.

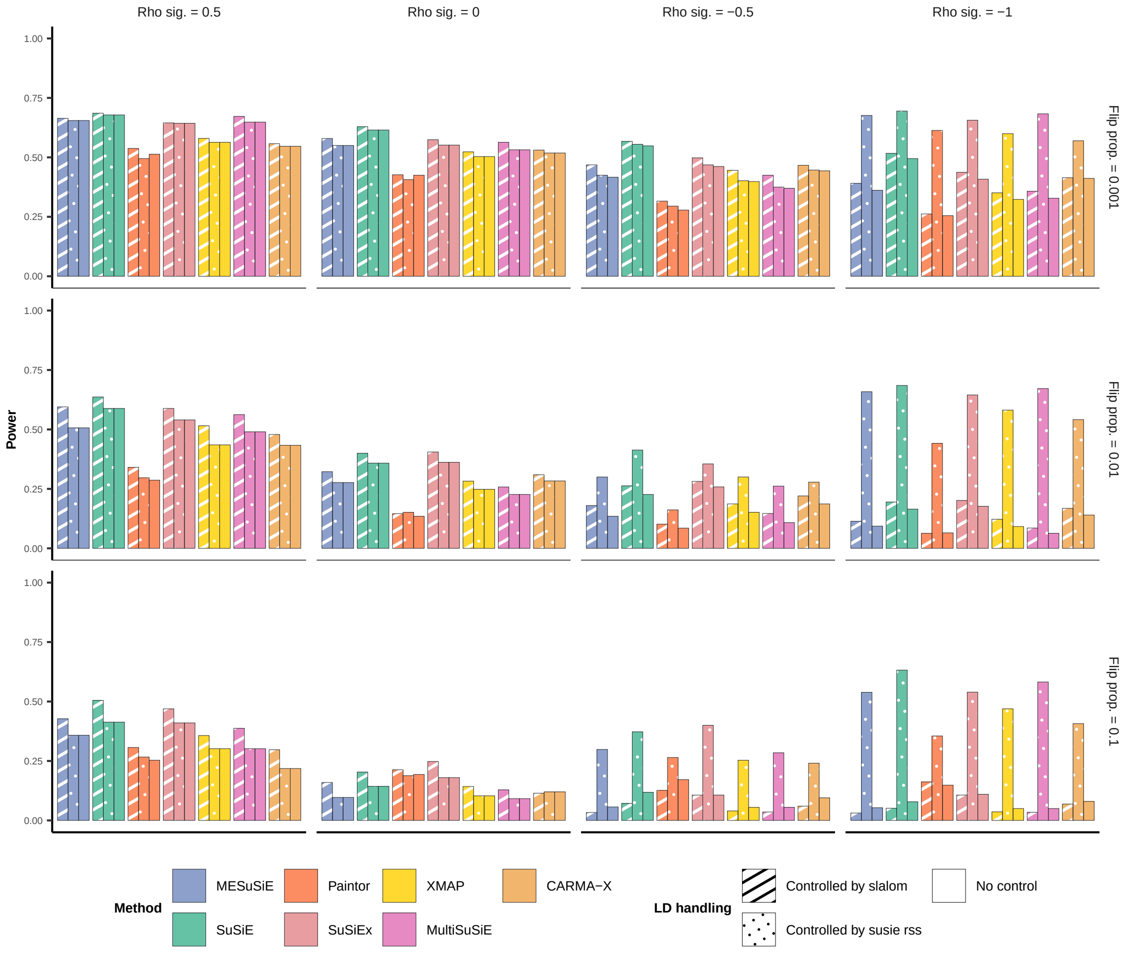

Supplementary Figure 40: **Impact of systematic LD mismatch on power achieved by credible set (LD Mismatch with Systematic Mismatch).** Distribution of power achieved by 95% credible set across $n = 100$ independent genomic regions. Panels are stratified by the proportion of variants with induced mismatch (rows: 0.001, 0.01, 0.1) and the scale of the mismatch factor (columns: $\rho_{sig} = 0.5$, 0, -0.5, -1). To denote LD handling strategies, boxplots are filled with **diagonal stripes** for methods controlled by **Slalom**, **dots** for methods controlled by **SuSiE-RSS**, and **solid colors** for methods with no additional control.

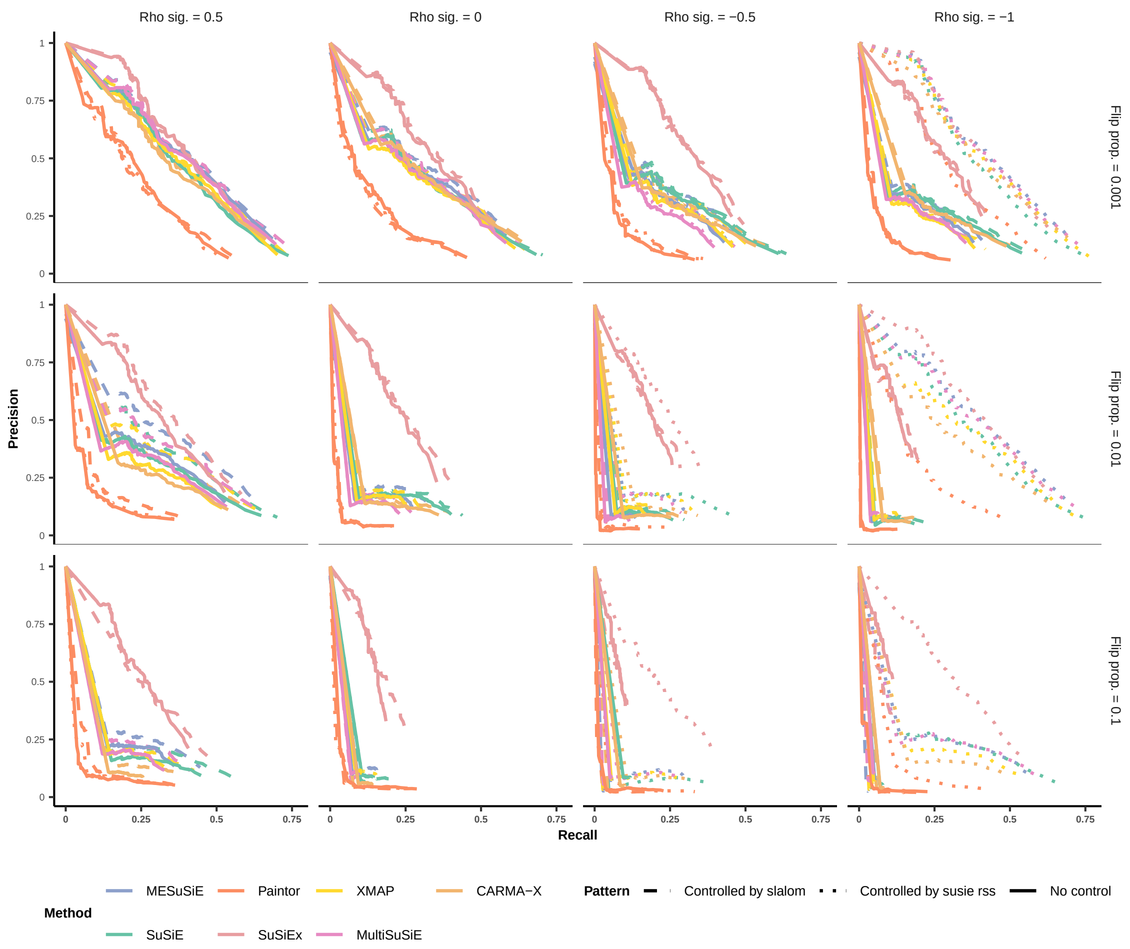

Supplementary Figure 41: **Comparison of method performance in detecting causal signals with non-zero effects in at least one ancestry (LD Mismatch with Systematic Mismatch, 'Either').** Precision-recall curves with PIP_{either} thresholds ranging from 0.01 to 1. Precision is defined as $1 - \text{FDR}$ and Recall as $\frac{\text{TP}}{\text{TP+FN}}$, where TP, FP, and FN represent true positives, false positives, and false negatives, respectively. The PIP threshold of 0.5 is marked with a circle. Panels are stratified by the proportion of variants with induced mismatch (rows: 0.001, 0.01, 0.1) and the scale of the mismatch factor (columns: ρ_{sig} = 0.5, 0, -0.5, -1). To denote LD handling strategies, boxplots are filled with **diagonal stripes** for methods controlled by **Slalom**, **dots** for methods controlled by **SuSiE-RSS**, and **solid colors** for methods with no additional control.

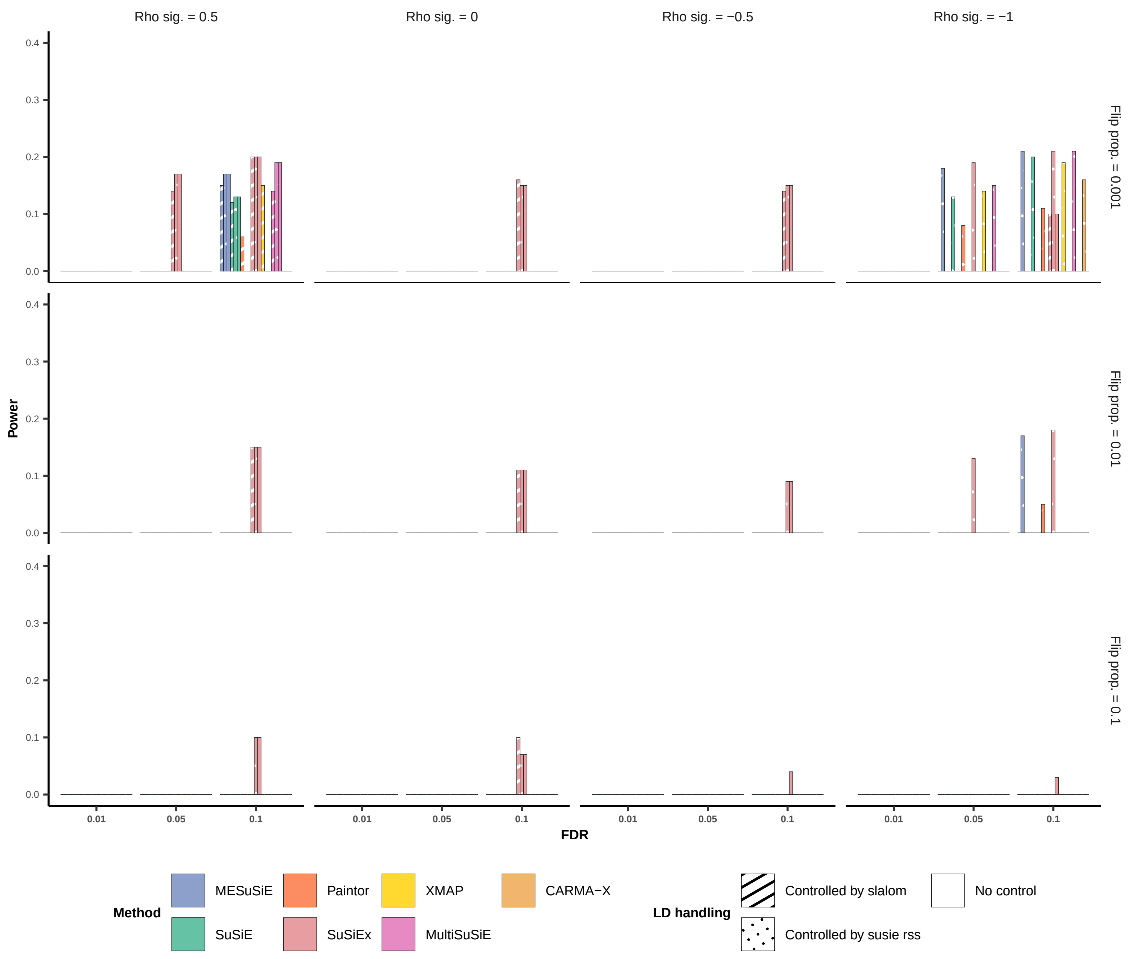

Supplementary Figure 42: **Comparison of method performance in detecting causal signals with non-zero effects in at least one ancestry (LD Mismatch with Systematic Mismatch, 'Either').** Bar plots displaying power at controlled FDR levels of 0.01, 0.05, and 0.1. The PIP threshold of 0.5 is marked with a circle. Panels are stratified by the proportion of variants with induced mismatch (rows: 0.001, 0.01, 0.1) and the scale of the mismatch factor (columns: ρ_{sig} = 0.5, 0, -0.5, -1). To denote LD handling strategies, boxplots are filled with **diagonal stripes** for methods controlled by **Slalom**, **dots** for methods controlled by **SuSiE-RSS**, and **solid colors** for methods with no additional control.

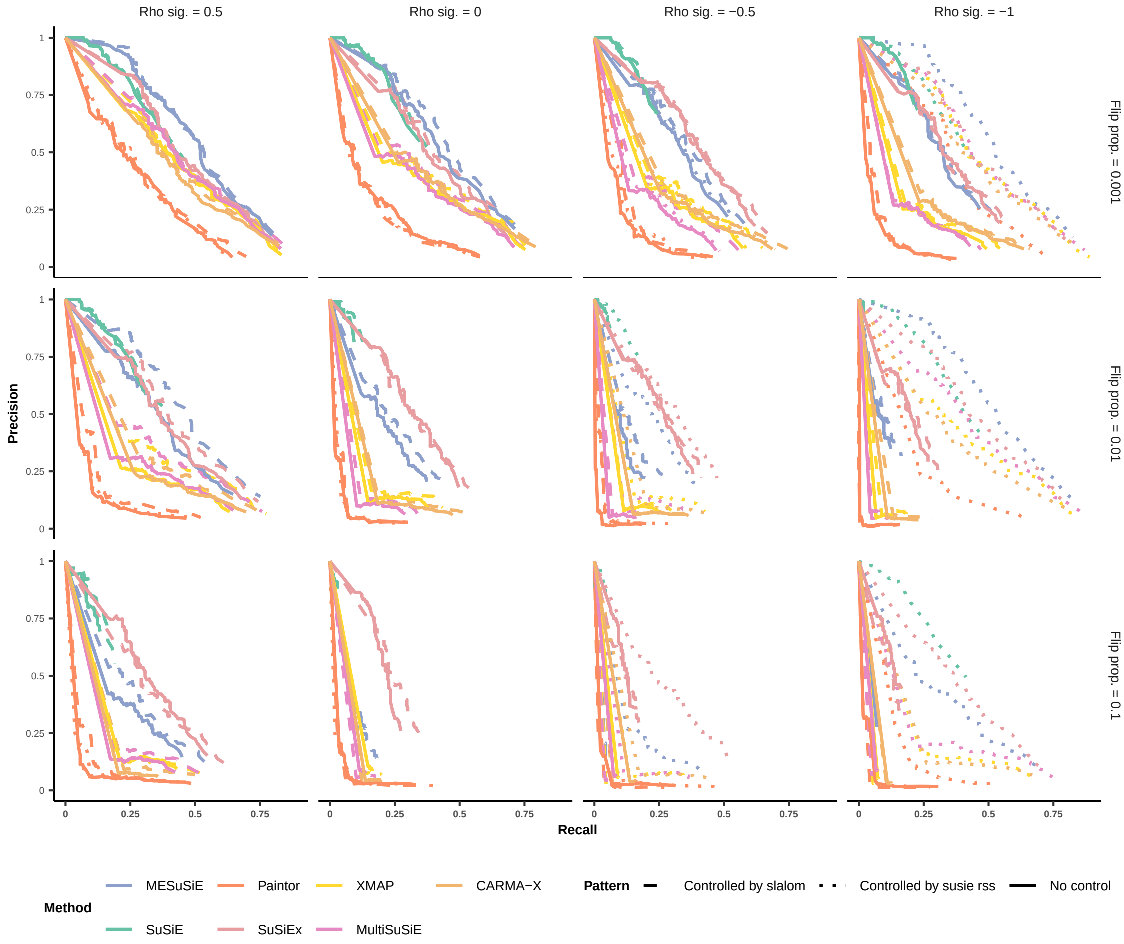

Supplementary Figure 43: **Comparison of method performance in detecting causal signals with non-zero effects in both ancestries (LD Mismatch with Systematic Mismatch, 'Cross-ancestry').** Precision-recall curves with PIP_{shared} thresholds ranging from 0.01 to 1. Precision is defined as $1 - \text{FDR}$ and Recall as $\frac{\text{TP}}{\text{TP+FN}}$, where TP, FP, and FN represent true positives, false positives, and false negatives, respectively. The PIP threshold of 0.5 is marked with a circle. Panels are stratified by the proportion of variants with induced mismatch (rows: 0.001, 0.01, 0.1) and the scale of the mismatch factor (columns: ρ_{sig} = 0.5, 0, -0.5, -1). To denote LD handling strategies, boxplots are filled with **diagonal stripes** for methods controlled by **Slalom**, **dots** for methods controlled by **SuSiE-RSS**, and **solid colors** for methods with no additional control.

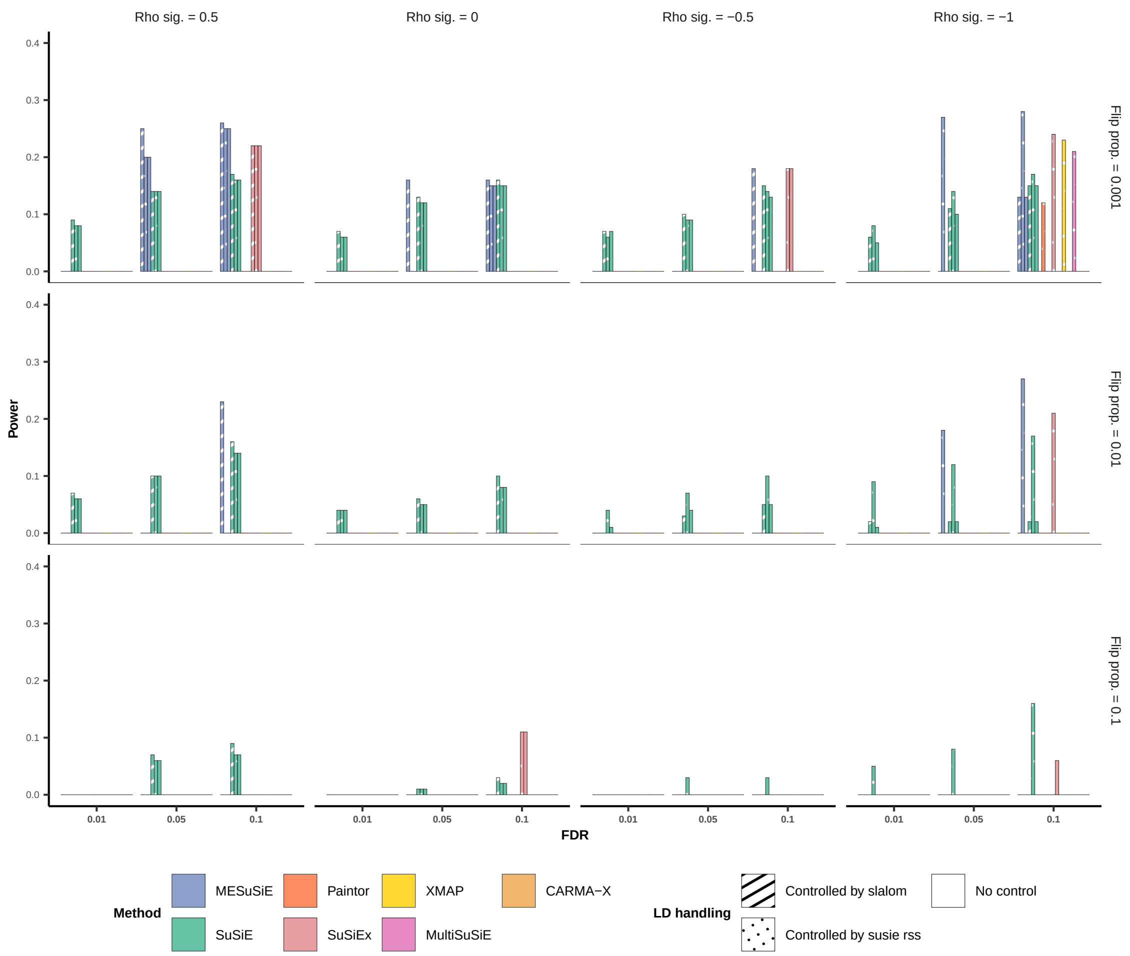

Supplementary Figure 44: **Comparison of method performance in detecting causal signals with non-zero effects in both ancestries (LD Mismatch with Systematic Mismatch, 'Cross-ancestry').** Bar plots displaying power at controlled FDR levels of 0.01, 0.05, and 0.1. The PIP threshold of 0.5 is marked with a circle. Panels are stratified by the proportion of variants with induced mismatch (rows: 0.001, 0.01, 0.1) and the scale of the mismatch factor (columns: ρ_{sig} = 0.5, 0, -0.5, -1). To denote LD handling strategies, boxplots are filled with **diagonal stripes** for methods controlled by **Slalom**, **dots** for methods controlled by **SuSiE-RSS**, and **solid colors** for methods with no additional control.

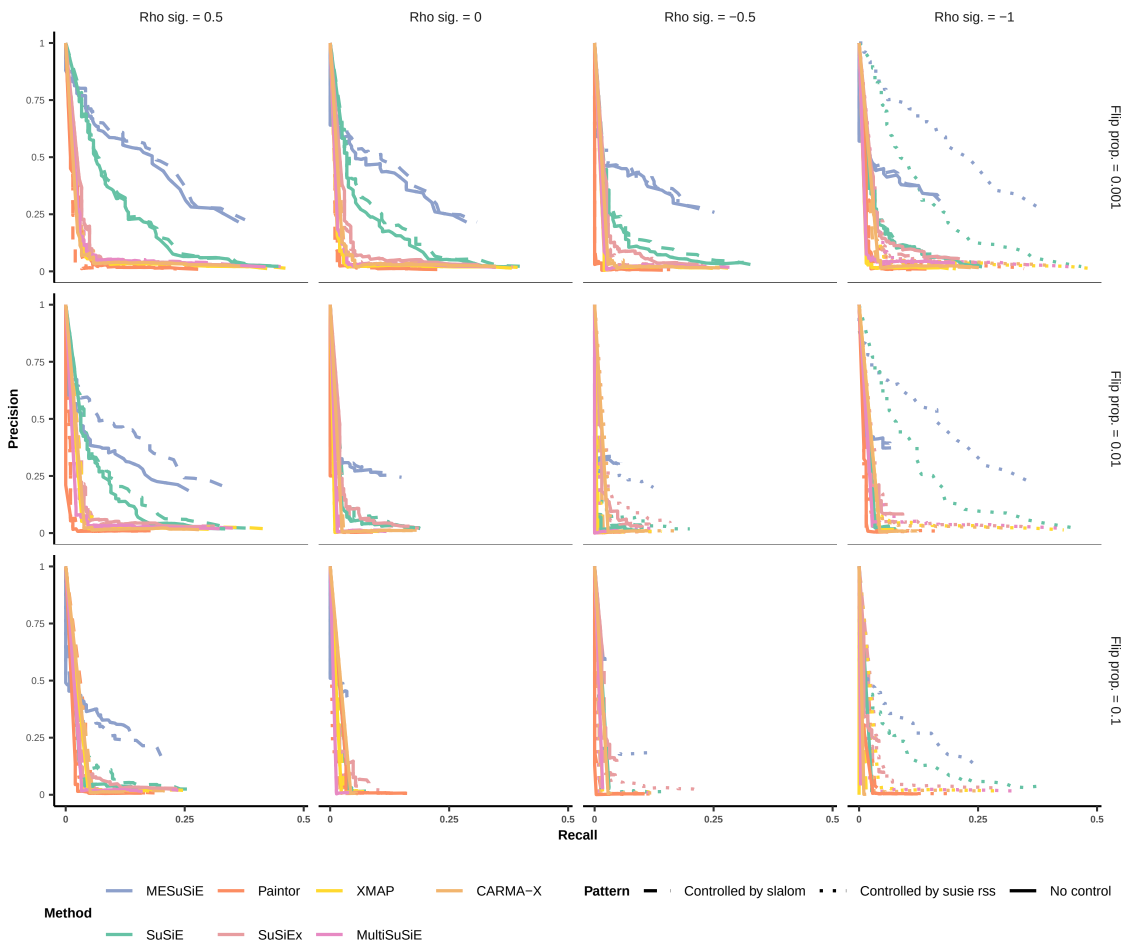

Supplementary Figure 45: **Performance metrics for White British (WB) contextual causal signals (LD Mismatch with Systematic Mismatch).** Comparison of method performance in detecting causal signals with non-zero effects in WB only. Precision-recall curves with PIP_{WB} thresholds ranging from 0.01 to 1. Precision is defined as $1 - \text{FDR}$ and Recall as $\frac{\text{TP}}{\text{TP+FN}}$, where TP, FP, and FN represent true positives, false positives, and false negatives, respectively. The PIP threshold of 0.5 is marked with a circle. Panels are stratified by the proportion of variants with induced mismatch (rows: 0.001, 0.01, 0.1) and the scale of the mismatch factor (columns: ρ_{sig} = 0.5, 0, -0.5, -1). To denote LD handling strategies, boxplots are filled with **diagonal stripes** for methods controlled by **Slalom**, **dots** for methods controlled by **SuSiE-RSS**, and **solid colors** for methods with no additional control.

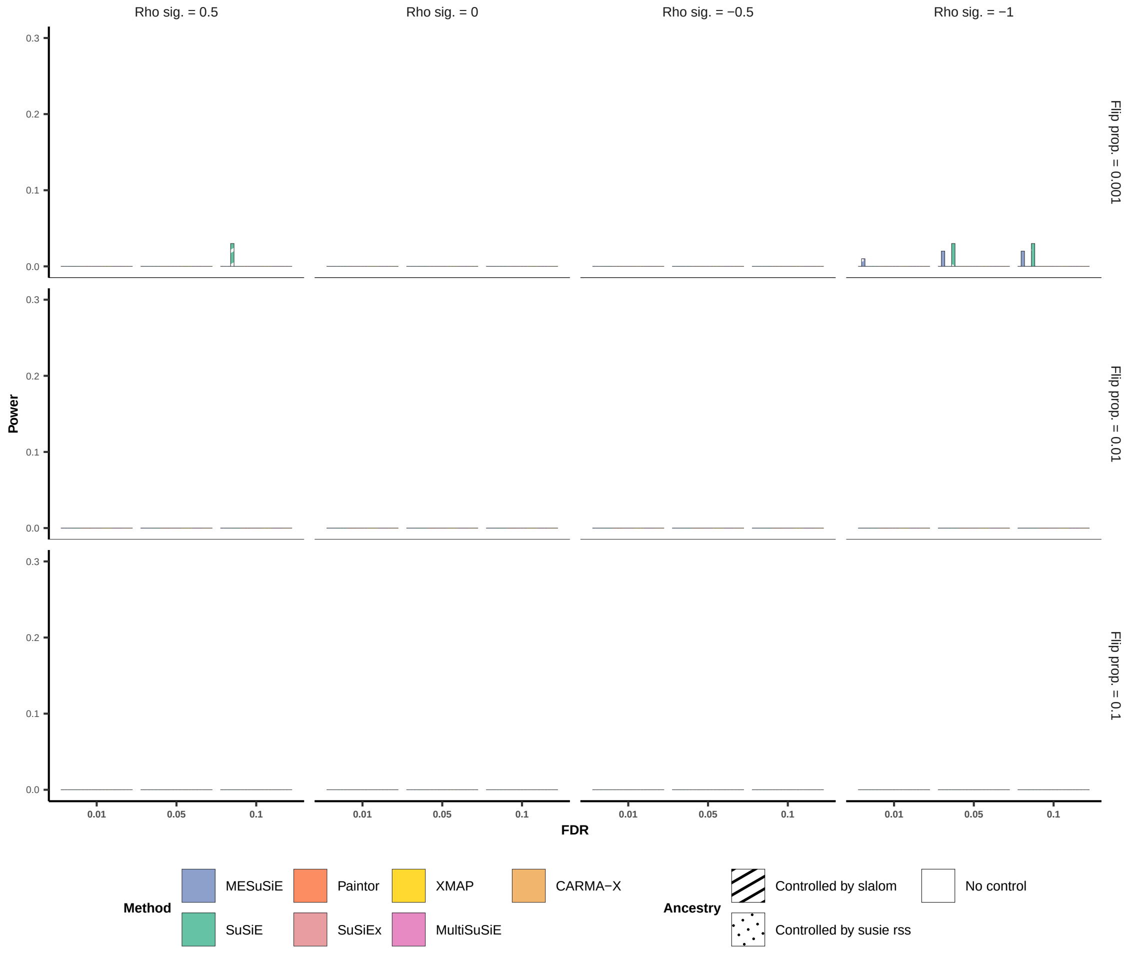

Supplementary Figure 46: **Performance metrics for White British (WB) contextual causal signals (LD Mismatch with Systematic Mismatch).** Comparison of method performance in detecting causal signals with non-zero effects in WB only. Bar plots displaying power at controlled FDR levels of 0.01, 0.05, and 0.1. The PIP threshold of 0.5 is marked with a circle. Panels are stratified by the proportion of variants with induced mismatch (rows: 0.001, 0.01, 0.1) and the scale of the mismatch factor (columns: ρ_{sig} = 0.5, 0, -0.5, -1). To denote LD handling strategies, boxplots are filled with **diagonal stripes** for methods controlled by **Slalom**, **dots** for methods controlled by **SuSiE-RSS**, and **solid colors** for methods with no additional control.

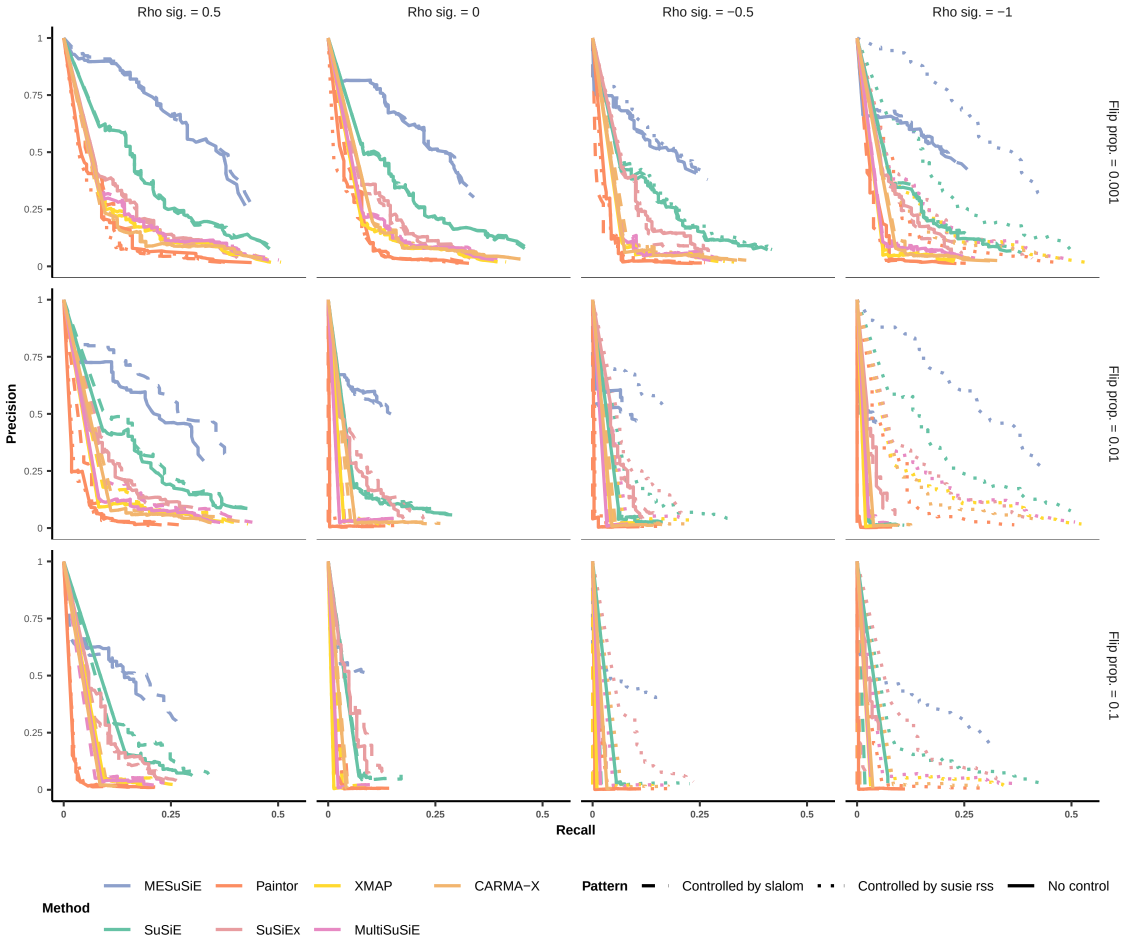

Supplementary Figure 47: **Performance metrics for Black British (BB) contextual causal signals (LD Mismatch with Systematic Mismatch).** Comparison of method performance in detecting causal signals with non-zero effects in BB only. Precision-recall curves with PIP_{WB} thresholds ranging from 0.01 to 1. Precision is defined as 1 − FDR and Recall as $\frac{\text{TP}}{\text{TP+FN}}$, where TP, FP, and FN represent true positives, false positives, and false negatives, respectively. The PIP threshold of 0.5 is marked with a circle. Panels are stratified by the proportion of variants with induced mismatch (rows: 0.001, 0.01, 0.1) and the scale of the mismatch factor (columns: ρ_{sig} = 0.5, 0, -0.5, -1). To denote LD handling strategies, boxplots are filled with **diagonal stripes** for methods controlled by **Slalom**, **dots** for methods controlled by **SuSiE-RSS**, and **solid colors** for methods with no additional control.

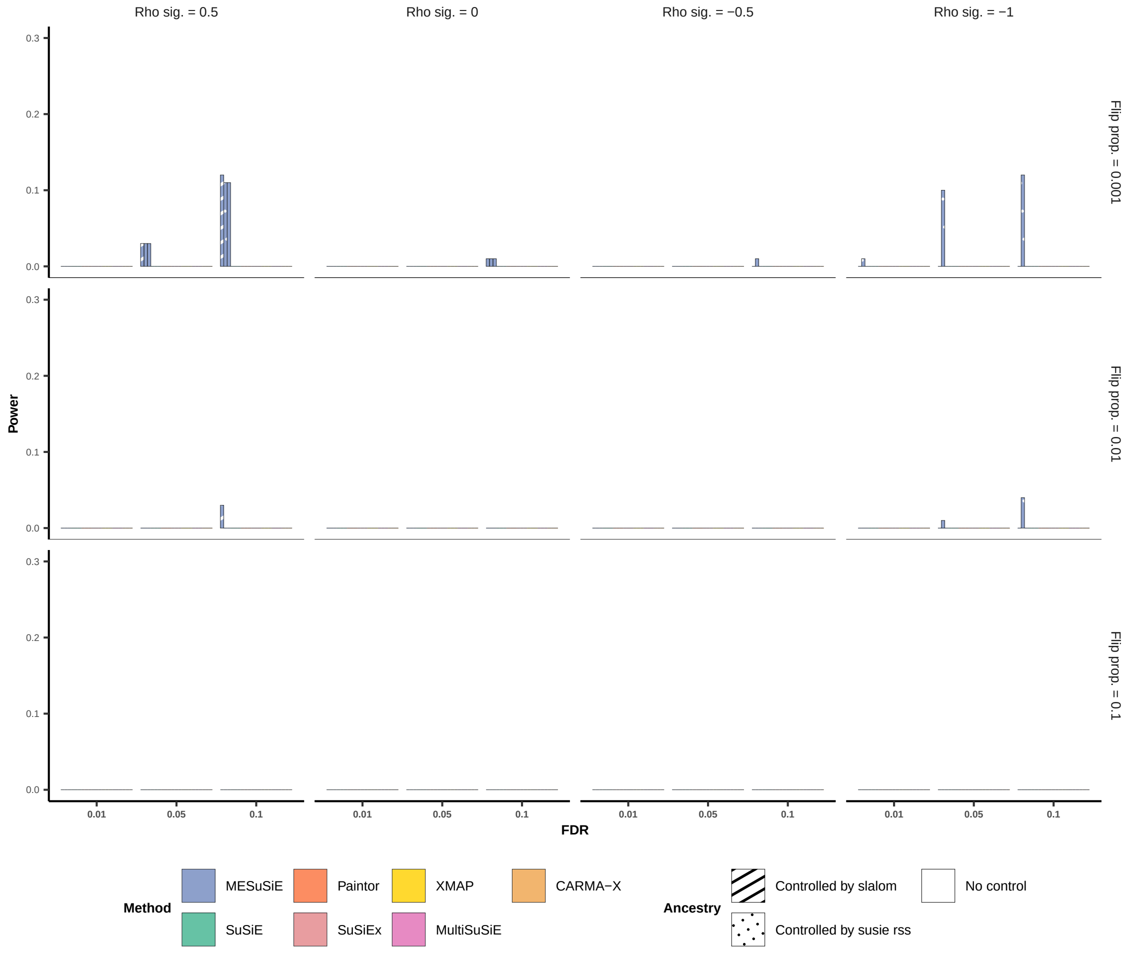

Supplementary Figure 48: **Performance metrics for Black British (BB) contextual causal signals (LD Mismatch with Systematic Mismatch).** Comparison of method performance in detecting causal signals with non-zero effects in BB only. Bar plots displaying power at controlled FDR levels of 0.01, 0.05, and 0.1. The PIP threshold of 0.5 is marked with a circle. Panels are stratified by the proportion of variants with induced mismatch (rows: 0.001, 0.01, 0.1) and the scale of the mismatch factor (columns: ρ_{sig} = 0.5, 0, -0.5, -1). To denote LD handling strategies, boxplots are filled with **diagonal stripes** for methods controlled by **Slalom**, **dots** for methods controlled by **SuSiE-RSS**, and **solid colors** for methods with no additional control.

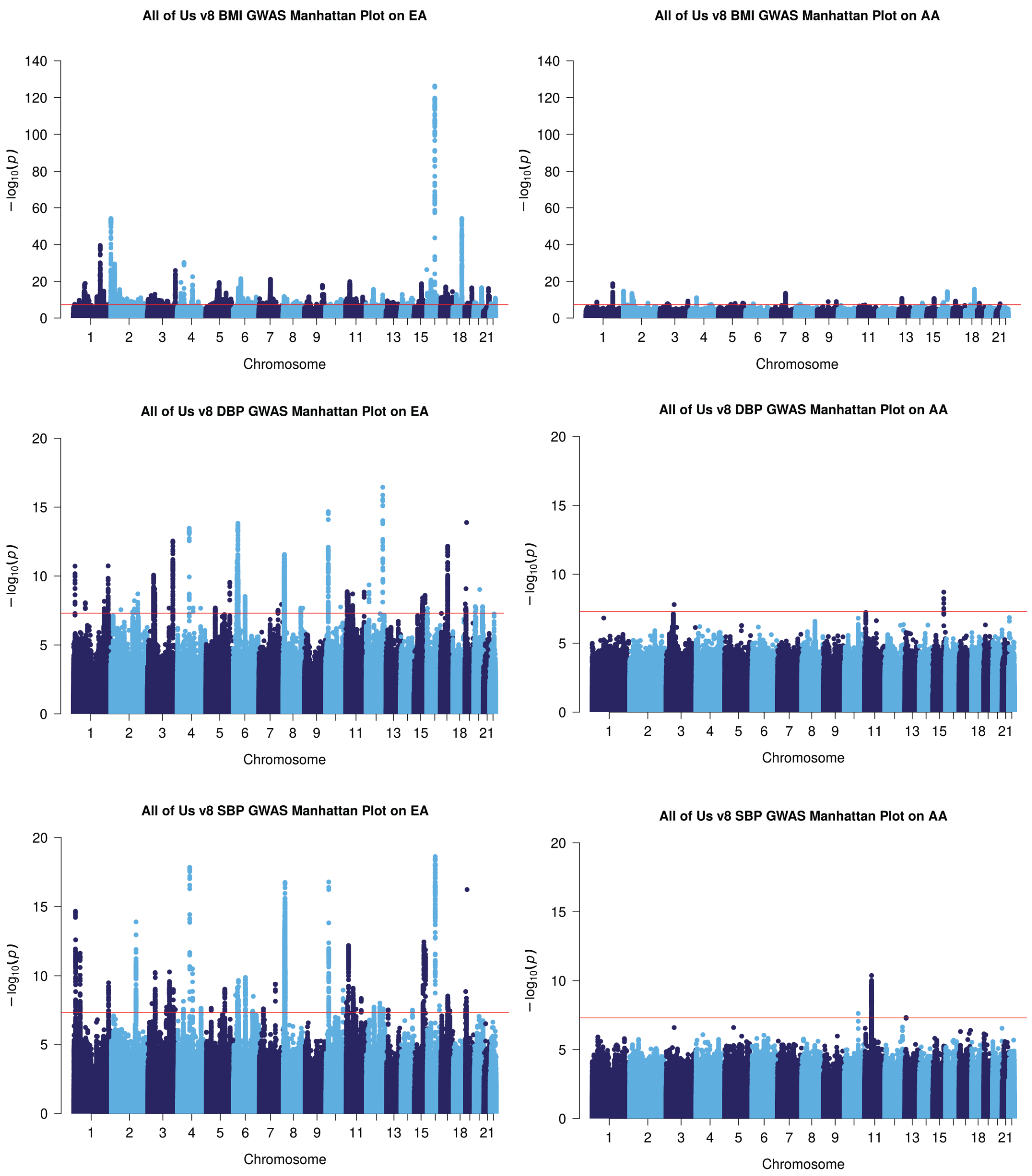

Supplementary Figure 49: **GWAS Manhattan plots for All of Us traits.** This figure displays summary statistics stratified by trait and genetic ancestry, with rows representing Body Mass Index (BMI, top), Diastolic Blood Pressure (DBP, middle), and Systolic Blood Pressure (SBP, bottom), and columns distinguishing between European Americans (EA, left) and African Americans (AA, right). In each panel, genetic variants are plotted according to their chromosomal position on the x-axis against their statistical significance on the y-axis, expressed as the negative base-10 logarithm of the P-value ($-\log_{10}(P)$), where the horizontal red line denotes the genome-wide significance threshold (5×10^{-8}).

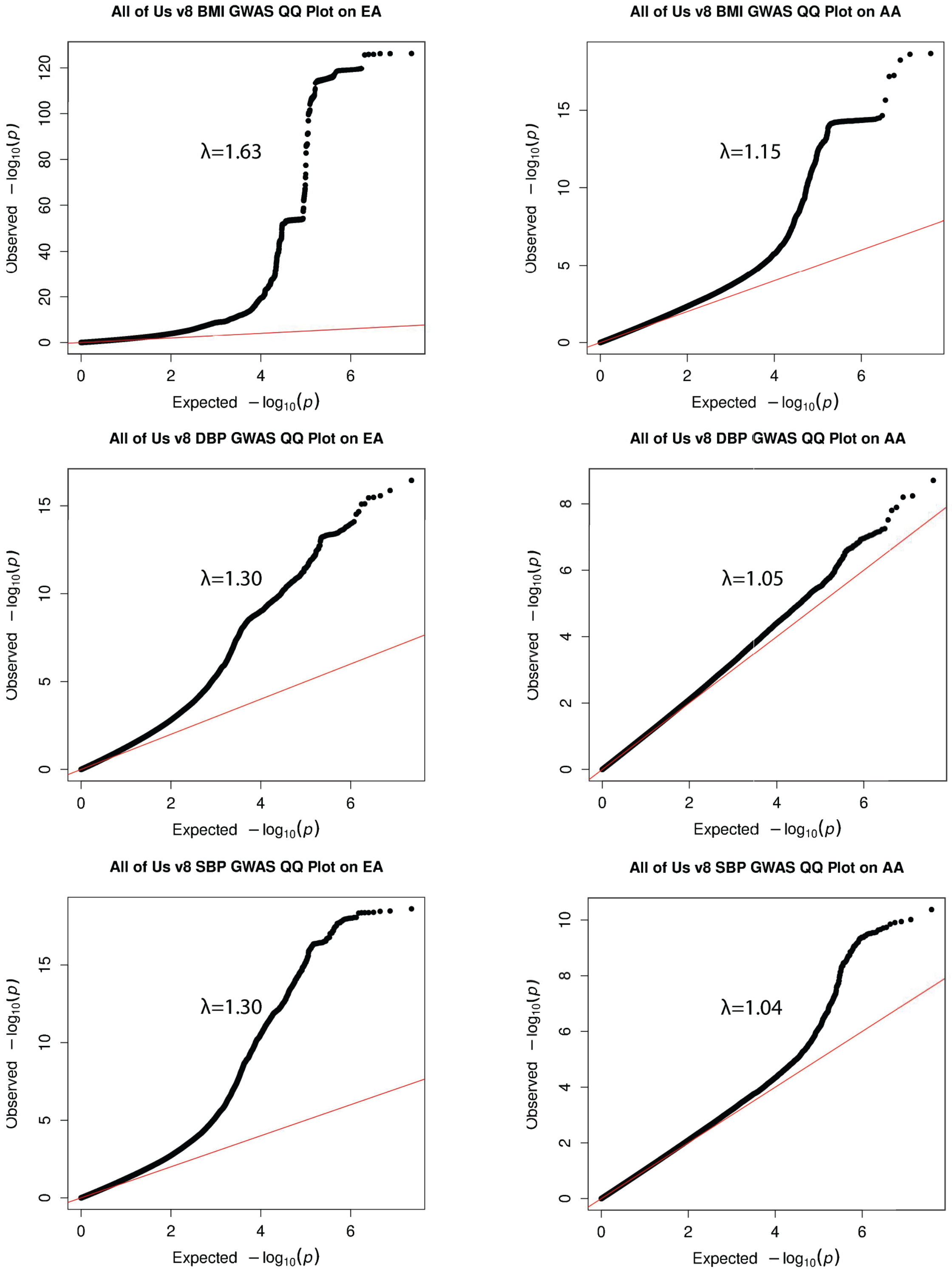

Supplementary Figure 50: **GWAS QQ plots for All of Us traits.** This figure displays Quantile-Quantile (QQ) plots stratified by trait and genetic ancestry, with rows representing Body Mass Index (BMI, top), Diastolic Blood Pressure (DBP, middle), and Systolic Blood Pressure (SBP, bottom), and columns distinguishing between European Ancestry (EA, left) and African Ancestry (AA, right). In each panel, the observed negative base-10 logarithm of the P-values ($-\log_{10}(P)$) are plotted on the y-axis against the expected values under a theoretical null distribution on the x-axis. The solid red line represents the null expectation of no association, and the genomic inflation factor (λ) is annotated in each plot to summarize the deviation of the observed statistics from the null.

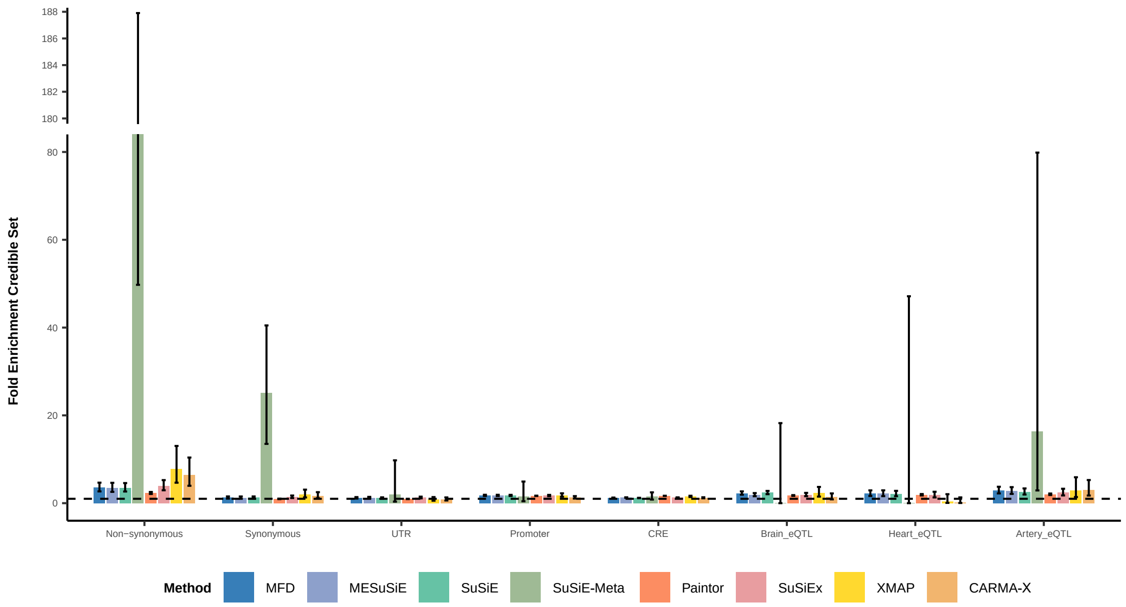

Supplementary Figure 51: **Functional enrichment comparison of fine-mapped variants in All of Us traits.** This analysis evaluates variants within 95% credible sets across 289 candidate regions for three traits: Body Mass Index (BMI), Diastolic Blood Pressure (DBP), and Systolic Blood Pressure (SBP). Relative enrichment is calculated as the ratio of the proportion of functionally annotated SNPs within the 95% credible sets to the proportion of annotated SNPs outside of these sets across eight functional annotation categories: non-synonymous mutation, synonymous mutation, promoter, untranslated regions (UTRs), cis-regulatory elements (CRE), independent heart eQTL, independent artery eQTL, and independent brain eQTL. Error bars represent 95% confidence intervals.

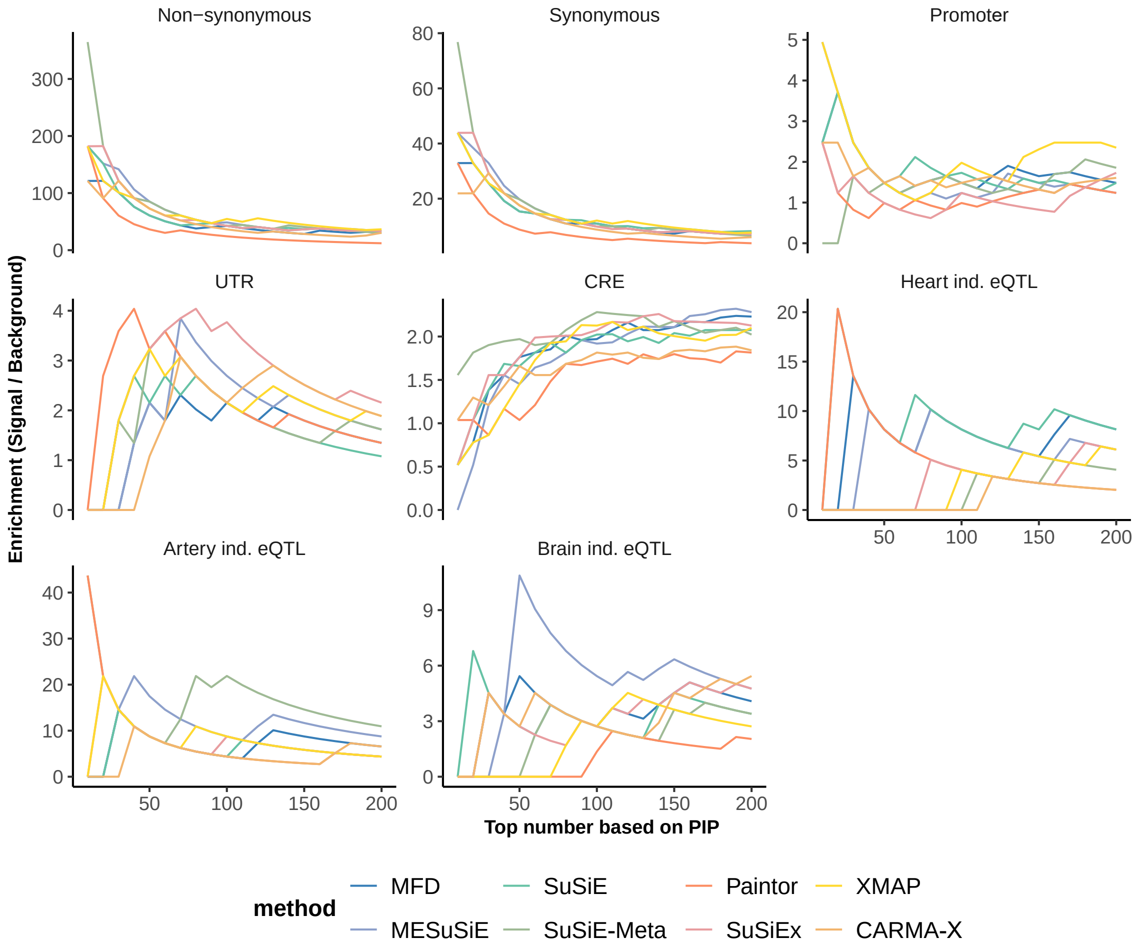

Supplementary Figure 52: **Functional enrichment of fine-mapped variants across varying top-ranked thresholds.** This figure compares the functional enrichment of variants identified by evaluated fine-mapping methods, aggregated across three quantitative traits (BMI, DBP, SBP). Panels display enrichment for eight functional annotation categories: non-synonymous mutation, synonymous mutation, promoter, untranslated regions (UTRs), cis-regulatory elements (CRE), independent heart eQTL, independent artery eQTL, and independent brain eQTL as a function of the number of top-ranked variants selected (ranging from the top 10 to 200 variants based on PIP_{either}, shown on the x-axis). The y-axis represents the fold enrichment calculated as the ratio of the proportion of annotated variants within the selected top set to the proportion of that annotation in the background set.

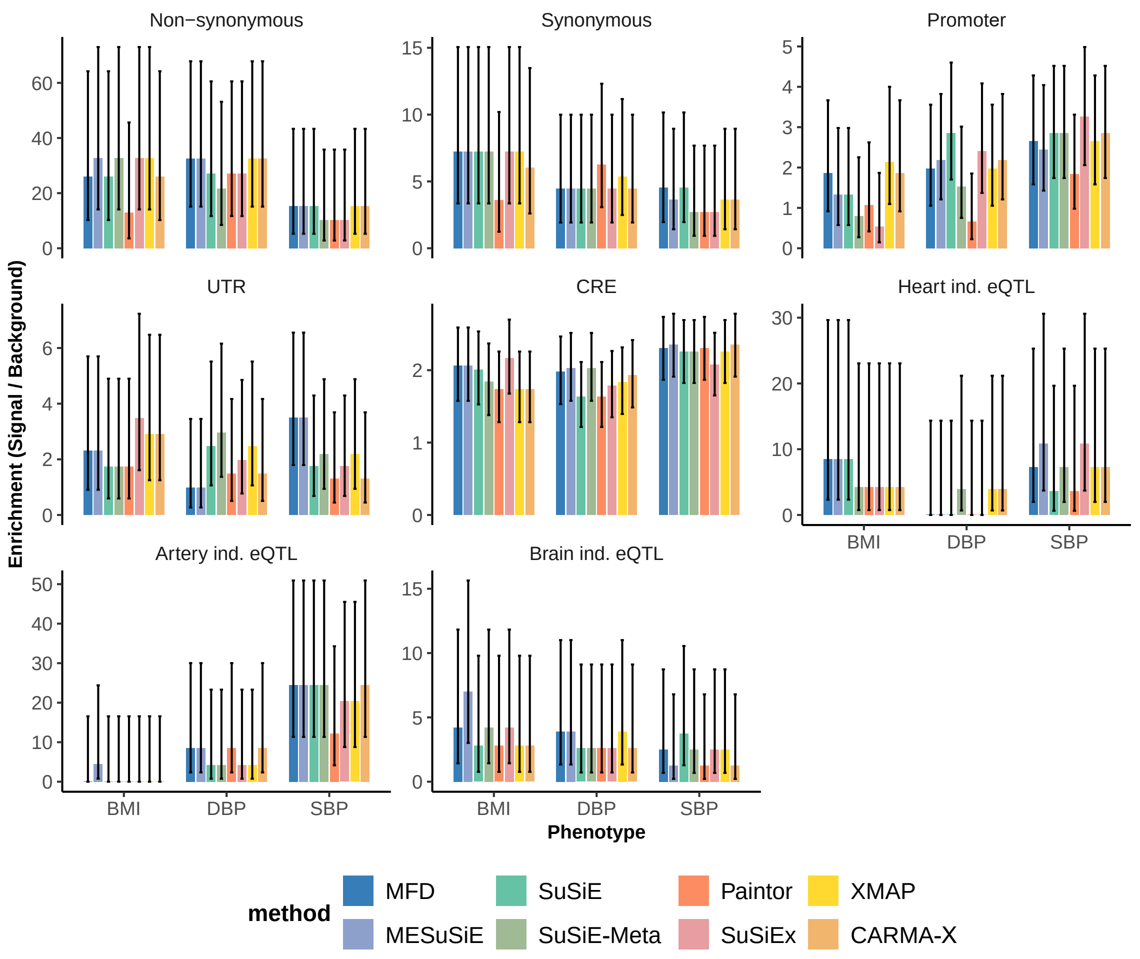

Supplementary Figure 53: **Functional enrichment of top 100 fine-mapped signals.** This figure summarizes multi-ancestry fine-mapping results for Body Mass Index (BMI), Diastolic Blood Pressure (DBP), and Systolic Blood Pressure (SBP). It displays the fold enrichment across eight functional annotation categories: non-synonymous mutation, synonymous mutation, promoter, untranslated regions (UTRs), cis-regulatory elements (CRE), independent heart eQTL, independent artery eQTL, and independent brain eQTL. Enrichment is calculated as the ratio of the proportion of potential causal SNPs (defined as the top 100 variants based on PIP_{either}) carrying a specific annotation to the proportion of that annotation in the background set. Error bars represent 95% confidence intervals.

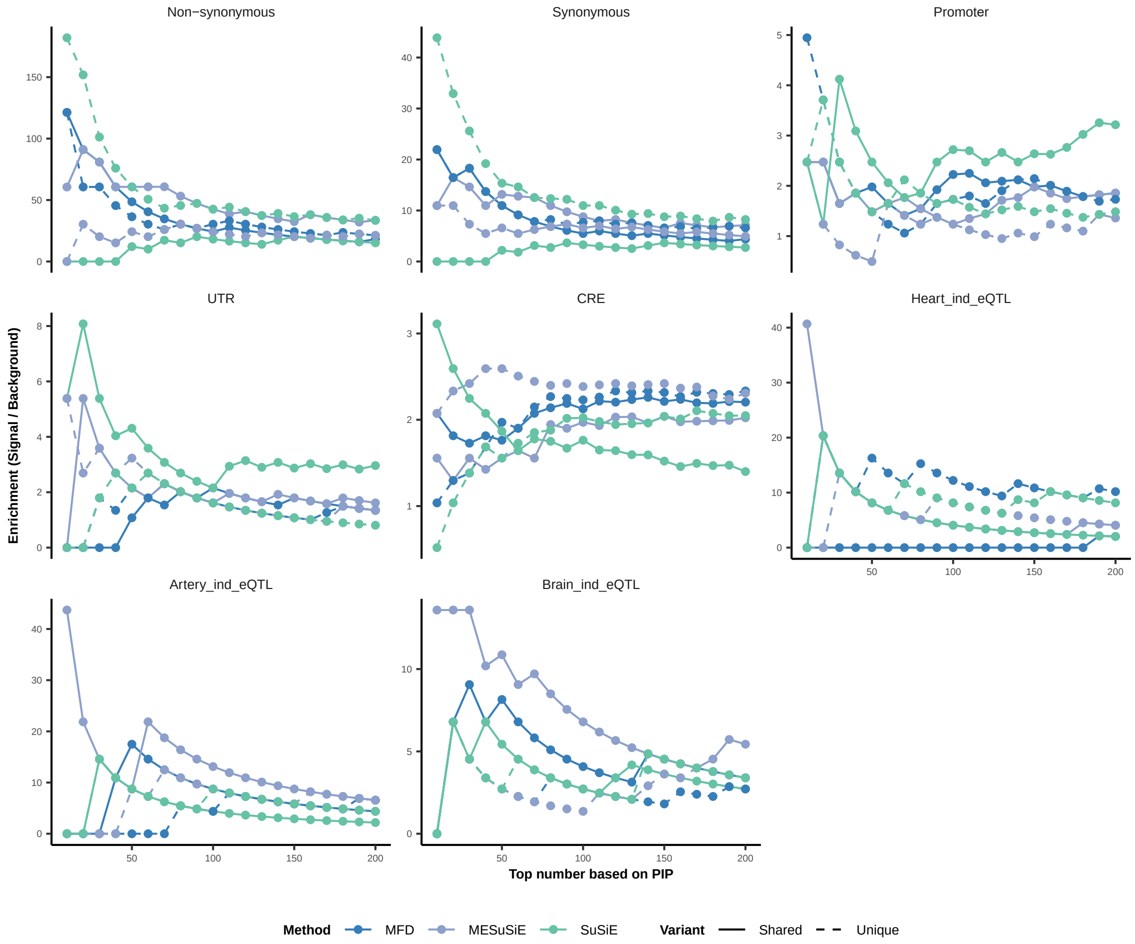

Supplementary Figure 54: **Functional enrichment of cross-ancestry and ancestry-specific (unique) fine-mapped signals.** This figure evaluates the functional enrichment of variants classified as cross-ancestry (solid lines) or ancestry-specific/unique (dashed lines), aggregated across three quantitative traits (BMI, DBP, SBP). The analysis compares three methods (MFD, MESuSiE, and SuSiE) across functional annotation categories (Non-synonymous mutation, synonymous mutation, Promoter, untranslated regions (UTRs), cis-regulatory elements (CRE), and independent heart/artery/brain eQTLs). The x-axis displays the number of top-ranked variants selected based on PIP_{shared} for cross-ancestry signals, PIP_{WB} and PIP_{BB} for ancestry-specific signals (ranging from 10 to 200), and the y-axis represents the fold enrichment, calculated as the ratio of the proportion of annotated variants in the selected set to the proportion in the background set.

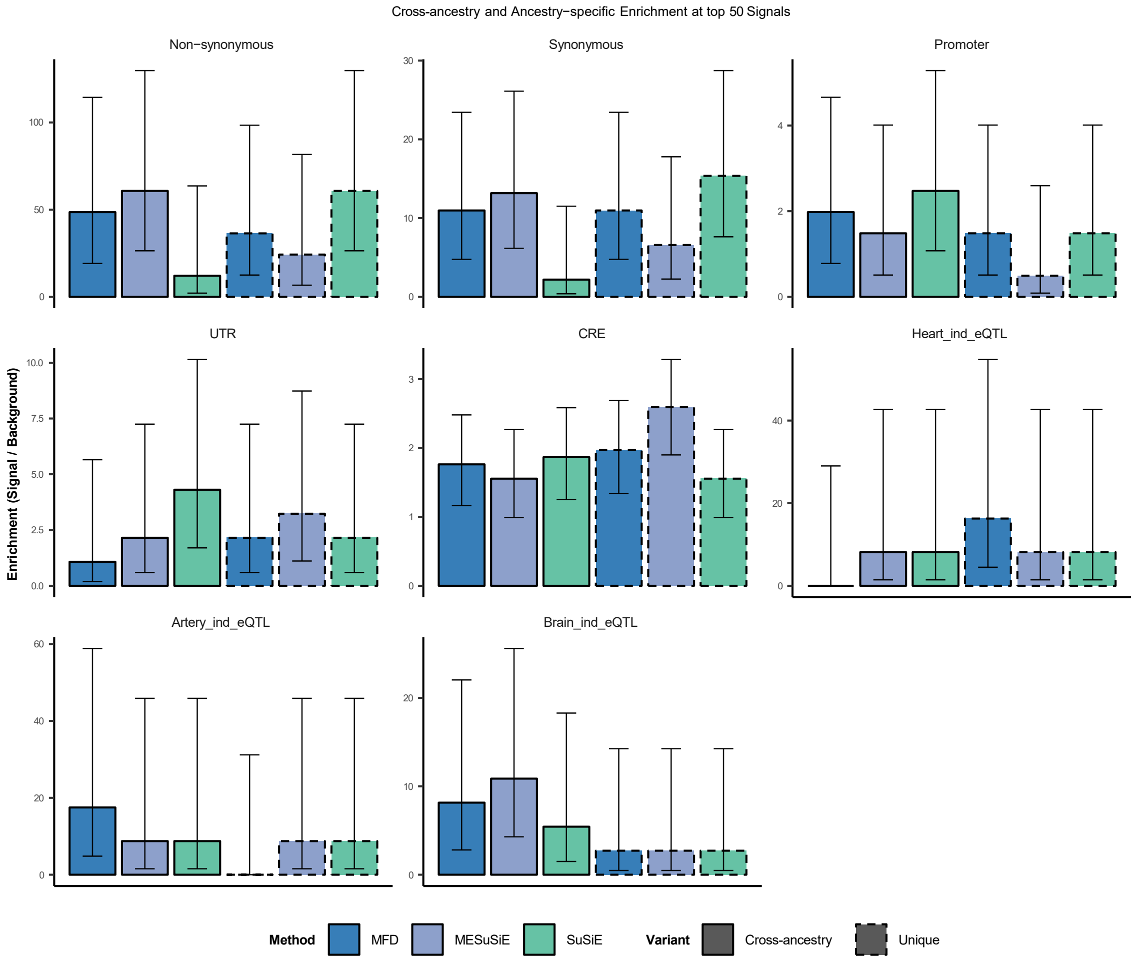

Supplementary Figure 55: **Functional enrichment of top 50 cross-ancestry and ancestry-specific (unique) fine-mapped signals.** This figure evaluates the functional enrichment of variants classified as cross-ancestry (solid lines) or ancestry-specific/unique (dashed lines), aggregated across three quantitative traits (BMI, DBP, SBP). The analysis compares three methods (MFD, MESuSiE, and SuSiE) across functional annotation categories (Non-synonymous mutation, synonymous mutation, Promoter, untranslated regions (UTRs), cis-regulatory elements (CRE), and independent heart/artery/brain eQTLs). The x-axis displays the number of top-ranked variants selected based on PIP_{shared} for cross-ancestry signals, PIP_{WB} and PIP_{BB} for ancestry-specific signals, and the y-axis represents the fold enrichment, calculated as the ratio of the proportion of annotated variants in the selected set to the proportion in the background set. Error bars represent 95% confidence intervals.

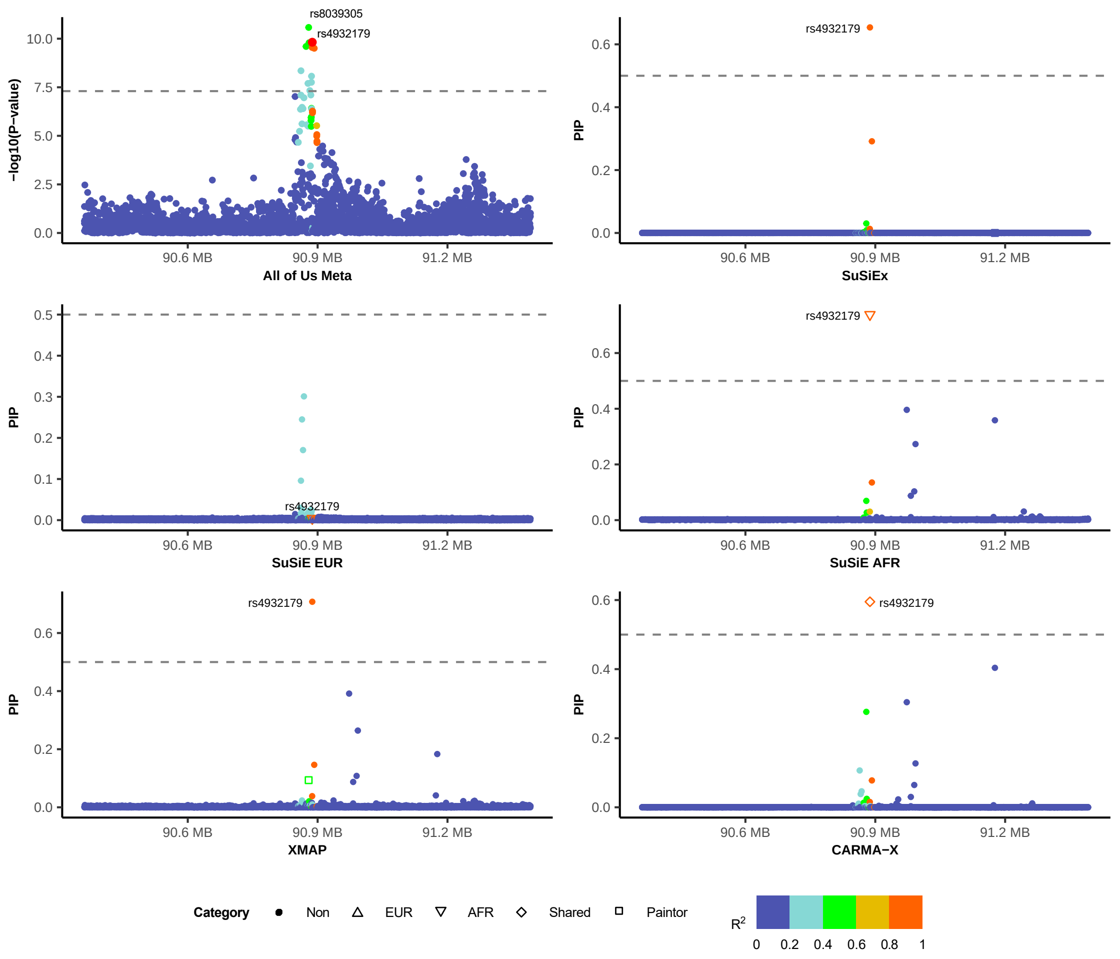

Supplementary Figure 56: **Regional association and fine-mapping comparison for the DBP locus around *FES*.** The top-left panel displays the fixed-effect meta-analysis results for Diastolic Blood Pressure (DBP), plotting the negative base-10 logarithm of the P-value against genomic position on chromosome 11. The remaining panels present the Posterior Inclusion Probabilities (PIP) for variants in this region as estimated by different methods: SuSiEx (PIP_{either}), SuSiE (PIP_{WB}), SuSiE (PIP_{BB}), XMAP (PIP_{either}), and CARMA-X (PIP_{either}). The lead variant rs4932179 is annotated across all panels, rs8093905 is the variant with most significant P-value in fixed-effect meta-analysis. In the PIP panels, we denote European-specific signals by upward-pointing triangles, African-specific signals by downward-pointing triangles, cross-ancestry (shared) signals by diamonds, Paintor-only signals by squares, and non-signals by dots.

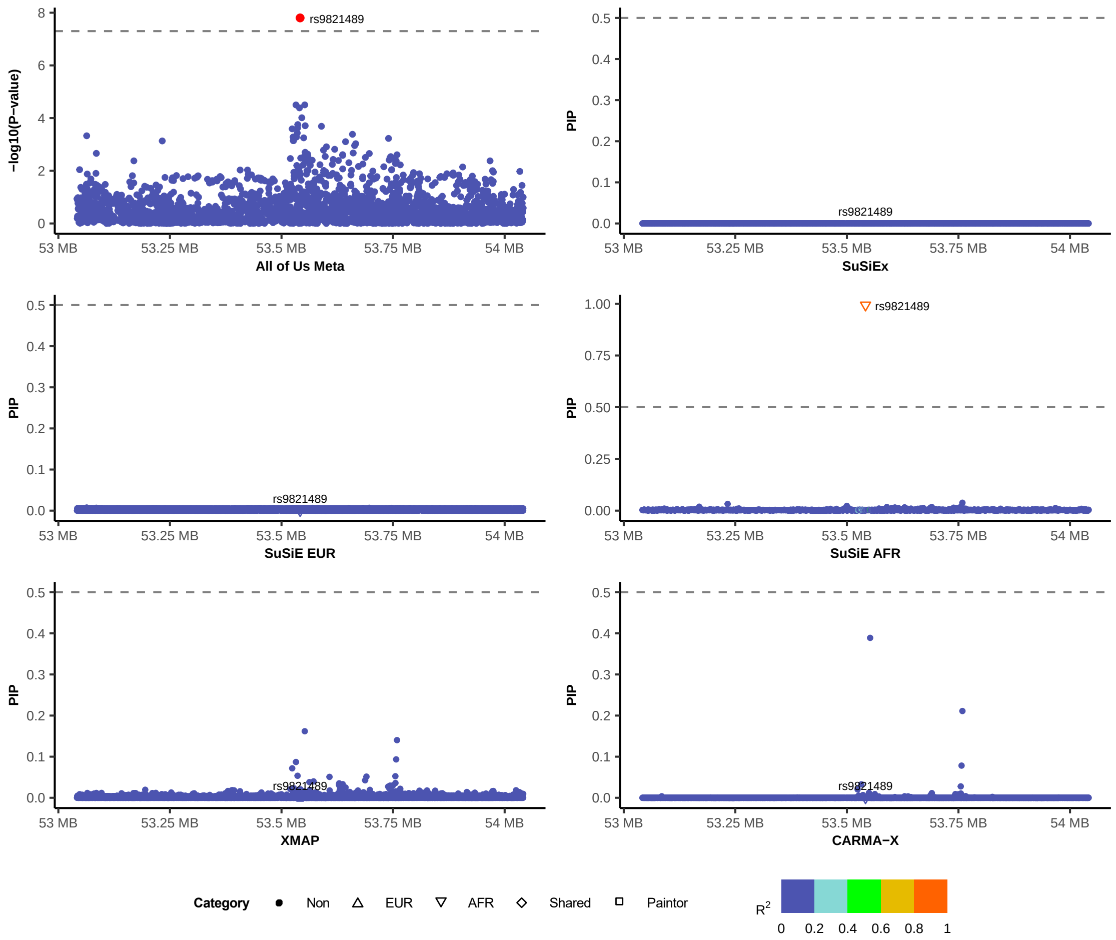

Supplementary Figure 57: **Regional association and fine-mapping comparison for the DBP locus around *CACNA1D*.** The top-left panel displays the fixed-effect meta-analysis results for Diastolic Blood Pressure (DBP), plotting the negative base-10 logarithm of the P-value against genomic position on chromosome 3. The remaining panels present the Posterior Inclusion Probabilities (PIP) for variants in this region as estimated by different methods: SuSiEx (PIP_{either}), SuSiE (PIP_{WB}), SuSiE (PIP_{BB}), XMAP (PIP_{either}), and CARMA-X (PIP_{either}). The lead variant rs9821489 is annotated across all panels. In the PIP panels, we denote European-specific signals by upward-pointing triangles, African-specific signals by downward-pointing triangles, cross-ancestry (shared) signals by diamonds, Paintor-only signals by squares, and non-signals by dots.

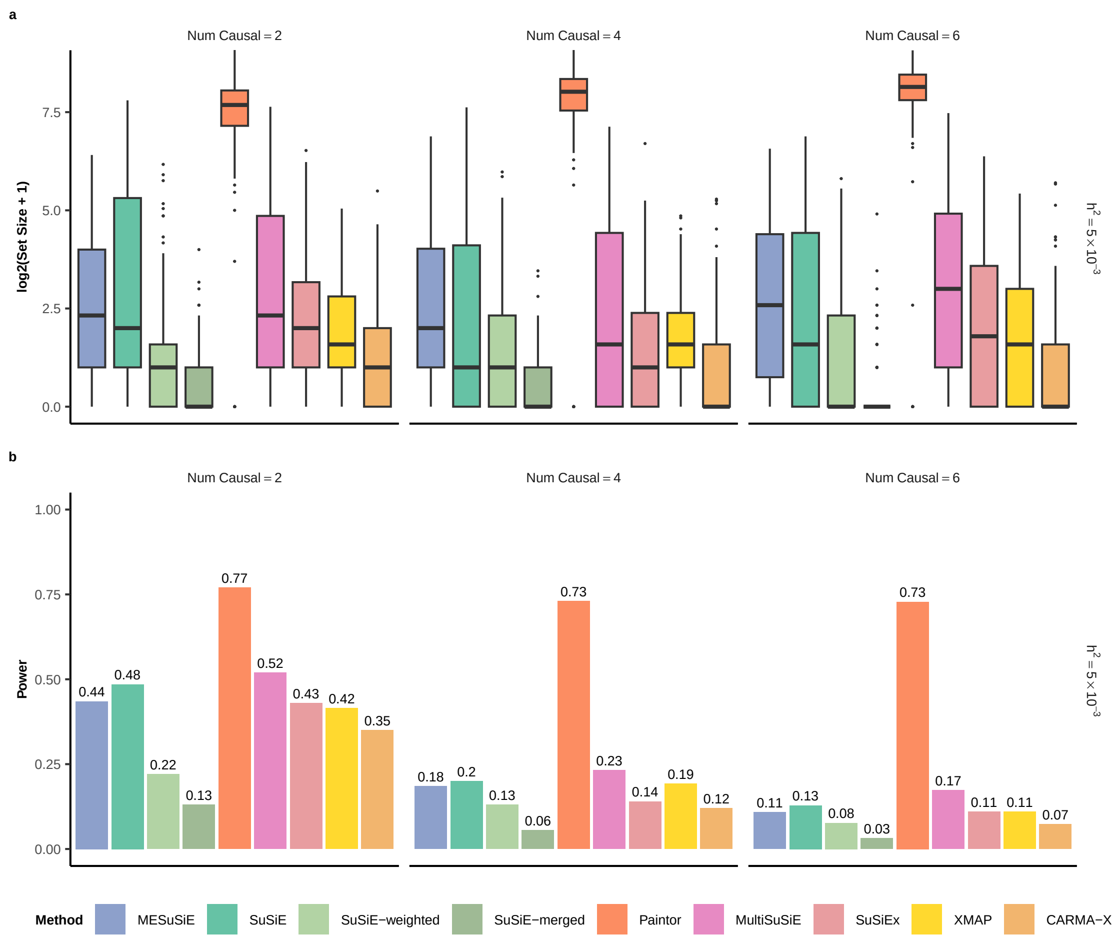

Supplementary Figure 58: **Performance metrics based on credible sets (Admixed-cohort simulation).** Comparison of 95% credible set sizes and power in detecting causal signals with non-zero effects in at least one local-ancestry component. The admixed cohort comprises 10,000 individuals with 80% CEU and 20% YRI ancestry. Local ancestry-specific summary statistics (EUR and AFR components) were derived from the same admixed individuals. Results are summarized across $n = 100$ independent regions, stratified by the number of causal SNPs (2, 4, or 6 total causal SNPs per region) with total regional heritability $h^2 = 0.005$. **(a)** Boxplots displaying the $\log_2(\text{set size} + 1)$ of the 95% credible sets. **(b)** Bar plots displaying the power of the constructed 95% credible sets, defined as the proportion of simulated causal variants successfully included in the credible set.

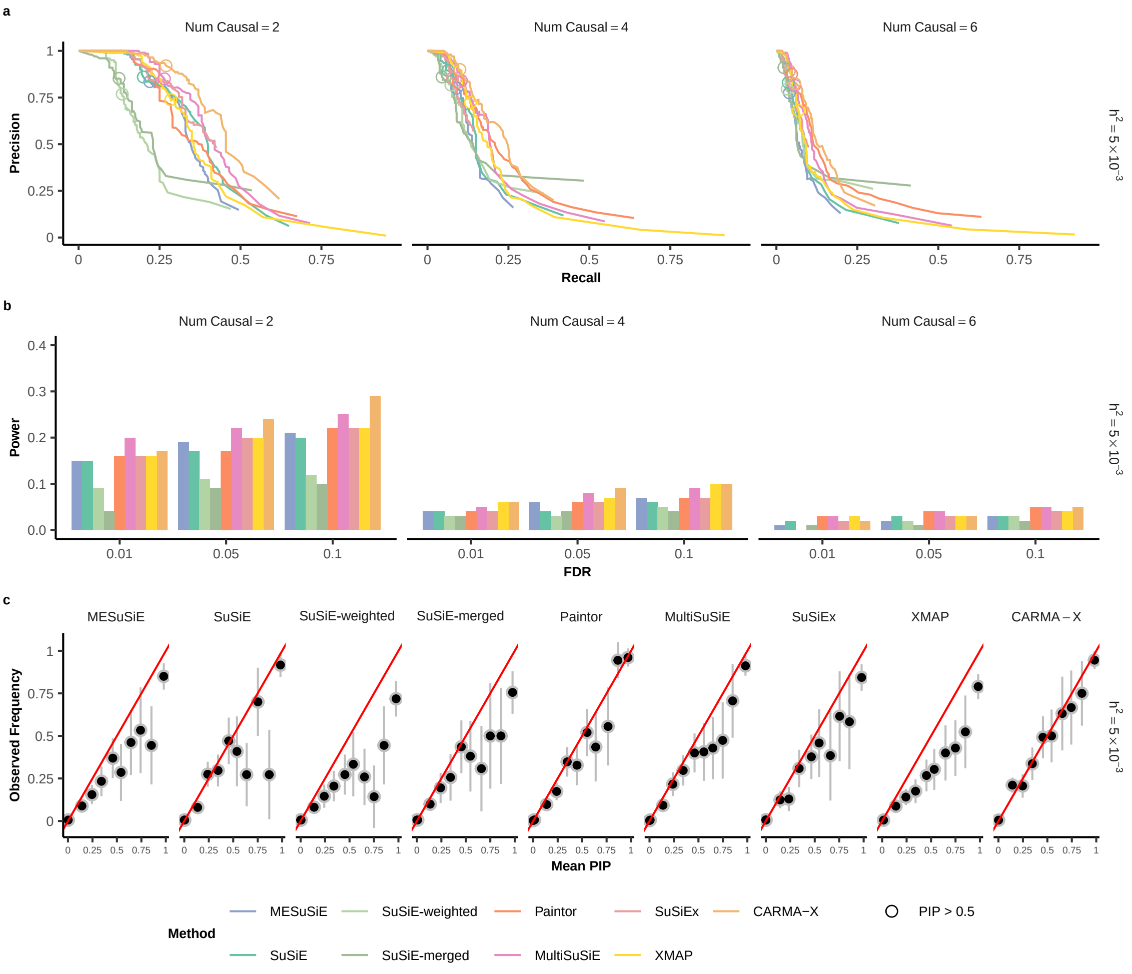

Supplementary Figure 59: **Comparison of method performance in detecting causal signals with non-zero effects in at least one local-ancestry component (Admixed-cohort simulation, 'Either').** The admixed cohort comprises 10,000 individuals with 80% CEU and 20% YRI ancestry. Results are stratified by the number of causal SNPs (2, 4, or 6 total causal SNPs per region) with total regional heritability $h^2 = 0.005$. **(a)** Precision-recall curves with PIP_{either} thresholds ranging from 0.01 to 1. Precision is defined as $1 - \text{FDR}$ and Recall as $\frac{\text{TP}}{\text{TP+FN}}$, where TP, FP, and FN represent true positives, false positives, and false negatives, respectively. The PIP threshold of 0.5 is marked with a circle. **(b)** Bar plots displaying power at controlled FDR levels of 0.01, 0.05, and 0.1. **(c)** Calibration plots aggregating $n = 300$ independent regions (combining all causal SNP scenarios). SNPs are ranked by PIP and divided into 10 equally sized bins. The y-axis shows the observed proportion of causal signals, and the x-axis shows the mean PIP within each bin. Gray error bars represent ± 2 standard errors. The red diagonal line indicates perfect calibration; points below the line suggest inflated PIPs (more false discoveries), while points above indicate conservative PIPs.

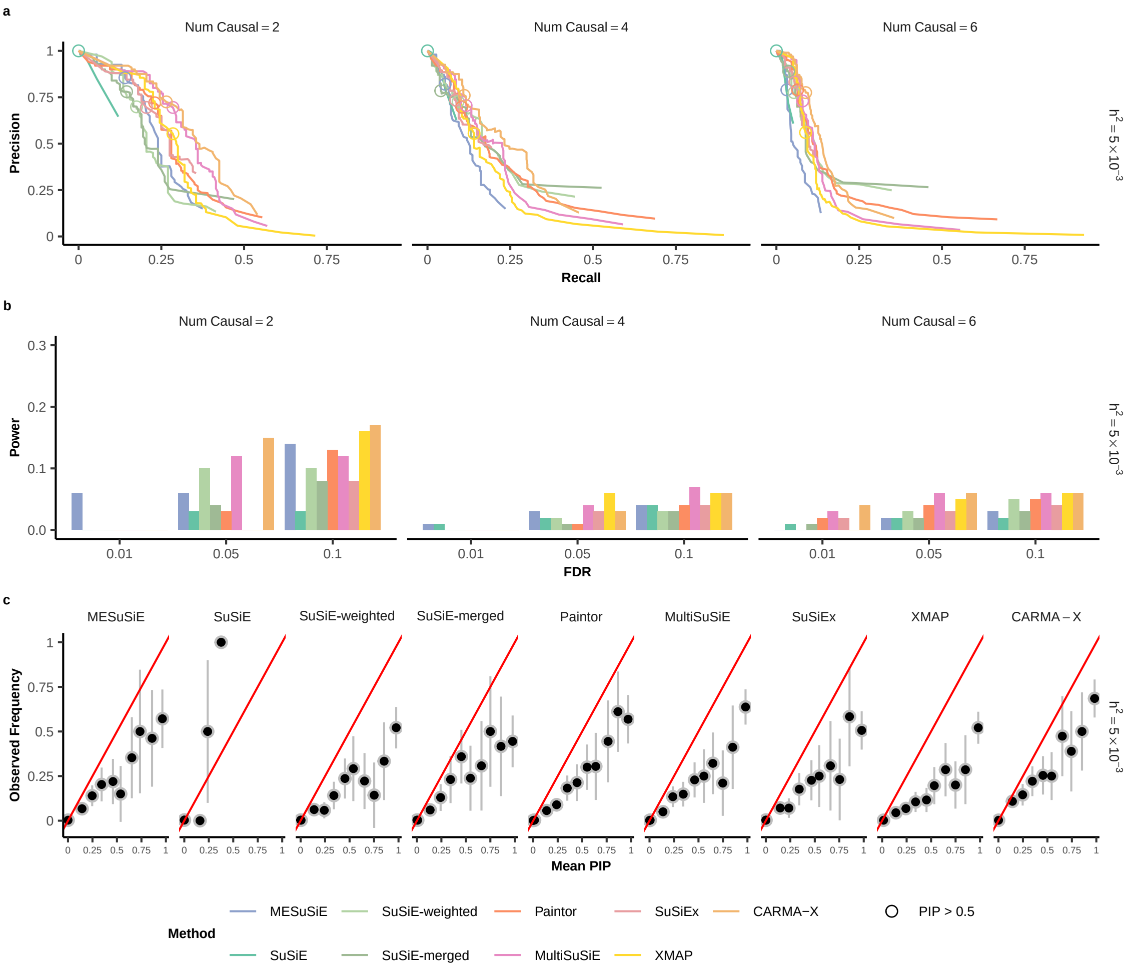

Supplementary Figure 60: **Comparison of method performance in detecting causal signals with non-zero effects in both local-ancestry components (Admixed-cohort simulation, 'Cross-ancestry').** The admixed cohort comprises 10,000 individuals with 80% CEU and 20% YRI ancestry. Results are stratified by the number of causal SNPs (2, 4, or 6 total causal SNPs per region) with total regional heritability $h^2 = 0.005$. **(a)** Precision-recall curves with PIP_{shared} thresholds ranging from 0.01 to 1. Precision is defined as 1 − FDR and Recall as $\frac{\mathrm{TP}}{\mathrm{TP+FN}}$, where TP, FP, and FN represent true positives, false positives, and false negatives, respectively. The PIP threshold of 0.5 is marked with a circle. **(b)** Bar plots displaying power at controlled FDR levels of 0.01, 0.05, and 0.1. **(c)** Calibration plots aggregating $n = 300$ independent regions (combining all causal SNP scenarios). SNPs are ranked by PIP and divided into 10 equally sized bins. The y-axis shows the observed proportion of causal signals, and the x-axis shows the mean PIP within each bin. Gray error bars represent ±2 standard errors. The red diagonal line indicates perfect calibration; points below the line suggest inflated PIPs (more false discoveries), while points above indicate conservative PIPs.

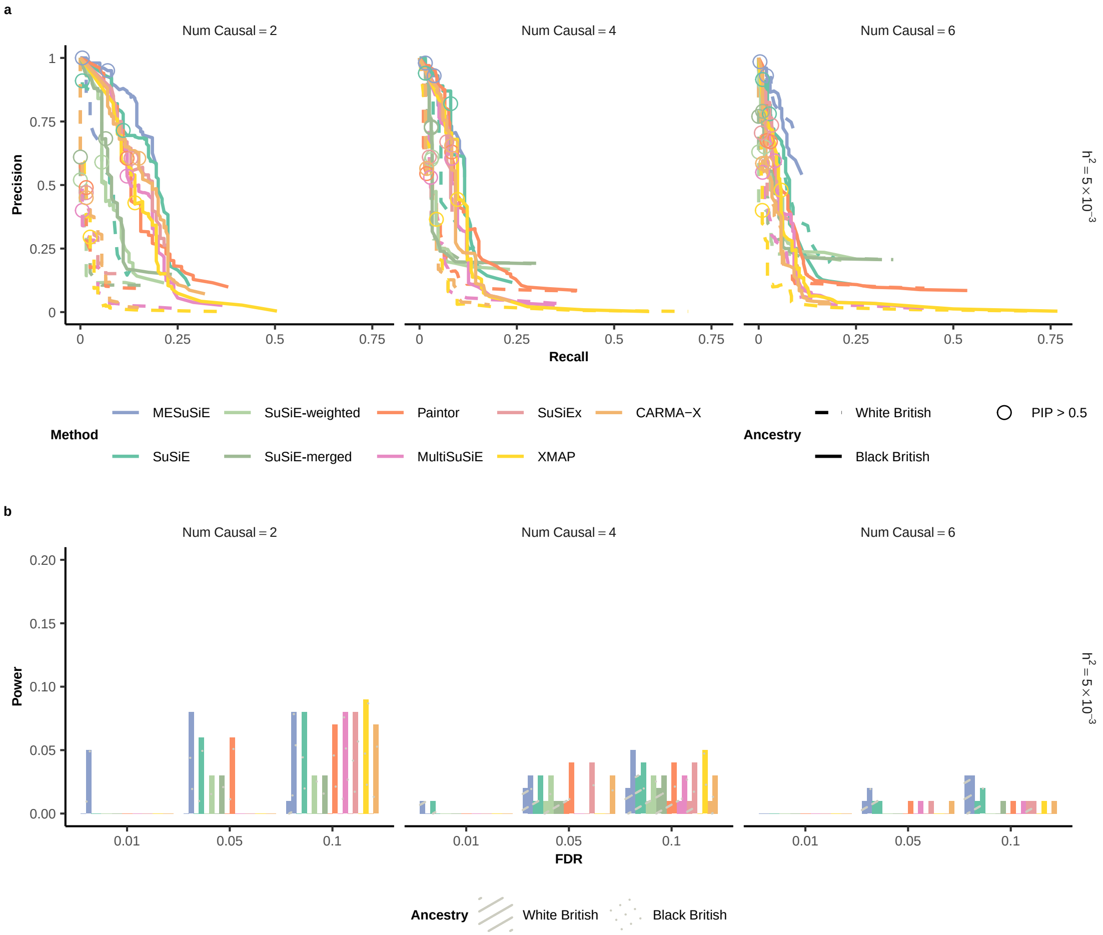

Supplementary Figure 61: **Performance metrics for ancestry-specific contextual causal signals (Admixed-cohort simulation).** Comparison of method performance in detecting causal signals with non-zero effects in the EUR local-ancestry component only and the AFR local-ancestry component only. The admixed cohort comprises 10,000 individuals with 80% CEU and 20% YRI ancestry. Results are stratified by the number of causal SNPs (2, 4, or 6 total causal SNPs per region) with total regional heritability $h^2 = 0.005$. **(a)** Precision-recall curves with ancestry-specific PIP thresholds ranging from 0.01 to 1. Precision is defined as $1 - \text{FDR}$ and Recall as $\frac{\text{TP}}{\text{TP+FN}}$, where TP, FP, and FN represent true positives, false positives, and false negatives, respectively. The PIP threshold of 0.5 is marked with a circle. **(b)** Bar plots displaying power at controlled FDR levels of 0.01, 0.05, and 0.1.

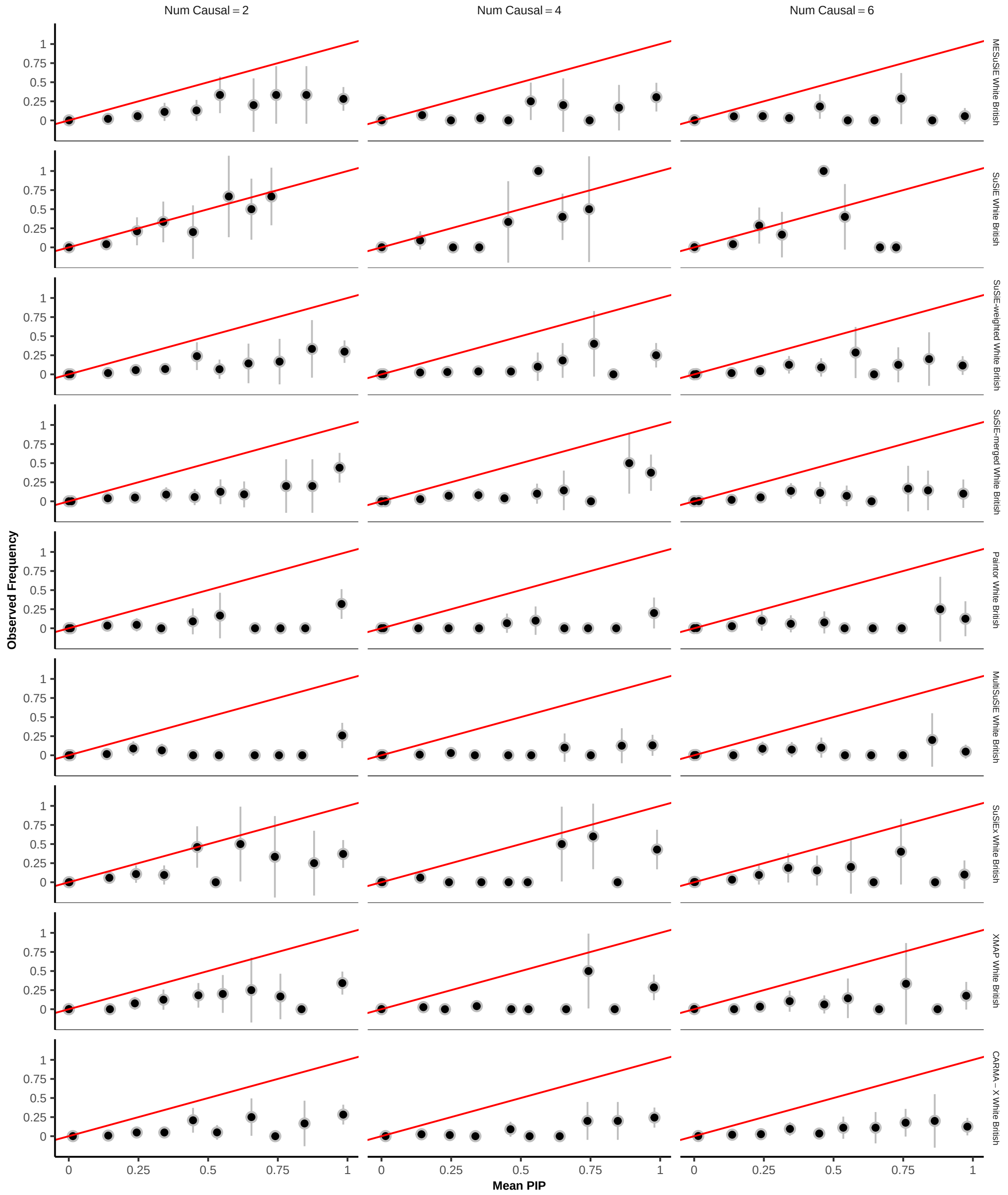

Supplementary Figure 62: **PIP Calibration for the EUR local-ancestry component (Admixed-cohort simulation).** Calibration plots aggregating $n = 300$ independent regions (combining all causal SNP scenarios). The admixed cohort comprises 10,000 individuals with 80% CEU and 20% YRI ancestry. Local ancestry-specific summary statistics (EUR and AFR components) were derived from the same admixed individuals. SNPs are ranked by PIP and divided into 10 equally sized bins. The y-axis shows the observed proportion of causal signals, and the x-axis shows the mean PIP within each bin. Gray error bars represent ± 2 standard errors. The red diagonal line indicates perfect calibration; points below the line suggest inflated PIPs (more false discoveries), while points above indicate conservative PIPs.

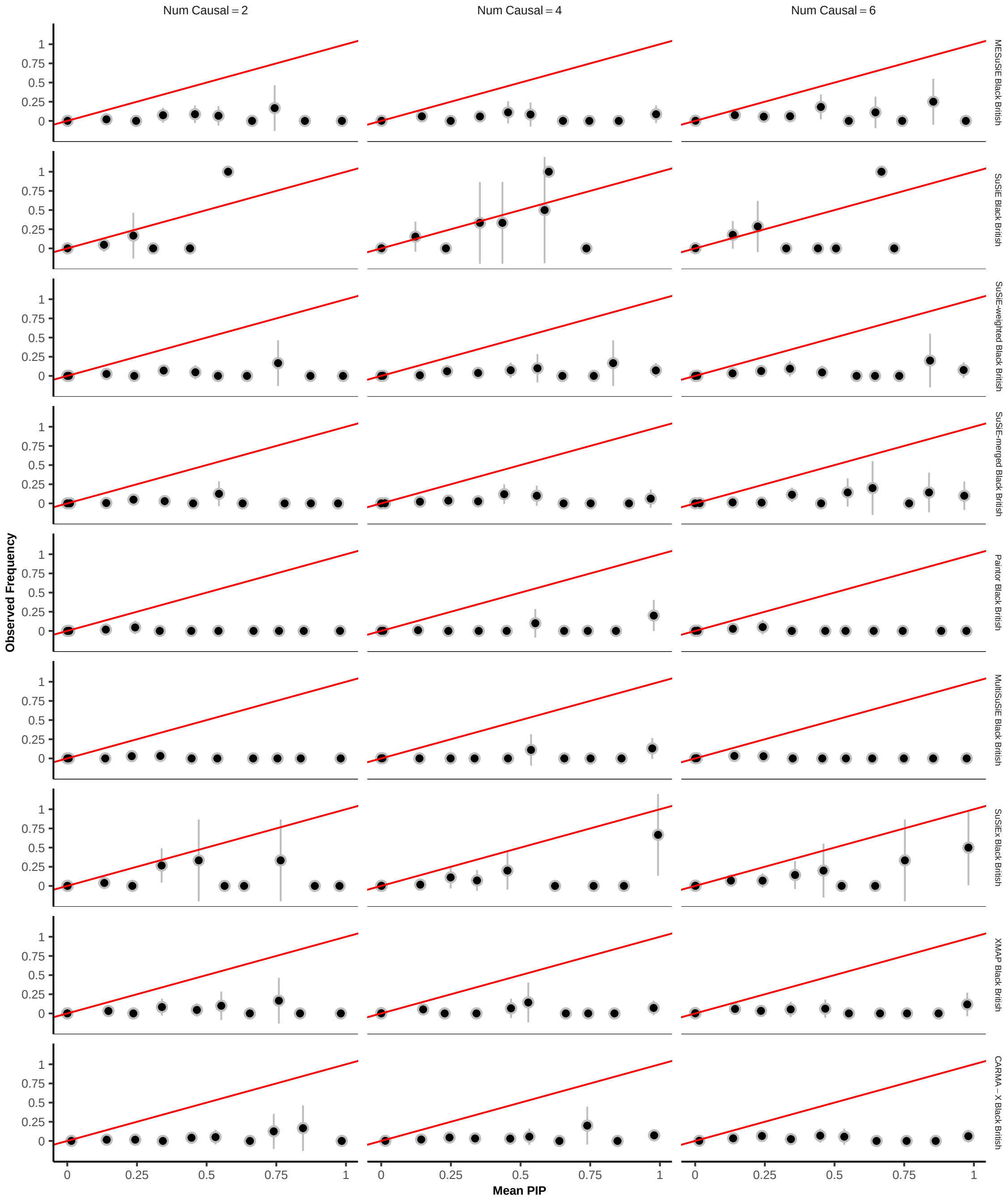

Supplementary Figure 63: **PIP Calibration for the AFR local-ancestry component (Admixed-cohort simulation).** Calibration plots aggregating $n = 300$ independent regions (combining all causal SNP scenarios). The admixed cohort comprises 10,000 individuals with 80% CEU and 20% YRI ancestry. Local ancestry-specific summary statistics (EUR and AFR components) were derived from the same admixed individuals. SNPs are ranked by PIP and divided into 10 equally sized bins. The y-axis shows the observed proportion of causal signals, and the x-axis shows the mean PIP within each bin. Gray error bars represent ± 2 standard errors. The red diagonal line indicates perfect calibration; points below the line suggest inflated PIPs (more false discoveries), while points above indicate conservative PIPs.

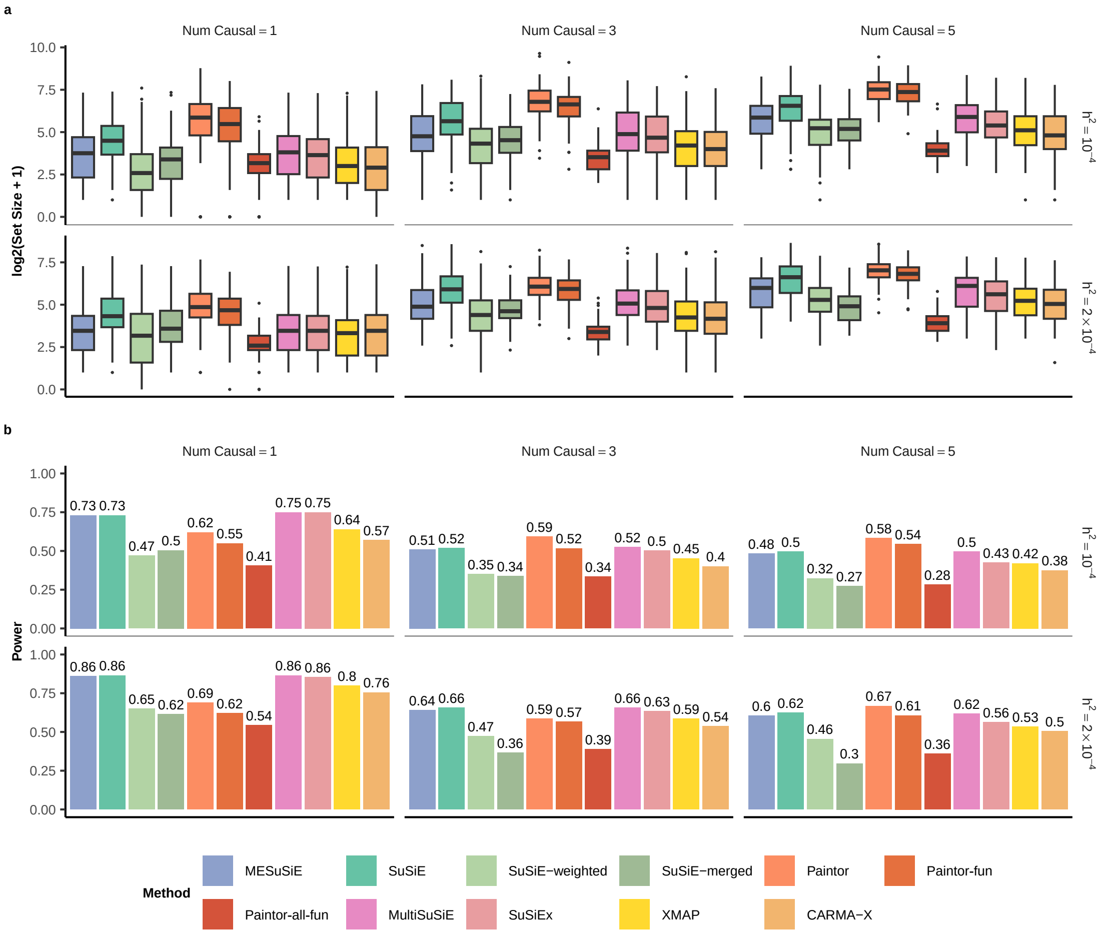

Supplementary Figure 64: **Performance metrics based on credible sets (Functional annotation sensitivity, $w = 0$, uniform causal SNP sampling).** Comparison of 95% credible set sizes and power in detecting causal signals with non-zero effects in at least one ancestry under the functional annotation simulation with enrichment weight $w = 0$ (causal SNPs sampled uniformly with respect to annotations). Results are summarized across $n = 100$ independent regions, stratified by the number of causal SNPs (1, 3, or 5 cross-ancestry, corresponding to a total of 2, 6, and 10 causal SNPs, respectively) and per-causal SNP heritability ($h^2 = 10^{-4}$ or 2×10^{-4}; rows). **(a)** Boxplots displaying the $\log_2(\text{set size} + 1)$ of the 95% credible sets. **(b)** Bar plots displaying the power of the constructed 95% credible sets, defined as the proportion of simulated causal variants successfully included in the credible set.

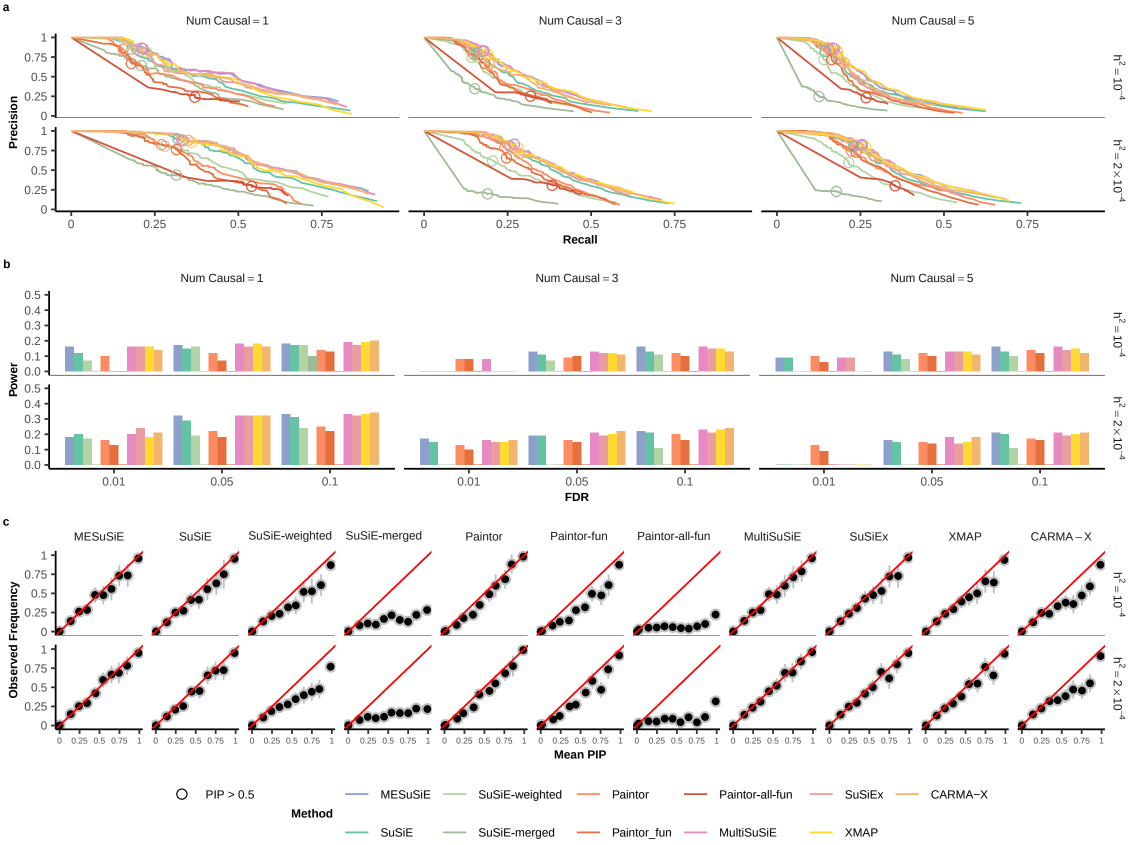

Supplementary Figure 65: **Comparison of method performance in detecting causal signals with non-zero effects in at least one ancestry (Functional annotation sensitivity, $w = 0$, 'Either').** Results under uniform causal SNP sampling ($w = 0$, no annotation enrichment) are stratified by the number of cross-ancestry causal SNPs (1, 3, or 5, corresponding to a total of 2, 6, and 10 causal SNPs, respectively) and per-causal SNP heritability ($h^2 = 10^{-4}$ or 2×10^{-4}; rows). **(a)** Precision-recall curves with PIP_{either} thresholds ranging from 0.01 to 1. Precision is defined as $1 - \text{FDR}$ and Recall as $\frac{\text{TP}}{\text{TP+FN}}$, where TP, FP, and FN represent true positives, false positives, and false negatives, respectively. The PIP threshold of 0.5 is marked with a circle. **(b)** Bar plots displaying power at controlled FDR levels of 0.01, 0.05, and 0.1. **(c)** Calibration plots aggregating $n = 300$ independent regions (combining all causal SNP scenarios). SNPs are ranked by PIP and divided into 10 equally sized bins. The y-axis shows the observed proportion of causal signals, and the x-axis shows the mean PIP within each bin. Gray error bars represent ± 2 standard errors. The red diagonal line indicates perfect calibration; points below the line suggest inflated PIPs (more false discoveries), while points above indicate conservative PIPs.

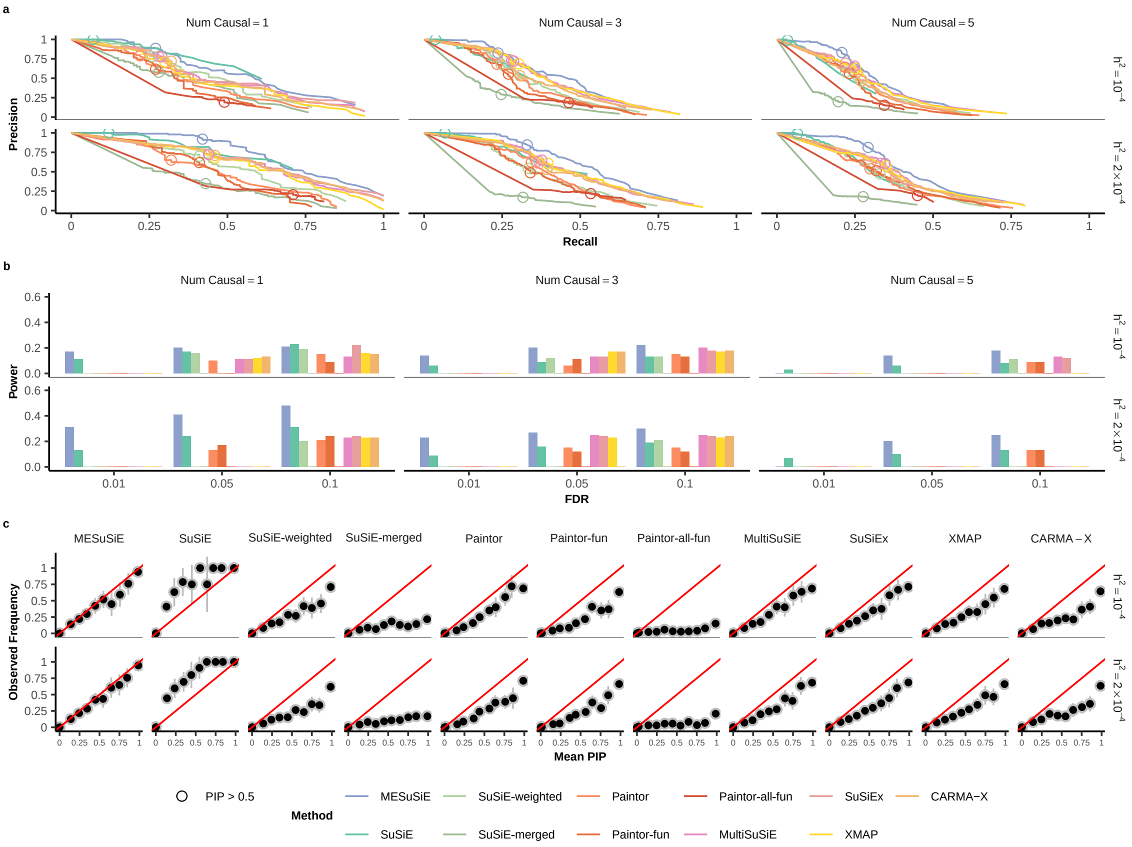

Supplementary Figure 66: **Comparison of method performance in detecting causal signals with non-zero effects in both ancestries (Functional annotation sensitivity, $w = 0$, 'Cross-ancestry').** Results under uniform causal SNP sampling ($w = 0$, no annotation enrichment) are stratified by the number of cross-ancestry causal SNPs (1, 3, or 5, corresponding to a total of 2, 6, and 10 causal SNPs, respectively) and per-causal SNP heritability ($h^2 = 10^{-4}$ or 2×10^{-4}; rows). **(a)** Precision-recall curves with PIP_{shared} thresholds ranging from 0.01 to 1. Precision is defined as $1 - \text{FDR}$ and Recall as $\frac{\text{TP}}{\text{TP+FN}}$, where TP, FP, and FN represent true positives, false positives, and false negatives, respectively. The PIP threshold of 0.5 is marked with a circle. **(b)** Bar plots displaying power at controlled FDR levels of 0.01, 0.05, and 0.1. **(c)** Calibration plots aggregating $n = 300$ independent regions (combining all causal SNP scenarios). SNPs are ranked by PIP and divided into 10 equally sized bins. The y-axis shows the observed proportion of causal signals, and the x-axis shows the mean PIP within each bin. Gray error bars represent ± 2 standard errors. The red diagonal line indicates perfect calibration; points below the line suggest inflated PIPs (more false discoveries), while points above indicate conservative PIPs.

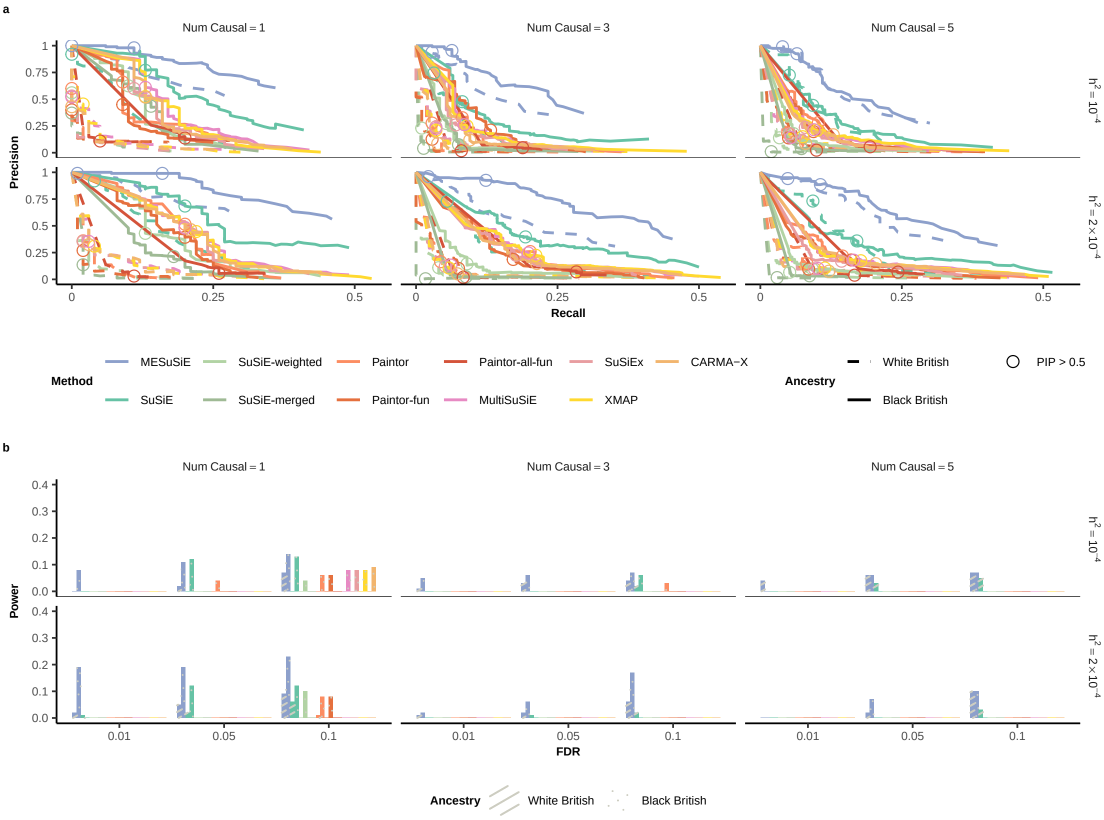

Supplementary Figure 67: **Performance metrics for ancestry-specific contextual causal signals (Functional annotation sensitivity, $w = 0$).** Comparison of method performance in detecting causal signals with non-zero effects in White British only and Black British only under uniform causal SNP sampling ($w = 0$, no annotation enrichment). Results are stratified by the number of cross-ancestry causal SNPs (1, 3, or 5, corresponding to a total of 2, 6, and 10 causal SNPs, respectively) and per-causal SNP heritability ($h^2 = 10^{-4}$ or 2×10^{-4}; rows). **(a)** Precision-recall curves with PIP_{WB} and PIP_{BB} thresholds ranging from 0.01 to 1. Precision is defined as $1 - \text{FDR}$ and Recall as $\frac{\text{TP}}{\text{TP+FN}}$, where TP, FP, and FN represent true positives, false positives, and false negatives, respectively. The PIP threshold of 0.5 is marked with a circle. **(b)** Bar plots displaying power at controlled FDR levels of 0.01, 0.05, and 0.1.

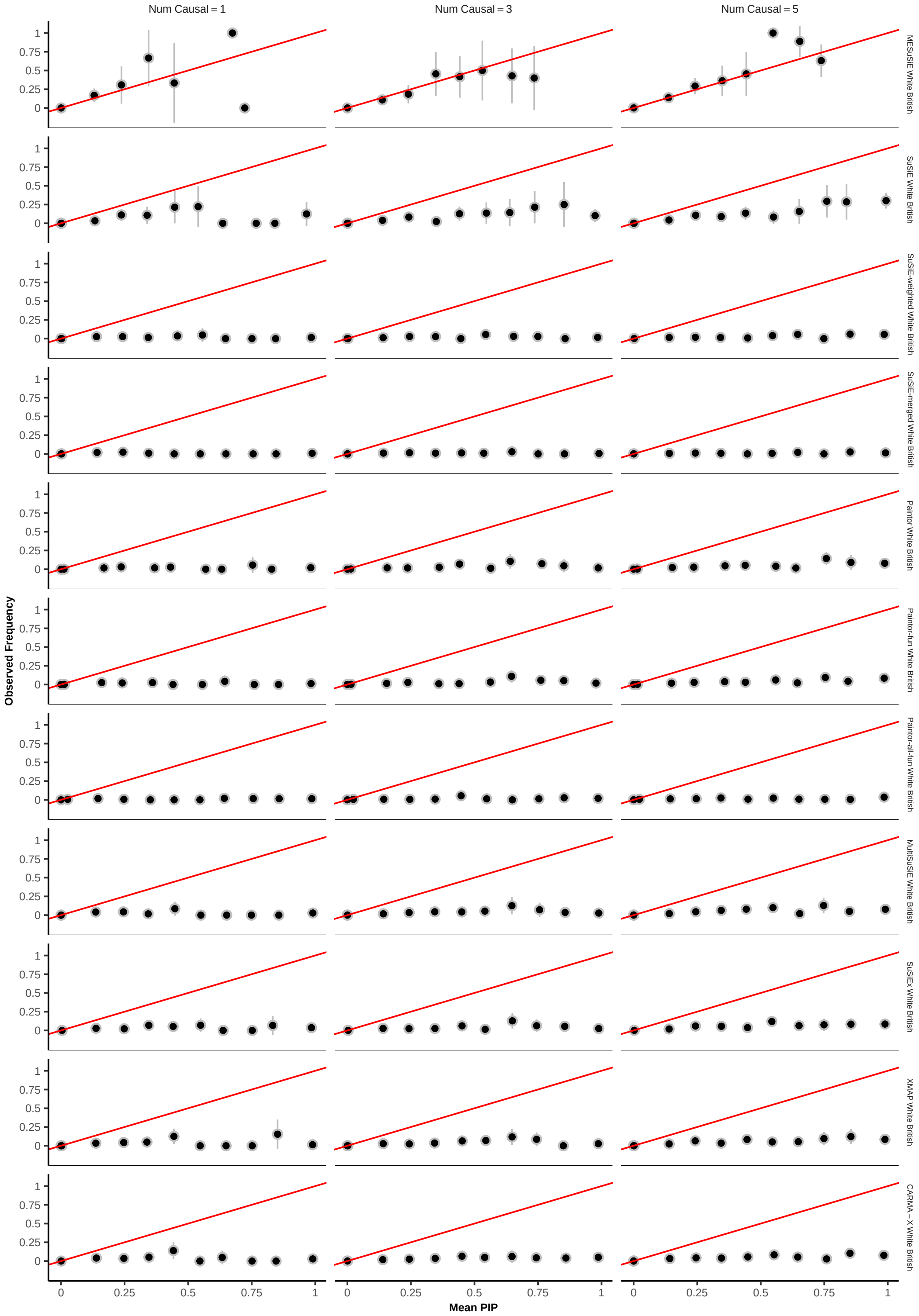

Supplementary Figure 68: **PIP calibration for White British (WB) ancestry (functional-annotation sensitivity, $w = 0$).** Calibration plots aggregating $n = 300$ independent regions (combining all causal SNP scenarios). SNPs are ranked by PIP and divided into 10 equally sized bins. The y-axis shows the observed proportion of causal signals, and the x-axis shows the mean PIP within each bin. Gray error bars represent ± 2 standard errors. The red diagonal line indicates perfect calibration; points below the line suggest inflated PIPs (more false discoveries), while points above indicate conservative PIPs.

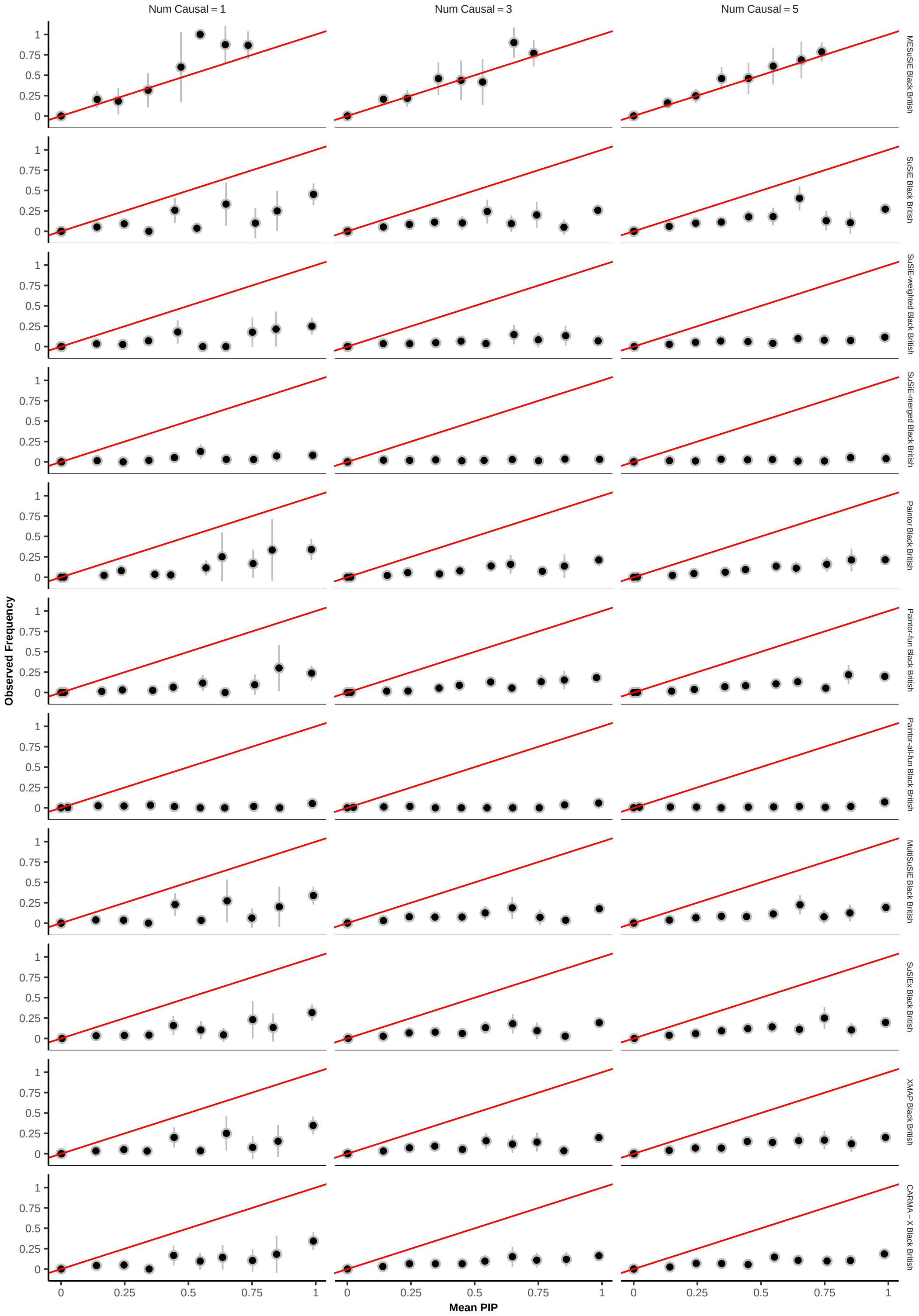

Supplementary Figure 69: **PIP calibration for Black British (BB) ancestry (functional-annotation sensitivity, $w = 0$).** Calibration plots aggregating $n = 300$ independent regions (combining all causal SNP scenarios). SNPs are ranked by PIP and divided into 10 equally sized bins. The y-axis shows the observed proportion of causal signals, and the x-axis shows the mean PIP within each bin. Gray error bars represent ± 2 standard errors. The red diagonal line indicates perfect calibration; points below the line suggest inflated PIPs (more false discoveries), while points above indicate conservative PIPs.

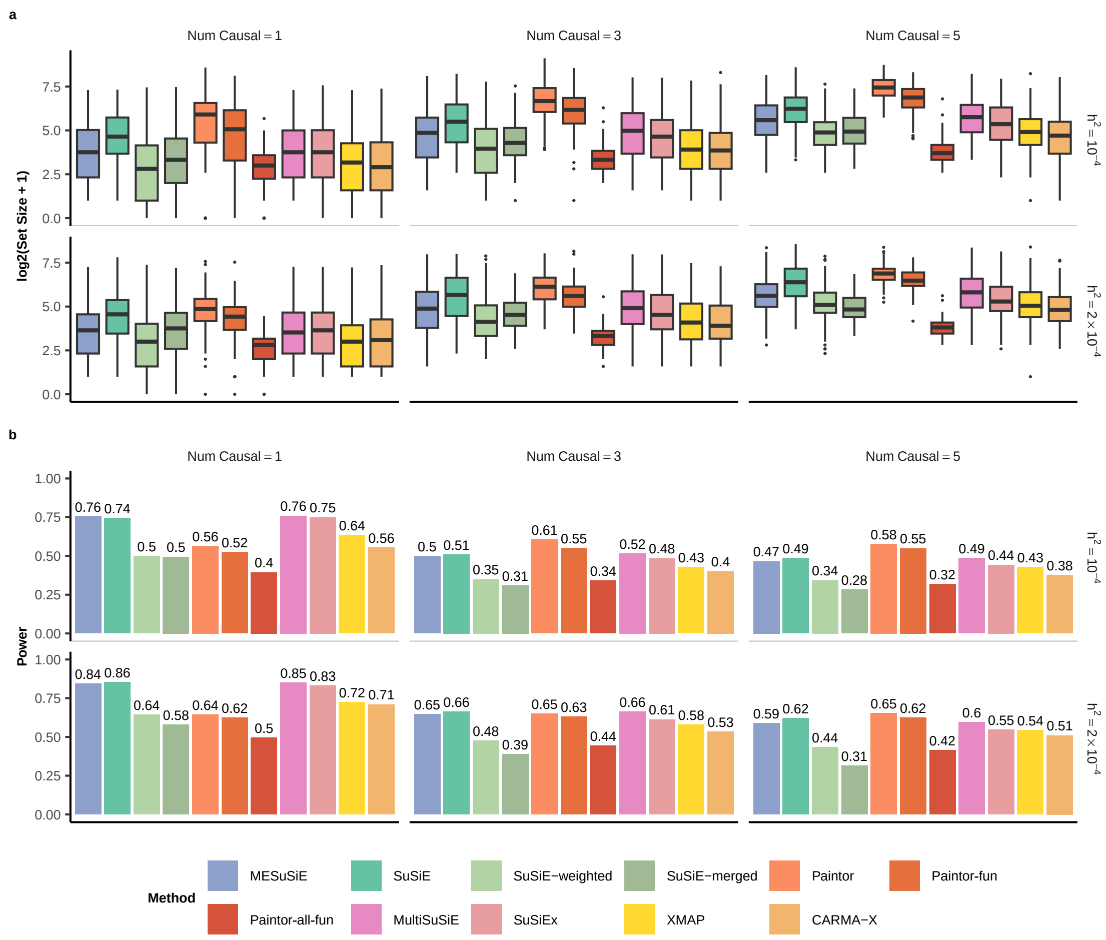

Supplementary Figure 70: **Performance metrics based on credible sets (Functional annotation sensitivity, $w = 1$, moderate enrichment).** Comparison of 95% credible set sizes and power in detecting causal signals with non-zero effects in at least one ancestry under the functional annotation simulation with enrichment weight $w = 1$ (moderate enrichment of causal variants among annotated SNPs). Results are summarized across $n = 100$ independent regions, stratified by the number of causal SNPs (1, 3, or 5 cross-ancestry, corresponding to a total of 2, 6, and 10 causal SNPs, respectively) and per-causal SNP heritability ($h^2 = 10^{-4}$ or 2×10^{-4}; rows). **(a)** Boxplots displaying the $\log_2(\text{set size} + 1)$ of the 95% credible sets. **(b)** Bar plots displaying the power of the constructed 95% credible sets, defined as the proportion of simulated causal variants successfully included in the credible set.

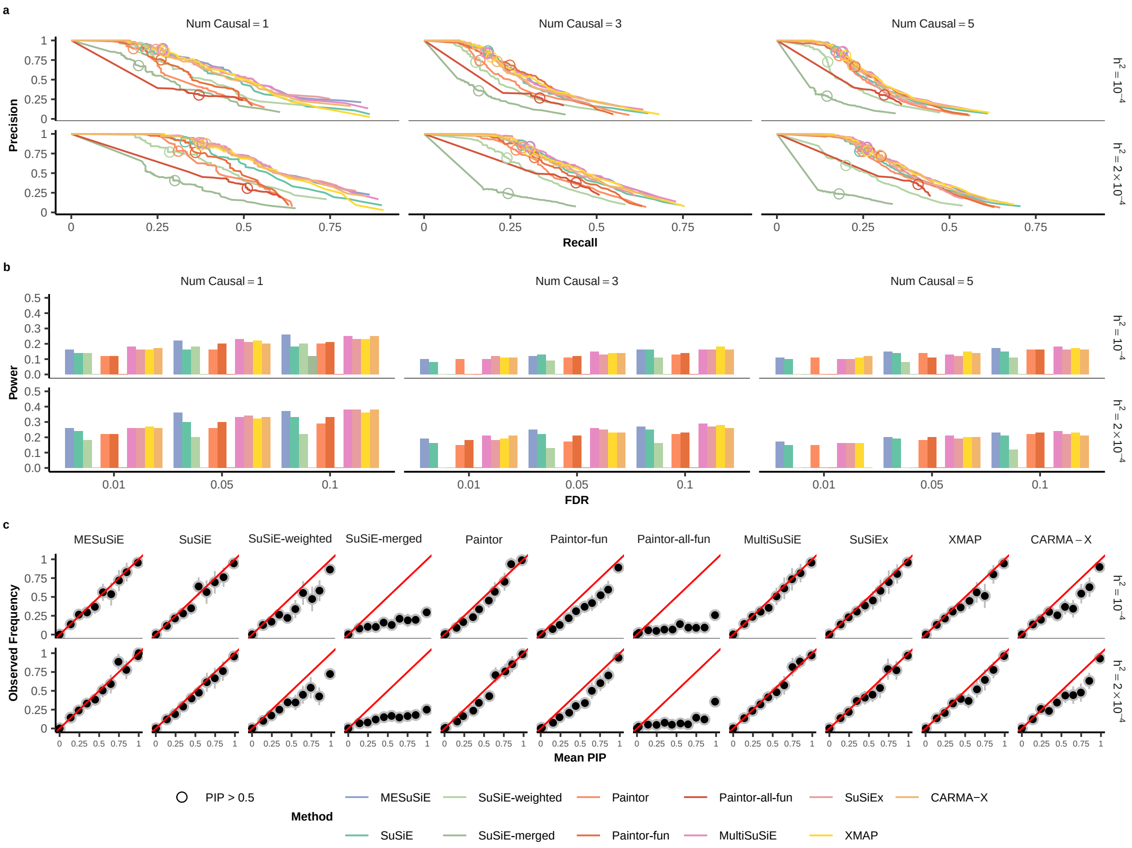

Supplementary Figure 71: **Comparison of method performance in detecting causal signals with non-zero effects in at least one ancestry (Functional annotation sensitivity, $w = 1$, 'Either').** Results under moderate enrichment ($w = 1$) are stratified by the number of cross-ancestry causal SNPs (1, 3, or 5, corresponding to a total of 2, 6, and 10 causal SNPs, respectively) and per-causal SNP heritability ($h^2 = 10^{-4}$ or 2×10^{-4}; rows). **(a)** Precision-recall curves with PIP_{either} thresholds ranging from 0.01 to 1. Precision is defined as $1 - \text{FDR}$ and Recall as $\frac{\text{TP}}{\text{TP+FN}}$, where TP, FP, and FN represent true positives, false positives, and false negatives, respectively. The PIP threshold of 0.5 is marked with a circle. **(b)** Bar plots displaying power at controlled FDR levels of 0.01, 0.05, and 0.1. **(c)** Calibration plots aggregating $n = 300$ independent regions (combining all causal SNP scenarios). SNPs are ranked by PIP and divided into 10 equally sized bins. The y-axis shows the observed proportion of causal signals, and the x-axis shows the mean PIP within each bin. Gray error bars represent ± 2 standard errors. The red diagonal line indicates perfect calibration; points below the line suggest inflated PIPs (more false discoveries), while points above indicate conservative PIPs.

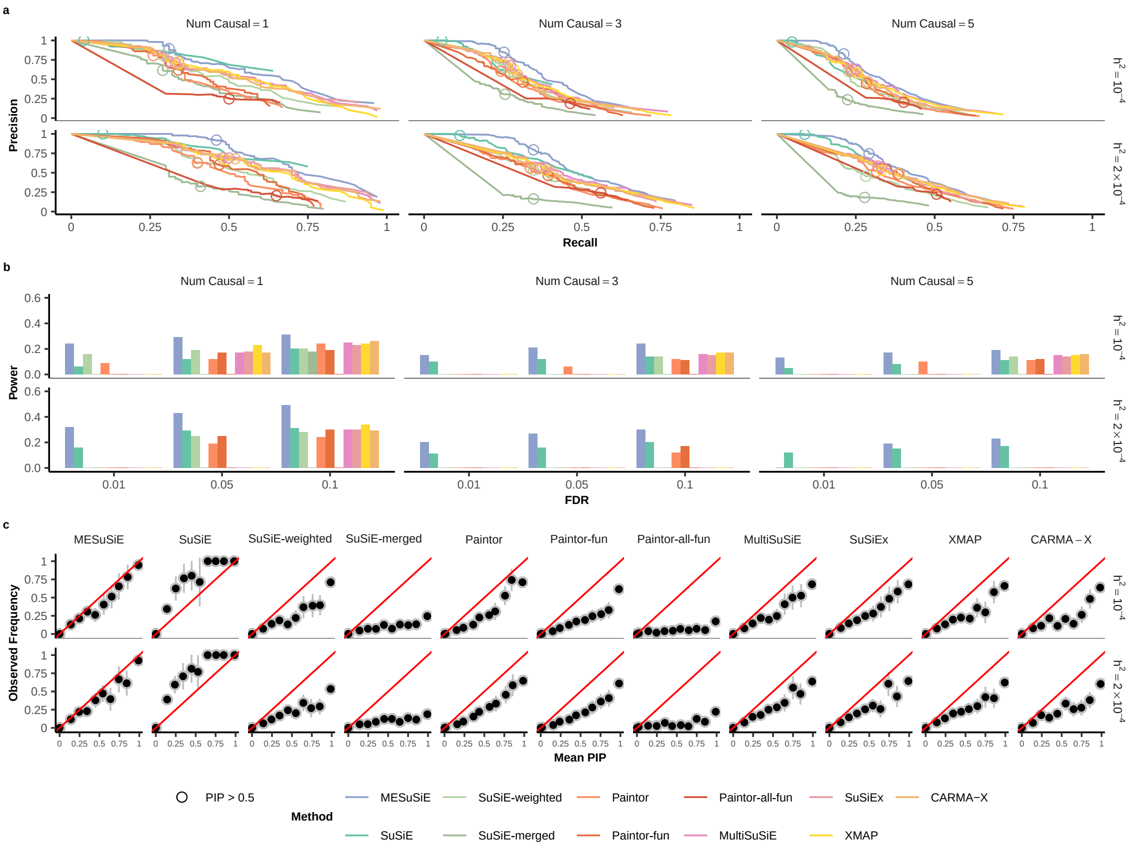

Supplementary Figure 72: **Comparison of method performance in detecting causal signals with non-zero effects in both ancestries (Functional annotation sensitivity, $w = 1$, 'Cross-ancestry').** Results under moderate enrichment ($w = 1$) are stratified by the number of cross-ancestry causal SNPs (1, 3, or 5, corresponding to a total of 2, 6, and 10 causal SNPs, respectively) and per-causal SNP heritability ($h^2 = 10^{-4}$ or 2×10^{-4}; rows). **(a)** Precision-recall curves with PIP_{shared} thresholds ranging from 0.01 to 1. Precision is defined as $1 - \text{FDR}$ and Recall as $\frac{\text{TP}}{\text{TP+FN}}$, where TP, FP, and FN represent true positives, false positives, and false negatives, respectively. The PIP threshold of 0.5 is marked with a circle. **(b)** Bar plots displaying power at controlled FDR levels of 0.01, 0.05, and 0.1. **(c)** Calibration plots aggregating $n = 300$ independent regions (combining all causal SNP scenarios). SNPs are ranked by PIP and divided into 10 equally sized bins. The y-axis shows the observed proportion of causal signals, and the x-axis shows the mean PIP within each bin. Gray error bars represent ± 2 standard errors. The red diagonal line indicates perfect calibration; points below the line suggest inflated PIPs (more false discoveries), while points above indicate conservative PIPs.

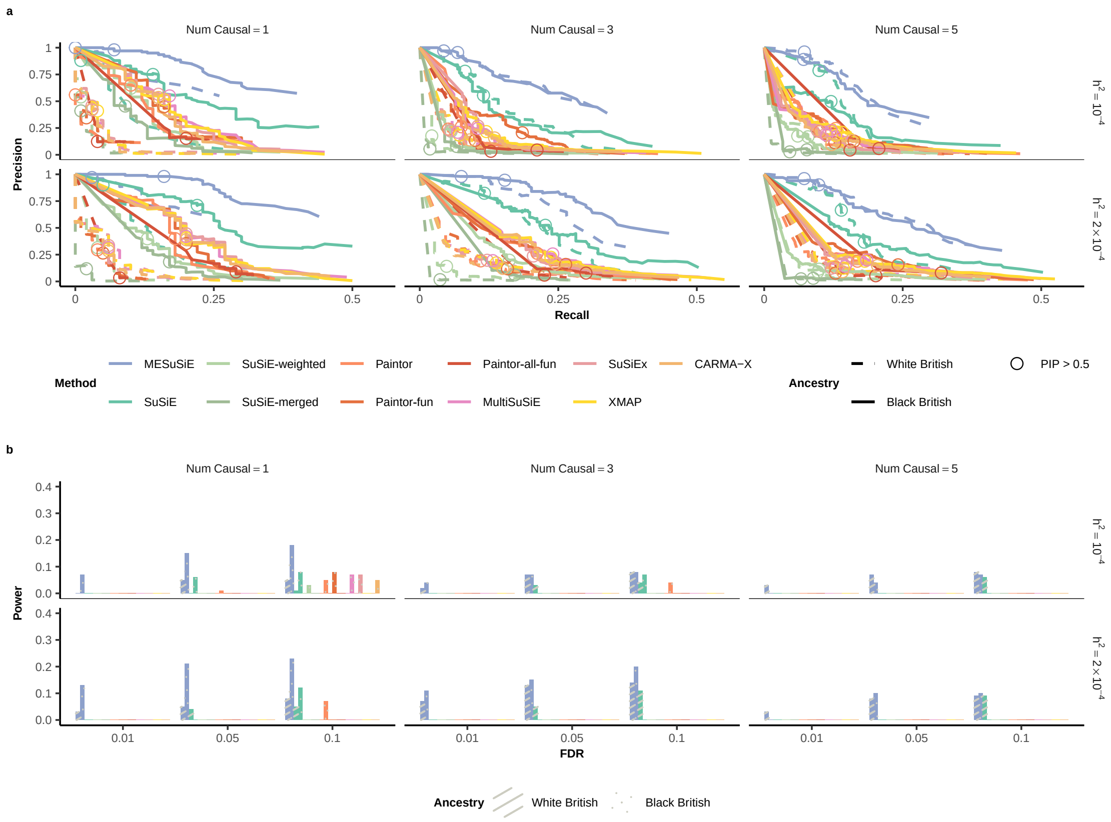

Supplementary Figure 73: **Performance metrics for ancestry-specific contextual causal signals (Functional annotation sensitivity, $w = 1$).** Comparison of method performance in detecting causal signals with non-zero effects in White British only and Black British only under moderate enrichment ($w = 1$). Results are stratified by the number of cross-ancestry causal SNPs (1, 3, or 5, corresponding to a total of 2, 6, and 10 causal SNPs, respectively) and per-causal SNP heritability ($h^2 = 10^{-4}$ or 2×10^{-4}; rows). **(a)** Precision-recall curves with PIP_{WB} and PIP_{BB} thresholds ranging from 0.01 to 1. Precision is defined as $1 - \text{FDR}$ and Recall as $\frac{\text{TP}}{\text{TP+FN}}$, where TP, FP, and FN represent true positives, false positives, and false negatives, respectively. The PIP threshold of 0.5 is marked with a circle. **(b)** Bar plots displaying power at controlled FDR levels of 0.01, 0.05, and 0.1.

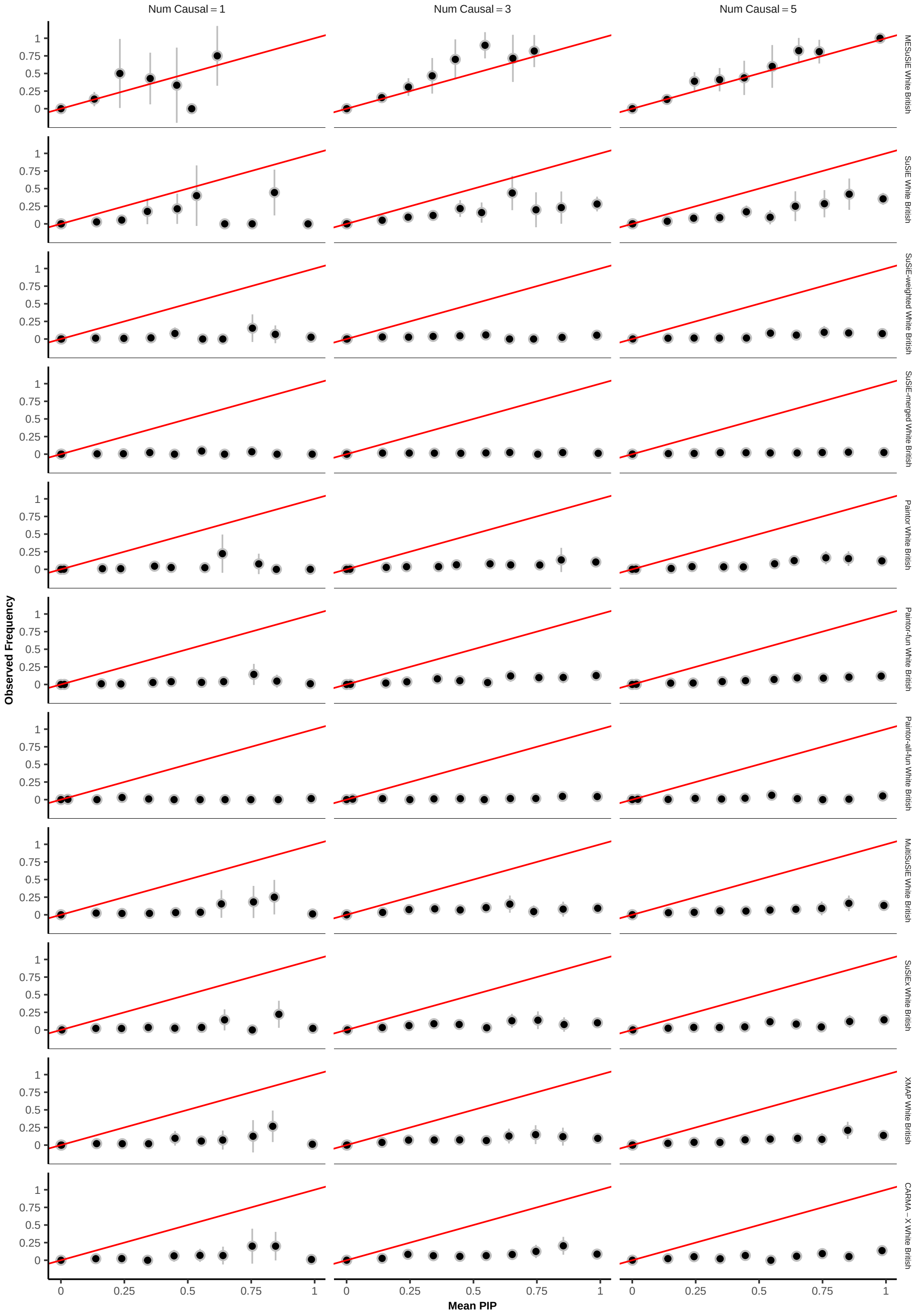

Supplementary Figure 74: **PIP calibration for White British (WB) ancestry (functional-annotation sensitivity, $w = 1$, moderate enrichment).** Calibration plots aggregating $n = 300$ independent regions (combining all causal SNP scenarios). SNPs are ranked by PIP and divided into 10 equally sized bins. The y-axis shows the observed proportion of causal signals, and the x-axis shows the mean PIP within each bin. Gray error bars represent ± 2 standard errors. The red diagonal line indicates perfect calibration; points below the line suggest inflated PIPs (more false discoveries), while points above indicate conservative PIPs.

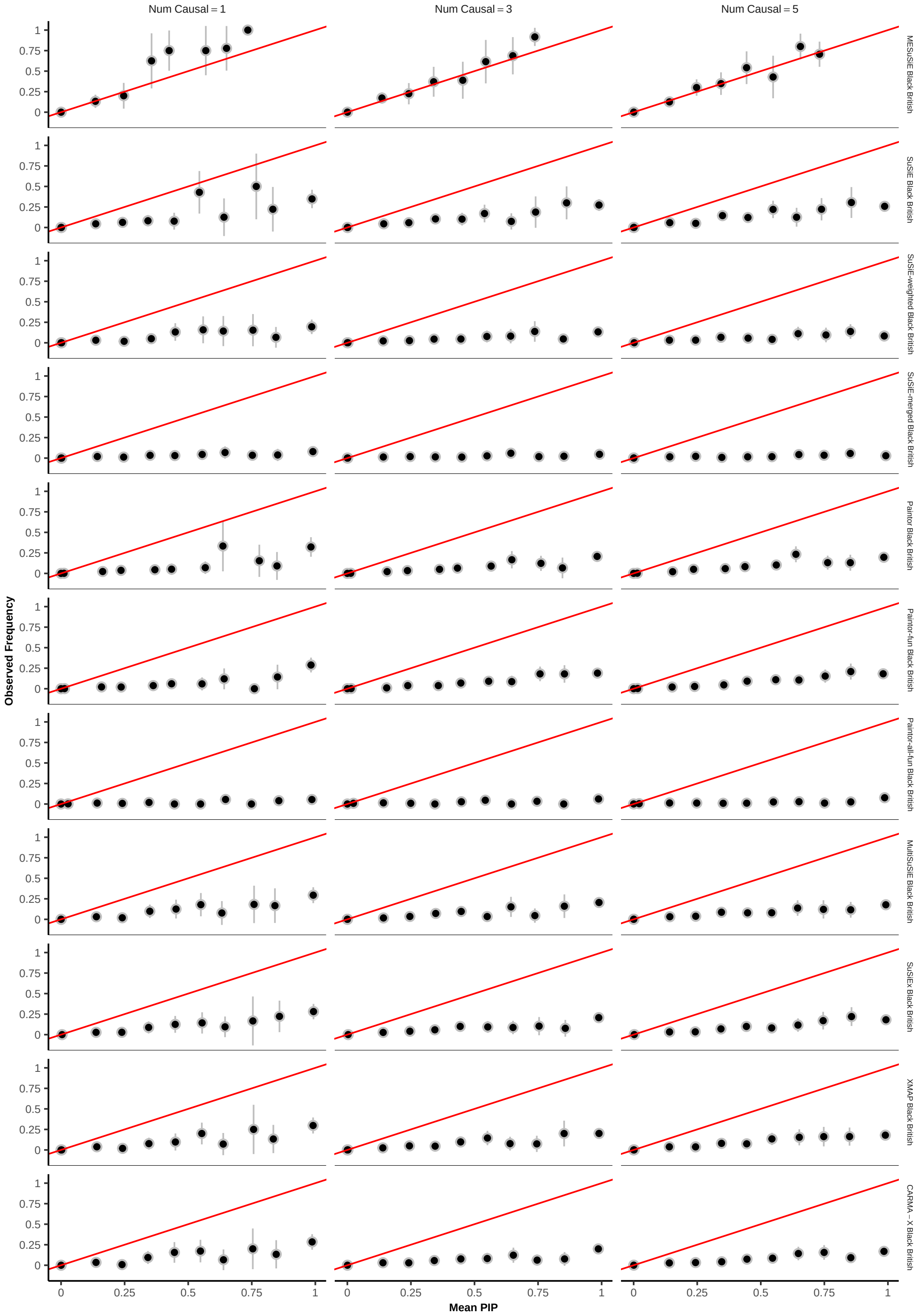

Supplementary Figure 75: **PIP calibration for Black British (BB) ancestry (functional-annotation sensitivity, $w = 1$, moderate enrichment).** Calibration plots aggregating $n = 300$ independent regions (combining all causal SNP scenarios). SNPs are ranked by PIP and divided into 10 equally sized bins. The y-axis shows the observed proportion of causal signals, and the x-axis shows the mean PIP within each bin. Gray error bars represent ± 2 standard errors. The red diagonal line indicates perfect calibration; points below the line suggest inflated PIPs (more false discoveries), while points above indicate conservative PIPs.

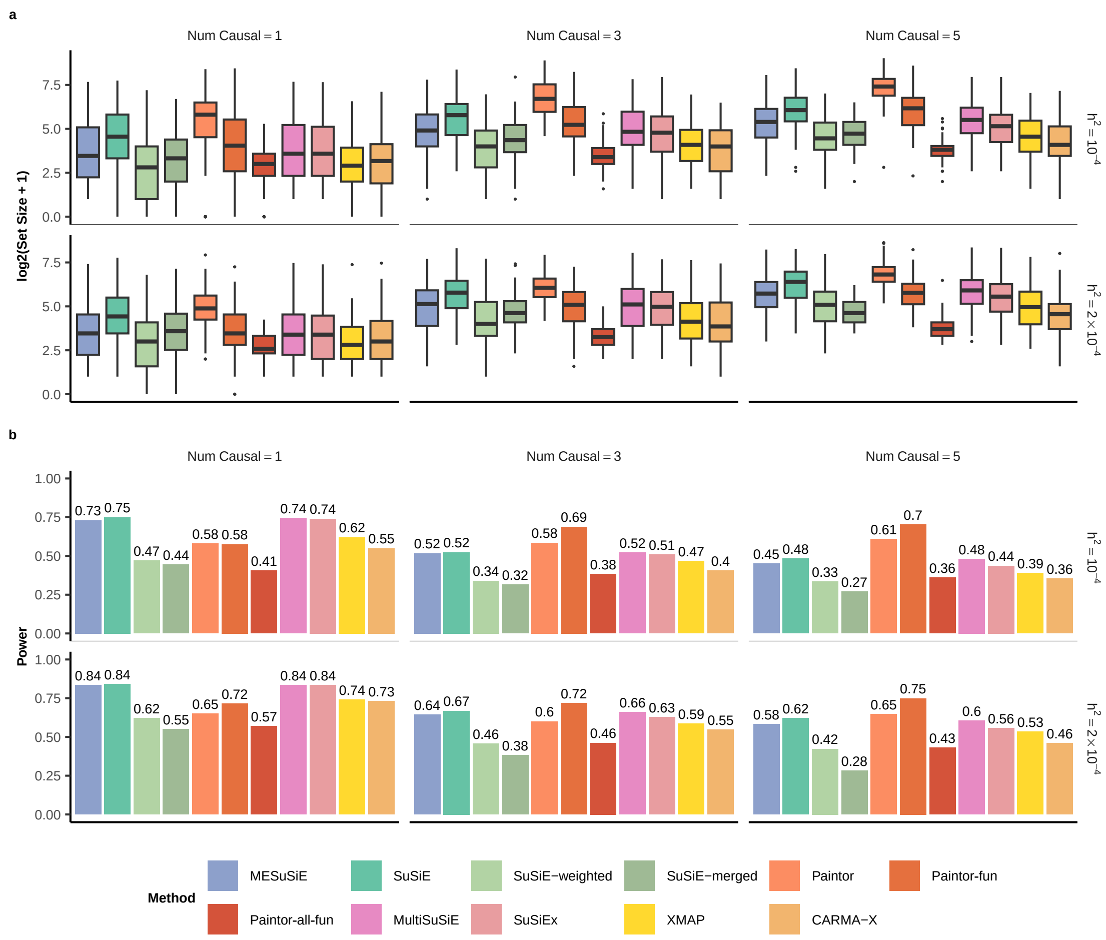

Supplementary Figure 76: **Performance metrics based on credible sets (Functional annotation sensitivity, $w = 2$, strong enrichment).** Comparison of 95% credible set sizes and power in detecting causal signals with non-zero effects in at least one ancestry under the functional annotation simulation with enrichment weight $w = 2$ (strong enrichment of causal variants among annotated SNPs). Results are summarized across $n = 100$ independent regions, stratified by the number of causal SNPs (1, 3, or 5 cross-ancestry, corresponding to a total of 2, 6, and 10 causal SNPs, respectively) and per-causal SNP heritability ($h^2 = 10^{-4}$ or 2×10^{-4}; rows). **(a)** Boxplots displaying the $\log_2(\text{set size} + 1)$ of the 95% credible sets. **(b)** Bar plots displaying the power of the constructed 95% credible sets, defined as the proportion of simulated causal variants successfully included in the credible set.

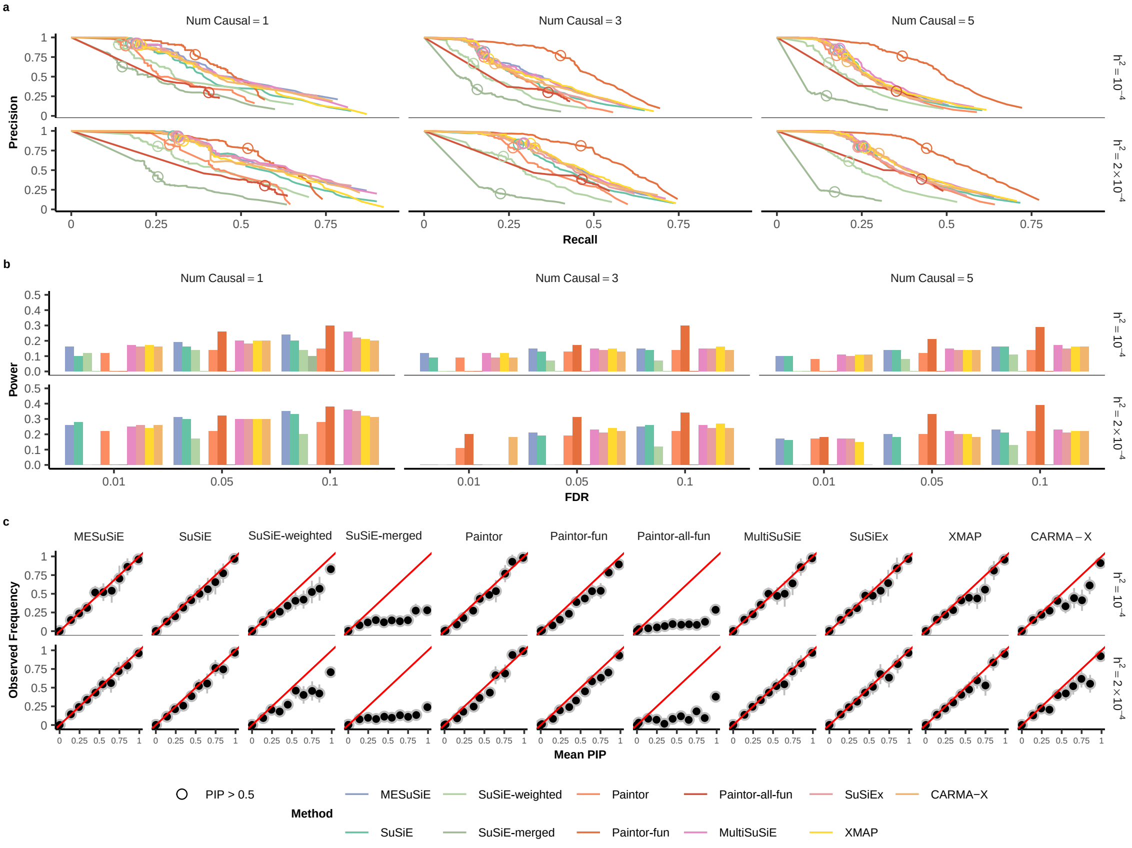

Supplementary Figure 77: **Comparison of method performance in detecting causal signals with non-zero effects in at least one ancestry (Functional annotation sensitivity, $w = 2$, 'Either').** Results under strong enrichment ($w = 2$) are stratified by the number of cross-ancestry causal SNPs (1, 3, or 5, corresponding to a total of 2, 6, and 10 causal SNPs, respectively) and per-causal SNP heritability ($h^2 = 10^{-4}$ or 2×10^{-4}; rows). **(a)** Precision-recall curves with PIP_{either} thresholds ranging from 0.01 to 1. Precision is defined as $1 - \text{FDR}$ and Recall as $\frac{\text{TP}}{\text{TP+FN}}$, where TP, FP, and FN represent true positives, false positives, and false negatives, respectively. The PIP threshold of 0.5 is marked with a circle. **(b)** Bar plots displaying power at controlled FDR levels of 0.01, 0.05, and 0.1. **(c)** Calibration plots aggregating $n = 300$ independent regions (combining all causal SNP scenarios). SNPs are ranked by PIP and divided into 10 equally sized bins. The y-axis shows the observed proportion of causal signals, and the x-axis shows the mean PIP within each bin. Gray error bars represent ± 2 standard errors. The red diagonal line indicates perfect calibration; points below the line suggest inflated PIPs (more false discoveries), while points above indicate conservative PIPs.

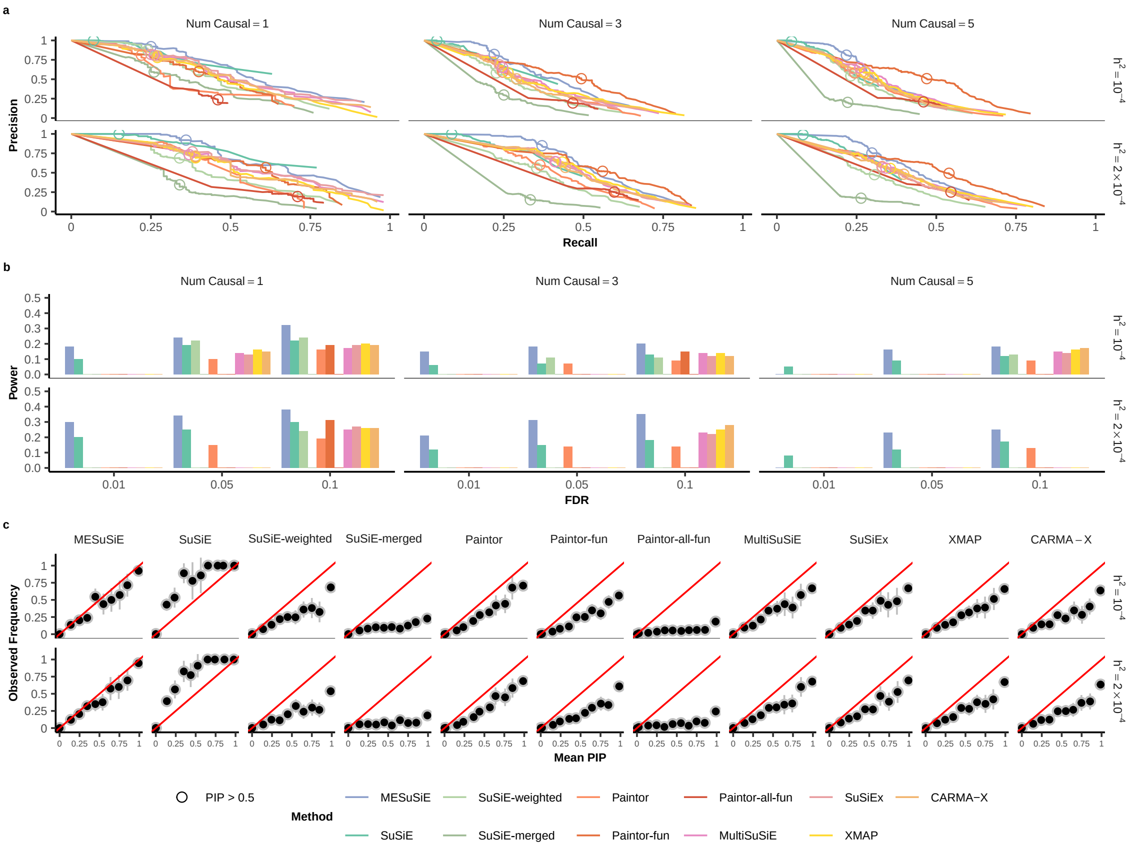

Supplementary Figure 78: **Comparison of method performance in detecting causal signals with non-zero effects in both ancestries (Functional annotation sensitivity, $w = 2$, 'Cross-ancestry').** Results under strong enrichment ($w = 2$) are stratified by the number of cross-ancestry causal SNPs (1, 3, or 5, corresponding to a total of 2, 6, and 10 causal SNPs, respectively) and per-causal SNP heritability ($h^2 = 10^{-4}$ or 2×10^{-4}; rows). **(a)** Precision-recall curves with PIP_{shared} thresholds ranging from 0.01 to 1. Precision is defined as $1 - \text{FDR}$ and Recall as $\frac{\text{TP}}{\text{TP+FN}}$, where TP, FP, and FN represent true positives, false positives, and false negatives, respectively. The PIP threshold of 0.5 is marked with a circle. **(b)** Bar plots displaying power at controlled FDR levels of 0.01, 0.05, and 0.1. **(c)** Calibration plots aggregating $n = 300$ independent regions (combining all causal SNP scenarios). SNPs are ranked by PIP and divided into 10 equally sized bins. The y-axis shows the observed proportion of causal signals, and the x-axis shows the mean PIP within each bin. Gray error bars represent ± 2 standard errors. The red diagonal line indicates perfect calibration; points below the line suggest inflated PIPs (more false discoveries), while points above indicate conservative PIPs.

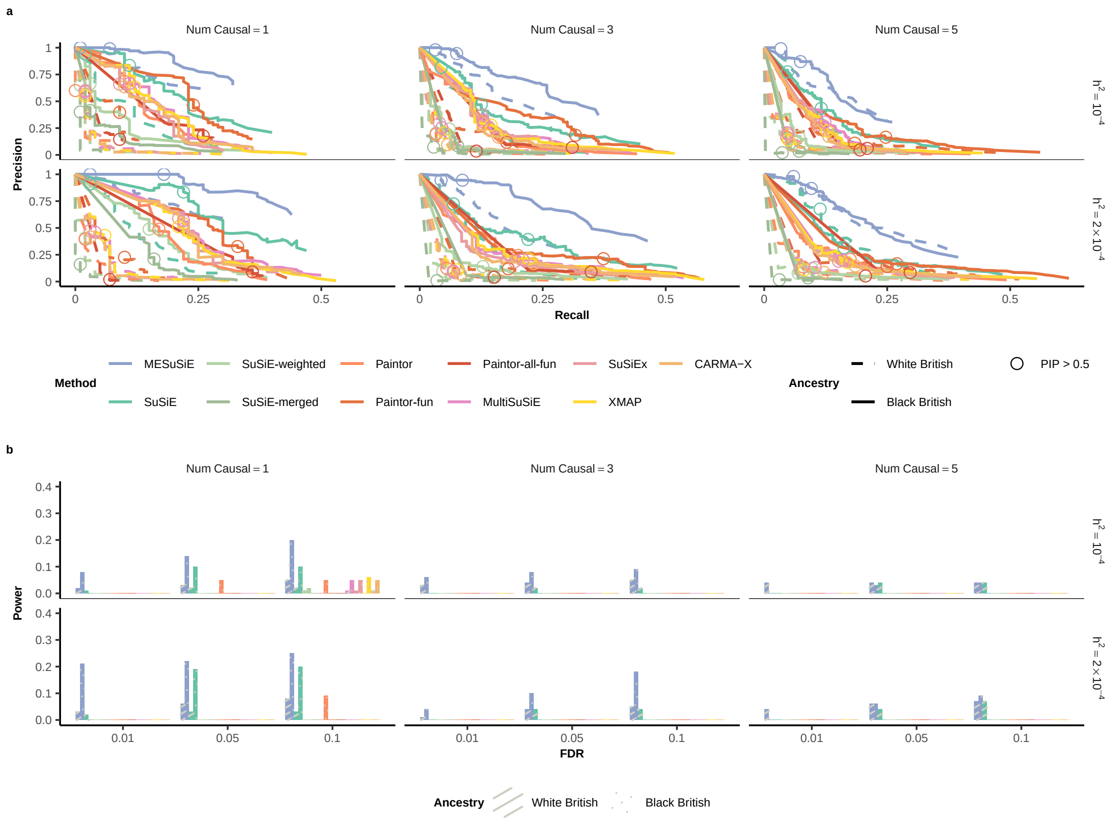

Supplementary Figure 79: **Performance metrics for ancestry-specific contextual causal signals (Functional annotation sensitivity, $w = 2$).** Comparison of method performance in detecting causal signals with non-zero effects in White British only and Black British only under strong enrichment ($w = 2$). Results are stratified by the number of cross-ancestry causal SNPs (1, 3, or 5, corresponding to a total of 2, 6, and 10 causal SNPs, respectively) and per-causal SNP heritability ($h^2 = 10^{-4}$ or 2×10^{-4}; rows). **(a)** Precision-recall curves with PIP_{WB} and PIP_{BB} thresholds ranging from 0.01 to 1. Precision is defined as $1 - \text{FDR}$ and Recall as $\frac{\text{TP}}{\text{TP+FN}}$, where TP, FP, and FN represent true positives, false positives, and false negatives, respectively. The PIP threshold of 0.5 is marked with a circle. **(b)** Bar plots displaying power at controlled FDR levels of 0.01, 0.05, and 0.1.

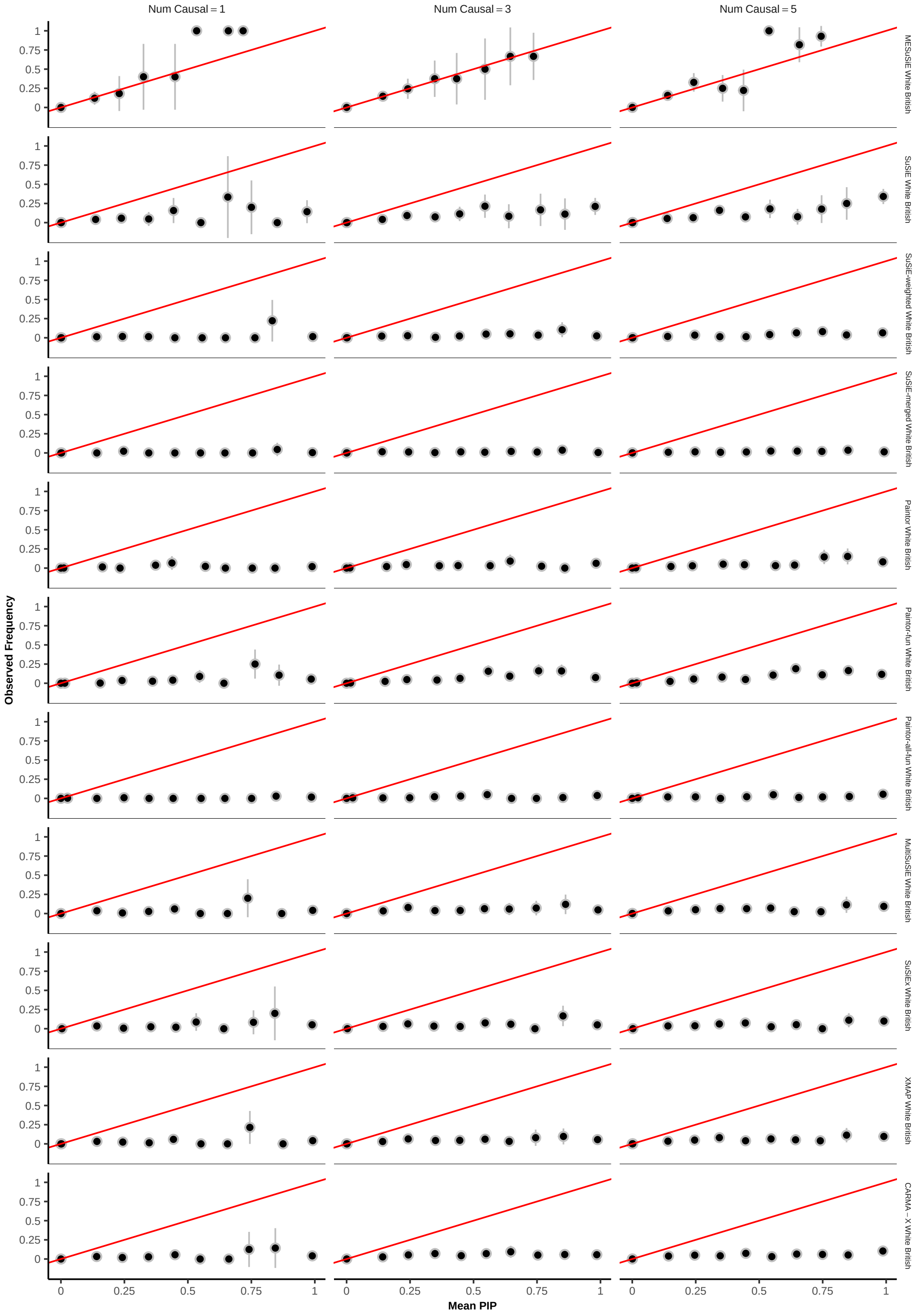

Supplementary Figure 80: **PIP calibration for White British (WB) ancestry (functional-annotation sensitivity, $w = 2$, strong enrichment).** Calibration plots aggregating $n = 300$ independent regions (combining all causal SNP scenarios). SNPs are ranked by PIP and divided into 10 equally sized bins. The y-axis shows the observed proportion of causal signals, and the x-axis shows the mean PIP within each bin. Gray error bars represent ± 2 standard errors. The red diagonal line indicates perfect calibration; points below the line suggest inflated PIPs (more false discoveries), while points above indicate conservative PIPs.

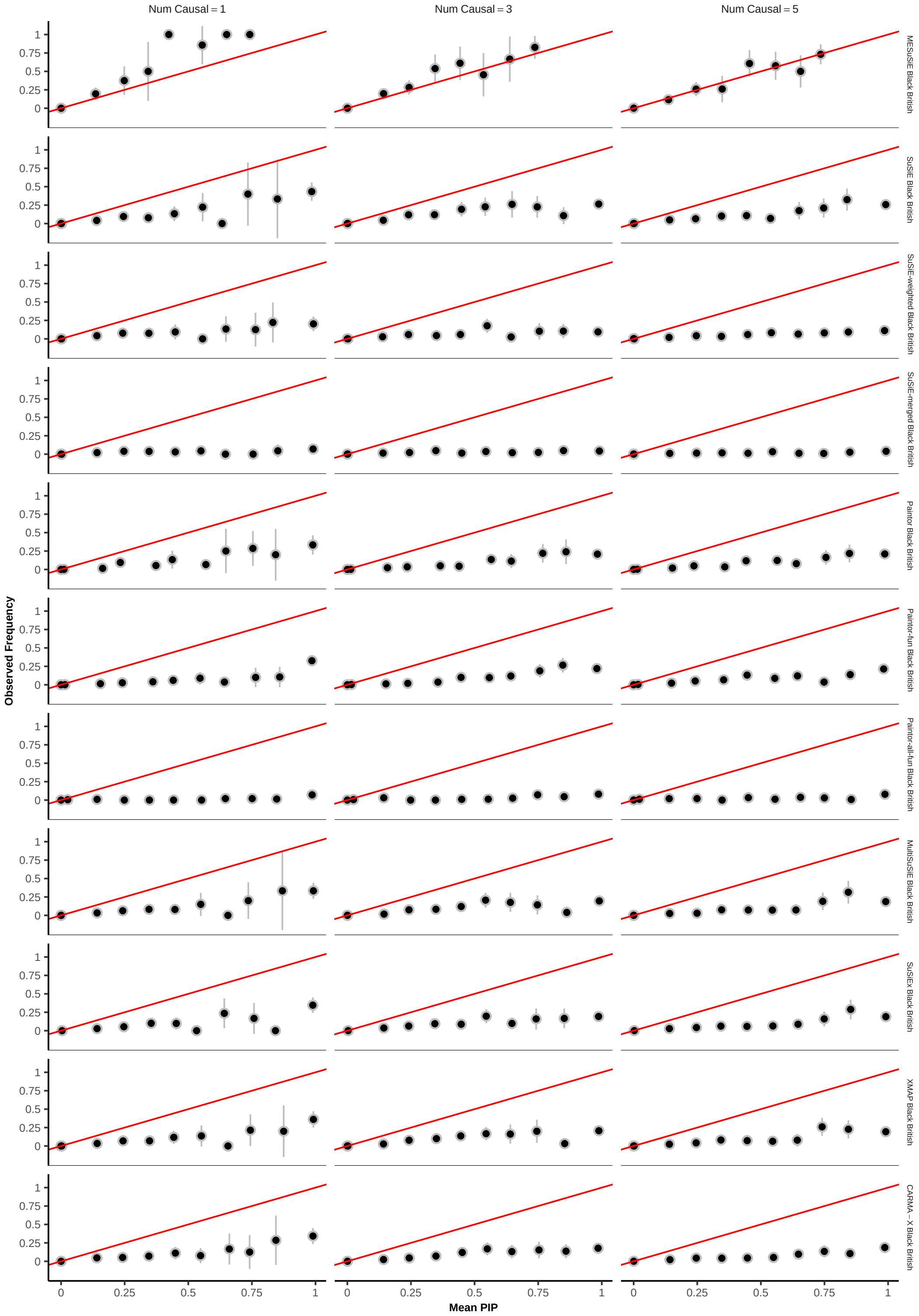

Supplementary Figure 81: **PIP calibration for Black British (BB) ancestry (functional-annotation sensitivity, $w = 2$, strong enrichment).** Calibration plots aggregating $n = 300$ independent regions (combining all causal SNP scenarios). SNPs are ranked by PIP and divided into 10 equally sized bins. The y-axis shows the observed proportion of causal signals, and the x-axis shows the mean PIP within each bin. Gray error bars represent ± 2 standard errors. The red diagonal line indicates perfect calibration; points below the line suggest inflated PIPs le points above indicate conservative PIPs.

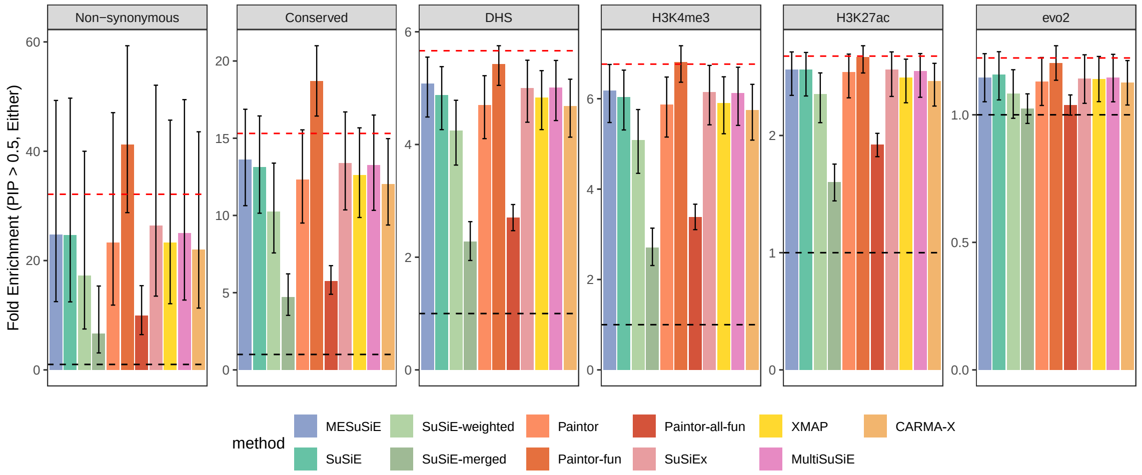

Supplementary Figure 82: **Functional annotation enrichment of fine-mapped variants at PIP > 0.5 (Functional annotation sensitivity, $w = 2$, strong enrichment).** Bar plots showing the fold enrichment of variants with $PIP_{either} > 0.5$ across functional annotation categories under the strong enrichment setting ($w = 2$). Enrichment is calculated as the ratio of the proportion of annotated variants among those with $PIP_{either} > 0.5$ to the proportion of annotated variants in the background set. PAINTOR is evaluated under three annotation prior specifications: no annotations, selected annotations (6 features), and high-dimensional annotations (188 features). The black dashed line indicates an enrichment ratio of 1 (no enrichment), and the red dashed line indicates the true enrichment of each annotation category as determined by the simulation data-generating process. Error bars represent 95% confidence intervals.

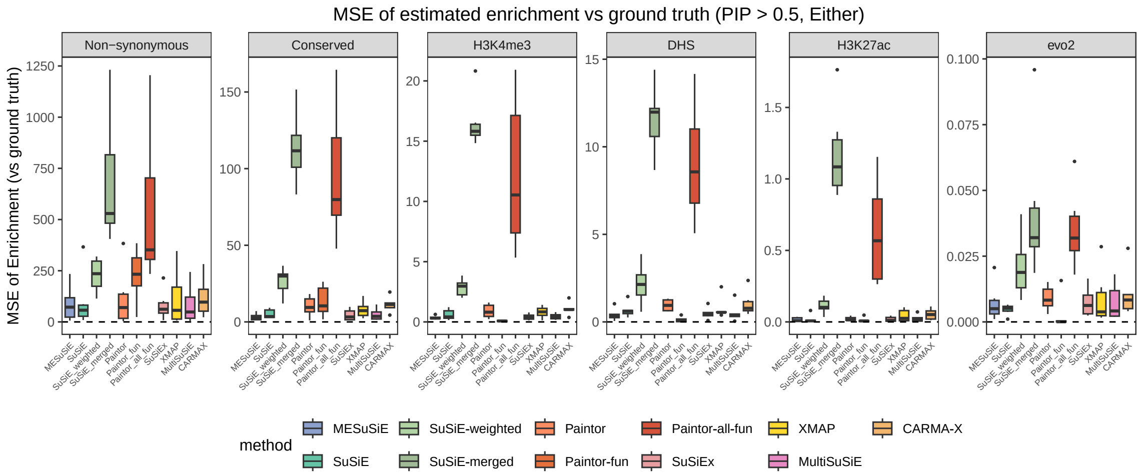

Supplementary Figure 83: **Mean squared error of enrichment estimates (Functional annotation sensitivity, $w = 2$, strong enrichment).** Comparison of enrichment estimation accuracy across methods and PIP thresholds under the strong enrichment setting ($w = 2$). The MSE measures the deviation of estimated functional annotation enrichment from the true enrichment used in the data-generating process.

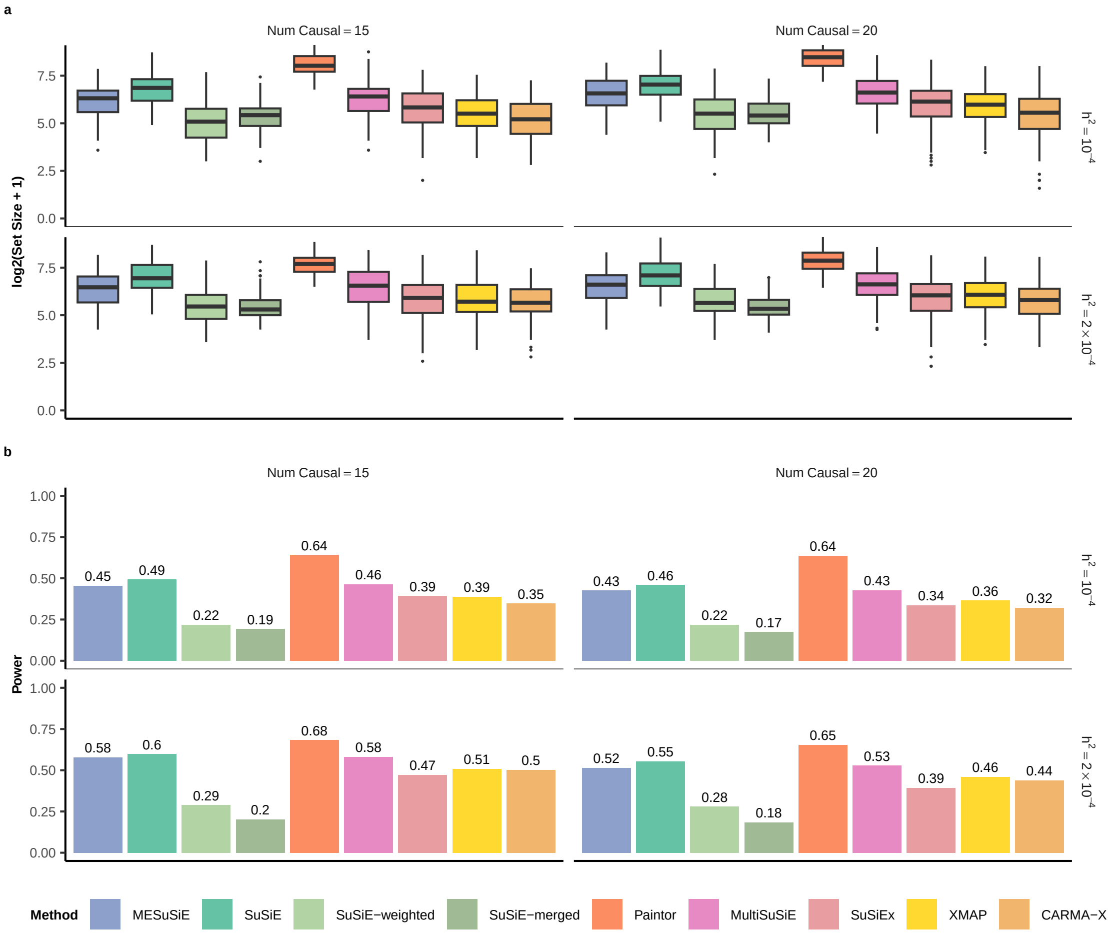

Supplementary Figure 84: **Performance metrics based on credible sets (High allelic heterogeneity simulation).** Comparison of 95% credible set sizes and power in detecting causal signals with non-zero effects in at least one ancestry under high allelic heterogeneity (15 and 20 causal SNPs per 1-Mb region, approximately 50% cross-ancestry across ancestries). Results are summarized across $n = 100$ independent regions, stratified by the number of causal SNPs and per-causal SNP heritability ($h^2 = 10^{-4}$ or 2×10^{-4}; rows). **(a)** Boxplots displaying the $\log_2(\text{set size} + 1)$ of the 95% credible sets. **(b)** Bar plots displaying the power of the constructed 95% credible sets, defined as the proportion of simulated causal variants successfully included in the credible set.

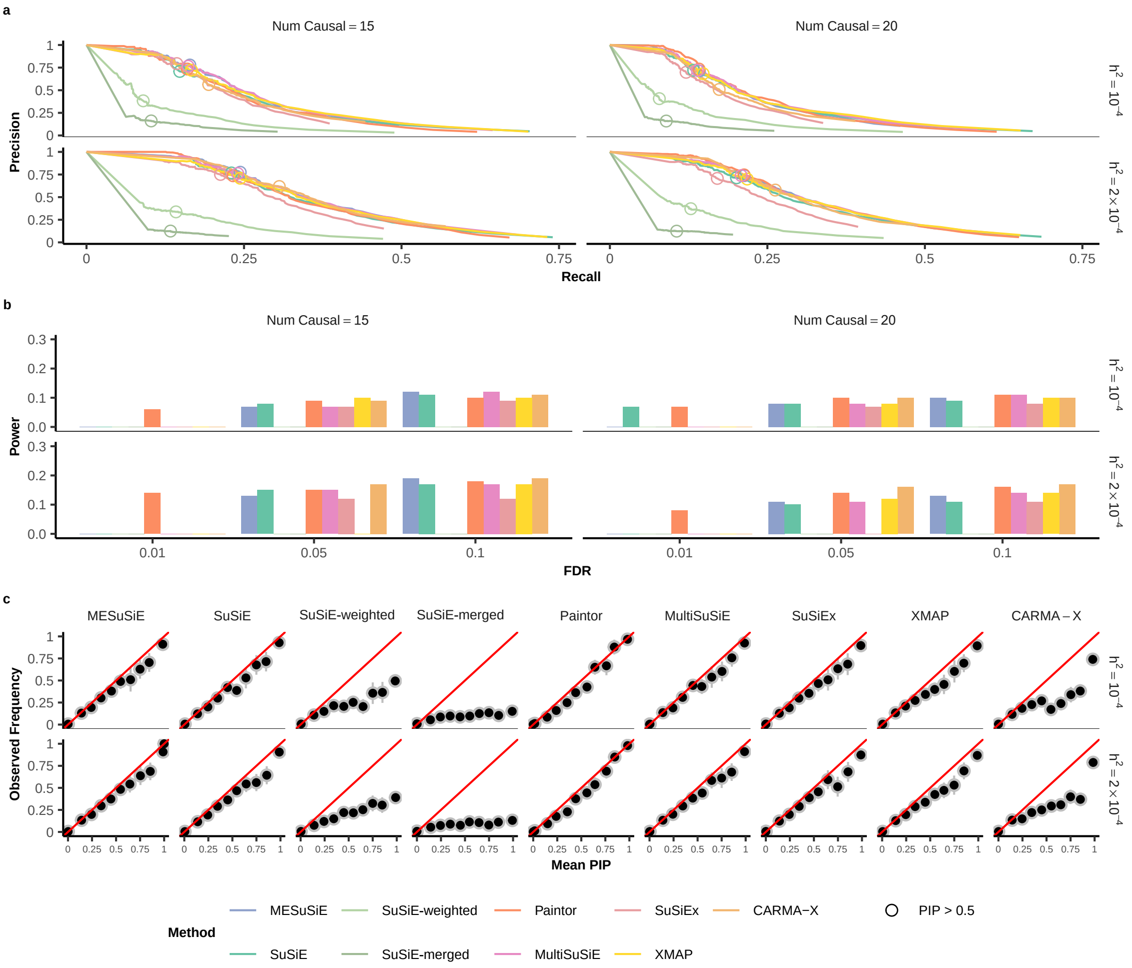

Supplementary Figure 85: **Comparison of method performance in detecting causal signals with non-zero effects in at least one ancestry (High allelic heterogeneity simulation, 'Either').** Results under high allelic heterogeneity (15 and 20 causal SNPs per 1-Mb region, approximately 50% cross-ancestry across ancestries) are stratified by the number of causal SNPs and per-causal SNP heritability ($h^2 = 10^{-4}$ or 2×10^{-4}; rows). **(a)** Precision-recall curves with PIP_{either} thresholds ranging from 0.01 to 1. Precision is defined as 1 − FDR and Recall as $\frac{\text{TP}}{\text{TP+FN}}$, where TP, FP, and FN represent true positives, false positives, and false negatives, respectively. The PIP threshold of 0.5 is marked with a circle. **(b)** Bar plots displaying power at controlled FDR levels of 0.01, 0.05, and 0.1. **(c)** Calibration plots aggregating across independent regions and causal SNP scenarios. SNPs are ranked by PIP and divided into 10 equally sized bins. The y-axis shows the observed proportion of causal signals, and the x-axis shows the mean PIP within each bin. Gray error bars represent ±2 standard errors. The red diagonal line indicates perfect calibration.

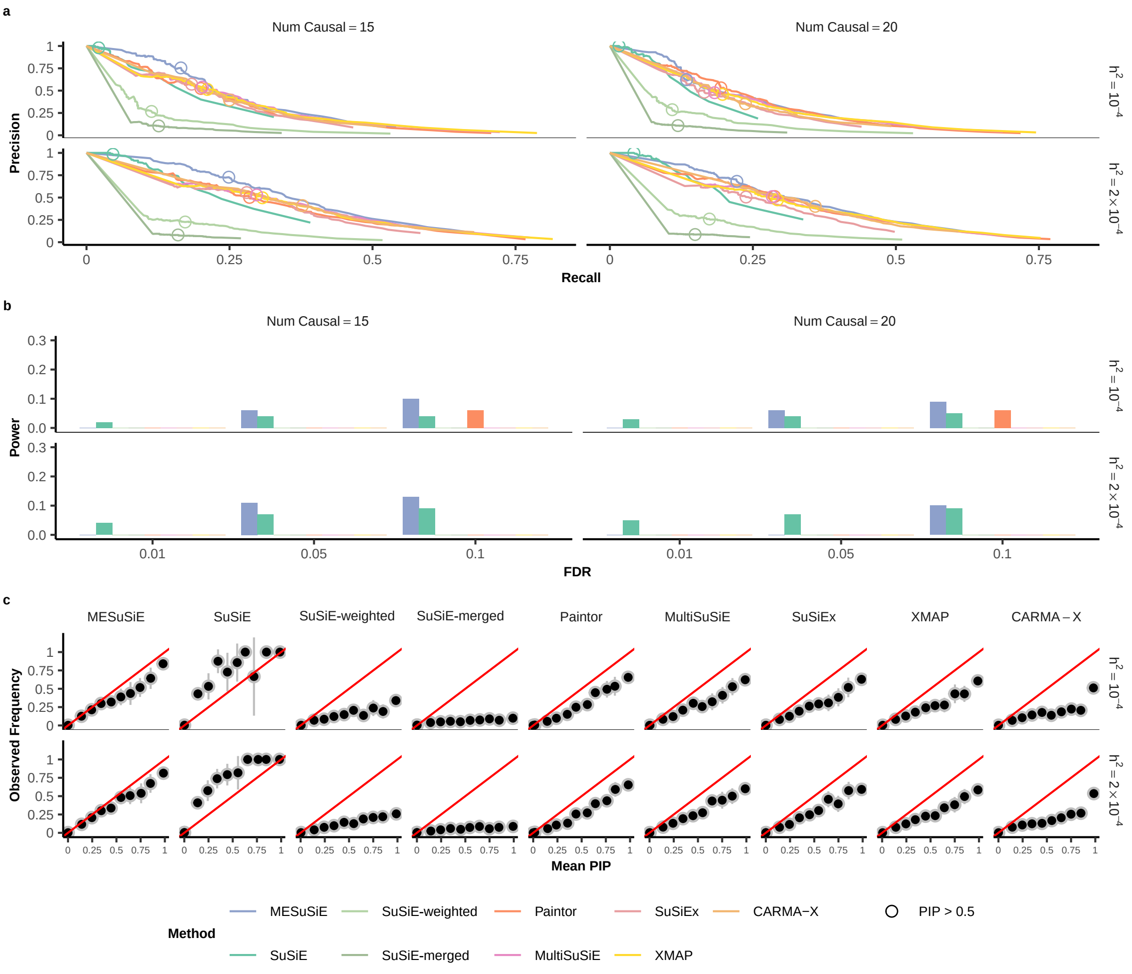

Supplementary Figure 86: **Comparison of method performance in detecting causal signals with non-zero effects in both ancestries (High allelic heterogeneity simulation, 'Cross-ancestry').** Results under high allelic heterogeneity (15 and 20 causal SNPs per 1-Mb region, approximately 50% cross-ancestry across ancestries) are stratified by the number of causal SNPs and per-causal SNP heritability ($h^2 = 10^{-4}$ or 2×10^{-4}; rows). **(a)** Precision-recall curves with PIP_{shared} thresholds ranging from 0.01 to 1. Precision is defined as $1 - \text{FDR}$ and Recall as $\frac{\text{TP}}{\text{TP+FN}}$, where TP, FP, and FN represent true positives, false positives, and false negatives, respectively. The PIP threshold of 0.5 is marked with a circle. **(b)** Bar plots displaying power at controlled FDR levels of 0.01, 0.05, and 0.1. **(c)** Calibration plots aggregating across independent regions and causal SNP scenarios. SNPs are ranked by PIP and divided into 10 equally sized bins. The y-axis shows the observed proportion of causal signals, and the x-axis shows the mean PIP within each bin. Gray error bars represent ±2 standard errors. The red diagonal line indicates perfect calibration.

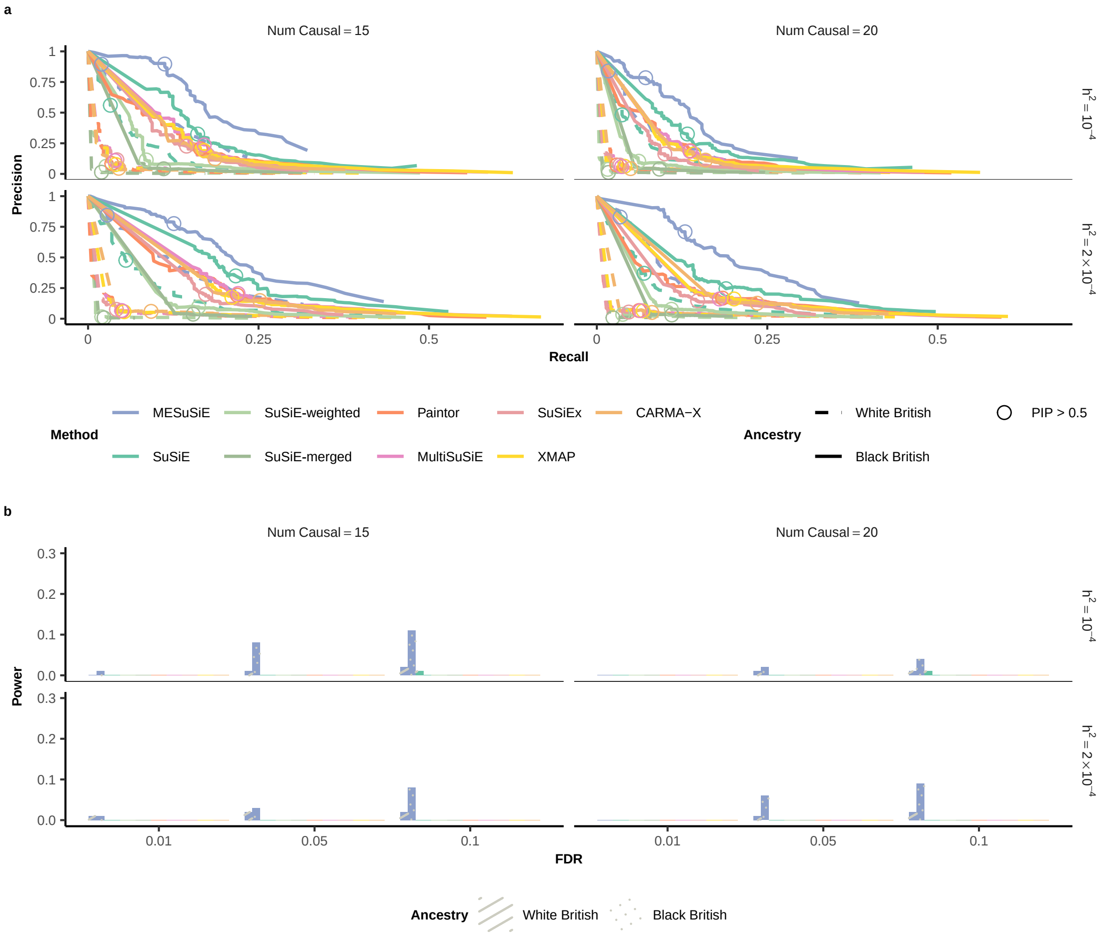

Supplementary Figure 87: **Performance metrics for ancestry-specific contextual causal signals (High allelic heterogeneity simulation).** Comparison of method performance in detecting causal signals with non-zero effects in White British only and Black British only under high allelic heterogeneity (15 and 20 causal SNPs per region). Results are stratified by the number of causal SNPs and per-causal SNP heritability. **(a)** Precision-recall curves with PIP_{WB} and PIP_{BB} thresholds ranging from 0.01 to 1. Precision is defined as $1 - \text{FDR}$ and Recall as $\frac{\text{TP}}{\text{TP+FN}}$, where TP, FP, and FN represent true positives, false positives, and false negatives, respectively. The PIP threshold of 0.5 is marked with a circle. **(b)** Bar plots displaying power at controlled FDR levels of 0.01, 0.05, and 0.1.

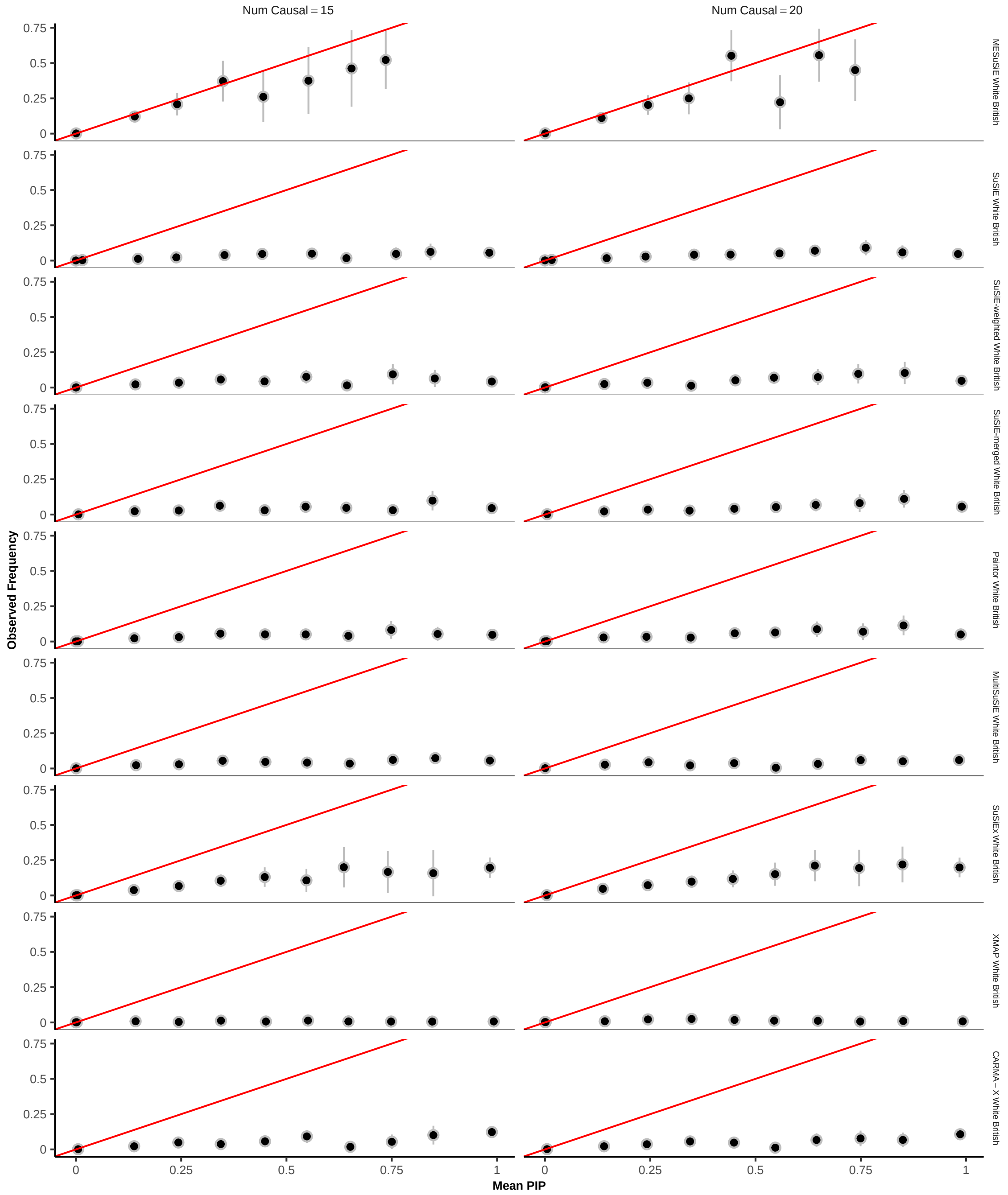

Supplementary Figure 88: **PIP Calibration for White British (WB) Ancestry (High allelic heterogeneity simulation).** Calibration plots under high allelic heterogeneity (15 and 20 causal SNPs per 1-Mb region, approximately 50% cross-ancestry across ancestries), aggregating across independent regions and causal SNP scenarios. SNPs are ranked by PIP and divided into 10 equally sized bins. The y-axis shows the observed proportion of causal signals, and the x-axis shows the mean PIP within each bin. Gray error bars represent ± 2 standard errors. The red diagonal line indicates perfect calibration; points below the line suggest inflated PIPs (more false discoveries), while points above indicate conservative PIPs.

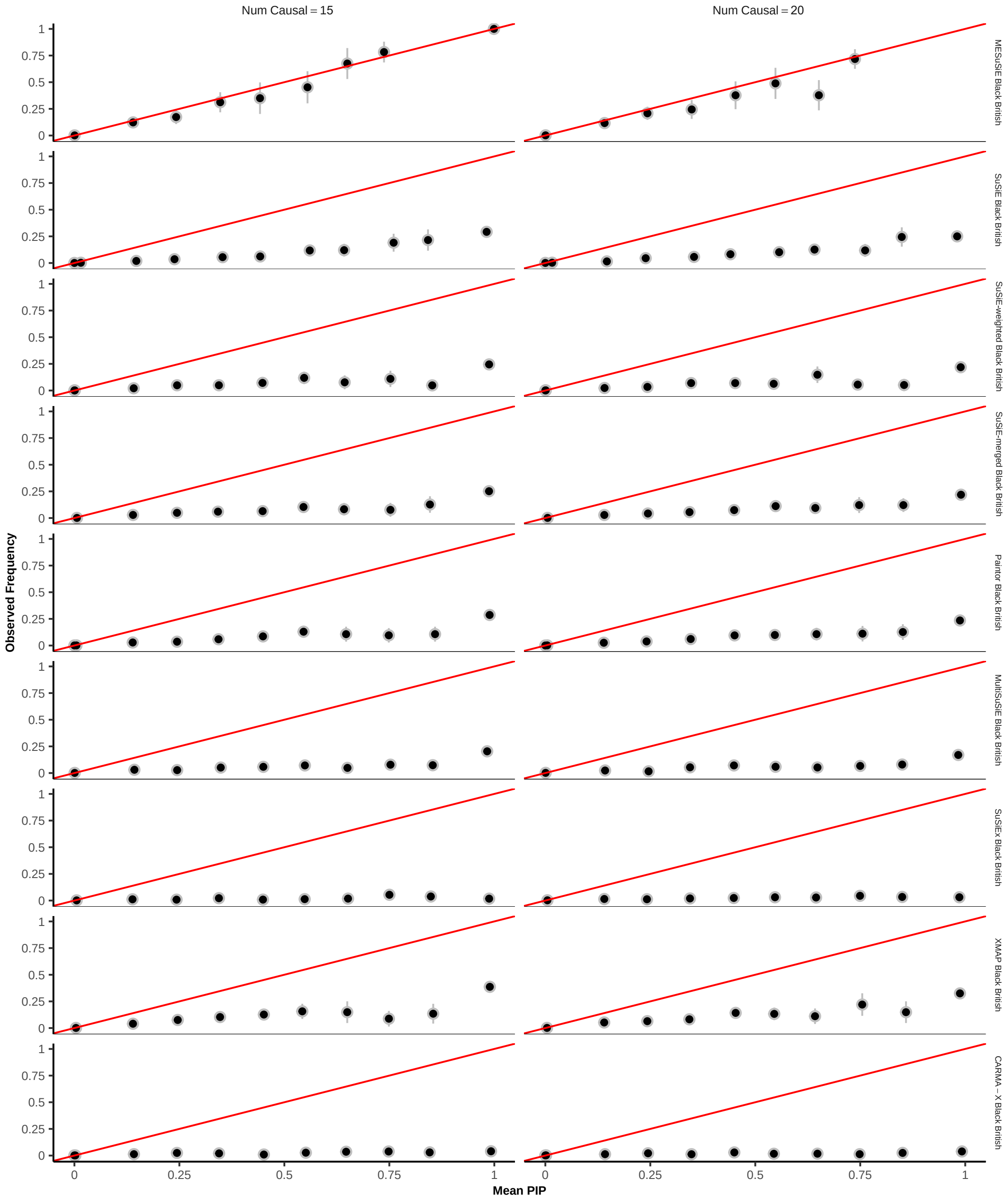

Supplementary Figure 89: **PIP Calibration for Black British (BB) Ancestry (High allelic heterogeneity simulation).** Calibration plots under high allelic heterogeneity (15 and 20 causal SNPs per 1-Mb region, approximately 50% cross-ancestry across ancestries), aggregating across independent regions and causal SNP scenarios. SNPs are ranked by PIP and divided into 10 equally sized bins. The y-axis shows the observed proportion of causal signals, and the x-axis shows the mean PIP within each bin. Gray error bars represent ±2 standard errors. The red diagonal line indicates perfect calibration; points below the line suggest inflated PIPs (more false discoveries), while points above indicate conservative PIPs.

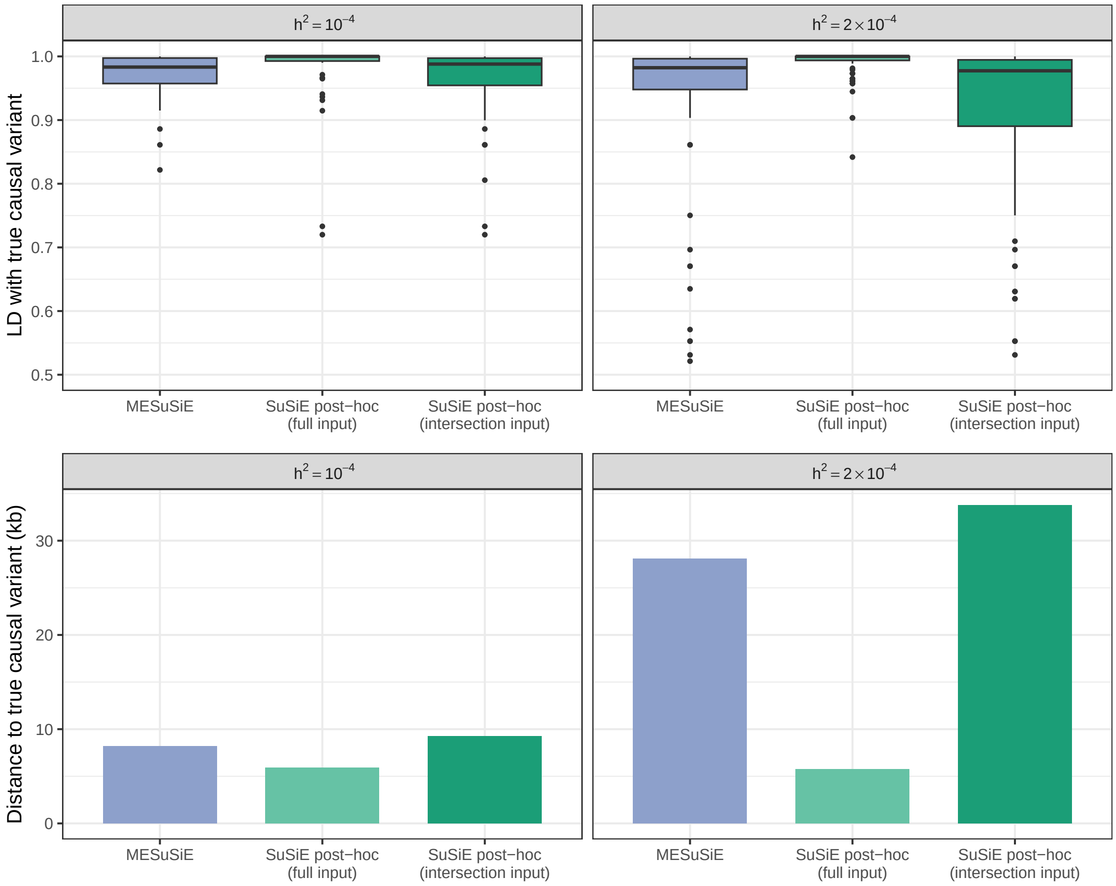

Supplementary Figure 90: **Input-matched sensitivity analysis of post-hoc integration under ancestry-specific causal-variant availability.** The analysis considers the setting in which the causal ancestry-specific segregating variant (AS-SV) is available in one ancestry but excluded from the harmonized candidate-variant set used by joint modeling approach like MESuSiE. SuSiE post-hoc with full input uses all variants available within each ancestry, whereas SuSiE post-hoc with intersection input restricts both ancestry-specific analyses to the same harmonized candidate-variant set used by MESuSiE. Results are summarized across $n = 100$ independent regions and stratified by per-causal SNP heritability ($h^2 = 10^{-4}$ or 2×10^{-4}). **(a)** Boxplots showing the linkage disequilibrium (LD) between the prioritized variant and the true causal variant. **(b)** Bar plots showing the mean genomic distance, in kilobases, between prioritized variants and the true causal variant.

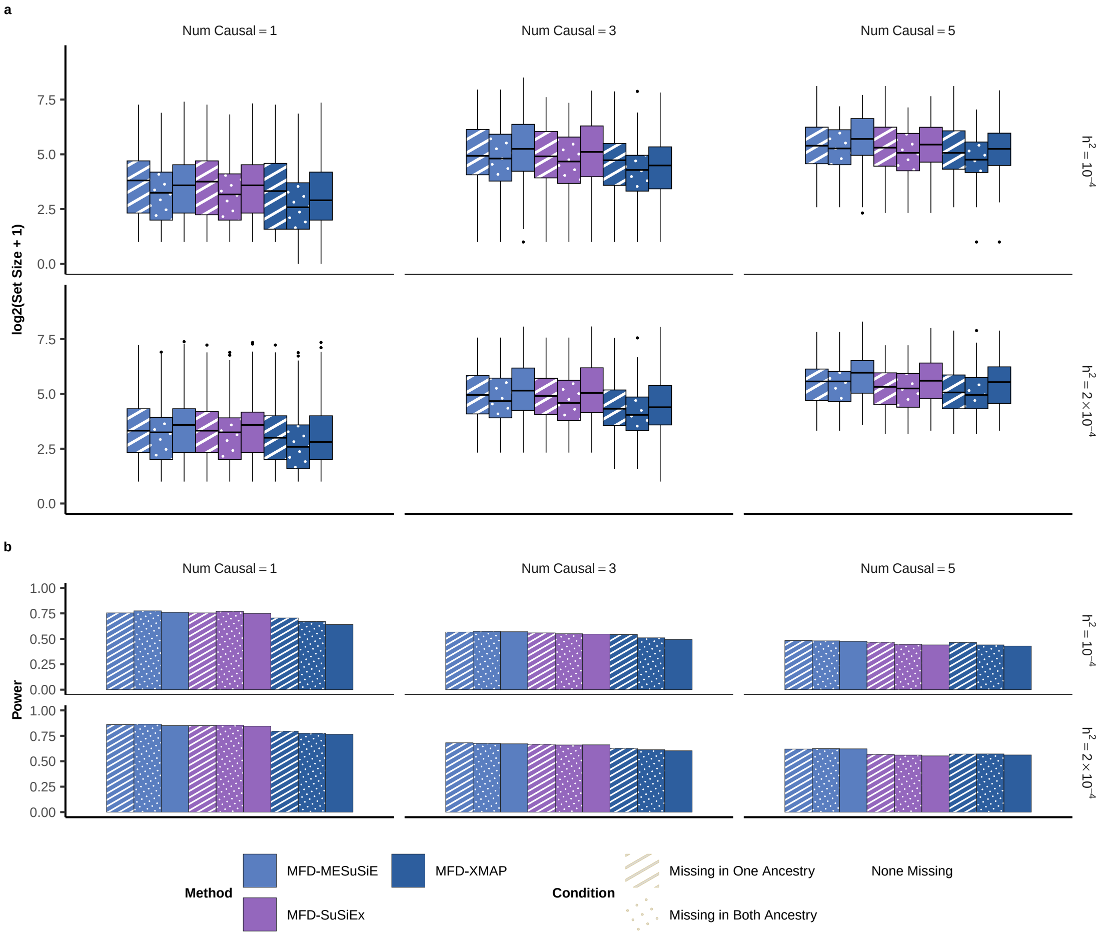

Supplementary Figure 91: **MFD endpoint sensitivity: credible set metrics with non-causal ancestry-specific variants from GWAS summary statistics.** Comparison of 95% credible set sizes and power for MFD with alternative joint endpoints (MFD-MESuSiE, MFD-SuSiEx, MFD-XMAP) when non-causal ancestry-specific variants are missing from GWAS summary statistics in one (stripe) or both (circle) ancestries. Results are summarized across $n = 100$ independent regions, stratified by the number of causal SNPs and per-causal SNP heritability. **(a)** Boxplots displaying the $\log_2(\text{set size}+1)$ of the 95% credible sets. **(b)** Bar plots displaying the power of the constructed 95% credible sets.

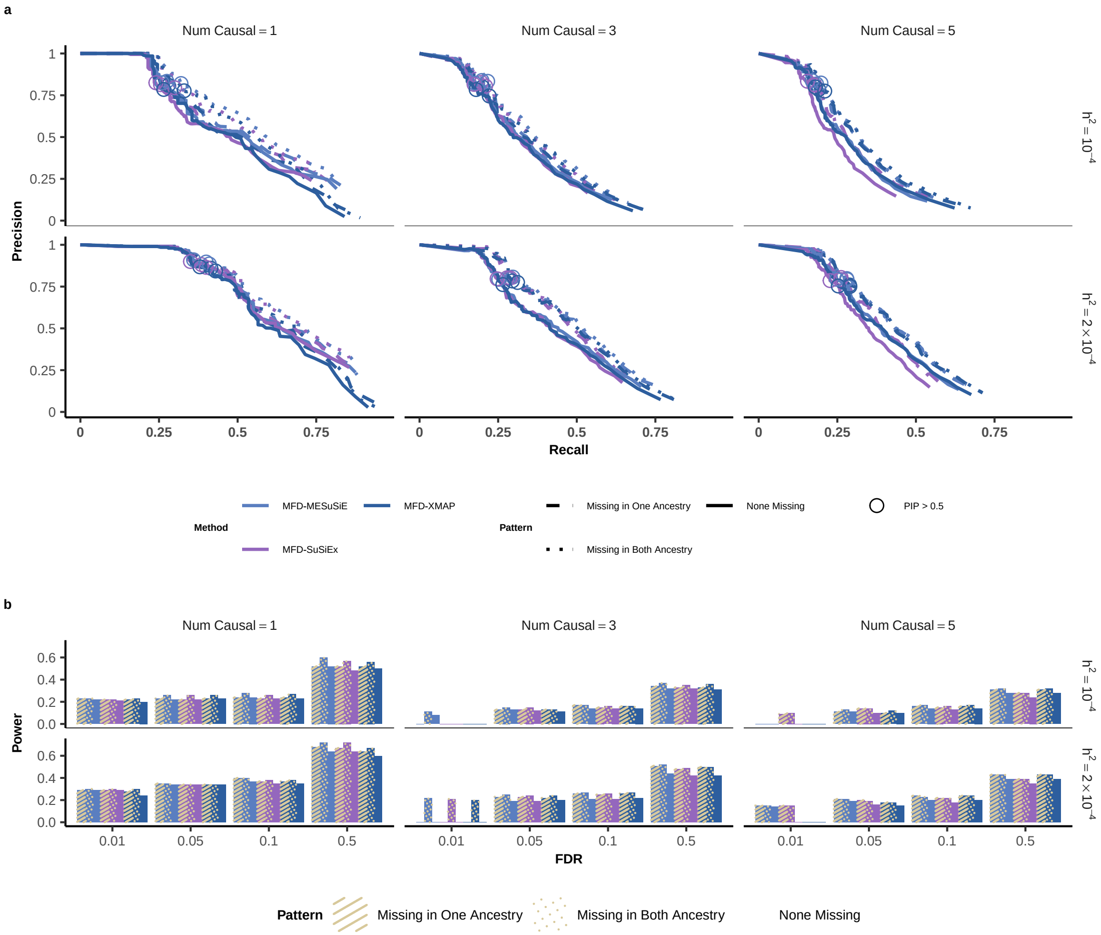

Supplementary Figure 92: **MFD endpoint sensitivity: detecting causal signals in at least one ancestry with non-causal ancestry-specific variants from GWAS summary statistics ('Either').** Comparison of MFD with alternative joint endpoints (MFD-MESuSiE, MFD-SuSiEx, MFD-XMAP) with non-causal ancestry-specific variants from GWAS summary statistics in one (stripe) or both (circle) ancestries. Results are stratified by the number of cross-ancestry causal SNPs (1, 3, or 5, corresponding to a total of 2, 6, and 10 causal SNPs, respectively) and per-causal SNP heritability ($h^2 = 10^{-4}$ or 2×10^{-4}; rows). **(a)** Precision-recall curves with PIP_{either} thresholds ranging from 0.01 to 1. **(b)** Bar plots displaying power at controlled FDR levels of 0.01, 0.05, and 0.1.

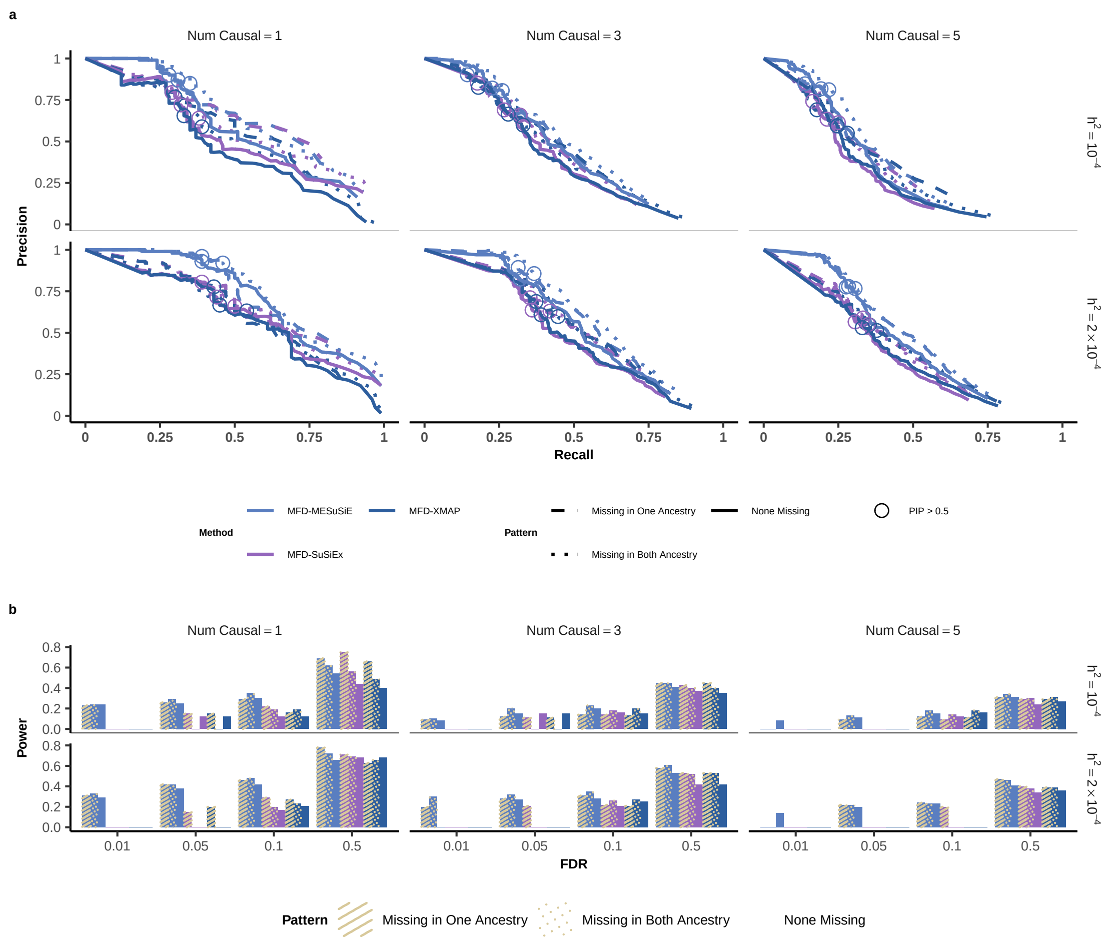

Supplementary Figure 93: **MFD endpoint sensitivity: detecting causal signals in both ancestries with non-causal ancestry-specific variants from GWAS summary statistics ('Cross-ancestry').** Comparison of MFD with alternative joint endpoints (MFD-MESuSiE, MFD-SuSiEx, MFD-XMAP) with non-causal ancestry-specific variants from GWAS summary statistics in one (stripe) or both (circle) ancestries. Results are stratified by the number of cross-ancestry causal SNPs (1, 3, or 5, corresponding to a total of 2, 6, and 10 causal SNPs, respectively) and per-causal SNP heritability ($h^2 = 10^{-4}$ or 2×10^{-4}; rows). **(a)** Precision-recall curves with PIP_{shared} thresholds ranging from 0.01 to 1. **(b)** Bar plots displaying power at controlled FDR levels of 0.01, 0.05, and 0.1.

a

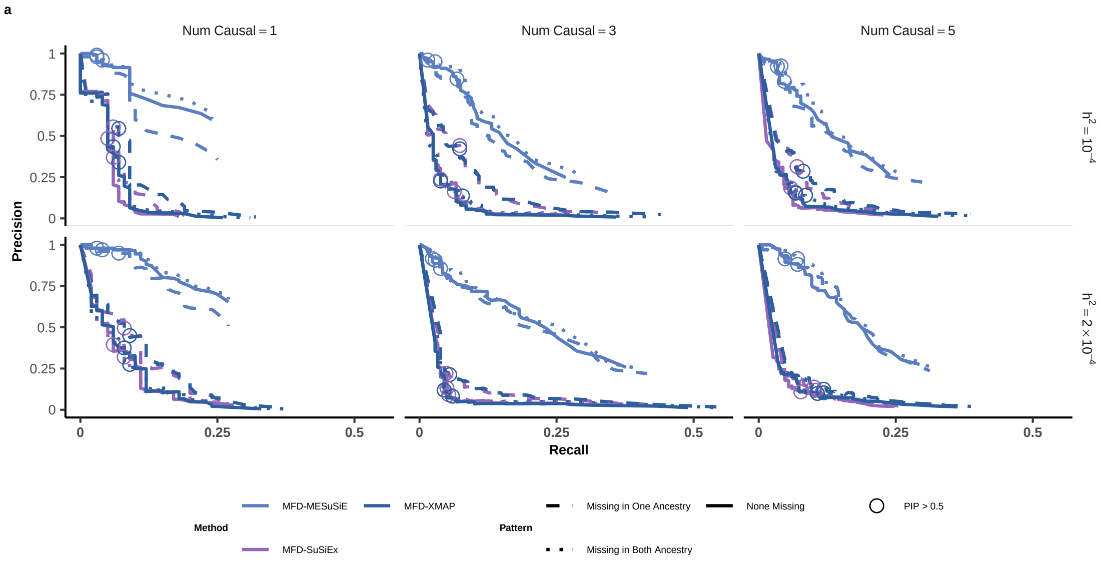

b

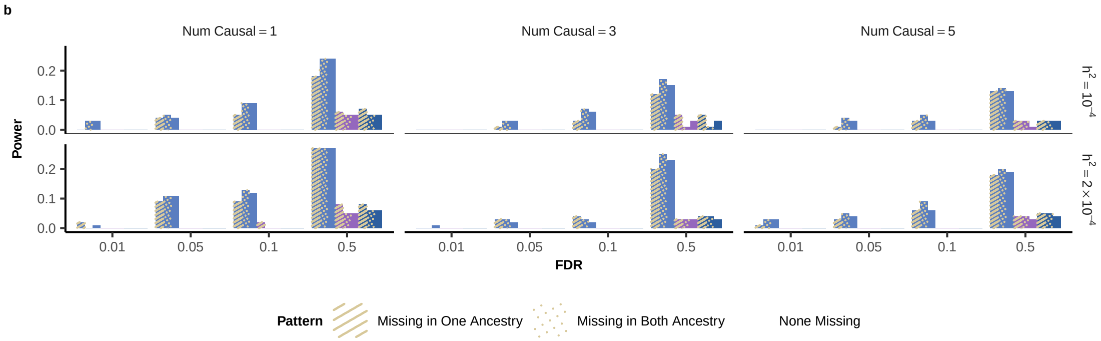

Supplementary Figure 94: **MFD endpoint sensitivity: WB contextual causal signals with non-causal ancestry-specific variants from GWAS summary statistics.** Comparison of MFD with alternative joint endpoints (MFD-MESuSiE, MFD-SuSiEx, MFD-XMAP) for detecting causal signals with non-zero effects in WB only, with non-causal ancestry-specific variants from GWAS summary statistics in one (stripe) or both (circle) ancestries. Results are stratified by the number of cross-ancestry causal SNPs (1, 3, or 5, corresponding to a total of 2, 6, and 10 causal SNPs, respectively) and per-causal SNP heritability ($h^2 = 10^{-4}$ or 2×10^{-4}; rows). **(a)** Precision-recall curves with PIP_{WB} thresholds ranging from 0.01 to 1. **(b)** Bar plots displaying power at controlled FDR levels of 0.01, 0.05, and 0.1.

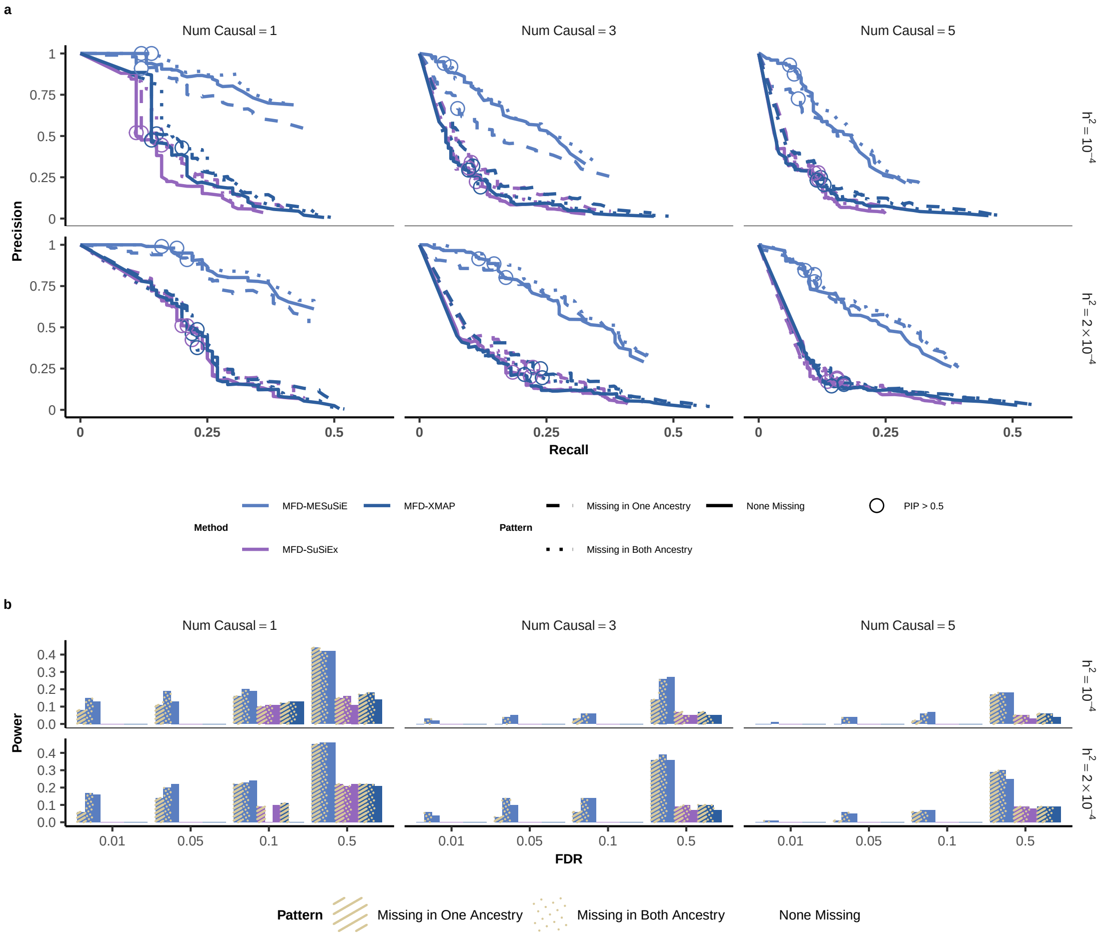

Supplementary Figure 95: **MFD endpoint sensitivity: BB contextual causal signals with non-causal ancestry-specific variants from GWAS summary statistics.** Comparison of MFD with alternative joint endpoints (MFD-MESuSiE, MFD-SuSiEx, MFD-XMAP) for detecting causal signals with non-zero effects in BB only, with non-causal ancestry-specific variants from GWAS summary statistics in one (stripe) or both (circle) ancestries. Results are stratified by the number of cross-ancestry causal SNPs (1, 3, or 5, corresponding to a total of 2, 6, and 10 causal SNPs, respectively) and per-causal SNP heritability ($h^2 = 10^{-4}$ or 2×10^{-4}; rows). **(a)** Precision-recall curves with PIP_{BB} thresholds ranging from 0.01 to 1. **(b)** Bar plots displaying power at controlled FDR levels of 0.01, 0.05, and 0.1.

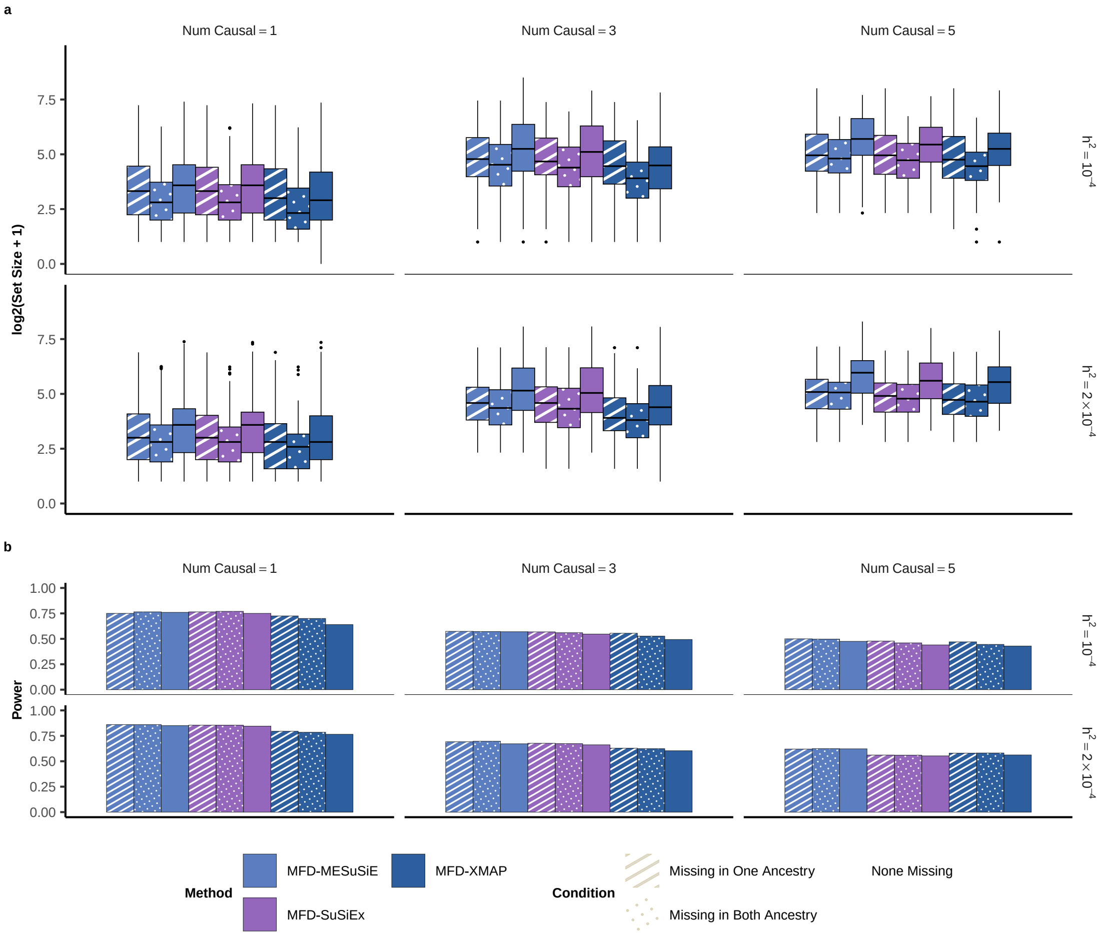

Supplementary Figure 96: **MFD endpoint sensitivity: credible set metrics with non-causal ancestry-specific variants from GWAS summary statistics and LD reference panel.** Comparison of 95% credible set sizes and power for MFD with alternative joint endpoints (MFD-MESuSiE, MFD-SuSiEx, MFD-XMAP) when non-causal ancestry-specific variants are missing from both GWAS summary statistics and LD reference panel in one (stripe) or both (circle) ancestries. Results are summarized across $n = 100$ independent regions, stratified by the number of causal SNPs and per-causal SNP heritability. **(a)** Boxplots displaying the $\log_2(\text{set size} + 1)$ of the 95% credible sets. **(b)** Bar plots displaying the power of the constructed 95% credible sets.

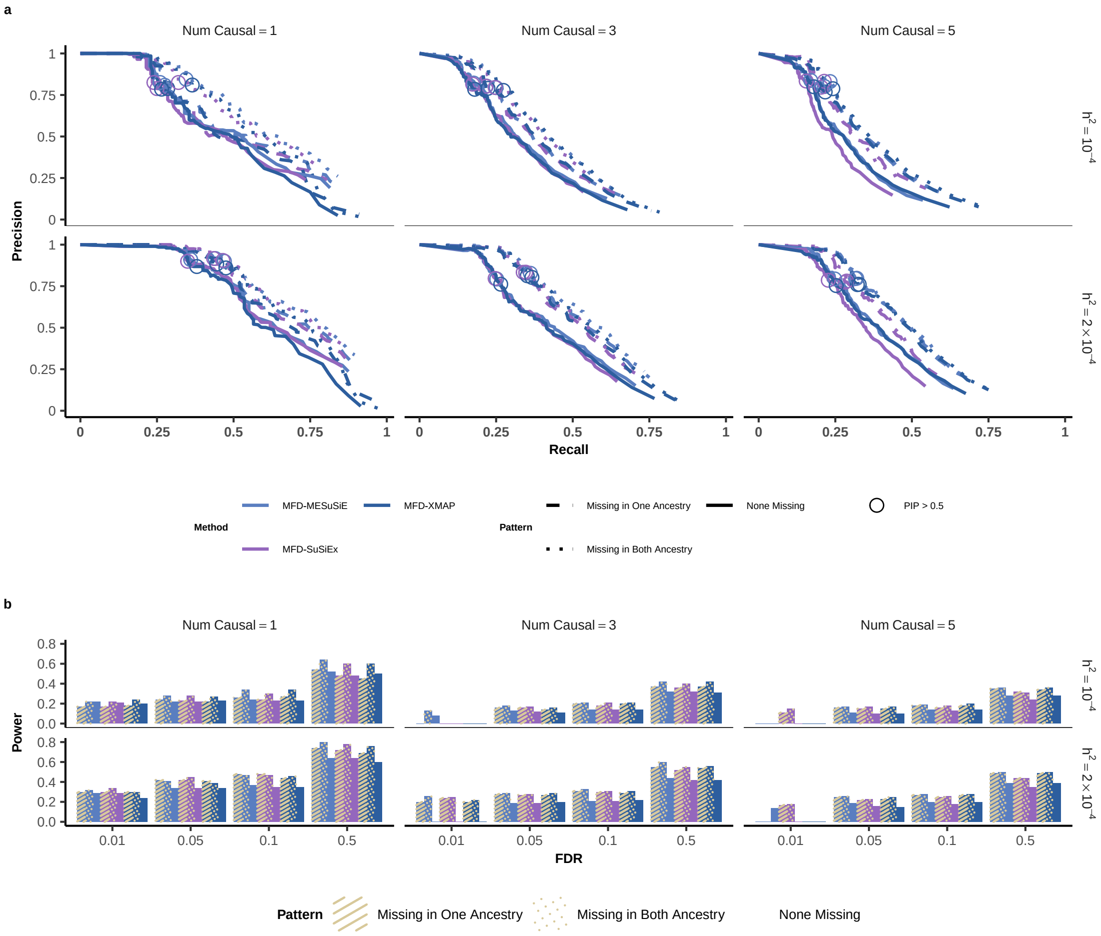

Supplementary Figure 97: **MFD endpoint sensitivity: detecting causal signals in at least one ancestry with non-causal ancestry-specific variants from GWAS summary statistics and LD reference panel ('Either').** Comparison of MFD with alternative joint endpoints (MFD-MESuSiE, MFD-SuSiEx, MFD-XMAP) with non-causal ancestry-specific variants missing from both GWAS summary statistics and LD reference panel in one (stripe) or both (circle) ancestries. Results are stratified by the number of cross-ancestry causal SNPs (1, 3, or 5, corresponding to a total of 2, 6, and 10 causal SNPs, respectively) and per-causal SNP heritability ($h^2 = 10^{-4}$ or 2×10^{-4}; rows). **(a)** Precision-recall curves with PIP_{either} thresholds ranging from 0.01 to 1. **(b)** Bar plots displaying power at controlled FDR levels of 0.01, 0.05, and 0.1.

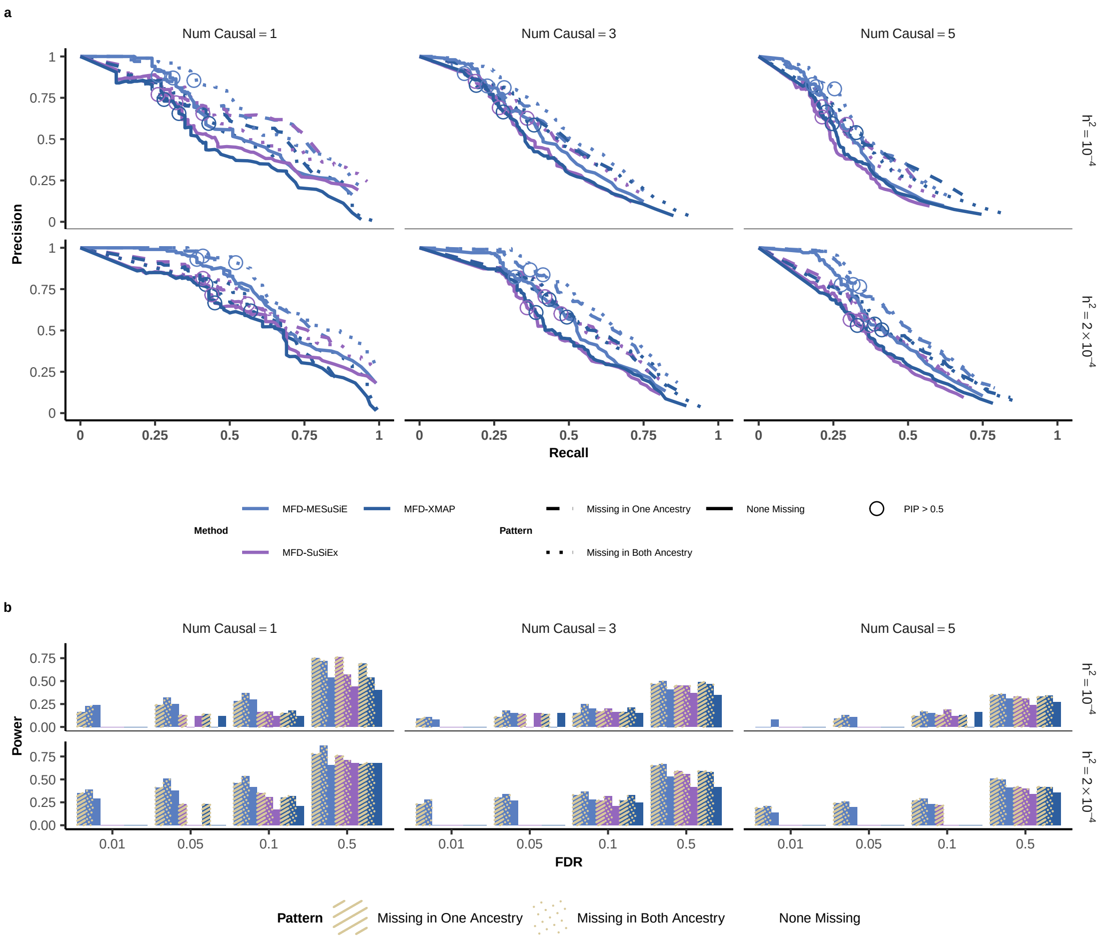

Supplementary Figure 98: **MFD endpoint sensitivity: detecting causal signals in both ancestries with non-causal ancestry-specific variants from GWAS summary statistics and LD reference panel ('Cross-ancestry').** Comparison of MFD with alternative joint endpoints (MFD-MESuSiE, MFD-SuSiEx, MFD-XMAP) with non-causal ancestry-specific variants missing from both GWAS summary statistics and LD reference panel in one (stripe) or both (circle) ancestries. Results are stratified by the number of cross-ancestry causal SNPs (1, 3, or 5, corresponding to a total of 2, 6, and 10 causal SNPs, respectively) and per-causal SNP heritability ($h^2 = 10^{-4}$ or 2×10^{-4}; rows). **(a)** Precision-recall curves with PIP_{shared} thresholds ranging from 0.01 to 1. **(b)** Bar plots displaying power at controlled FDR levels of 0.01, 0.05, and 0.1.

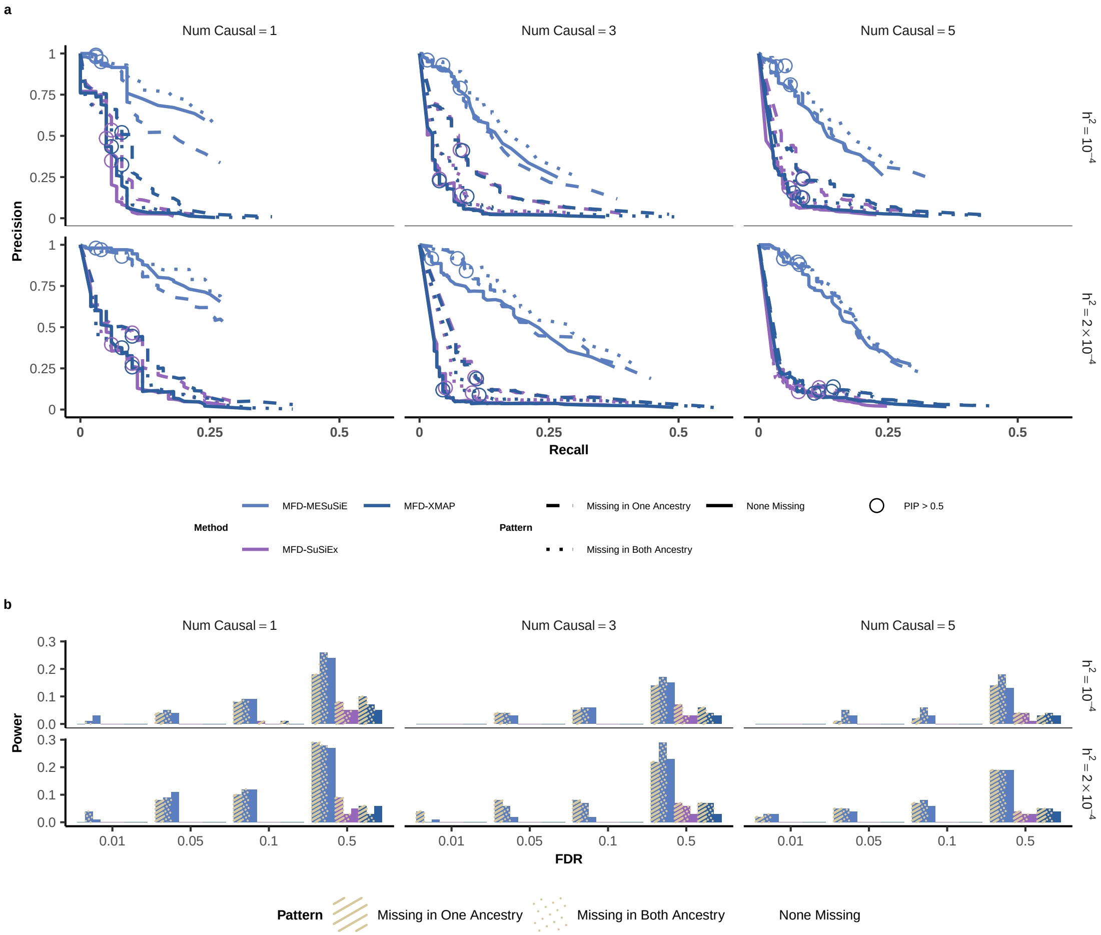

Supplementary Figure 99: **MFD endpoint sensitivity: WB contextual causal signals with non-causal ancestry-specific variants from GWAS summary statistics and LD reference panel.** Comparison of MFD with alternative joint endpoints (MFD-MESuSiE, MFD-SuSiEx, MFD-XMAP) for detecting causal signals with non-zero effects in WB only, with non-causal ancestry-specific variants missing from both GWAS summary statistics and LD reference panel in one (stripe) or both (circle) ancestries. Results are stratified by the number of cross-ancestry causal SNPs (1, 3, or 5, corresponding to a total of 2, 6, and 10 causal SNPs, respectively) and per-causal SNP heritability ($h^2 = 10^{-4}$ or 2×10^{-4}; rows). **(a)** Precision-recall curves with PIP_{WB} thresholds ranging from 0.01 to 1. **(b)** Bar plots displaying power at controlled FDR levels of 0.01, 0.05, and 0.1.

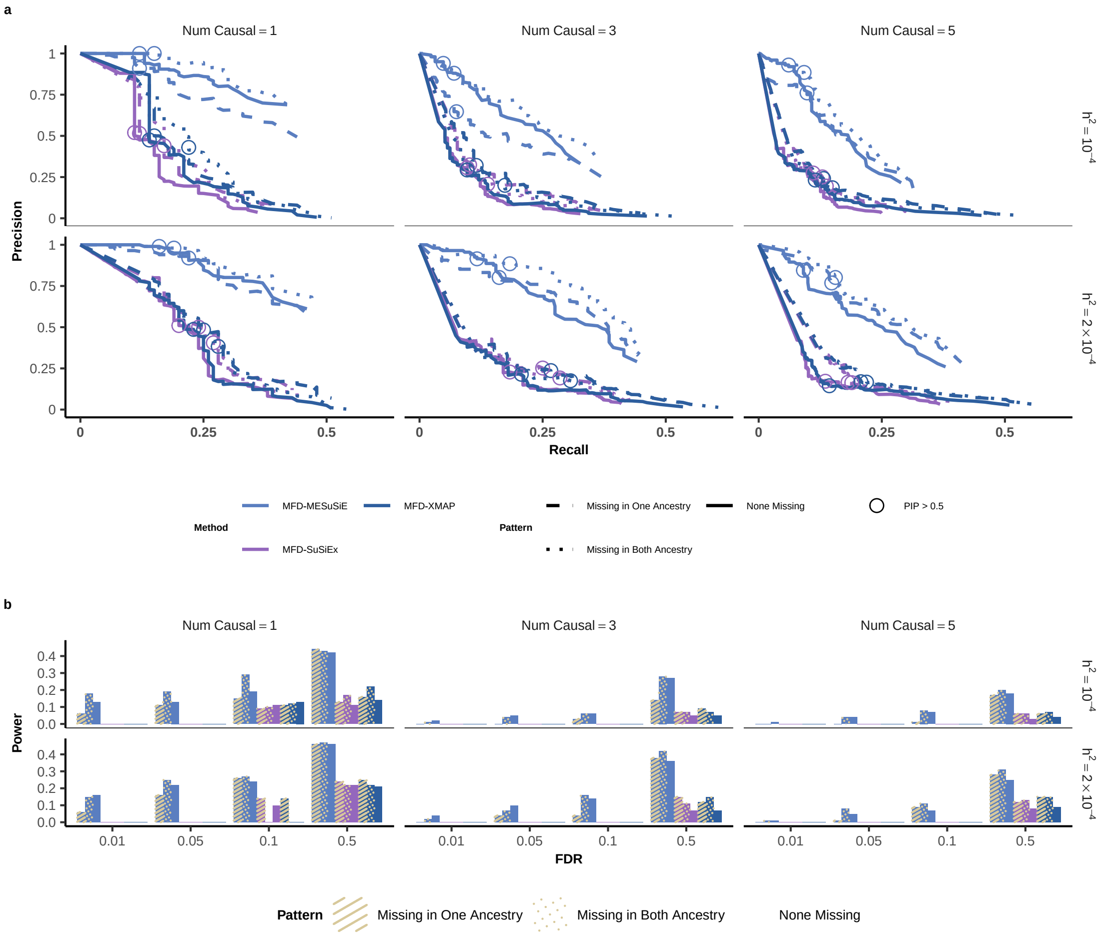

Supplementary Figure 100: **MFD endpoint sensitivity: BB contextual causal signals with non-causal ancestry-specific variants from GWAS summary statistics and LD reference panel.** Comparison of MFD with alternative joint endpoints (MFD-MESuSiE, MFD-SuSiEx, MFD-XMAP) for detecting causal signals with non-zero effects in BB only, with non-causal ancestry-specific variants missing from both GWAS summary statistics and LD reference panel in one (stripe) or both (circle) ancestries. Results are stratified by the number of cross-ancestry causal SNPs (1, 3, or 5, corresponding to a total of 2, 6, and 10 causal SNPs, respectively) and per-causal SNP heritability ($h^2 = 10^{-4}$ or 2×10^{-4}; rows). **(a)** Precision-recall curves with PIP_{BB} thresholds ranging from 0.01 to 1. **(b)** Bar plots displaying power at controlled FDR levels of 0.01, 0.05, and 0.1.

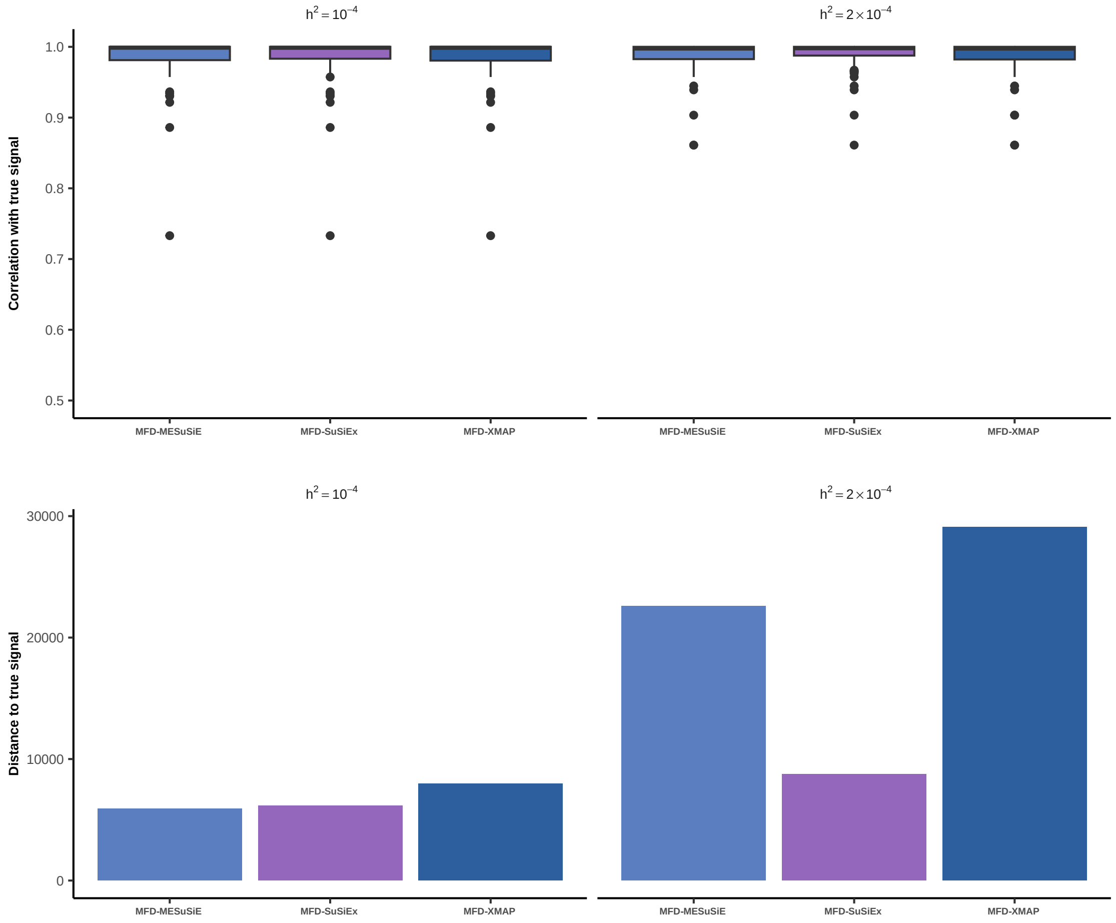

Supplementary Figure 101: **MFD endpoint sensitivity: causal AS-SV present in one ancestry from GWAS summary statistics.** Comparison of MFD with alternative joint endpoints (MFD-MESuSiE, MFD-SuSiEx, MFD-XMAP) when the causal ancestry-specific segregating variant (AS-SV) is present in one ancestry from GWAS summary statistics. Results are summarized across $n = 100$ independent regions, stratified by per-causal SNP heritability ($h^2 = 10^{-4}$ or 2×10^{-4}). **(a)** Boxplots displaying the correlation (LD $|r_{\max}|$) of the detected signal with the AS-SV for each MFD variant. **(b)** Bar plots displaying the mean genomic distance (in base pairs) from the detected signal to the AS-SV.

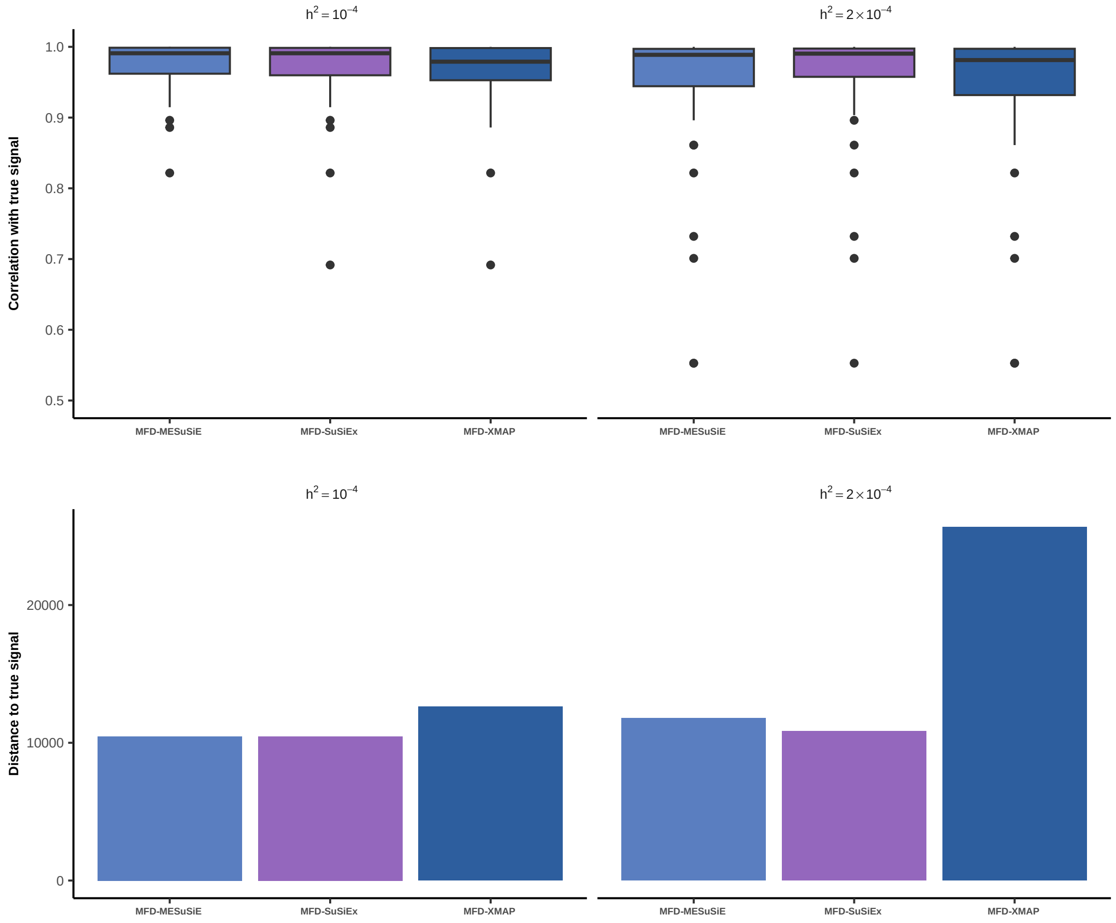

Supplementary Figure 102: **MFD endpoint sensitivity: causal AS-SV masked in both ancestries from GWAS summary statistics.** Comparison of MFD with alternative joint endpoints (MFD-MESuSiE, MFD-SuSiEx, MFD-XMAP) when the causal ancestry-specific segregating variant (AS-SV) is masked in both ancestries from GWAS summary statistics. Results are summarized across $n = 100$ independent regions, stratified by per-causal SNP heritability ($h^2 = 10^{-4}$ or 2×10^{-4}). **(a)** Boxplots displaying the correlation (LD $|r_{\max}|$) of the detected signal with the AS-SV for each MFD variant. **(b)** Bar plots displaying the mean genomic distance (in base pairs) from the detected signal to the AS-SV.

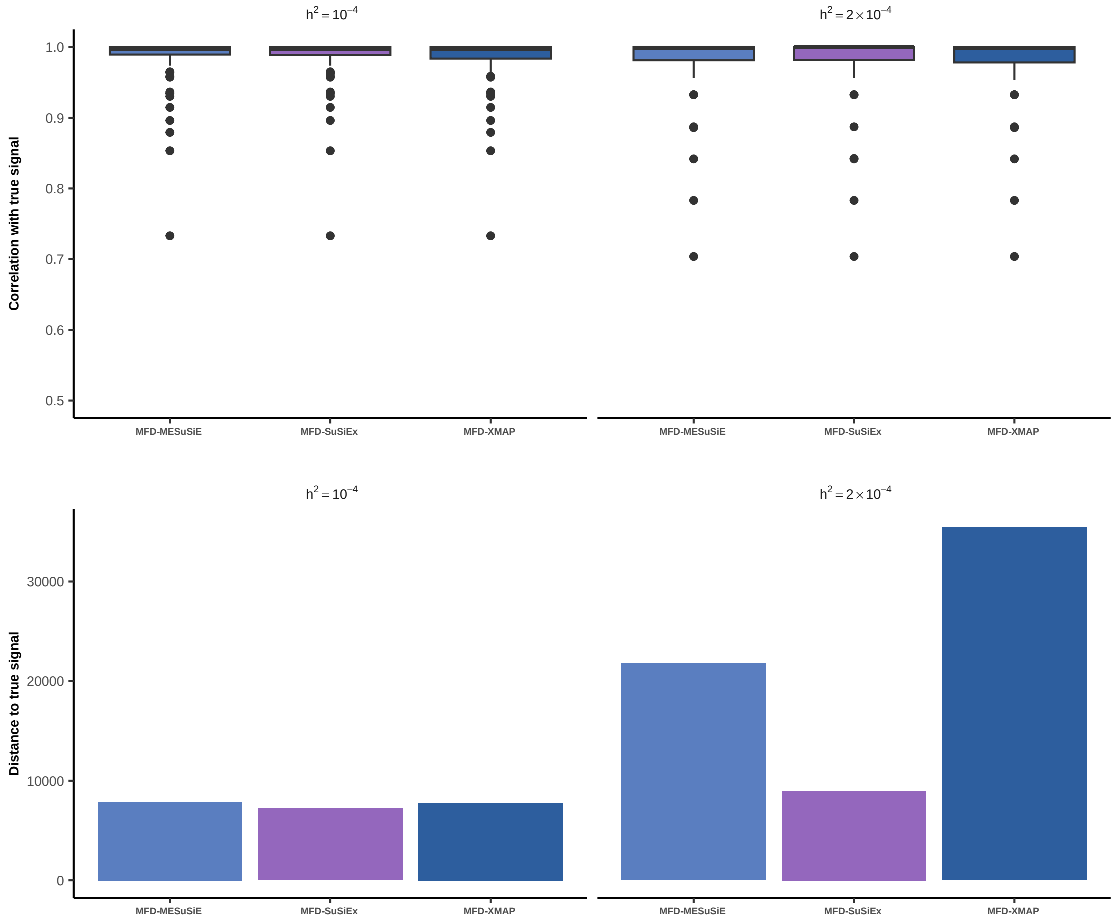

Supplementary Figure 103: **MFD endpoint sensitivity: causal AS-SV present in one ancestry from GWAS summary statistics and LD reference panel.** Comparison of MFD with alternative joint endpoints (MFD-MESuSiE, MFD-SuSiEx, MFD-XMAP) when the causal ancestry-specific segregating variant (AS-SV) is present from both GWAS and LD reference panel in one ancestry. Results are summarized across $n = 100$ independent regions, stratified by per-causal SNP heritability ($h^2 = 10^{-4}$ or 2×10^{-4}). **(a)** Boxplots displaying the correlation (LD $|r_{\max}|$) of the detected signal with the AS-SV for each MFD variant. **(b)** Bar plots displaying the mean genomic distance (in base pairs) from the detected signal to the AS-SV.

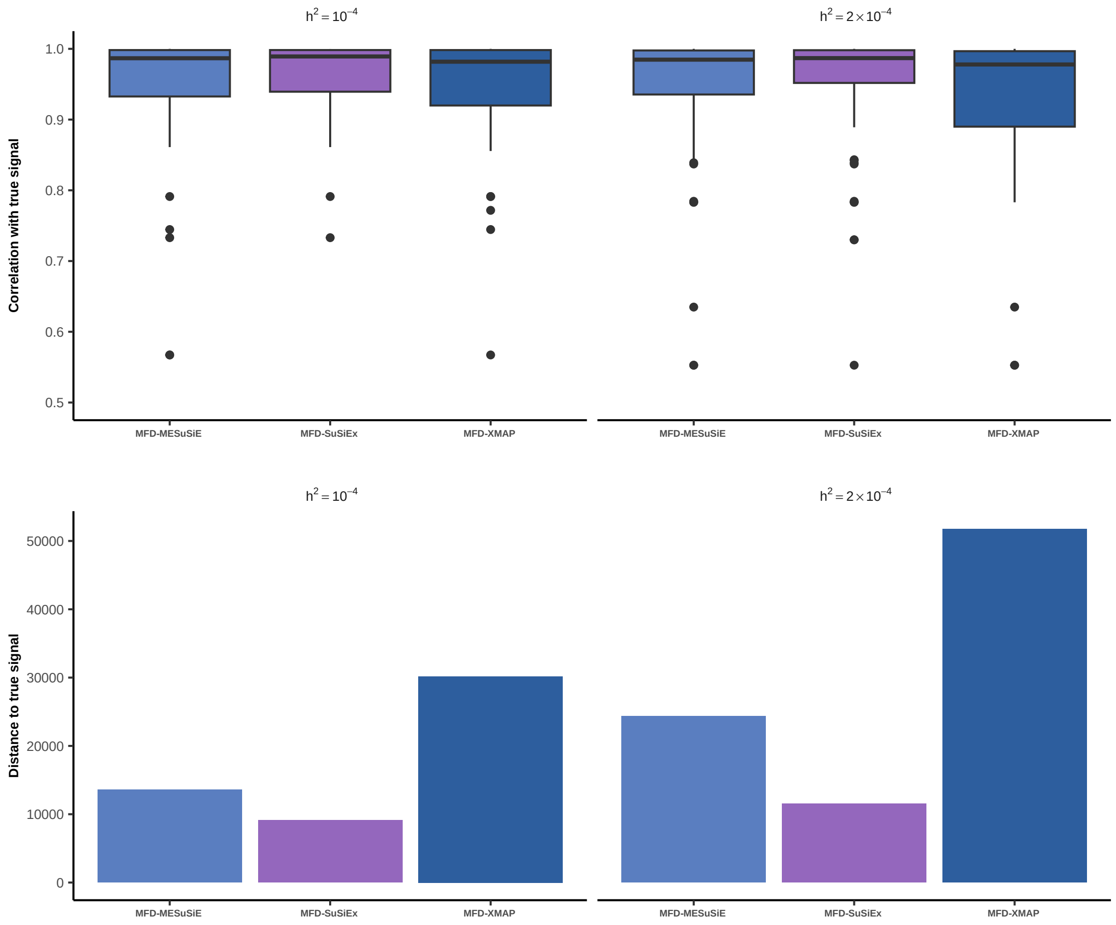

Supplementary Figure 104: **MFD endpoint sensitivity: causal AS-SV masked in both ancestries from GWAS summary statistics and LD reference panel.** Comparison of MFD with alternative joint endpoints (MFD-MESuSiE, MFD-SuSiEx, MFD-XMAP) when the causal ancestry-specific segregating variant (AS-SV) is masked in both ancestries from both GWAS summary statistics and the LD reference panel. Results are summarized across $n = 100$ independent regions, stratified by per-causal SNP heritability ($h^2 = 10^{-4}$ or 2×10^{-4}). **(a)** Boxplots displaying the correlation (LD r^2) of the detected signal with the AS-SV for each MFD variant. **(b)** Bar plots displaying the mean genomic distance (in base pairs) from the detected signal to the AS-SV.

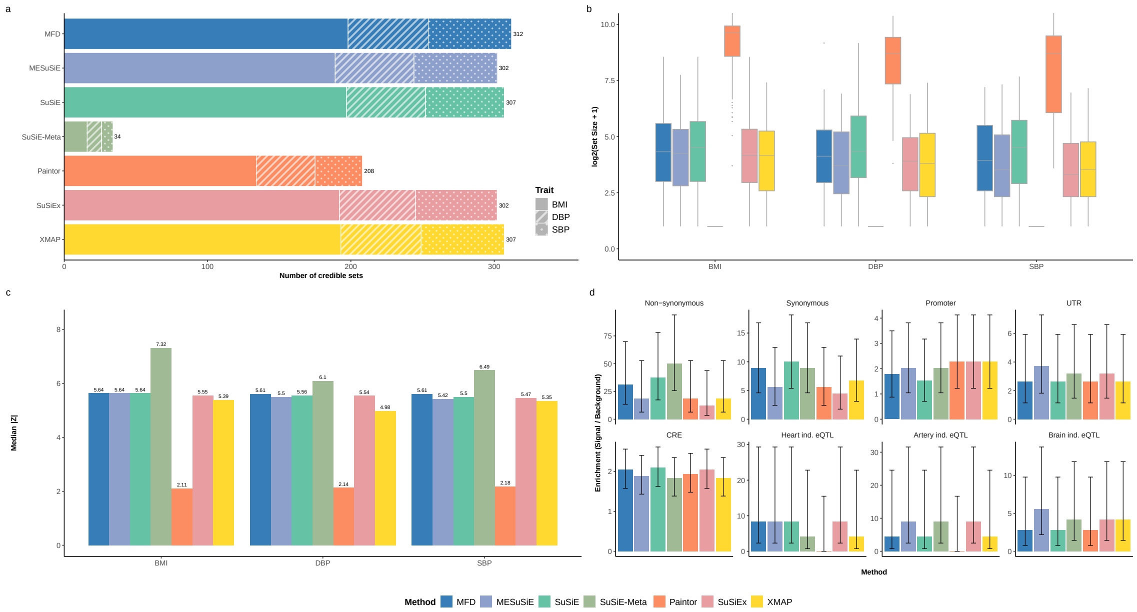

Supplementary Figure 105: **Multi-ancestry fine-mapping results summary in the three-ancestry real-data analysis.** This figure summarizes multi-ancestry fine-mapping results for Body Mass Index (BMI), Diastolic Blood Pressure (DBP), and Systolic Blood Pressure (SBP) using EUR, AFR, and EAS GWAS summary statistics with ancestry-matched LD reference panels. **(a)** Boxplot displaying the number of credible sets discovered by each method across traits. **(b)** Boxplot displaying the average $\log_2(\text{set size} + 1)$ of the 95% credible sets across analyzed genomic regions for BMI, DBP, and SBP. **(c)** Bar plot showing the marginal association strength of SNPs within the 95% credible sets, represented by the median absolute GWAS Z-score across ancestries. **(d)** Fold enrichment across functional annotation categories, calculated as the ratio of the proportion of potential causal SNPs carrying a given annotation to the corresponding proportion in the background set. Potential causal SNPs were defined as the top 100 variants ranked by PIP_{either} for each method. Error bars represent 95% confidence intervals.

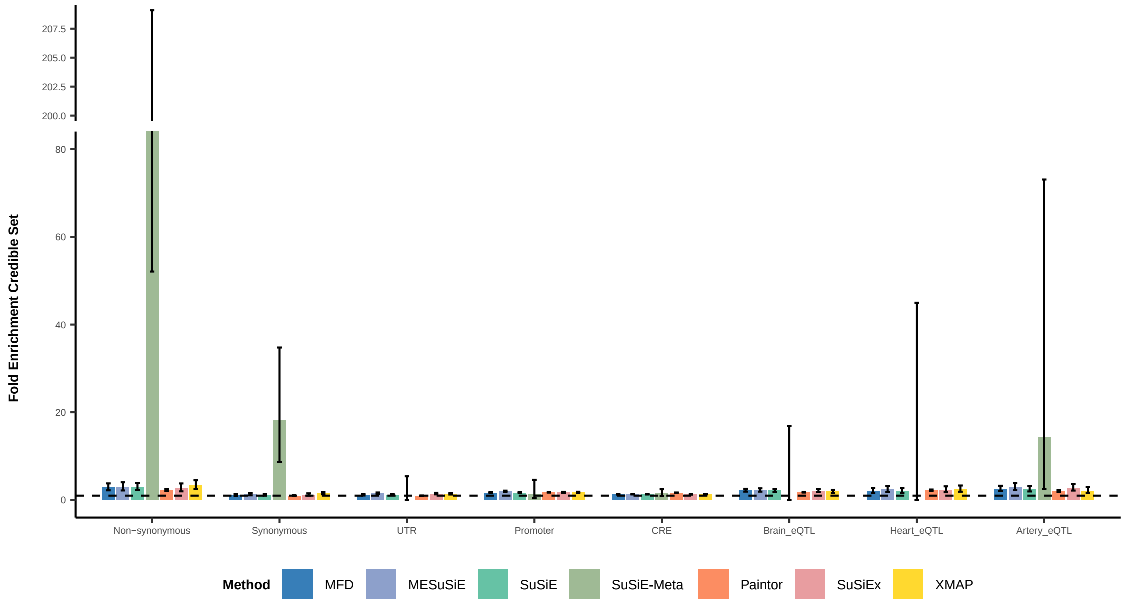

Supplementary Figure 106: **Functional enrichment comparison of fine-mapped variants in 95% credible sets (Three-ancestry analysis, EUR+AFR+EAS).** This analysis evaluates variants within 95% credible sets across 334 candidate regions for three traits: Body Mass Index (BMI), Diastolic Blood Pressure (DBP), and Systolic Blood Pressure (SBP) in the three-ancestry setting. Relative enrichment is calculated as the ratio of the proportion of functionally annotated SNPs within the 95% credible sets to the proportion of annotated SNPs outside of these sets across eight functional annotation categories: non-synonymous mutation, synonymous mutation, promoter, untranslated regions (UTRs), cis-regulatory elements (CRE), independent heart eQTL, independent artery eQTL, and independent brain eQTL. Error bars represent 95% confidence intervals.

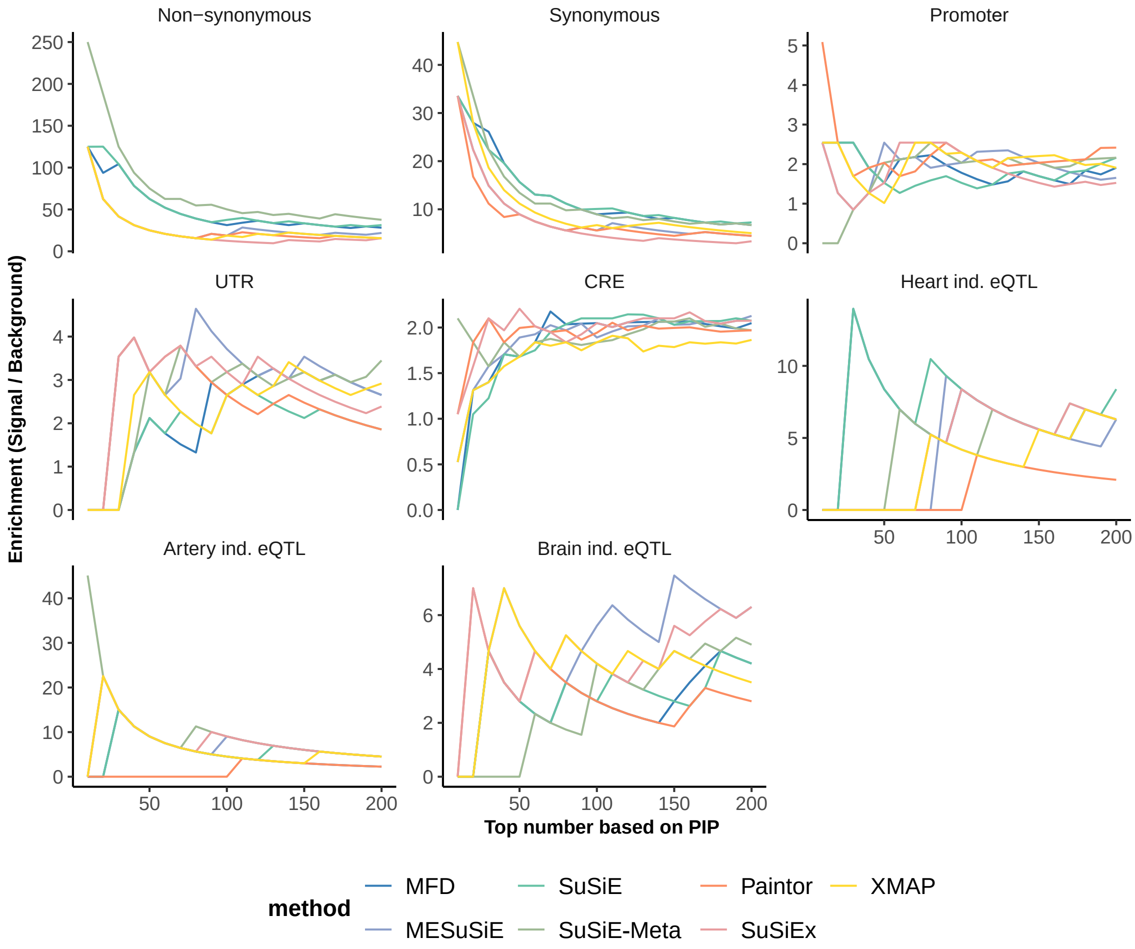

Supplementary Figure 107: **Functional enrichment of fine-mapped variants across varying top-ranked thresholds (Three-ancestry analysis, EUR+AFR+EAS).** This figure compares the functional enrichment of variants identified by evaluated fine-mapping methods, aggregated across three quantitative traits (BMI, DBP, SBP) in the three-ancestry setting. Panels display enrichment for eight functional annotation categories: non-synonymous mutation, synonymous mutation, promoter, untranslated regions (UTRs), cis-regulatory elements (CRE), independent heart eQTL, independent artery eQTL, and independent brain eQTL as a function of the number of top-ranked variants selected (ranging from the top 10 to 200 variants based on PIP_{either}, shown on the x-axis). The y-axis represents the fold enrichment calculated as the ratio of the proportion of annotated variants within the selected top set to the proportion of that annotation in the background set.

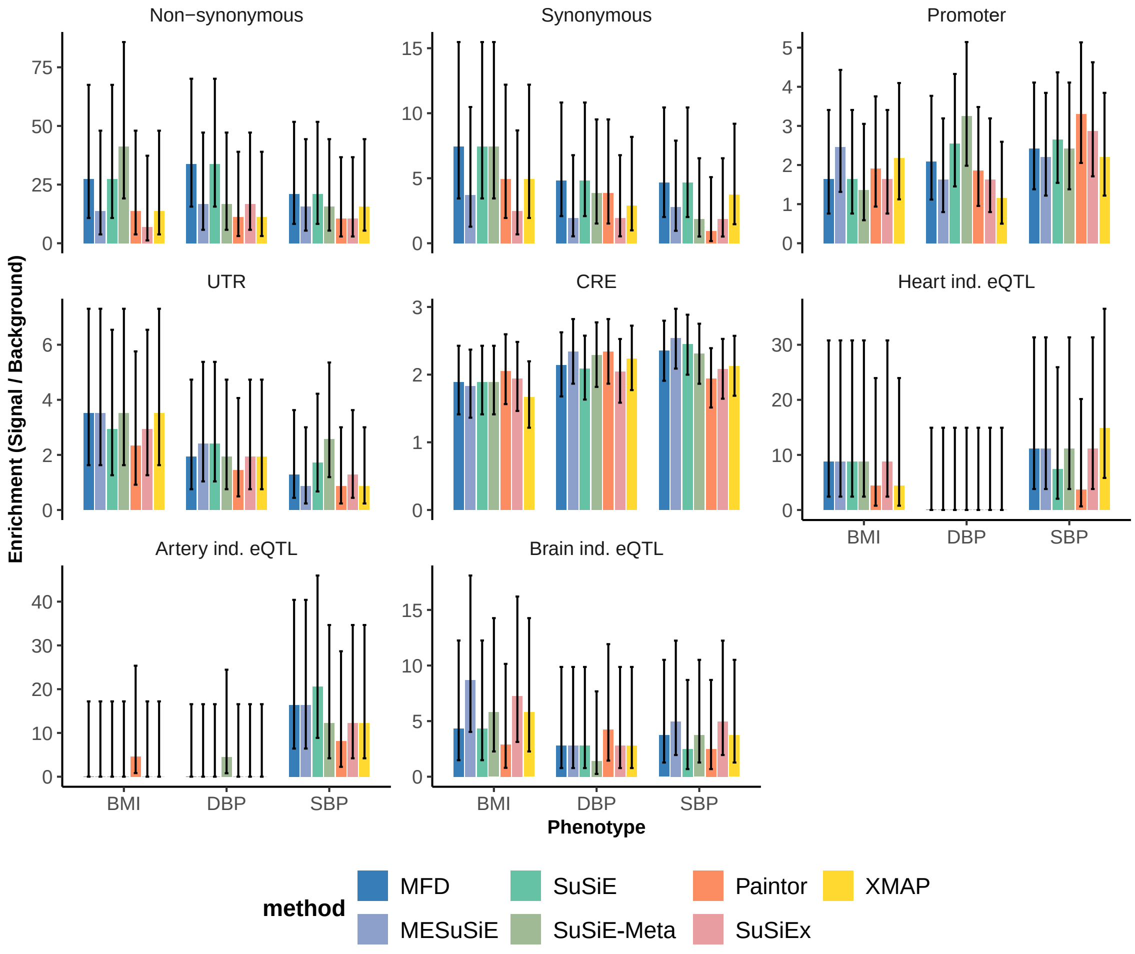

Supplementary Figure 108: **Functional enrichment of top 100 fine-mapped signals by trait (Three-ancestry analysis, EUR+AFR+EAS).** This figure summarizes multi-ancestry fine-mapping results for Body Mass Index (BMI), Diastolic Blood Pressure (DBP), and Systolic Blood Pressure (SBP) in the three-ancestry setting. It displays the fold enrichment across eight functional annotation categories: non-synonymous mutation, synonymous mutation, promoter, untranslated regions (UTRs), cis-regulatory elements (CRE), independent heart eQTL, independent artery eQTL, and independent brain eQTL. Enrichment is calculated as the ratio of the proportion of potential causal SNPs (defined as the top 100 variants based on PIP_{either}) carrying a specific annotation to the proportion of that annotation in the background set. Error bars represent 95% confidence intervals.

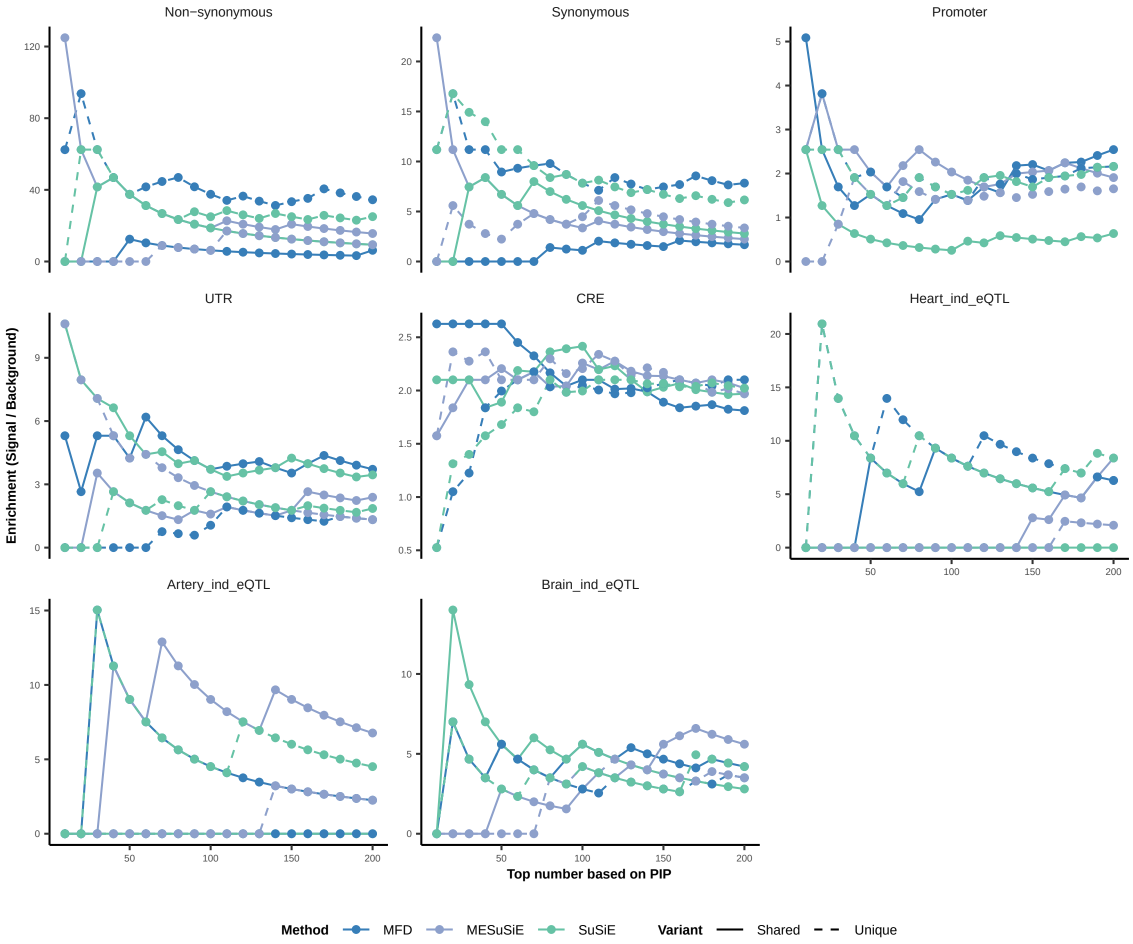

Supplementary Figure 109: **Functional enrichment of cross-ancestry and ancestry-specific fine-mapped signals across varying top-ranked thresholds (Three-ancestry analysis, EUR+AFR+EAS).** This figure evaluates the functional enrichment of variants classified as cross-ancestry (solid lines) or ancestry-specific/unique (dashed lines), aggregated across three quantitative traits (BMI, DBP, SBP) in the three-ancestry setting (EUR, AFR, EAS). The analysis compares methods across functional annotation categories (non-synonymous mutation, synonymous mutation, promoter, untranslated regions, cis-regulatory elements, and independent heart/artery/brain eQTLs). The x-axis displays the number of top-ranked variants selected based on PIP_{shared} for cross-ancestry signals, and ancestry-specific PIPs for ancestry-specific signals (ranging from 10 to 200), and the y-axis represents the fold enrichment, calculated as the ratio of the proportion of annotated variants in the selected set to the proportion in the background set.

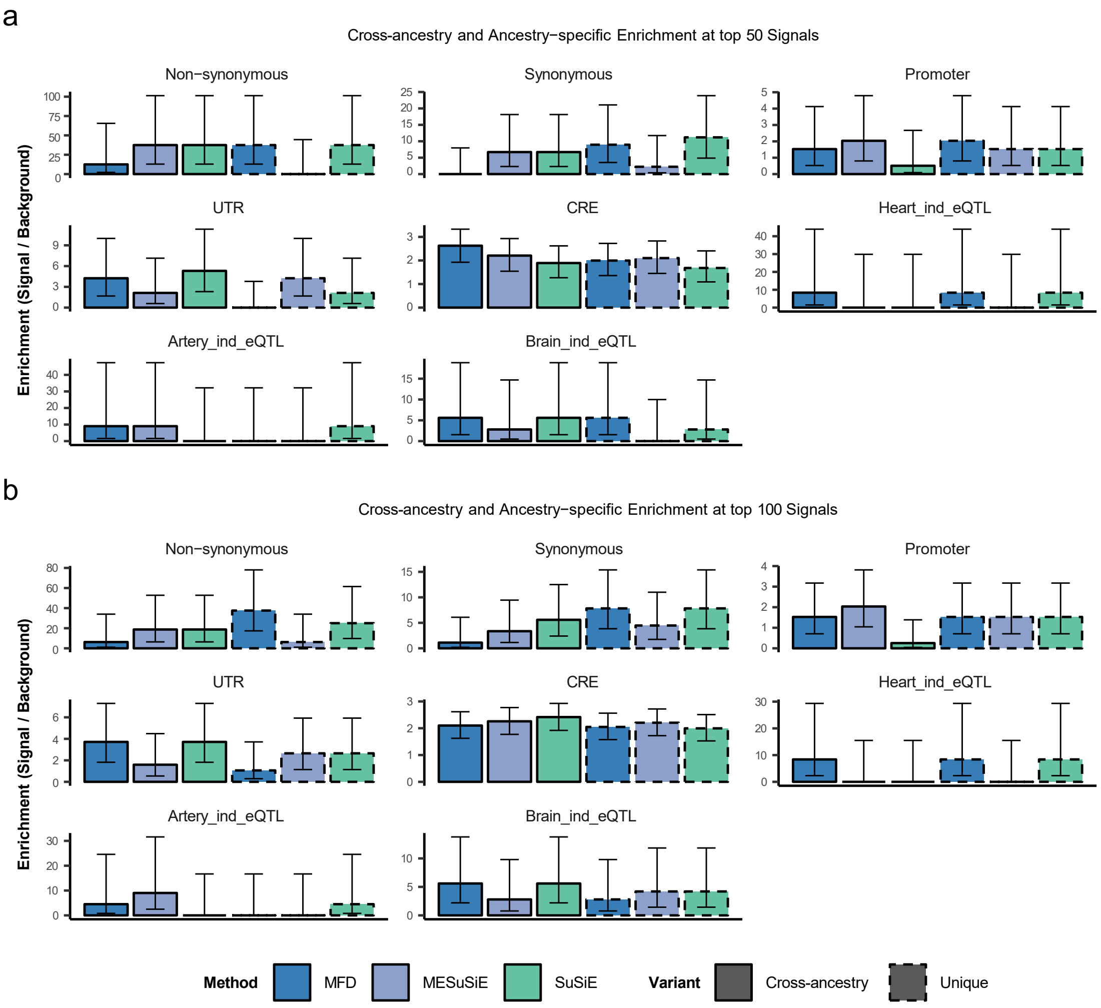

Supplementary Figure 110: **Functional enrichment of cross-ancestry and ancestry-specific fine-mapped signals in the three-ancestry analysis.** This figure evaluates the functional enrichment of variants classified as cross-ancestry (solid lines) or ancestry-specific/unique (dashed lines), aggregated across three quantitative traits (BMI, DBP, and SBP). The three-ancestry analysis combines European and African ancestry GWAS from All of Us with East Asian ancestry GWAS from the Taiwan Precision Medicine Initiative, and compares three fine-mapping methods (MFD, MESuSiE, and SuSiE) across functional annotation categories, including non-synonymous mutation, synonymous mutation, promoter, untranslated regions (UTRs), cis-regulatory elements (CRE), and independent heart, artery, and brain eQTLs. The x-axis displays the functional annotation categories, and the y-axis represents fold enrichment, calculated as the ratio of the proportion of annotated variants in the selected set to the proportion in the background set. **(a)** Functional enrichment of the top 50 variants, ranked by PIP_{shared} for cross-ancestry signals and by ancestry-specific PIPs for unique signals. **(b)** Functional enrichment of the top 100 variants, ranked by PIP_{shared} for cross-ancestry signals and by ancestry-specific PIPs for unique signals. Error bars represent 95% confidence intervals.
