# Supplementary material for: Systematic benchmarking of multi-ancestry fine-mapping under variant heterogeneity and LD mismatch
Source: Brief Bioinform. 2026 Oct 19;27(5):bbag574. doi: 10.1093/bib/bbag574 (PMC13677793; doi:10.1093/bib/bbag574)
Supplement: Supplementary file 1 [file supplementary_material_bbag574.zip › Supplementary_Notes_Submission_Revision_submission_bbag574.pdf]

Supplementary Notes

Table of Contents

Supplementary Note 1 | Unified Generative Model for Benchmarking 2

1.1 Joint modeling approaches 3

1.2 Post-hoc integration approaches 5

1.3 Association-level integration approaches 5

Supplementary Note 2 | Implementation of Multi-Ancestry Fine-mapping Methods 6

2.1 Association-level integration approach 6

2.2 Post-hoc integration approach 7

2.3 Joint modeling approaches 7

2.4 Multi-ancestry Fine-mapping Decision (MFD) 9

Supplementary Note 3 | General Simulation Framework and Extensions 10

3.1 Ancestry-specific SNP scenario 12

3.1.1 Input-matched sensitivity analysis 13

3.2 LD mismatch scenario 14

3.3 Admix simulation 15

3.3.1 Admix simulation design 15

3.3.2 Admix simulation results 17

3.4 Functional annotation and Evo2-derived sequence-effect annotation analysis 18

3.4.1 Simulation design 18

3.4.2 Simulation results 19

3.5 High allelic heterogeneity simulation 21

3.5.1 High allelic heterogeneity simulation design 21

3.5.2 High allelic heterogeneity simulation results 22

3.6 MFD endpoint sensitivity analysis 23

3.7 Bootstrap confidence-interval analysis 24

3.8 Empirical credible set coverage analysis 25

3.9 Computation time and memory usage 27

Supplementary Note 4 | Genome-wide Association Study in All of Us 27

Supplementary Note 5 | Supplementary Three-Ancestry Real Data Application 29

5.1 Supplementary three-ancestry real data application design 29

5.2 Supplementary results for three-ancestry real data applications 30

Supplementary Note 6 | Construction of the Summary Table 32

Supplementary References 34

Unified Generative Model for Benchmarking

To rigorously evaluate and compare multi-ancestry fine-mapping methods, we require a standardized generative framework that accurately captures genetic architecture across diverse populations. Specifically, we need a data-generating model that explicitly distinguishes between causal variants shared across ancestries and those polymorphic only within specific groups.

Consider two ancestries, indexed by $k \in \{1, 2\}$, comprising N_k individuals with a continuous phenotype vector $\boldsymbol{y}_k$. We partition the total set of variants into M_{shared} shared variants (observed in both ancestries) and $M_{\text{AS},k}$ ancestry-specific segregating variants (AS-SVs, observed exclusively within ancestry k). Let $\mathbf{X}_{\text{shared},k}$ denote the genotype matrix for the shared variants in ancestry k, and $\mathbf{X}_{\text{AS},k}$ denote the genotype matrix for the AS-SVs, where entries represent alternative-allele counts.

To express this multi-ancestry formulation compactly for our simulation and evaluation pipeline, we stack the phenotypes into a single joint vector $\boldsymbol{y}$. Similarly, we construct a stacked genotype matrix for the shared variants and a block-diagonal matrix for AS-SVs, denoted as $\mathbf{X}_{\text{shared}}$ and $\mathbf{X}_{\text{AS}}$ respectively. We allow the underlying causal effect sizes for shared variants to be heterogeneous across ancestries, stacking them as $\boldsymbol{b}_{\text{shared}} = (\boldsymbol{b}_{\text{shared},1}^{\top}, \boldsymbol{b}_{\text{shared},2}^{\top})^{\top}$. Likewise, the effect sizes for AS-SVs are collected in the stacked vector $\boldsymbol{b}_{\text{AS}}$. The joint relationship between phenotypes, genotypes, and causal effect sizes across ancestries in our benchmarking framework is defined as:

$$\boldsymbol{y} = \mathbf{X}_{\text{shared}}\boldsymbol{b}_{\text{shared}} + \mathbf{X}_{\text{AS}}\boldsymbol{b}_{\text{AS}} + \boldsymbol{\epsilon}, \qquad (1)$$

where $\boldsymbol{\epsilon}$ stacks the ancestry-specific residual errors $\boldsymbol{\epsilon}_k$. We assume independent homoskedastic noise within each ancestry, $\boldsymbol{\epsilon}_k \sim \mathcal{N}(0, \sigma_k^2\mathbf{I})$ [1], implying a block-diagonal covariance structure for $\boldsymbol{\epsilon}$. Equation (1) serves as the unified ground truth for our benchmarking study. By decomposing the true genetic effects into a shared component, $\boldsymbol{b}_{\text{shared}}$, and an ancestry-specific component, $\boldsymbol{b}_{\text{AS}}$, this framework allows us to directly measure a method's ability to disentangle cross-ancestry causal signals from those that are unique, reliably imputed, or polymorphic in only certain populations.

We evaluate how well existing multi-ancestry fine-mapping methods recover these causal effects. These existing methods can be broadly classified into two categories based on how they integrate genotype–phenotype associations across ancestries. The first category comprises joint modeling approaches that fit a unified probabilistic model across ancestries, enabling information sharing through shared genetic architecture and correlated effect-size priors. The second category consists of univariate-based approaches that rely on ancestry-specific univariate models, differing primarily in when cross-ancestry information is aggregated. One subclass within this category performs integration at the association level, combining ancestry-specific GWAS summary statistics into a single meta-analyzed dataset before applying standard univariate fine-mapping using a composite or working linkage disequilibrium (LD) matrix. A second subclass combines results in a post-hoc manner, performing univariate fine-mapping separately within each ancestry and subsequently combining the resulting fine-mapping outputs using heuristic or statistical aggregation strategies. By mapping these distinct algorithmic assumptions regarding cross-ancestry LD structure, variant support, and effect sharing back to our unified generative framework, we can systematically benchmark the conditions under which multi-ancestry data is most effectively leveraged.

Joint modeling approaches

Existing joint modeling approaches such as Paintor [2], SuSiEx [3], MultiSuSiE [4], MESuSiE [1], XMAP [5], and CARMA-X [6] are typically applied to the common set of SNPs observed across ancestry groups. As a result, the fitted model is effectively restricted to variants shared across ancestries, which can be represented in Eq. (1) as

$$\boldsymbol{y} = \mathbf{X}_{\text{shared}}\boldsymbol{b}_{\text{shared}} + \boldsymbol{\epsilon},$$

by setting $\mathbf{X}_{\text{AS}} = \mathbf{0}$. Under this restriction, these methods differ primarily in how they represent multiple causal signals within a fine-mapping region and how they couple effect sizes $\boldsymbol{b}_{shared,1}$ and $\boldsymbol{b}_{shared,2}$ a priori. Broadly, these methods fall into two categories: SuSiE-based models that decompose effects into a sum of sparse components, and global configuration models that place priors directly on regional causal patterns.

SuSiE-based approaches, including SuSiEx, MultiSuSiE, MESuSiE, and XMAP, adopt a “sum of single effects” representation for each ancestry [7]. Specifically, for ancestry $k \in \{1,2\}$, the shared-SNP effect vector is expressed as

$$\boldsymbol{b}_{\text{shared},k} = \sum_{l=1}^{L} \boldsymbol{\gamma}_l \beta_{kl},$$

where $\boldsymbol{\gamma}_l \in \{0,1\}^{M_{\text{shared}}}$ is a one-hot indicator selecting a single shared SNP for effect l, and $\beta_{kl} \in \mathbb{R}$ denotes the effect size for that SNP in ancestry k. Methods in this family differ in the prior structure assigned to β_{1l} and β_{2l}. For instance, SuSiEx enforces a shared causal index across ancestries through $\boldsymbol{\gamma}_l$ while allowing for effect-size heterogeneity assuming independence between β_{1l} and β_{2l}. MultiSuSiE and XMAP extend this by retaining the shared index but explicitly coupling effect sizes via a bivariate normal prior,

$$\begin{pmatrix} \beta_{1l} \\ \beta_{2l} \end{pmatrix} \sim \mathcal{N}\left(\begin{pmatrix} 0 \\ 0 \end{pmatrix}, \boldsymbol{\Sigma}\right),$$

where $\boldsymbol{\Sigma}$ is the covariance matrix that models the correlation between variant effects across ancestries. This dependence structure allows the model to borrow strength across populations, where strong evidence in one ancestry informs the estimate in the other, thereby increasing detection power for cross-ancestry signals. MESuSiE further relaxes strict sharing by augmenting the decomposition with ancestry-specific activation indicators $s_{kl} \in \{0,1\}$, replacing β_{kl} with $s_{kl}\beta_{kl}$ so that a selected SNP may be active in one ancestry but null in the other:

$$\boldsymbol{b}_{\text{shared},k} = \sum_{l=1}^{L} \boldsymbol{\gamma}_l \left(s_{kl}\beta_{kl}\right).$$

In contrast, Paintor and CARMA-X do not rely on the single effect decomposition. Instead, they model the posterior over regional causal configurations directly. Paintor evaluates the probability of a binary causal configuration vector, leveraging functional annotations to modulate prior inclusion probabilities. CARMA-X utilizes stochastic search to perform Bayesian model averaging over sparse multi-SNP configurations. This allows it to accommodate complex LD patterns and multiple causal variants without committing to a fixed sum of effect representation.

Despite their methodological differences, these joint modeling approaches tend to focus on the shared variant intersection (M_{shared}) under $\mathbf{X}_{\text{AS}} = \mathbf{0}$. As a result, causal effects driven by AS-SVs may be misattributed or missed in the analysis.

Post-hoc integration approaches

Post-hoc integration approaches decompose equation (1) into k independent estimation tasks. Rather than fitting the stacked phenotype vector jointly, these methods perform fine-mapping separately within each ancestry $k \in \{1,2\}$ using the ancestry-specific model:

$$\mathbf{y}_k = \mathbf{X}_{\text{shared},k}\boldsymbol{b}_{\text{shared},k} + \mathbf{X}_{\text{AS},k}\boldsymbol{b}_{\text{AS},k} + \boldsymbol{\epsilon}_k,$$

where each analysis uses the full ancestry-specific design matrix $\widetilde{\mathbf{X}}_k = [\mathbf{X}_{\text{shared},k}, \mathbf{X}_{\text{AS},k}]$ to estimate the corresponding effect vector $\widetilde{\boldsymbol{b}}_k = (\boldsymbol{b}_{\text{shared},k}^\top, \boldsymbol{b}_{\text{AS},k}^\top)^\top$. As a result, post-hoc integration approaches naturally accommodate both shared variants and AS-SVs.

A defining feature of this framework is the independent estimation of $\widetilde{\boldsymbol{b}}_1$ and $\widetilde{\boldsymbol{b}}_2$. This preserves ancestry-specific LD patterns and allows for unrestricted heterogeneity in effect sizes. Multi-ancestry integration is performed only after each analysis, typically by combining ancestry-specific posterior inclusion probabilities (PIPs) using heuristic rules [8,9]. For example, a pooled PIP for variant j is often defined as the maximum PIP across ancestries [1]:

$$\text{PIP}_{\text{pool},j} = \max_k \text{PIP}_{kj}.$$

While effectively detecting ancestry-specific segregating variants, this approach fails to leverage the increased sample size, potential genetic correlations and LD diversity across populations to improve fine-mapping resolution. Consequently, post-hoc integration approaches can be underpowered for truly cross-ancestry signals compared to methods that jointly model $\boldsymbol{b}_{shared,1}$ and $\boldsymbol{b}_{shared,2}$ during posterior inference.

Association-level integration approaches

Association-level integration approaches approximate the joint generative model in Equation (1) by collapsing the ancestry-specific genetic signals into a single set of summary statistics defined across the union of all variant indices. Rather than explicitly pooling the genotype and phenotype matrices to construct a synthetic working model, these methods naturally operate directly at the summary statistic level. They implicitly assume a unified vector of causal effects across both shared and ancestry-specific variants, utilizing ancestry-specific marginal GWAS estimates to proxy the association signals. Let $\widehat{\boldsymbol{b}}_1$ and $\widehat{\boldsymbol{b}}_2$ denote these ancestry-specific marginal effect

estimates, padded such that they are defined on the complete union index set of variants. A generic meta-analysis estimator is then constructed as:

$$\widehat{\boldsymbol{b}}_{meta} = \mathbf{W}\widehat{\boldsymbol{b}}_1 + (\mathbf{I} - \mathbf{W})\widehat{\boldsymbol{b}}_2,$$

where $\mathbf{W}$ is a diagonal weight matrix determined by sample size or inverse-variance weights, and missing variants are handled by assigning zero weight in the ancestry where they are unobserved. For shared variants, this aggregation typically corresponds to a fixed-effect model [10], which increases power when effects are consistent across ancestries.

Fine-mapping is then performed by applying a single-ancestry summary-statistic method to $\widehat{\boldsymbol{b}}_{meta}$ together with an LD matrix $\mathbf{R}_{meta}$ that represents the synthetic population, producing PIPs over the combined variant set. The principal limitation is that collapsing ancestries into a synthetic model averages over effect-size heterogeneity and ancestry-specific LD patterns; when cross-ancestry effects differ between ancestries or when AS-SVs drive the association signal, this approximation can deviate from the data-generating process in Eq. (1) and degrade posterior calibration and fine-mapping resolution.

Implementation of Multi-Ancestry Fine-mapping Methods

Below, we describe the implementation details of different multi-ancestry fine-mapping methods evaluated in this study. For each method, we specify the parameters used and the procedure for computing PIPs and 95% CSs, which serve as the primary metrics for evaluating fine-mapping performance.

Association-level integration approach

We first obtained meta-analyzed Z-scores across ancestry groups using fixed-effect meta-analysis implemented in METAL (version 2011-03-25). Corresponding to Z-scores, we considered two strategies for constructing the LD reference: (i) a sample size weighted average of ancestry-specific LD matrices (“SuSiE-weighted”), and (ii) LD estimated from pooled genotype data across individuals (“SuSiE-merged”).

We applied the susie_rss function to the meta-analyzed Z-scores and corresponding LD matrix, setting the maximum number of causal variants (L) to 10. For simulated data, the check_prior parameter was set to FALSE; all other parameters were left at default values. PIPs and CSs were obtained directly from the fitted SuSiE model. Because the meta-analysis based SuSiE model

produces a single PIP per variant, we used this output PIP as the measure of causal evidence in benchmarking, without distinguishing cross-ancestry and ancestry-specific effects.

Post-hoc integration approach

We performed fine-mapping separately within each ancestry using the susie_rss function from the susieR package (v0.12.35), with default settings throughout, except the check_prior parameter was set to FALSE to allow the model to proceed despite potential discrepancies between the summary statistics and the reference LD matrix for simulated data. To integrate ancestry-specific fine-mapping results into multi-ancestry outputs, we applied a post-hoc aggregation strategy following [[1]].

Specifically, let PIP_k denote the PIP of a variant in ancestry *k.* We derived the following metrics to summarize multi-ancestry evidence. First, we defined the overall evidence as

$$PIP_{either} = \max_k PIP_k$$

which serves as a heuristic summary of causal evidence across ancestries.

Second, to capture cross-ancestry evidence, we defined

$$PIP_{shared} = \min_k PIP_k$$

which prioritizes variants consistently supported across ancestries.

Finally, the original ancestry-specific PIPs were retained to quantify ancestry-specific effects. Furthermore, we constructed multi-ancestry credible sets by taking the union of the independent 95% CSs from each ancestry; this combined set is expected to contain at least one causal variant from either ancestry with at least 95% probability.

Joint modeling approaches

Joint modeling approaches considered in this study, including Paintor, SuSiEx, XMAP, MultiSuSiE, and CARMA-X, produce a single PIP per variant, which reflects overall evidence that the variant is causal in the locus. This PIP was used as the ranking statistic for benchmarking across all evaluation tasks, including detection of cross-ancestry, ancestry-specific, and overall (either) causal signals. In contrast, MESuSiE explicitly decomposes causal evidence into multiple ancestry-aware PIPs, which were evaluated using their corresponding definitions.

Paintor

We applied Paintor (v3.0) using GWAS summary statistics and ancestry-specific LD matrices from both ancestry groups, running the software with the -mcmc flag. Paintor outputs a single PIP per variant, which we used as the measure of causal evidence. To construct the 95% credible set, we rescaled the PIPs across all variants in the locus to sum to one, ranked the variants in descending order, and included variants until their cumulative normalized posterior probability reached at least 0.95.

SuSiEx

We applied SuSiEx (v1.1.2) using GWAS summary statistics and ancestry-specific LD matrices from both ancestry groups, following the SuSiEx guidelines [https://github.com/getian107/SuSiEx]. To quantify the overall evidence for causality, we first extracted the per-signal PIPs from the .snp output files, where each signal corresponds to one single-effect component identified by SuSiEx. We then calculated an overall PIP (PIP_{total}) for each SNP using the formula $PIP_{total} = 1 - \prod_l (1 - PIP_l)$ [7], where PIP_l denotes the PIP of the SNP in the l-th credible set. This aggregate metric represents the probability that the SNP is a causal variant for at least one of the independent signals identified by the model, and we utilized the resulting PIP_{total} as a unified metric to detect causal variants, regardless of their ancestry specificity. Finally, the variants belonging to the 95% credible sets were identified directly from the .cs output files.

XMAP

We applied XMAP (v1.0.1) using GWAS summary statistics and ancestry-specific LD matrices, following the XMAP guidelines [https://github.com/YangLabHKUST/XMAP]. Polygenic parameters were estimated only in real data analyses as no polygenic component was included in the simulation design [5]. We extracted PIPs and CSs using the get_pip and get_CS functions, respectively. The resulting PIP served as a unified metric for causal variant detection.

MultiSuSiE

We applied MultiSuSiE using GWAS summary statistics and ancestry-specific LD matrices with multisusie_rss function, following MultiSuSiE guidelines [https://github.com/jordanero/MultiSuSiE]. We extracted PIPs and CS from the output result directly. The resulting PIP was used as a unified metric for causal variant detection.

CARMA-X

We applied CARMA-X (v1.0.0) using GWAS summary statistics and ancestry-specific LD matrices with the "CARMAX" function, following the CARMA-X tutorial [https://github.com/ZikunY/CARMAX], with the effect size prior as "Spike-slab" and LD.estimation = FALSE to use the block-diagonal LD matrix directly rather than estimating cross-ancestry LD. Credible sets were obtained by taking the intersection of credible set variants across ancestries. The PIP was extracted from the CARMA-X output directly and used as a unified metric for causal variant detection.

MESuSiE

We applied MESuSiE (v1.0) using the meSuSie_core function with default settings and $L = 10$. MESuSiE explicitly models cross-ancestry architecture and directly outputs distinct probability metrics for each SNP: an overall PIP (PIP_{either}, representing the probability that a variant is causal in at least one population), a cross-ancestry PIP (PIP_{shared}, quantifying the evidence for a cross-ancestry effect), and ancestry-specific PIPs. We utilized these metrics directly to prioritize variants and characterize their ancestry specificity. The 95% credible sets were extracted directly from the model output.

Multi-ancestry Fine-mapping Decision (MFD)

Motivated by the complementary strengths and limitations observed across methods in our benchmarking analyses, we developed MFD as an empirical decision framework that selects between existing fine-mapping strategies according to locus-level summary-statistic and variant-overlap characteristics. Rather than introducing a new statistical model, MFD provides a practical rule for choosing between a joint modeling multi-ancestry approach, MESuSiE, and post-hoc integration of single-ancestry results, SuSiE post-hoc (Fig. S22). The rule was implemented for K ancestry inputs and can therefore be applied to both two-ancestry and three-ancestry analyses.

For K ancestry inputs, shared variants were defined as variants available across all included ancestries. Variants outside this K-way shared set were treated as ancestry-specific relative to the K-ancestry analysis. For K > 2, this definition does not require a variant to be unique to one ancestry. For example, in a three-ancestry analysis, a variant present in two ancestries but absent from the third is still considered ancestry-specific because it is not available in the full three-way shared variant set. Thus, adding ancestries makes the shared variant definition more restrictive and increases the fraction of variants classified as ancestry-specific.

First, we assessed whether significant ancestry-specific variants were present within each locus. We defined a significant ancestry-specific variant as a variant outside the K-way shared set that achieved genome-wide significance ($P < 5 \times 10^{-8}$) in at least one ancestry where it was observed. If no significant ancestry-specific variants were detected, suggesting a predominantly cross-ancestry or null signal, MFD selected MESuSiE to leverage its increased power for modeling shared causal architecture across ancestries.

If significant ancestry-specific variants were present, we evaluated their LD patterns to distinguish true ancestry-specificity from differential tagging of cross-ancestry signals. For each significant ancestry-specific variant, we searched for a cross-ancestry proxy variant in high LD with that variant. A cross-ancestry proxy was considered adequate only if the squared correlation between the ancestry-specific variant and the proxy was at least 0.6 in every ancestry where the ancestry-specific variant was genome-wide significant. If a significant ancestry-specific variant lacked such a high-LD proxy, MFD selected SuSiE post-hoc to avoid overlooking potentially ancestry-specific segregating variants.

Finally, for ancestry-specific variants with high-LD cross-ancestry proxies, we assessed the consistency of the signal across ancestries. We required the proxy to show genome-wide significance ($P < 5 \times 10^{-8}$) across all included ancestries. If all significant ancestry-specific variants were adequately tagged by globally significant proxies, MFD selected MESuSiE, interpreting the ancestry-specific variants as likely tags of cross-ancestry causal signals. Conversely, if any significant ancestry-specific variant lacked a high-LD proxy with consistent genome-wide support across ancestries, MFD selected SuSiE post-hoc. This conservative rule reduces the risk of discarding potentially ancestry-specific segregating variants when variant overlap across ancestry inputs is incomplete.

General Simulation Framework and Extensions

We first randomly sampled 100 genomic regions from 266 candidate loci showing genome-wide significant associations in the Psychiatric Genomics Consortium GWAS of six neuropsychiatric disorders [11–16]. Candidate regions were identified by selecting SNPs that reached genome-wide significance ($P < 5 \times 10^{-8}$). For each significant SNP, we defined a 1-Mb region centered on it (±500 kb up and downstream) and merged the overlapping genomic regions. The length of the sampled genomic regions varied from 0.51 Mb to 2.56 Mb (mean = 1.09 Mb; median = 1.24 Mb),

with 575 to 5,648 SNPs per region (mean = 2,038 SNPs; median = 2,190 SNPs). For each genomic region in turn, we obtained genotypes from a randomly selected $\tilde{n}_{WB}$ = 50,000 White British individuals and $\tilde{n}_{BB} =$ 6,374 Black British individuals from UK Biobank to serve as the reference, with which we further simulated summary statistics in the two ancestries for GWAS with a targeted sample size of n_{WB} and n_{BB}, respectively.

We denote the SNP effect sizes for all SNPs in the genomic region as $\boldsymbol{\beta}_{WB}$ and $\boldsymbol{\beta}_{BB}$ for White British and Black British. We allow $\boldsymbol{\beta}_{WB}$ and $\boldsymbol{\beta}_{BB}$ to vary across simulation scenarios to reflect different genetic architectures, as described in detail below. Each effect-size vector was an m_c dimensional vector, where m_c is the number of SNPs present in both ancestry groups, with non-zero elements for the causal variants and zero elsewhere. For each ancestry, we normalized the SNP genotype matrices to have mean zero and variance one column-wise, and computed the corresponding LD matrices $\mathbf{R}_{WB}$ and $\mathbf{R}_{BB}$ for White British and Black British. To generate marginal effect size estimates consistent with large-scale GWAS, we adopted the simulation framework of [1]. Using reference panels of sample size of $\tilde{n}_{WB}$ and $\tilde{n}_{BB}$, we began by computing the scaled estimates as follows:

$$\widehat{\boldsymbol{\beta}}_{\boldsymbol{WB}} = \mathbf{R}_{WB}\boldsymbol{\beta}_{WB} + \boldsymbol{\epsilon}_{WB},$$

$$\widehat{\boldsymbol{\beta}}_{\boldsymbol{BB}} = \mathbf{R}_{BB}\boldsymbol{\beta}_{BB} + \boldsymbol{\epsilon}_{BB},$$

where $\boldsymbol{\epsilon}_{WB}$ and $\boldsymbol{\epsilon}_{BB}$ are the two m_c-vectors of estimation errors in the White British and Black British, respectively. To simulate the estimation errors $\boldsymbol{\epsilon}_{WB}$ and $\boldsymbol{\epsilon}_{BB}$, we first simulated null phenotypes, denoted as $\tilde{y}_{WB} \sim N(0,1-h^2_{WB})$ and $\tilde{y}_{BB} \sim N(0,1-h^2_{BB})$, regressing them on the SNP genotypes to obtain the marginal SNP effect size estimates $\widetilde{\boldsymbol{\beta}}_{WB}$ and $\widetilde{\boldsymbol{\beta}}_{BB}$ for the White British and Black British populations, respectively. These estimates were subsequently rescaled according to the ratio of reference to target sample sizes to approximate the estimation errors that would be observed in GWAS with larger cohorts: $\boldsymbol{\epsilon}_{WB} = \sqrt{\frac{\tilde{n}_{WB}}{n_{WB}}} * \widetilde{\boldsymbol{\beta}}_{WB}$ and $\boldsymbol{\epsilon}_{BB} = \sqrt{\frac{\tilde{n}_{BB}}{n_{BB}}} * \widetilde{\boldsymbol{\beta}}_{BB}$. Finally, we computed the corresponding marginal Z-scores as: $\boldsymbol{z}_{WB} = \frac{\widehat{\boldsymbol{\beta}}_{\boldsymbol{WB}}}{\widehat{\boldsymbol{se}}_{\boldsymbol{WB}}}$ and $\boldsymbol{z}_{BB} = \frac{\widehat{\boldsymbol{\beta}}_{\boldsymbol{BB}}}{\widehat{\boldsymbol{se}}_{\boldsymbol{BB}}}$, where $\widehat{\boldsymbol{se}}_{\boldsymbol{WB}}$ and $\widehat{\boldsymbol{se}}_{\boldsymbol{BB}}$ are m_c-vectors with constant entries $\sqrt{\frac{1}{n_{WB}}}$ and $\sqrt{\frac{1}{n_{BB}}}$, respectively, and both n_{WB} and n_{BB} were set to be 300,000 in simulation by default.

Ancestry-specific SNP scenario

We further considered an ancestry-specific SNP scenario to account for population-specific variant availability in multi-ancestry GWAS summary statistics, which arise due to differences in allele frequency, imputation quality, and quality-control procedures across populations. Specifically, we examined three settings: (i) ancestry-specific variants without causal effect, (ii) ancestry-specific variants with causal effect (mimicking AS-SVs), and (iii) ancestry-specific variants arising from additional incompleteness of LD reference panel coverage.

First, we assessed the impact of ancestry-specific SNP involving only non-causal variants. Using the same 100 genomic regions and simulated effect sizes as in the genetic architecture driven scenario, we randomly masked 30% of non-causal variants in the GWAS summary statistics of either one or both ancestries. We selected the 30% masking level for two reasons. First, this proportion matches that used in the MESuSiE supplementary simulations [[1]], facilitating comparison with a key benchmarked method. Second, 30% reflects the substantial, although asymmetric, differences in variant availability observed between the AFR and EUR datasets in All of Us. Across the 289 matched All of Us loci used in our primary AFR–EUR analysis, variants present in only one ancestry constituted a mean of 41.6% and a median of 41.0% of the combined variant set. Relative to each ancestry's own variant set, 37.5% of AFR variants were absent from the EUR data, whereas 9.3% of EUR variants were absent from the AFR data. Thus, the 30% setting was intended as a standardized simulation condition rather than as an estimate of a universal ancestry-specific missingness rate. All causal variants were retained in this setting.

We next extended this framework to settings involving ancestry-specific variants with causal effect (mimicking AS-SVs), in which causal variants are present in the GWAS summary statistics of only a subset of ancestries. Using the same 100 genomic regions, we designated one cross-ancestry causal variant per region and set its effect size to achieve a regional heritability of either 10^{-4} or 2×10^{-4}. We then restricted the availability of the causal variant to either one or both ancestries by excluding it from the summary statistics in the remaining ancestry groups. To maintain comparability with settings involving ancestry-specific non-causal variants, we additionally restricted the availability of randomly selected non-causal variants such that the overall proportion of ancestry-specific variants was fixed at 30% in either one or both ancestries.

Finally, we considered the combined ancestry-specific variant availability in the GWAS summary statistics with incomplete coverage of the LD reference panel. Building on the setting above, we further restricted the LD reference by randomly excluding an additional 30% of variants to mimic limited coverage in external reference panels. Under this design, the cumulative proportion of variants unavailable for fine-mapping reached approximately 51% (derived as 30% + 70%*30%) in either one or both ancestries. As before, we examined settings involving ancestry-specific non-causal variants alone as well as settings involving ancestry-specific variants both with and without causal effects, enabling us to quantify the joint impact of summary-statistic and reference-panel missingness on fine-mapping performance.

Input-matched sensitivity analysis design

We performed an input-matched sensitivity analysis to determine whether differences in candidate-variant availability, rather than statistical modeling, explained the better performance of the post-hoc integration approach relative to other approaches in the ancestry-specific SNP scenario. We focused on the scenario in which the true causal variant was excluded from one ancestry but retained in the other, mimicking an AS-SV. In the primary analysis, SuSiE post-hoc used the full variant set available within each ancestry and therefore retained the causal variant in one ancestry. In contrast, joint modeling approaches such as MESuSiE used a harmonized cross-ancestry candidate set that excluded the causal variant.

We reran SuSiE post-hoc with both ancestry-specific analyses restricted to the same harmonized candidate-variant set used by MESuSiE. We retained the same simulated summary statistics, LD matrices, missing-variant masks, SuSiE parameters, and post-hoc integration procedure as the ancestry-specific SNP scenario described above. The comparison between full-input and input-matched SuSiE post-hoc therefore isolates the effect of candidate-variant availability. The comparison between input-matched SuSiE post-hoc and MESuSiE evaluates statistical modeling performance under the same candidate-variant set.

Input-matched sensitivity analysis results

Averaged across the two heritability settings, the variants prioritized by full-input SuSiE post-hoc had a mean absolute LD correlation of 0.986 with the true causal variant and a mean distance of 5.84 kb. Restricting SuSiE post-hoc to the harmonized candidate set reduced its mean LD to 0.922 and increased its mean distance to 21.52 kb. Under the matched candidate set, MESuSiE achieved

modestly higher mean LD than SuSiE post-hoc (0.933 versus 0.922) and a shorter mean distance (18.15 versus 21.52 kb). Thus, the advantage of full-input SuSiE post-hoc disappeared when candidate-variant availability was matched. These results indicate that its advantage in the AS-SV setting primarily reflected retention of the ancestry-specific causal variant, whereas MESuSiE provided a modest modeling advantage when both approaches received the same candidate variants (Fig. S90; Tables S7 and S8).

LD mismatch scenario

We further considered LD mismatch scenarios to evaluate the robustness of fine-mapping methods to discrepancies between the LD structure underlying GWAS summary statistics and that estimated from external reference panels.

First, we examined the impact of sampling-induced LD uncertainty by replacing in-sample LD with external LD reference panels derived from the 1000 Genomes Project Phase 3 (503 European and 661 African samples) [17]. This setting mimics a realistic use case where LD is estimated from a finite external cohort, introducing stochastic deviations from the in-sample LD structure of large biobanks due to limited sample size and subtle cohort differences [18]. Fine-mapping performance was evaluated across the same 100 genomic regions and simulated effect sizes used in the genetic architecture-driven scenario. To assess the impact of LD outlier detection procedures, analyses were performed under three regimes: (1) using perturbed statistics directly ('No Control'), (2) filtering variants based on the Slalom diagnostic [19], and (3) filtering variants using the SuSiE-RSS LD outlier test [20].

Subsequently, we evaluated fine-mapping performance in response to systematic discrepancies between summary statistics and LD by generating artificial outliers, following the protocol established by Zhang et al. [21]. This scenario was designed to mimic structural sources of LD inconsistency, such as unmodeled population substructure or allele alignment errors, which frequently occur in large-scale GWAS meta-analyses [19,22,23]. Using the same 100 realistic genomic regions derived from the UK Biobank, we simulated $n_0 = 6$ causal variants per region, each with a 50% probability of being ancestry-specific and a per-SNP heritability of $h^2 = 2 \times 10^{-4}$. We randomly selected a proportion of variants, $p \in \{0.001, 0.01, 0.1\}$, and multiplied their summary statistic Z-scores by a mismatch factor $\rho \in \{0.5, 0.0, -0.5, -1.0\}$ within each ancestry. We then assessed downstream fine-mapping performance with the mismatched GWAS

summary statistics as input along with in-sample LD matrices, both with and without LD outlier detection procedures.

Admix simulation

Admix simulation design

We developed a local ancestry-aware simulation framework to evaluate multi-ancestry fine-mapping methods in an admixed-cohort setting. For an admixed cohort with two ancestral components, we modeled the phenotype as the combined contribution of local ancestry-specific genotype components:

$$\boldsymbol{y} = \mathbf{X_1}\boldsymbol{\beta}_1 + \mathbf{X_2}\boldsymbol{\beta}_2 + \boldsymbol{\epsilon},$$

where $\mathbf{y}$ is the standardized phenotype vector for N individuals, $\mathbf{X_1}$ and $\mathbf{X}_2$ are the local ancestry-specific genotype dosage matrices for ancestry components 1 and 2, $\boldsymbol{\beta}_1$ and $\boldsymbol{\beta}_2$ are the corresponding ancestry-specific SNP effect vectors, and $\boldsymbol{\epsilon}$ is the residual error term.

For individual i and SNP j, the local ancestry-specific dosages can be written as:

$$x_{ij1} = x_{ijM} I\big(h_{ijM} = 1\big) + x_{ijP} I\big(h_{ijP} = 1\big),$$
$$x_{ij2} = x_{ijM} I\big(h_{ijM} = 2\big) + x_{ijP} I\big(h_{ijP} = 2\big),$$

where $x_{i,j,M}$ and $x_{i,j,P}$ denote allele counts on the maternal and paternal haplotypes, $h_{i,j,M}$ and $h_{i,j,P}$ denote their corresponding local ancestries, and $I(\cdot)$ is the indicator function. Under this representation, local ancestry-specific association statistics estimated from an admixed cohort are not equivalent to independent summary statistics from homogeneous ancestry cohorts, because they are estimated from the same individuals and phenotype.

Let $\boldsymbol{z}_1$ and $\boldsymbol{z}_2$ denote local ancestry-specific marginal association statistics and let $\mathbf{R}_{ab} = N^{-1}\mathbf{X}_a^{\top}\mathbf{X}_b$ denote the covariance between local ancestry-specific genotype components (a) and (b). Under standardized genotypes and phenotype, the expected local ancestry-specific association statistics are approximately:

$$E[\boldsymbol{z}_1] = \sqrt{N}(\mathbf{R}_{11}\boldsymbol{\beta}_1 + \mathbf{R}_{12}\boldsymbol{\beta}_2),$$

$$E[\boldsymbol{z}_2] = \sqrt{N}(\mathbf{R}_{21}\boldsymbol{\beta}_1 + \mathbf{R}_{22}\boldsymbol{\beta}_2),$$

where $\mathbf{R}_{11}$ and $\mathbf{R}_{22}$ are the within-ancestry LD matrices for ancestry components 1 and 2, and $\mathbf{R}_{12}$ and $\mathbf{R}_{21}$ capture cross-ancestry covariance between the local ancestry-specific genotype components. This formulation illustrates that admixed-cohort fine-mapping requires explicit consideration of local ancestry-specific LD, correlation between local ancestry-specific association statistics, and cross-ancestry or ancestry-specific causal effects.

We simulated 10,000 EUR-AFR admixed individuals with admix-kit [24] using CEU and YRI haplotypes from the 1000 Genomes Project Phase 3 reference panel, restricting variants to HapMap3 SNPs through the admix-kit HapMap3 subset workflow [24]. The cohort was generated under 80% CEU and 20% YRI ancestry over 10 generations. Using the simulated local ancestry calls, we decomposed each haplotype into ancestry-specific genotype dosages and combined maternal and paternal haplotypes into individual-level local ancestry-specific dosage matrices.

We sampled 100 non-MHC genomic regions from a set of African-ancestry GWAS signal regions derived from All of Us summary statistics across eight quantitative traits. Within each region, we extracted SNPs present across the ancestry-specific local ancestry dosage matrices and retained variants with MAF > 1% in both ancestry-specific components. For each region, we simulated one phenotype from the EUR and AFR local ancestry genotype components with regional heritability fixed at 0.005, following the CARMA-X simulation setting [6]. We considered three genetic architectures with 2, 4, or 6 causal SNPs per region. Each causal SNP was independently assigned an EUR-specific, AFR-specific, or cross-ancestry effect configuration with probabilities 0.25, 0.25, and 0.50, respectively, and effect sizes were scaled to the target regional heritability. This design preserves the broad distinction between cross-ancestry and ancestry-specific effects used in the genetic architecture-driven scenario while replacing separate homogeneous ancestry cohorts with a single admixed cohort, in which local ancestry-specific summary statistics are estimated from the same individuals and phenotype.

For each simulated region, we generated local ancestry-specific association statistics and LD matrices from the EUR and AFR local ancestry genotype components. MESuSiE, SuSiEx, XMAP, Paintor, and MultiSuSiE were applied in their standard multi-ancestry input format, using ancestry-specific summary statistics with within-ancestry LD matrices. For association level integration approaches, we followed the implementation described in Supplementary Note 2; in the admixed-cohort simulation, the pooled-LD variant used LD computed from the total admixed genotype

dosage. CARMA-X was supplied with the full in-sample cross-ancestry LD structure so that it could account for the covariance between EUR and AFR local ancestry association statistics estimated from the same admixed individuals. Because the admixed simulation was restricted to variants segregating in both ancestral populations, it contained no ancestry-specific variants. Consequently, the MFD decision rule selected the joint endpoint in every replicate, making MFD numerically identical to MESuSiE in this setting. We therefore report MESuSiE as the corresponding MFD endpoint. Performance was evaluated using PIP calibration, FDR-controlled causal-variant recovery, credible-set size, and credible-set power.

Admix simulation results

Averaged across the three causal variant counts, CARMA-X achieved the highest power at an empirical FDR of 5%. This advantage was consistent with its explicit modeling of covariance between summary statistics estimated for each local ancestry, a feature not incorporated by the other evaluated methods. CARMA-X achieved a mean power of 0.120 for the either ancestry target, compared with 0.113 for the second best MultiSuSiE, and 0.080 for the cross ancestry causal target, compared with 0.073 for MultiSuSiE. It also showed better PIP calibration, particularly for cross ancestry causal effects and either ancestry signals with high PIPs. However, this advantage did not extend to recovery using 95% credible sets. Mean credible set power was 0.181 for CARMA-X, compared with 0.309 for MultiSuSiE and 0.243 for MESuSiE. CARMA-X produced smaller credible set size, with a median size of one variant, compared with four variants for both MultiSuSiE and MESuSiE, reflecting a tradeoff between causal variant recovery and fine-mapping resolution. Paintor achieved the highest credible set power of 0.743, but its median credible set contained 248 variants, indicating substantially lower resolution. These results show that the advantage of CARMA-X was specific to PIP calibration and causal variant recovery under PIP threshold-based FDR control and did not extend uniformly across fine mapping endpoints (Figs. S59–S60; Table S18).

MESuSiE, and therefore MFD, did not lead in this admixed setting: its either-ancestry power ranged from 0.02 to 0.19 at 5% FDR across the causal-variant counts, below both CARMA-X and MultiSuSiE (Table S18). This pattern is consistent with the fact that methods formulated for independent, ancestry-stratified summary statistics do not directly model the correlation between local ancestry-specific association statistics estimated from the same admixed individuals. We

therefore interpret this simulation as a stress test and as motivation for extending MFD to admixed data through explicit cross-ancestry covariance modeling, rather than as evidence that the current framework can be applied to admixed cohorts without modification.

Functional annotation sensitivity and Evo2-derived sequence-effect annotation analysis

Simulation design

We performed a systematic annotation-sensitivity simulation using Paintor to evaluate how annotation-informed priors affect fine-mapping performance and how sensitive the method is to prior specification. Paintor was chosen because it incorporates SNP-level annotations directly through an enrichment prior on causal status, providing a natural setting for this evaluation.

For each fine-mapping region, we assigned SNP-level prior inclusion probabilities using an annotation-informed softmax prior. For SNP j, the prior inclusion probability π_j was defined as

$$\pi_j = \frac{\exp\left(\boldsymbol{A}_j^T \boldsymbol{w}\right)}{\sum_{j'=1}^{m} \exp\left(\boldsymbol{A}_{j'}^T \boldsymbol{w}\right)},$$

where $\boldsymbol{A_j} \in \mathbb{R}^d$ is the SNP annotation vector in the region and $\boldsymbol{w} \in \mathbb{R}^d$ is the enrichment weight, and d is the total number of available annotations. We varied the annotation enrichment strength across $w \in \{0,1,2\}$ in simulations, where $w = 0$ corresponds to no annotation enrichment, and $w = 1$ and $w = 2$ represent increasing enrichment of causal variants in annotated SNPs.

The other simulation settings matched the genetic architecture-driven scenario. Within each region, we simulated 2, 6, or 10 causal variants. Half of the causal variants were assigned effects in both ancestries, and the remaining half were assigned ancestry-specific effects, with the nonzero effect present in only one ancestry. All causal variants were present in both ancestry-specific variant sets; the distinction therefore reflects differences in effect patterns rather than ancestry-specific variant availability. Both ancestries had a GWAS sample size of 300,000 and per-SNP heritability of (10^{-4}) or (2×10^{-4}). Causal variants were sampled from the annotation-informed softmax prior, and GWAS summary statistics were generated using the same ancestry-specific LD structure as in the baseline simulations.

We evaluated three Paintor input configurations. The first used no functional annotations, corresponding to a non-annotation-informed model. The second used six informative annotations

that generated the causal enrichment: five functional features following SparsePro [25], including H3K27ac, DNase hypersensitivity sites, H3K4me3, conservation, and nonsynonymous status, together with a scalar sequence-effect score derived from Evo2 [26]. This setting therefore represents a correctly specified low-dimensional annotation prior. The third used a high-dimensional set of 188 annotations, comprising all baselineLF2.2.UKB annotations [27] together with the Evo2 score, embedding the informative features within many annotations of uncertain relevance.

DNA sequence foundation models are trained on large collections of genomic sequences and can be used to predict the potential functional effects of genetic variants from sequence context. Evo2 supports zero shot variant effect prediction by comparing the likelihood of a sequence carrying the alternate allele with that of the corresponding reference sequence [26]. We used this likelihood difference as a scalar SNP level annotation in Paintor rather than using raw high dimensional embeddings. For each SNP, we extracted an 8,192-bp sequence window centered on the focal site from the GRCh37 reference genome and constructed matched reference and alternate sequences. Variants whose reported alleles were inconsistent with both the reference and alternate states in GRCh37 were excluded. Each unique reference and alternate sequence was scored with Evo2-7B, and the sequence-effect score was defined as the alternate-sequence likelihood minus the reference-sequence likelihood. More negative values indicate a larger allele-induced reduction in sequence likelihood and were interpreted as greater predicted deleteriousness.

Because Paintor reports SNP-level posterior inclusion probabilities but does not distinguish cross-ancestry from ancestry-specific causal effects, we evaluated it using an either-ancestry causal indicator, treating a variant as causal if it had a nonzero effect in at least one ancestry. Performance was summarized by FDR-controlled power, median credible-set size, mean credible-set power, and runtime. As in the genetic architecture-driven scenario, this simulation contained no ancestry-specific segregating variants, so MFD reduced to its MESuSiE endpoint and served as an annotation-agnostic baseline alongside the other methods that do not use functional annotations.

Simulation results

In this analysis, we found the benefit of annotation-informed priors depending on the strength and specificity of the annotation signal. Under stronger enrichment ($w = 2$), Paintor with the six informative annotations improved FDR-controlled power from 0.167 without annotations to 0.267,

reduced the median credible-set size from 80 to 34 variants, and increased mean credible-set power from 0.612 to 0.691 (Figs. S76-S77; Table S19). In this setting, annotation-informed Paintor also exceeded the annotation-agnostic methods: FDR-controlled power was 0.267 for Paintor with selected annotations, compared with 0.200 for MFD/MESuSiE, 0.195 for SuSiEx, and 0.205 for XMAP, and mean credible-set power was 0.691, compared with 0.626, 0.618, and 0.556, respectively. Consistent with this, Paintor with the selected annotations most accurately recovered the true functional enrichment of prioritized variants, achieving the lowest mean squared error for the informative regulatory annotations, whereas high-dimensional Paintor was the least accurate; annotation-agnostic MFD/MESuSiE fell between the two and remained stable (Figs. S82–S83).

Under moderate enrichment ($w = 1$), the benefit was smaller. FDR-controlled power increased from 0.170 to 0.190 and the median credible-set size decreased from 85 to 59.5 variants, but mean credible-set power was marginally lower than without annotations, 0.585 versus 0.616 (Figs. S70 –S71; Table S19). In this setting, annotation-informed Paintor did not exceed annotation-agnostic MFD/MESuSiE, whose FDR-controlled power was 0.217.

The high-dimensional configuration of Paintor, which included 188 annotations, performed poorly across enrichment strengths despite containing the same informative annotations. It produced very small credible sets, with a median size of 9 variants, but had lower mean credible-set power, 0.436 under stronger enrichment and 0.402 under moderate enrichment, and failed to achieve nonzero per-variant FDR-controlled power at 5% FDR (Figs. S70–S71 and S76–S77; Table S19). The high-dimensional prior also increased computational cost and instability: median Paintor runtime increased from approximately 2.7–3.0 minutes without annotations and 2.8–3.1 minutes with selected annotations to approximately 5.0 minutes with the high-dimensional annotation set. A small number of loci required extremely long runtimes of up to 599–758 minutes, consistent with convergence difficulties in high-dimensional enrichment models (Table S11a). This behavior is consistent with the documentation [[28]], which recommends limiting the number of annotations used in the enrichment model.

Together, these results support two conclusions. First, annotation-informed priors can improve fine-mapping when annotations are predictive of causal status and the prior is well specified, but they can degrade performance by producing overconfident credible sets, reduced causal-variant recovery, and computational instability when the prior is mis-specified, overly strong, or too high-

dimensional for the method. Second, foundation model-derived annotations should be incorporated in a form compatible with the fine-mapping model. In this analysis, the scalar Evo2 sequence-effect score could be incorporated as a single SNP-level annotation within the selected annotation set, whereas embedding it within a high-dimensional annotation stack eliminated this benefit. These results suggest that representation-derived annotations should be evaluated for calibration, redundancy with existing annotations, computational stability, and impact on credible-set size and causal-variant prioritization before being adopted as fine-mapping priors.

High allelic heterogeneity simulation

High allelic heterogeneity simulation design

We extended the genetic architecture-driven simulation framework to settings of high allelic heterogeneity, with 15 or 20 causal variants per 1-Mb locus, to test whether fine-mapping performance and the relative ranking of methods remained stable as the number of causal signals increased. These densities exceed the 2, 6, or 10 causal variants per region used in the genetic architecture-driven scenario and were designed to approximate the signal densities reported in empirical fine-mapping of highly polygenic traits.

We selected these densities using the number of reported credible sets as a practical proxy for the number of statistically resolvable independent signals per region. In SuSiE-based fine-mapping, each single-effect component corresponds to one credible set, and the maximum number of components, (L), caps the number of independent signals a study can report. Across the Open Targets Genetics platform (15,592 GWAS studies; 227,797 study-region pairs) [[29]], 5.0% of study-region pairs reached this component ceiling (L=10), indicating that the most complex loci likely harbor more independent signals than are directly resolved in routine catalogs. Consistent with this, joint multi-trait fine-mapping with mvSuSiE identified 14 independent credible sets at the PIEZO1/ZFPM1 locus [[30]], and conditional and joint analysis (COJO) of GIANT height data resolved up to 25 conditionally independent signals at ACAN [[31]]. Our settings of 15 and 20 causal variants per 1-Mb region therefore extend beyond typical empirical multi-trait fine-mapping and approach the density of the most complex single-trait loci. They were chosen as a stress-test scenario rather than a typical-case scenario.

The design otherwise matched the genetic architecture-driven scenario. We analyzed the same 100 non-MHC genomic regions using EUR and AFR LD matrices, with GWAS sample sizes fixed at

300,000 individuals per ancestry and per-SNP heritability set to 10^{-4} or 2×10^{-4}. Approximately half of the causal variants were assigned cross-ancestry effects, and the remaining causal variants were assigned ancestry-specific effects, with the effect present in either EUR or AFR. Effect sizes were generated and scaled as in the genetic architecture-driven scenario. All causal variants were present in both ancestry-specific variant sets; the distinction therefore reflects differences in effect patterns rather than ancestry-specific variant availability, and MFD consequently reduced to its MESuSiE endpoint. To match model capacity to the increased number of signals, all methods that accept a preset number of single-effect components were configured with (L=20). Performance was assessed with the same metrics: PIP calibration, FDR-controlled power, credible-set size, and credible-set power.

High allelic heterogeneity simulation results

Increasing allelic heterogeneity substantially reduced fine mapping resolution across all methods (Figs. S84–S89; Table S20). At 15 and 20 causal variants per region, per-variant PIP-based power was low for all methods; for MFD/MESuSiE, either-ancestry power at 5% FDR ranged from 0.07 to 0.13 across the two densities and heritability settings. PIP calibration remained well behaved, indicating that the loss of power reflected the limited statistical resolution achievable when many signals co-occur within a single LD block, rather than posterior miscalibration.

Credible-set metrics showed the same resolution tradeoff. Joint modeling multi-ancestry methods, particularly MESuSiE and MultiSuSiE, together with the SuSiE post-hoc fallback, retained comparatively high credible-set power as heterogeneity increased, but only by returning progressively larger credible sets. Averaged across heritability settings, credible-set power for MESuSiE declined from 0.81 at 2 causal variants to 0.55 at 10 and 0.47 at 20, and MultiSuSiE showed a similar trajectory, 0.80, 0.56, and 0.48. This power was maintained at the cost of resolution: the median credible-set size for MESuSiE grew from 11 variants at 2 causal variants to 96 variants at 20, and for SuSiE post-hoc from 21 to 131 variants over the same range (Fig. S84). Methods that achieved comparable credible-set power did so with even larger sets. For example, Paintor reached a median credible-set size of 278 variants at 20 causal variants, reflecting inflated credible sets rather than preserved precision. This pattern reflects the growing difficulty of resolving causal signals embedded in shared local LD.

Overall, increasing the number of causal variants reduced fine-mapping resolution for every method, whereas the relative ranking of methods remained broadly consistent with the genetic architecture-driven scenario. High allelic heterogeneity therefore represents a shared resolution limit of current fine-mapping methods rather than a method-specific failure. Joint multi-ancestry modeling approaches like MESuSiE remained among the most robust in credible-set power, although their higher power was accompanied by larger credible sets.

MFD endpoint sensitivity analysis

MFD endpoint sensitivity simulation design

We evaluated whether the MFD decision rule was sensitive to the choice of joint multi-ancestry endpoint by replacing the default MESuSiE endpoint with SuSiEx or XMAP while keeping the MFD decision rule and SuSiE post-hoc fallback unchanged. This produced three configurations: MFD-MESuSiE, MFD-SuSiEx, and MFD-XMAP. Each configuration was evaluated in the ancestry-specific variant availability simulation using both in-sample summary statistics and an external LD reference panel.

MFD endpoint sensitivity simulation results

When ancestry-specific variants were non-causal, substituting SuSiEx or XMAP for MESuSiE had little effect on overall causal-variant recovery. Averaged across heritability settings and causal-variant counts, either-ancestry power at 5% FDR was 0.21 for MFD-MESuSiE, 0.21 for MFD-SuSiEx, and 0.20 for MFD-XMAP, with comparable credible-set behavior across the three configurations. This pattern was preserved when in-sample LD was replaced by an external LD reference panel, where either-ancestry power was 0.25, 0.24, and 0.24 for MFD-MESuSiE, MFD-SuSiEx, and MFD-XMAP, respectively (Figs. S92 and S97; Table S21a).

Differences between endpoints were more apparent for ancestry-resolved signal classification. MFD-MESuSiE recovered causal signals with effects across ancestries at more than twice the power of the alternative endpoints, with 5% FDR-controlled power of 0.23 compared with 0.10 for MFD-SuSiEx and 0.08 for MFD-XMAP. It was also the only configuration with nonzero power for ancestry-specific contextual variants (AS-CVs), reaching 0.04 for White British-specific AS-CVs and 0.05 for Black British-specific AS-CVs, whereas both MFD-SuSiEx and MFD-XMAP had zero power for these classes at the same threshold (Table S21a). This difference reflects the posterior quantities returned by each joint endpoint rather than the MFD decision rule itself:

MESuSiE reports ancestry-decomposed PIPs for cross-ancestry and ancestry-specific effects, whereas SuSiEx and XMAP primarily return a single either-ancestry PIP that does not separate cross-ancestry from ancestry-specific signals.

For configurations in which the causal variant was ancestry-specific to a single ancestry, all three MFD variants recovered proxy variants in high LD with the true causal variant. Mean LD correlations between the prioritized proxy and the causal variant ranged from 0.963 to 0.988 across endpoints and heritability settings, with comparable proxy-to-causal distances at lower heritability (Figs. S101–S104; Tables S21b and S21c). Together, these results indicate that the MFD decision rule is portable across joint endpoints for overall causal-variant recovery, while MESuSiE remains the preferred default when ancestry-resolved classification of cross-ancestry and ancestry-specific effects is required.

Bootstrap confidence-interval analysis

Bootstrap confidence-interval analysis design

We quantified sampling uncertainty in the genetic architecture-driven simulation benchmarks using a nonparametric, region-level bootstrap. The benchmark comprised 100 genomic regions in each of six simulation settings, defined by three causal architectures crossed with two levels of variant-specific heritability. For each method and setting, every bootstrap replicate resampled the 100 regions with replacement. For the overall credible-set estimates, we pooled the six settings and resampled the resulting 600 region–setting observations with replacement within each method. Resampling was performed at the region level so that the dependence among variants within a region was preserved.

Within each bootstrap replicate, we recomputed the primary performance metrics. For the credible-set analyses, these were the mean power, the mean FDR, and the median credible-set size. For PIP-based power at the nominal FDR targets of 0.05 and 0.10, we averaged the region-specific ROC curves across the resampled regions and read off power at the observed FDR closest to each target. We generated 1,000 bootstrap replicates for every metric and defined the 95% confidence limits as the 2.5th and 97.5th percentiles of the corresponding bootstrap distribution. Setting-specific and pooled estimates are reported in Tables S22a–S22c.

Bootstrap confidence-interval analysis result

The bootstrap confidence intervals were narrow and preserved the ranking of methods across replicates. Among the joint modeling approaches, the pooled credible-set power was 0.658 for MESuSiE/MFD (95% CI, 0.640–0.676), 0.633 for SuSiEx (0.615–0.652), 0.582 for XMAP (0.564–0.601), 0.663 for MultiSuSiE (0.645–0.679), 0.616 for Paintor (0.595–0.637), and 0.543 for CARMA-X (0.526–0.561). SuSiE post-hoc reached a credible-set power of 0.674 (0.657–0.691), whereas the association-level integration approaches SuSiE-weighted and SuSiE-merged were substantially lower, at 0.371 (0.352–0.390) and 0.335 (0.315–0.356), respectively (Tables S22a and S22b).

We applied the same procedure to FDR-controlled power, defined as the empirical power evaluated under the corresponding nominal FDR-control criterion. At the 0.05 target, power was 0.198 for MESuSiE (95% CI, 0.175–0.219), 0.196 for MultiSuSiE (0.172–0.217), 0.189 for SuSiEx (0.166–0.208) and CARMA-X (0.174–0.214), 0.188 for XMAP (0.169–0.212), 0.174 for SuSiE post-hoc (0.155–0.200), and 0.161 for Paintor (0.144–0.181), whereas the association-level approaches remained low (SuSiE-weighted, 0.061 [0.044–0.077]; SuSiE-merged, 0.000 [0.000–0.012]). These intervals quantify region-to-region variability in method performance while preserving the false-discovery constraint, and the relative rankings were stable across bootstrap replicates (Table S22c).

Empirical credible set coverage analysis

Empirical credible set coverage analysis design

We evaluated the empirical coverage of the reported 95% credible sets in the genetic architecture-driven simulations, treating the simulated causal variants as the known truth. The analysis included the 600 region–setting observations in total, corresponding to 100 genomic regions evaluated under each of six settings defined by three causal architectures and two levels of variant-specific heritability. For each observation, we retrieved the simulated causal variants and evaluated the reported credible sets against this known truth.

Every reported credible set was retained as a separate observation; multiple credible sets from the same region were not collapsed into a single union. For SuSiE post-hoc, credible sets from the ancestry-specific EUR and AFR analyses were evaluated separately. Credible sets for the remaining methods were extracted directly from their respective outputs. Because Paintor did not provide explicit credible-set labels, signal-specific proxy credible sets were constructed from its

raw marginal PIPs using the CARMA LD-based procedure [23]. Candidate absolute-correlation thresholds from 0.50 to 0.99 were evaluated, with variants assigned to a lead-SNP cluster if the threshold was exceeded in either the EUR or AFR LD matrix. For each eligible cluster, the smallest descending-PIP subset with cumulative raw PIP exceeding 0.95 was retained, and its members were removed before evaluating subsequent signals. The threshold producing the largest number of credible sets was selected, with the highest threshold chosen in the event of a tie.

We classified a reported credible set as covered if it contained at least one variant simulated as causal in either ancestry. For each method, empirical coverage was calculated as the total number of covered credible sets divided by the total number of reported credible sets across all region–setting combinations. This per-set coverage measure differs from credible-set power, which quantifies the proportion of simulated causal variants recovered by the union of a method's reported credible sets.

Empirical credible set coverage analysis result

Empirical coverage varied across methods and simulation settings (Tables S2b and S2c). Across the six settings, coverage ranged from 0.908 to 0.977 for MESuSiE, from 0.926 to 0.976 for SuSiE post-hoc, from 0.879 to 0.975 for XMAP, from 0.913 to 0.977 for MultiSuSiE, from 0.905 to 0.977 for SuSiEx, and from 0.947 to 0.994 for CARMA-X. Paintor coverage ranged from 0.637 to 0.720, and coverage ranged from 0.542 to 0.868 for SuSiE-weighted and from 0.224 to 0.512 for SuSiE-merged (Table S2b).

When credible-set counts were pooled across all six settings, most joint modeling approaches achieved empirical coverage above 0.90 (Table S2c). Coverage was 0.933 for MESuSiE (2,107 of 2,258 reported sets), 0.914 for XMAP (1,889 of 2,066), 0.930 for MultiSuSiE (2,139 of 2,299), 0.929 for SuSiEx (1,981 of 2,133), and 0.962 for CARMA-X (2,229 of 2,317). Paintor showed lower coverage of 0.690 (1,782 of 2,581). The SuSiE post-hoc integration approach achieved coverage of 0.942 (2,682 of 2,848). The association-level integration approaches also showed substantially lower coverage: 0.616 for SuSiE-weighted (1,168 of 1,895) and 0.288 for SuSiE-merged (1,019 of 3,534). Overall, most joint modeling approaches and SuSiE post-hoc provided high empirical coverage, whereas Paintor and the association-level integration approaches showed lower coverage. These findings also illustrate that high empirical coverage does not necessarily

imply high fine-mapping resolution, because empirical coverage is influenced by both calibration and credible-set size.

Computation time and memory usage

We evaluated computational efficiency by recording wall-clock runtime and peak memory usage for each method across 100 simulated genomic regions under the genetic architecture driven scenario, yielding 600 total model fits. Runtime was defined as the elapsed wall-clock time for a single run and was quantified for each locus by taking the average of 6 simulated genetic architecture. For R/Python implementations, we measured runtime with native timing utilities, whereas external command-line software was timed using system-level profiling tools. Peak memory usage was defined as the maximum resident memory footprint observed during execution. For R and Python implementations, we monitored peak memory using peakRAM (R) and the *resource* module (Python), respectively; for external software, we extracted the maximum resident set size from system logs. For the association-level integration approach, we did not include the runtime attributable to METAL [10] in the reported timing results. All benchmarking was performed on a Linux-based high performance computing (HPC) cluster using AMD EPYC 7763 64-Core processors at 2.45 GHz.

Genome-wide Association Study in All of Us

We performed GWAS on All of Us Data Release Version 8 short reads whole genome sequencing (WGS) data on three quantitative traits: body mass index (BMI), diastolic blood pressure (DBP) and systolic blood pressure (SBP). We extracted individuals with self-reported ancestry groups as "White" and "Black and African American" from All of Us demographic table and removed individuals whose self-reported ancestry did not match genetically inferred ancestry (White expected to match with European population, Black and African American expected to match with African population). Genetically predicted ancestry was provided in All of Us Data Release Version 8, which were predicted using a random forest classifier trained on reference samples from the Human Genome Diversity Project (HGDP) [32] and the 1000 Genomes Project Phase 3 [17]. We removed related individuals with kinship scores greater than 0.1 and kept individuals with sex at birth as male or female.

For each manually collected physical measurement, within each individual, we took the median of the repeated measurements. For all traits, we iteratively dropped outliers (defined as

individuals more than six standard deviations from the trait mean) until no outliers remained by ancestry group. For DBP and SBP, measures were considered ineligible if they occurred at or after an ICD-9/10 billing code from the groups 585/N18 (chronic kidney disease), 405/I15 (secondary hypertension), or 428/I50 (heart failure). For participants who had started anti-hypertensive medication (ATC codes C02, C03, C07, C08, C09) before the date of their median blood pressure measurement, 15 mm Hg was added to SBP and 10 mm Hg to DBP following [[33]]. Eligible SBP measures were restricted to a range of 30 to 300 mmHg. Eligible DBP measures were restricted to values over 30 mmHg. A rank-based inverse normal transformation was applied for quantitative traits to improve normality and control type I error [[34]].

We conducted GWAS within each ancestry group using REGENIE [[35]], adjusting for covariates including age, sex at birth, age2, sex*age, and sex*age2, ethnicity, and top 16 PCs to account for potential confounding. We then constructed LD matrices using ancestry-matched reference panels from the 1000 Genomes Project Phase 3 with 503 European and 661 African samples. We conducted SNP-level quality control and removed variants with minor allele frequency (MAF) < 1%, Hardy–Weinberg equilibrium $P < 1 \times 10^{-25}$, and genotype call rate < 95% within each ancestry group. The sex concordance and heterozygosity rate QC was done by All of Us upstream genomic data QC process [[36]]. Additionally, we excluded SNPs that were strand ambiguous, multi-allelic, detected inconsistencies between GWAS summary statistics and LD matrices, as well as those located within the major histocompatibility complex (MHC) region (chr6: 25–34 Mb).

We evaluated the functional relevance of fine-mapping results by assessing the enrichment of putative causal variants across major functional annotation categories. Enrichment analyses were conducted across coding and regulatory features, including non-synonymous and synonymous variants, 5′/3′ UTRs, promoters, and Cis-Regulatory Elements (CREs) [[23]]. Non-synonymous annotations were obtained from baselineLF2.2.UKB [[27]], while synonymous and UTR variants were predicted using Ensembl VEP v113.0 (GRCh38/GENCODE v47) based on the most severe consequence [[37]]. Promoter and CRE annotations were extracted from [[38]]. Additionally, we evaluated the enrichment of independent eQTLs from GTEx v8 [[39,40]], focusing on biologically relevant tissues: heart and artery for DBP/SBP, and a panel of 13 brain tissues for BMI to capture central nervous system regulation. Functional enrichment was quantified as the fold enrichment ratio, defined as the proportion of annotated variants among prioritized SNPs divided by the

proportion of annotated variants in the background set. Approximate 95% confidence intervals were constructed using Wilson score intervals for the annotation proportion among prioritized SNPs, scaled by the background annotation rate. The background rate was treated as fixed because the background set was substantially larger than the prioritized set. Statistical significance was assessed using chi-square tests on 2×2 contingency tables, with Fisher's exact test used when any expected cell count was below 5. P-values were corrected for multiple testing using Bonferroni adjustment across methods within each annotation category and prioritization threshold. To assess functional enrichment at different prioritization resolutions, we defined prioritized SNPs using three complementary criteria: (i) all variants within 95% credible sets, (ii) the top 100 SNPs ranked by posterior inclusion probabilities (PIPs) for detecting causal signals in at least one ancestry, and (iii) the top 50 SNPs ranked by PIPs for cross-ancestry and ancestry-specific signals. We ranked each method based on its observed enrichment within each functional category and calculated the average rank across all evaluated annotations.

Supplementary Three-Ancestry Real Data Application

Supplementary three-ancestry real data application design

We extended the real-data fine-mapping analysis to three ancestries using the same three cardiometabolic traits analyzed in the two-ancestry setting: body mass index (BMI), diastolic blood pressure (DBP), and systolic blood pressure (SBP). This analysis combined EUR and AFR GWAS summary statistics from All of Us v8 with East Asian (EAS) GWAS summary statistics from the Taiwan Precision Medicine Initiative (TPMI) [[41,42]]. Effective GWAS sample sizes were trait- and ancestry-specific: approximately 193,600 EUR, 61,200 AFR, and 191,500 EAS individuals for BMI; 161,800 EUR, 52,800 AFR, and 196,200 EAS individuals for DBP; and 161,900 EUR, 52,800 AFR, and 196,300 EAS individuals for SBP (Table S12).

GWAS quality control, SNP-level quality control, locus definition, and LD reference construction in the three-ancestry analysis followed the same procedures described in the main text and Supplementary Note 4, extended to incorporate the EAS ancestry input. Summary-statistic and LD consistency was assessed using SuSiE RSS diagnostics, and flagged variants were removed iteratively. The resulting three-way locus definition produced 334 fine-mapping loci, including 213 BMI loci, 60 DBP loci, and 61 SBP loci, compared with 289 loci in the two-ancestry analysis. The median number of variants in the union set per locus was 3,872.5, whereas the median number

of variants present across all three ancestries was 1,762.5. The mean three-way intersection variant fraction was 44.2%, indicating that the three-ancestry intersection imposed a stricter shared-variant definition than the two-ancestry analysis (Table S12).

Seven fine-mapping approaches were applied to each locus using the same method configurations described in the Supplementary Note 2, with adaptations only where required for the three-ancestry setting. MFD used the K=3 decision rule to select either MESuSiE or post-hoc SuSiE at each locus. MESuSiE, SuSiEx, Paintor, and XMAP were run with EUR, AFR, and EAS ancestry-specific summary statistics and LD matrices. MESuSiE output included ancestry-specific-only PIPs for each of the three ancestries, as well as a globally shared EUR–AFR–EAS PIP. Subset-shared configurations, such as EUR–EAS or AFR–EAS shared signals, were retained in credible-set construction but were not assigned to individual ancestry-specific PIP columns. For XMAP, credible sets were retained when the signal passed purity filtering in at least two of the three ancestry-specific LD matrices. Post-hoc SuSiE was run separately within EUR, AFR, and EAS, and multi-ancestry PIPs and credible sets were aggregated as described in the Supplementary Note 2. SuSiE-meta was applied by first performing fixed-effect meta-analysis across EUR, AFR, and EAS summary statistics, followed by single-population SuSiE fine-mapping of the meta-analyzed statistics. CARMA-X was excluded from this analysis because the current public implementation is structured around two ancestry components, and extending it to K=3 would require separate methodological modification and validation.

Method performance was evaluated using the same signal-recovery, credible-set size, high-PIP variant prioritization, and functional annotation enrichment criteria described in the main text. Because adding EAS data changes the set of available variants, LD proxy structure, and composition of high-PIP variant sets, the three-ancestry enrichment analysis was interpreted as an ancestry-expanded sensitivity analysis of functional enrichment patterns rather than as a direct replication of the two-ancestry enrichment ranking.

Supplementary results for three-ancestry real data applications

Across the 334 three-ancestry loci, the K=3 decision rule selected MESuSiE at 152 loci and post-hoc SuSiE at 182 loci. Routing to post-hoc SuSiE was driven by significant ancestry-specific variants that lacked adequate three-way shared-proxy support: at 144 loci, at least one genome-wide significant ancestry-specific signal lacked a globally significant cross-ancestry proxy, and at

38 loci no high-LD three-way cross-ancestry proxy was available. This higher fallback rate is consistent with the stricter three-way shared-variant definition, with a mean cross-ancestry fraction of 44.2%, under which more loci retain ancestry-specific signals that cannot be represented on the three-way intersection than in the two-ancestry analysis.

Overall fine-mapping recovery was consistent with the two-ancestry analysis (Figs. S105–S110; Tables S13–S17). MFD recovered at least one credible set at 312 of the 334 loci, with a median of 18 credible-set variants per locus. MESuSiE, post-hoc SuSiE, and SuSiEx showed similarly stable recovery, returning credible sets at 302–307 loci with median credible-set sizes of 15–22 variants. XMAP recovered credible sets at 307 loci with a comparable median size of 15 variants. This corresponds to a locus-level recovery rate comparable to the two-ancestry analysis, indicating that adding the EAS input did not substantially reduce fine-mapping sensitivity or resolution (Fig. S105).

Credible-set variants prioritized by MFD carried strong association signals. Averaged across the three traits, the median of the maximum absolute Z-score across ancestries among credible-set variants was 5.6 for MFD, comparable to MESuSiE, post-hoc SuSiE, SuSiEx, and XMAP. By contrast, Paintor credible sets had a much lower median absolute Z-score, reflecting the inclusion of many weak-signal variants in its large credible sets. SuSiE-meta had the highest median absolute Z-score but typically retained only the single strongest variant per recovered locus. Paintor recovered 208 loci with a median credible-set size of approximately 650 variants, whereas SuSiE-meta recovered only 34 loci with a median credible-set size of 1 variant (Fig. S105); these contrasting patterns mirrored those observed in the two-ancestry real-data analysis.

We also repeated the high-PIP functional-enrichment analysis in the three-ancestry setting, quantifying fold enrichment with 95% confidence intervals and chi-square or Fisher's exact tests across coding, regulatory, and eQTL annotations among the top-ranked variants (Figs. S105–S110; Tables S13–S16). MFD-prioritized variants remained strongly and significantly enriched for functional categories. Among the top 100 variants aggregated across the three traits, enrichment was 31.3-fold for nonsynonymous variants (95% CI: 13.5–69.9; $P < 0.001$), 9.0-fold for synonymous variants (95% CI: 4.6–16.8; $P < 0.001$), and 2.0-fold for cis-regulatory elements (95% CI: 1.6–2.6; $P < 0.001$), along with positive enrichment for tissue-specific eQTLs. Because adding EAS data changes the prioritized variants, LD proxy structure, and composition of the high-PIP

variant sets, the relative enrichment ranking across methods varied for some annotation categories. We therefore interpret the cross-method comparison as an ancestry-expanded sensitivity analysis of functional-enrichment patterns rather than as a direct replication of the two-ancestry enrichment ranking.

Lastly, to evaluate computational scalability with the number of ancestries, we compared the two- and three-ancestry settings across the benchmark methods (Table S11b). Among the joint-modeling approaches, MESuSiE showed a 2.04-fold increase in median within-locus runtime, whereas XMAP showed a 1.81-fold runtime increase and the largest increase in peak memory (1.94-fold). More modest runtime increases were observed for SuSiEx (1.50-fold), SuSiE post-hoc (1.33-fold), and Paintor (1.10-fold). SuSiE-meta was essentially unchanged; the median runtime for METAL followed by SuSiE was less than one second in both settings, and the small difference did not represent a meaningful computational speedup. Because MFD adaptively selects either MESuSiE or SuSiE post-hoc, its computational scaling depends on the selected endpoint and is expected to be intermediate between the endpoint-specific scaling profiles. Overall, adding a third ancestry produced method-dependent computational increases, with larger increases for MESuSiE and XMAP and more modest increases for the other evaluated approaches.

Together, these results indicate that the MFD framework can be extended to a three-ancestry real-data setting. The K=3 decision rule appropriately routed loci between the joint and ancestry-preserving endpoints under a stricter shared-variant definition, and overall recovery, credible-set resolution, signal strength, and qualitative method behavior remained broadly consistent with the two-ancestry analysis after adding East Asian summary statistics.

Construction of the Summary Table

To facilitate a direct and intuitive comparison of method performance, we synthesized all evaluation metrics into a summary visualization (Fig. 2) by mapping raw scores onto a uniform scale. For properties where lower values are preferred, including fine-mapping resolution, runtime and peak memory, we applied an inverted min-max normalization calculated following [43]:

$$x_{norm} = \frac{max(x) - x}{max(x) - min(x)},$$

where x represents the vector of raw values for a given metric across all methods, while $max(x)$ and $min(x)$ denote the maximum and minimum values within that vector, respectively. Conversely, for metrics where higher values denote superior performance (e.g., statistical power, functional enrichment scores), we applied standard min-max normalization:

$$x_{norm} = \frac{x - min(x)}{max(x) - min(x)},$$

such that a value of 1 always represents the best performance and 0 represents the worst.

Supplementary References

1. Gao, B. & Zhou, X. MESuSiE enables scalable and powerful multi-ancestry fine-mapping of causal variants in genome-wide association studies. *Nat. Genet.* **56**, 170–179 (2024).
2. Kichaev, G. & Pasaniuc, B. Leveraging Functional-Annotation Data in Trans-ethnic Fine-Mapping Studies. *Am. J. Hum. Genet.* **97**, 260–271 (2015).
3. Yuan, K. *et al.* Fine-mapping across diverse ancestries drives the discovery of putative causal variants underlying human complex traits and diseases. *Nat. Genet.* **56**, 1841–1850 (2024).
4. Rossen, J. *et al.* MultiSuSiE improves multi-ancestry fine-mapping in All of Us whole-genome sequencing data. *Nat. Genet.* **58**, 67–76 (2026).
5. Cai, M. *et al.* XMAP: Cross-population fine-mapping by leveraging genetic diversity and accounting for confounding bias. *Nat. Commun.* **14**, 6870 (2023).
6. Yang, Z. *et al.* Fine-mapping in admixed populations using CARMA-X, with applications to Latin American studies. *Am. J. Hum. Genet.* **112**, 1215–1232 (2025).
7. Wang, G., Sarkar, A., Carbonetto, P. & Stephens, M. A Simple New Approach to Variable Selection in Regression, with Application to Genetic Fine Mapping. *J. R. Stat. Soc. Ser. B Stat. Methodol.* **82**, 1273–1300 (2020).
8. Verma, A. *et al.* Diversity and scale: Genetic architecture of 2068 traits in the VA Million Veteran Program. *Science* **385**, eadj1182 (2024).
9. Kanai, M. *et al.* Insights from complex trait fine-mapping across diverse populations. Preprint at https://doi.org/10.1101/2021.09.03.21262975 (2021).

10. Willer, C. J., Li, Y. & Abecasis, G. R. METAL: fast and efficient meta-analysis of genomewide association scans. *Bioinformatics* **26**, 2190–2191 (2010).

11. Adams, M. J. *et al.* Trans-ancestry genome-wide study of depression identifies 697 associations implicating cell types and pharmacotherapies. *Cell* **188**, 640-652.e9 (2025).

12. Mullins, N. *et al.* Genome-wide association study of more than 40,000 bipolar disorder cases provides new insights into the underlying biology. *Nat. Genet.* **53**, 817–829 (2021).

13. Li, W. *et al.* Genome-wide meta-analysis, functional genomics and integrative analyses implicate new risk genes and therapeutic targets for anxiety disorders. *Nat. Hum. Behav.* **8**, 361–379 (2023).

14. Demontis, D. *et al.* Genome-wide analyses of ADHD identify 27 risk loci, refine the genetic architecture and implicate several cognitive domains. *Nat. Genet.* **55**, 198–208 (2023).

15. Strom, N. I. *et al.* Genome-Wide Association Study of Obsessive-Compulsive Symptoms including 33,943 individuals from the general population. *Mol. Psychiatry* **29**, 2714–2723 (2024).

16. Trubetskoy, V. *et al.* Mapping genomic loci implicates genes and synaptic biology in schizophrenia. *Nature* **604**, 502–508 (2022).

17. The 1000 Genomes Project Consortium *et al.* A global reference for human genetic variation. *Nature* **526**, 68–74 (2015).

18. Benner, C. *et al.* Prospects of Fine-Mapping Trait-Associated Genomic Regions by Using Summary Statistics from Genome-wide Association Studies. *Am. J. Hum. Genet.* **101**, 539–551 (2017).

19. Kanai, M. *et al.* Meta-analysis fine-mapping is often miscalibrated at single-variant resolution. *Cell Genomics* **2**, 100210 (2022).

20. Zou, Y., Carbonetto, P., Wang, G. & Stephens, M. Fine-mapping from summary data with the "Sum of Single Effects" model. *PLOS Genet.* **18**, e1010299 (2022).

21. Zhang, W., Lu, T., Sladek, R., Dupuis, J. & Lettre, G. Robust fine-mapping in the presence of linkage disequilibrium mismatch. Preprint at https://doi.org/10.1101/2024.10.29.620968 (2024).

22. Chen, W. *et al.* Improved analyses of GWAS summary statistics by reducing data heterogeneity and errors. *Nat. Commun.* **12**, 7117 (2021).

23. Yang, Z. *et al.* CARMA is a new Bayesian model for fine-mapping in genome-wide association meta-analyses. *Nat. Genet.* **55**, 1057–1065 (2023).

24. Hou, K. *et al.* Admix-kit: an integrated toolkit and pipeline for genetic analyses of admixed populations. *Bioinformatics* **40**, btae148 (2024).

25. Zhang, W., Najafabadi, H. & Li, Y. SparsePro: An efficient fine-mapping method integrating summary statistics and functional annotations. *PLOS Genet.* **19**, e1011104 (2023).

26. Brixi, G. *et al.* Genome modelling and design across all domains of life with Evo 2. *Nature* **652**, 1349–1361 (2026).

27. Weissbrod, O. *et al.* Functionally informed fine-mapping and polygenic localization of complex trait heritability. *Nat. Genet.* **52**, 1355–1363 (2020).

28. Kichaev, G. *et al.* Integrating Functional Data to Prioritize Causal Variants in Statistical Fine-Mapping Studies. *PLoS Genet.* **10**, e1004722 (2014).

29. Buniello, A. *et al.* Open Targets Platform: facilitating therapeutic hypotheses building in drug discovery. *Nucleic Acids Res.* **53**, D1467–D1475 (2025).

30. Zou, Y., Carbonetto, P., Xie, D., Wang, G. & Stephens, M. Fast and flexible joint fine-mapping of multiple traits via the Sum of Single Effects model. *Nat. Genet.* **58**, 454–462 (2026).

31. Yengo, L. *et al.* A saturated map of common genetic variants associated with human height. *Nature* **610**, 704–712 (2022).

32. Cavalli-Sforza, L. L. The Human Genome Diversity Project: past, present and future. *Nat. Rev. Genet.* **6**, 333–340 (2005).

33. the Million Veteran Program *et al.* Genetic analysis of over 1 million people identifies 535 new loci associated with blood pressure traits. *Nat. Genet.* **50**, 1412–1425 (2018).

34. The All of Us Research Program Genomics Investigators *et al.* Genomic data in the All of Us Research Program. *Nature* **627**, 340–346 (2024).

35. Mbatchou, J. *et al.* Computationally efficient whole-genome regression for quantitative and binary traits. *Nat. Genet.* **53**, 1097–1103 (2021).

36. All of Us Research Program Genomics Principal Investigators. All of Us Genomic Quality Report. *All of Us Research Program User Support*

https://support.researchallofus.org/hc/en-us/articles/29390274413716-All-of-Us-Genomic-Quality-Report.

37. McLaren, W. *et al.* The Ensembl Variant Effect Predictor. *Genome Biol.* **17**, 122 (2016).
38. ReproGen Consortium *et al.* Partitioning heritability by functional annotation using genome-wide association summary statistics. *Nat. Genet.* **47**, 1228–1235 (2015).
39. Lonsdale, J. *et al.* The Genotype-Tissue Expression (GTEx) project. *Nat. Genet.* **45**, 580–585 (2013).
40. The GTEx Consortium *et al.* The GTEx Consortium atlas of genetic regulatory effects across human tissues. *Science* **369**, 1318–1330 (2020).
41. Yang, H.-C. *et al.* The Taiwan Precision Medicine Initiative provides a cohort for large-scale studies. *Nature* **648**, 117–127 (2025).
42. Chen, H.-H. *et al.* Population-specific polygenic risk scores for people of Han Chinese ancestry. *Nature* **648**, 128–137 (2025).
43. Li, H. *et al.* A comprehensive benchmarking with practical guidelines for cellular deconvolution of spatial transcriptomics. *Nat. Commun.* **14**, 1548 (2023).
